# *Tripterygium wilfordii* cytochrome P450s catalyze the methyl shift and epoxidations in the biosynthesis of triptonide

**DOI:** 10.1038/s41467-022-32667-5

**Published:** 2022-08-25

**Authors:** Nikolaj Lervad Hansen, Louise Kjaerulff, Quinn Kalby Heck, Victor Forman, Dan Staerk, Birger Lindberg Møller, Johan Andersen-Ranberg

**Affiliations:** 1 Plant Biochemistry Laboratory, Department of Plant and Environment Sciences, University of Copenhagen, Thorvaldsensvej 40, DK-1871 Frederiksberg C, Denmark; 2 Department of Drug Design and Pharmacology, Faculty of Health and Medical Sciences, University of Copenhagen, Universitetsparken 2, DK-2100 Copenhagen, Denmark

**Keywords:** Natural product synthesis, Secondary metabolism, Metabolic engineering

## Abstract

The diterpenoid triepoxides triptolide and triptonide from *Tripterygium wilfordii* (thunder god wine) exhibit unique bioactivities with potential uses in disease treatment and as a non-hormonal male contraceptives. Here, we show that cytochrome P450s (CYPs) from the CYP71BE subfamily catalyze an unprecedented 18(4→3) methyl shift required for biosynthesis of the abeo-abietane core structure present in diterpenoid triepoxides and in several other plant diterpenoids. In combination with two CYPs of the CYP82D subfamily, four CYPs from *T. wilfordii* are shown to constitute the minimal set of biosynthetic genes that enables triptonide biosynthesis using *Nicotiana benthamiana* and *Saccharomyces cerevisiae* as heterologous hosts. In addition, co-expression of a specific *T. wilfordii* cytochrome *b*_5_ (*Tw*cyt*b*_5_-A) increases triptonide output more than 9-fold in *S. cerevisiae* and affords isolation and structure elucidation by NMR spectroscopic analyses of 18 diterpenoids, providing insights into the biosynthesis of diterpenoid triepoxides. Our findings pave the way for diterpenoid triepoxide production via fermentation.

## Abstract (web summary)

How triptonide is made in the medicinal plant *Tripterygium wilfordii* is largely unknown. Here, the authors report the identification and characterization of a suite of cytochrome P450s and show their function in catalyzing the formation of triptonide from miltriadiene in tobacco and baker’s yeast.

## Introduction

**Fig. 1:**
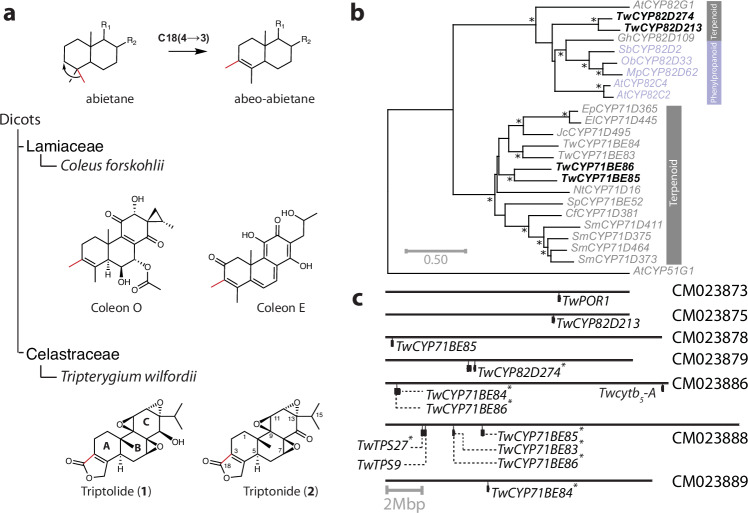
18(4→3) abeo-abietane methyl shift. **a** Hypothesized scheme for the rearrangement of methyl groups on the A-ring of labdane type diterpenoids and examples of diterpenoids with the 18(4→3) abeo-abietane core structure. Examples of labdane type diterpenoids from the Lamiaceae species *Coleus forskohlii* and the Celastracae species *T. wilfordii* with an abeo-abietane core structure are shown. The rings of the tricyclic core structure are denoted A, B, and C. **b** Maximum-likelihood phylogenetic analysis of selected CYP genes from the 71 clan, including plant CYPs from the CYP82 and CYP71 subfamily, and *Nt*CYP51 as the root. Shown CYPs are involved in either phenylpropanoid (purple) or terpenoid (grey) biosynthesis (Supplementary Table 22, Supplementary Fig. 1). *Tw*CYP genes functionally characterized in this work highlighted in bold. Nodes marked * are supported by bootstrap values >85%. **c** Schematic overview of the genome location of the *Tw* genes used for heterologous biosynthesis of triptonide in the *T. wilfordii* genome^6^. Genes marked with * are found as tandem repeats with each copy having on average nucleotide sequence identity of 88.7% to the cDNA sequence of each of the genes used in this paper (Supplementary Table 23). *TwCYP71BE84* and *TwCYP71BE86* homologs are each found in tandem repeats in two different loci of the *T. wilfordii* genome.

The Chinese medicinal plant *Tripterygium wilfordii* (léi gōng téng, Thunder god wine) is currently the exclusive source of the high value diterpenoids triptolide (**1**) and triptonide (**2**)^1^. These unique triepoxide diterpenoids (Fig. 1) have multiple applications as exemplified by the use of triptolide as the active component in new non-lethal rodent pest management products^2^ and the recent identification of triptonide as a promising non-hormonal male contraceptive agent that shows high efficacy, being reversible and safe. Biochemical analyses show that triptonide targets one of the final steps in sperm development resulting in loss of the motility required for egg fertilization and consequently reversible male infertility^3^. Widespread adoption of these novel applications for triepoxide diterpenoids is restricted by the high cost associated with triptolide extraction and purification from *T. wilfordii* or from established plant tissue cultures^4,5^. The establishment of a *Saccharomyces cerevisiae* production platform for *T. wilfordii* derived triepoxide diterpenoids has been hampered by insufficient knowledge about the biosynthetic pathway operating in the host plant^6^.

Diterpenoid biosynthesis in plants is initiated by carbocation mediated carbon rearrangement of geranylgeranyl diphosphate (GGPP) into labdane type diterpenoids catalyzed by diterpene synthases (diTPS)^7^. In contrast to the canonical labdane type diterpenoids, *T. wilfordii* triepoxides harbors an unusual 18(4→3) abeo-abietane core structure (Fig. 1a). No diTPS has been linked to the formation of the abeo-abietane diterpene backbone directly from GGPP and previous studies support that the triepoxide diterpenoids found in *T. wilfordii* are derived from the abietane diterpene miltiradiene^8,9^. Thus, the biosynthesis of triptonide and triptolide must include enzymes that accept miltiradiene as a substrate, and catalyze reactions that can account for the unique positioning of the methyl groups on the A-ring of *T. wilfordii* triepoxides.

Here, we present the identification and characterization of a suite of *T. wilfordii* cytochrome P450s (CYPs) that in the heterologous hosts *N. benthamiana* and *S. cerevisiae* catalyze the formation of triptonide (**2**) from miltiradiene (**3**). To shed light on the biosynthetic processes leading to triptonide accumulation, 13 hitherto undescribed and 5 previously described diterpenoids are isolated and structure elucidated by NMR spectroscopy (Supplementary Figs. 8–53 and Supplementary Tables 1–19). In *S. cerevisiae*, the flux of diterpenoid through the cascade of orchestrated CYP oxygenations is increased by co-expression of a gene encoding a specific cytochrome b5 (cyt*b*_5_). The functional expression of *TwCYP*s and *Tw*cyt*b*_5_ in *S. cerevisiae* constitute the foundation for the proof-of-concept strain for fermentation-based production of *T. wilfordii* derived triepoxide diterpenoids.

## Results

### Identification of *Tw*CYP82s involved in miltiradiene oxygenation

To identify candidate CYPs capable of catalyzing oxygenation of diterpenes in *T. wilfordii*, homology searches were performed in publicly available *T. wilfordii* RNA sequencing data deposits (Supplementary Table 20). More than 61 plant CYPs mainly from the CYP71 and CYP85 clans have been shown to utilize diterpenes as substrate^10,11^ (Supplementary Fig. 1) and genes encoding CYPs from these two clans were used as queries in the homology-based search for candidate genes. In total, 68 candidate *Tw*CYP-encoding transcripts (Supplementary Table 21) were identified and isolated from cDNA derived from root, stem and leaf tissue of *T. wilfordii*^8,12^. Three of these genes have previously been shown to be involved in terpenoid biosynthesis catalyzing key steps in the biosynthetic pathway of celastrol, a high value triterpenoid found in root extracts of *T. wilfordii*^12^.

**Fig. 2:**
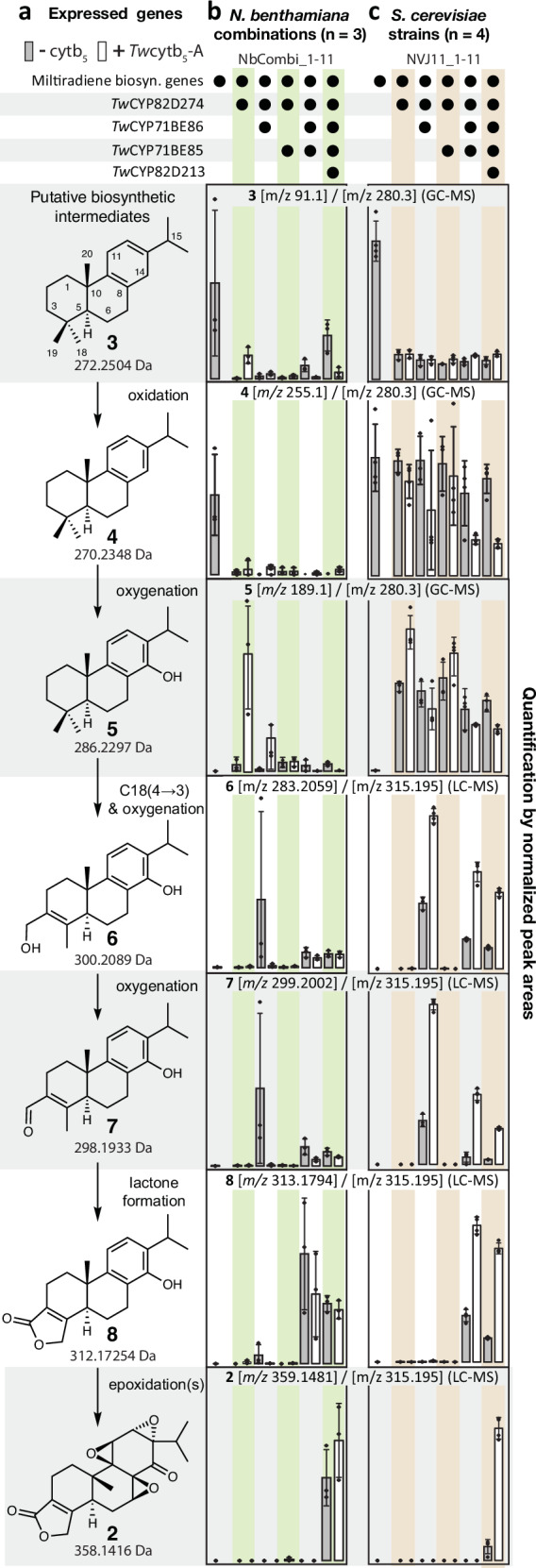
Biosynthesis of triptonide from miltiradiene. **a** Heterologously expressed genes constituting the minimal set of biosynthetic components required for heterologous triptonide biosynthesis. **b** quantity (bars) of putative key intermediates in the biosynthetic pathway of triptonide (right), when established in vivo via heterologous gene expression in *N. benthamian*a. **c**, quantification of intermediates from engineered *S. cerevisiae* (strains listed in Supplementary Table 28). The combination of genes co-expressed with the miltiradiene biosynthetic genes is indicated by black dots. Quantification was based on peak areas of signature *m/z* values for the putative intermediates, normalized to the peak area of signature *m/z* values for the internal standard (IS) used in the GC-MS (compounds **3–5**) and LC-qTOF-MS (compounds **6–8** & **2**). Signature *m/z* values are denoted in the header of each bar diagram. Bars represent the average of *n* = 3 [*N. benthamiana*] or *n* = 4 [*S. cerevisiae*] biological replicates, with error bars representing standard error of the mean (SEM). Values from each replicate is marked by black diamond squares. White- and grey fill color of the bars distinguishes compound quantity in relation to no expression (grey) or co-expression (white) of *Tw*cyt*b*_5_*-A*, respectively. Mass tolerance was ±0.1 *m*/*z* for GC-MS and ±0.005 *m*/*z* for LC-qTOF-MS for signature *m*/*z* values. Source data are provided as a Source Data file.

For *in planta* characterization of the *Tw*CYPs, each of the encoding genes were co-expressed separately in *N. benthamiana* plants^13^ together with *CfTPS1* and *CfTPS3*, that catalyze the formation of **3**^14^, and with GGPP booster genes^15,16^. Combined, these are denoted as the miltiradiene biosynthetic genes. Metabolite extracts of the *N. benthamiana* leaf discs were analyzed by GC-MS and LC-qTOF-MS. Of the 68 candidate *Tw*CYPs co-expressed individually with the miltiradiene biosynthetic genes, co-expression of *Tw*CYP82D274 resulted in the depletion of **3** with an accompanied appearance of 14-hydroxy-dehydroabietadiene (**5**) (Fig. 2, and Supplementary Note 1), 3-epi-triptobenzene B (**13**)^17^ (Supplementary Fig. 29 and Table 9) and a number of additional compounds with monoisotopic masses (detected by LC-qTOF-MS) corresponding to oxygenated diterpenoids (Supplementary Fig. 2). Metabolites **5** and **13** have previously been isolated from *T. wilfordii* root tissue or from tissue cultures along with **1** and **2**^17,18^.

To assess whether enzymes native to *N. benthamiana* contributed to the turnover of miltiradiene-derived diterpenoids, we established an *S. cerevisiae* strain (NVJ0) capable of producing high levels of miltiradiene^15^. Furthermore, to support downstream CYP functionality, the gene encoding CYP reductase *Tw*POR1 (Supplementary Table 23) identified by BLAST searches in *T. wilfordii* transcriptomes, was integrated into the *S. cerevisiae* genome along with the *TwCYP*s. Interestingly, when *TwCYP82D274* was genome integrated in the established *S. cerevisiae* strain, accumulation of **5** but not **13** was observed (Supplementary Fig. 2d), demonstrating consensus for formation of only **5** when comparing the plant and microbial host systems. The products specifically formed in the plant host could reflect intrinsic properties, such as the activity of endogenous enzymes, affecting the direct biosynthetic products of the co-expressed enzymes.

### *Tw*CYP71BE86 and *Tw*CYP71BE85 oxygenate the A-ring of 14-hydroxy-dehydroabietadiene resulting in a C4→C3 methyl shift and lactone formation

We hypothesized that oxygenation of the A-ring of **3** could facilitate the 18(4→3) abeo-abietane methyl shift. Previously, CYPs from the CYP701A, CYP99A, CYP71Z, CYP71BE, and CYP71D subfamilies have been shown to be capable of oxygenating the A-ring of labdane type diterpenoids (Fig. 1 and Supplementary Fig. 1)^10^. *Cf*CYP71D381 from *Coleus forskohlii* has been shown to oxygenate C-2 and C-20 of 13*R*-manoyl oxide when heterologous produced in *N. benthamiana*^19^, but to our knowledge, isolation of neither 2-hydroxy-13*R*-manoyl oxide nor 20-hydroxy-13-*R*-manoyl oxide from the host plant has been reported^20^. On the other hand, a number of abietane and 18(4→3) abeo-abietane diterpenoids have been isolated from *C. forskohlii* (Fig. 1a)^20^. We therefore hypothesized that *Cf*CYP71D381 possibly accepts **3** and that members of the CYP71D subfamily could be involved in catalysis of reactions leading to the 18(4→3) methyl shift found in abeo-abietanes of both *C. forskohlii* and *T. wilfordii*. Accordingly, we tested whether **3** was a substrate for *Cf*CYP71D381. Co-expression of miltiradiene biosynthetic genes^14^ and *CfCYP71D381* in *N. benthamiana* resulted in new metabolites detected in leaf extracts by LC-qTOF-MS. In the mass spectra representing these new metabolites, the monoisotopic masses of likely parental ions supported the identification (<5 ppm) of oxygenated diterpenoids (Supplementary Fig. 3), showing that *Cf*CYP71D381 can efficiently oxygenate **3** *in planta*. With this finding we searched the *Tw*CYP library for *CfCYP71D381* homologs and found four CYP-encoding genes of the CYP71BE subfamily. Recent expansion of the CYP71D subfamily by the identification of new CYP genes have caused the CYP71D subfamily to gain a phylogenetic overlap with the CYP71BE subfamily (Supplementary Fig. 1). These four CYP71BE subfamily encoding genes were selected for further studies.

To test for turnover of **3**, the four candidates *TwCYP71BE83*, *TwCYP71BE84*, *TwCYP71BE85*, and *TwCYP71BE86* were co-expressed individually in *N. benthamiana*, with the miltiradiene biosynthetic genes. Expression of *TwCYP71BE85* and *TwCYP71BE86* resulted in accumulation of oxygenated diterpenoids, with a larger number of new metabolites observed with *TwCYP71BE86* expression in comparison to *TwCYP71BE85* (Supplementary Fig. 3). We then tested whether the biosynthetic products of *Tw*CYP82D274 could be utilized by *Tw*CYP71BE85 or *Tw*CYP71BE86 by co-expressing each of these genes together with *TwCYP82D274*. LC-qTOF-MS analysis of *N. benthamiana* leaf extracts revealed that co-expression of either *TwCYP71BE85* or *TwCYP71BE86* with *TwCYP82D274* resulted in the accumulation of **13** in addition to other polyoxygenated diterpenoids (Fig. 2 and Supplementary Fig. 4). In leaves expressing *TwCYP71BE86*, we also observed formation of low amounts of additional metabolites, the structures of which were identified by NMR analyses or by comparison to authentic standards. One of these were identified as triptophenolide (**8**) (Fig. 2), a putative intermediate in the triptolide pathway that similar to compound **1** and **2** contains the 18(4→3) abeo-abietane core structure, and the lactone ring at the A-ring^18^ (Fig. 1b). Two other metabolites with similar core structures were 18(4→3) abeo-abietatrien-14,18-diol (**6**) and 14-hydroxy-18(4→3) abeo-abietatrien-18-al (**7**) (Fig. 2a, Supplementary Figs. 11–16 and Supplementary Tables 3–4). These results prompted us to co-express *TwCYP71BE85* and *TwCYP71BE86* in *N. benthamiana* leaves. Here we observed a significant increase in the accumulation of **8** (Fig. 2b, Supplementary Fig. 4), suggesting that both encoded enzymes can partake in the methyl rearrangement of the abietane core structure and in formation of the lactone moiety of **8**.

Similar to the observations in the *in planta* expression host, co-expression of *TwCYP82D274*, *TwCYP71BE85*, *TwCYP71BE86* and *TwPOR1* in a miltiradiene producing strain of *S. cerevisiae* resulted in accumulation of **8** (Fig. 2c). However, in contrast to the results obtained using the *N. benthamiana* system, co-expression of *TwCYP82D274* and *TwCYP71BE86* did not result in production of **8** in *S. cerevisiae*. This supports that *TwCYP71BE86* upon expression in *N. benthamiana* as well as in *S. cerevisiae* catalyzes the formation of the 18(4→3) abeo-abietane backbone and that *TwCYP71BE85* expression in *S. cerevisiae* is required for catalysis of lactone ring formation and thus for formation of **8** (Fig. 2b, c).

### *Tw*cyt*b*_5_-A enhances the capacity of *Tw*CYPs to catalyze multiple oxygenations of their diterpenoid substrates

It is well established that cyt*b*_5_ may serve as an additional electron donor in CYP catalyzed reactions^21,22^. To possibly improve the yield of the *Tw*CYP products obtained in *S. cerevisiae*, the genes encoding six different *Tw*cyt*b*_5_’s were expressed individually in *S. cerevisiae* strains together with *TwCYP*s needed for biosynthesis of **8**. Co-expression of *Tw*cyt*b*_5_-*A* resulted in a substantial increase of **8** in the *S. cerevisiae* extracts, while expression of the other *Tw*cyt*b*_5_’s did not have the same effect (Supplementary Fig. 5). Co-expression of *Tw*cyt*b*_5_-A with the different combinations of *TwCYP*s both in *N. benthamiana* and in *S. cerevisiae* (Fig. 2) revealed that the levels of CYP products were predominantly improved in the fungal host. Quantitative data for the accumulation of miltiradiene-derived diterpenoids by transient expression in leaves of *N. benthamiana* showed substantially higher variability across replicates when compared to production in engineered *S. cerevisiae*. This is consistent with the variability observed in other *N. benthamiana* heterologous biosynthesis studies^16^. Still, it is to be noticed that co-expression of *Tw*cyt*b*_5_A and *TwCYP82D274* in *N. benthamiana* increased the accumulation of **5** when compared to *N. benthamiana* only expressing *TwCYP82D274* alone. The opposite was observed in relation to the amount of **8**, **6** and **7** (Fig. 2b) detected in leaf extracts co-expressing *TwCYP82D274* and *TwCYP71BE86*, in which *Tw*cyt*b*_5_*-A* co-expression had a negative impact of accumulation of these compounds. This observation renders it possible that presence of *Tw*cyt*b*_5_-A effect the catalytic rate of *Tw*CYP82D274 and *Tw*CYP71BE86, differently *in planta*^23^. In contrast, the level of all intermediates identified in the *S. cerevisiae* extracts increased with *Tw*cyt*b*_5_*-A* co-expression, with the most pronounced effect seen on the levels of intermediates associated with expression of *TwCYP71BE86* (Fig. 2c).

### *Tw*CYP82D213 completes the biosynthetic pathway for triptonide

The uniqueness of *T. wilfordii* triepoxide diterpenoids as pharmaceutical agents are attributed to the distinct configuration of epoxides on the B- and C-rings (Fig. 1a). These epoxides are essential for bioactivity, e.g., anticancer activity of triptolide towards multiple cancer cell lines^24,25^. CYPs have previously been shown to catalyze epoxide formation in plant specialized metabolism^26^. *Tw*CYP82D274 catalyzed oxygenation of the C-ring in **3**, resulting in accumulation of **5**, but also oxygenations resulting in the accumulation of other miltiradiene derived compounds with more than two oxygens (Supplementary Fig. 2). Accordingly, we hypothesized that *Tw*CYP82D274 in combination with other CYP82D homologs identified from *T. wilfordii* cDNA could catalyze oxygenations leading to the formation of the epoxides on the B- and C-ring of **2**. Among the collection of *Tw*CYP82D encoding genes isolated from *T. wilfordii* cDNA (Supplementary Table 21), *TwCYP82D213* was selected and co-expressed with *TwCYP82D274*, *TwCYP71BE86* and *TwCYP71BE85* in *N. benthamiana* and subsequently in *S. cerevisiae*. In both expression hosts, co-expression of these four *Tw*CYP encoding genes resulted in the accumulation of **2** (Fig. 2).

**Fig. 3:**
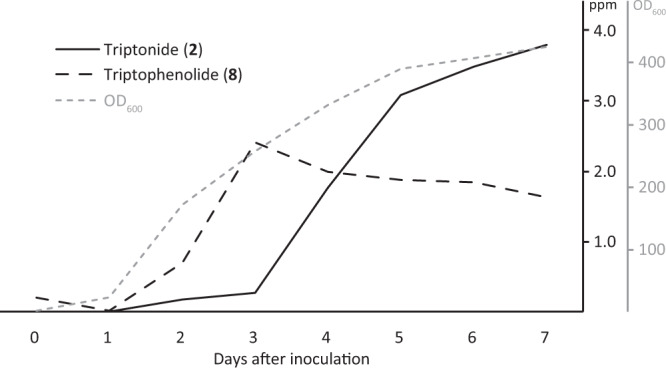
Accumulation in fed-batch fermentation. The accumulation of **8** (dotted black) and **2** (solid black) from NVJ8.5 (Supplementary Table 28) grown in a fed-batch fermentation. Density of the *S. cerevisiae* culture was based on the optical density of daily culture samples.

Based on these findings we conclude that the four *Tw*CYPs together with the miltiradiene biosynthetic genes constitute a minimal set of triptonide biosynthetic genes in *N. benthamiana* and *S. cerevisiae* expression hosts (Figs. 2 and 3).

### The route towards triepoxide formation by *Tw*CYP catalyzed oxygenations in two orthologous biosynthesis hosts

A number of miltiradiene derived products including compounds with monoisotopic masses corresponding to glucosides and glutathione diterpenoid conjugates were identified by LC-qTOF-MS analysis of extracts of *N. benthamiana* leaves expressing the triptonide biosynthetic genes (Supplementary Fig. 6, Supplementary Table 24). Glucosylation and glutathionylation of oxygenated terpenoids heterologously produced in *N. benthamiana* tissues have previously been observed^27,28^ and their accumulation is likely caused by native *N. benthamiana* glucosyltransferases and glutathione *S*-transferases^29^.

From the engineered biosynthetic hosts, 23 GGPP derived compounds were identified, including 18 which were structure elucidated by use of 1D and 2D NMR spectroscopy in this work (Supplementary Figs. 9–53, Supplementary Table 1–20). Besides isocopal-13(16)-en-3,12,15-triol (**19**), labda-8(17),13-*E*−15-*O*-acetate-dien-3-ol (**20**), 14,15-epoxygeranylgeraniol (**21**), and trimethylcyclohexane-11,14-diolgeraniol (**22**) (Supplementary Note 3, Supplementary Figs. 21–53 and Supplementary Tables 15–19), all compounds (including the lactam-diterpenoid (**23**) (Supplementary Note 2, Supplementary Figs. 51–53 and Supplementary Table 19) were considered to be derived from **3** (see below). Of these, **2**, **3**, **13**, **8**, and triptobenzene I (**16**) have previously been isolated from species within the *Tripterygium* genus^18,30,31^ (Supplementary Table 1). Furthermore, 4-epi-triptobenzene J (**14**) and 4-epi-tripquinone C (**18**), diastereomers of compounds found in *Tripterygium* species were identified^30,32^. Hence, a substantial fraction of the molecular diversity of diterpenoid compounds present in *Tripterygium* spp. can be found in the engineered *S. cerevisiae* strain (NJV11.11, Supplementary Table 28) extracts. This demonstrates that the concerted enzymatic capacity of only four *Tw*CYP enzymes enables biosynthesis of a number of abietane/abeo-abietane compounds that constitute a major part of the diterpenoid compound diversity found in the *Tripterygium* species.

### Alternative biosynthetic routes for triepoxide aboe-abietane diterpenoids

In previous work, it has been proposed that dihydroabietic acid is an intermediate in the biosynthetic pathway for abeo-abietanes including the triepoxide terpenoids from *T. wilfordii*^6,18,33^. A possible route for dihydroabietic acid biosynthesis in *T. wilfordii* could be through *Tw*CYP728B70 catalyzed oxygenation of miltiradiene and a subsequent spontaneous oxidation^6^. Here we find that CYPs from the CYP71BE and CYP82D subfamilies are sufficient for triptonide biosynthesis in both *N. benthamiana* and *S. cerevisisae*. Furthermore, none of the miltiradiene-derived compounds identified here carried a carboxyl group at C-18.

### Genomic organization of triptonide specific *Tw*CYP genes from *T. wilfordii*

Recently a high quality chromosome level genome of *T. wilfordii* have become available^6^. To our surprise genes from the *CYP82D* and *CYP71BE* subfamilies could not be identified by BLAST searches on the *T. wilfordii* RNA library based on the genome annotation. However, BLAST searches with the triptonide biosynthetic genes as queries on the assembled genome enabled us to identify the genome position of all (Supplementary Table 23). Interestingly, similar to *TwTPS9* and *TwTPS27*, close homologs of *TwCYP82D274* and *TwCYP71BE86* were found in tandem repeats on chromosome CM023879 and CM23888, respectively (Fig. 1c). The genes encoding *Tw*TPS9 and *Tw*TPS27 involved in miltiradiene biosynthesis^8^ were identified positioned approximately 3 Mbp downstream from the *TwCYP71BE86* tandem repeats (Supplementary Table 23). In contrast, the genome location of, the majority of genes enabling heterologous triptonide biosynthesis reveal that they are situated on different chromosomes in the *T. wilfordii* genome (Fig. 1c).

### *S. cerevisiae* as a heterologous fermentation host for triptonide production

Co-expression of the triptonide biosynthetic genes including *Tw*cyt*b*_5_*-A* in *S. cerevisiae* (NJV11.11) resulted in 0.081 (*n* = 3, SD: 0.004) mg triptonide/L in 0.5 mL cultures using a 96-well plate as fermentation vessel. Substantial quantities of other GGPP derived compounds were also identified as being produced in the triptonide biosynthesizing strains. It remains to be determined which of these may function as intermediates in the triptonide biosynthesis pathway or as shunt products arising from an inefficient, imbalanced or incomplete triptonide pathway (see below).

Based on the established triptonide biosynthetic pathway, we sought to optimize the biosynthetic output by stable integration of additional copies of the triptonide biosynthetic genes in the *S. cerevisiae* genome. Following successful integration of one additional copy of the four *Tw*CYP encoding genes, the yield of triptonide doubled to 0.20 (*n* = 3, SD: 0.06) mg triptonide/L in *S. cerevisiae* (NJV 8.15) extracts, making it our elite strain. To explore the use of *S. cerevisiae* as a production host for **2**, the elite strain was grown in a 1 L fed-batch fermentation with daily monitoring of OD_600_ and quantification of **8** and **2**. The level of **8** reached a maximum of 2.4 mg/L on day 3, whereas the highest level of 3.79 mg/L of **2** was at day 7 (Fig. 3). The reason for the observed differences between the accumulation of the compounds over time is unclear. It could be speculated that **8** is a rate limiting intermediate in the triptonide biosynthetic pathway. OD_600_ level at the end of the fermentation was high as a result of considerable water evaporation occurring during the fermentation run. Still, the fed-batch fermentation of NJV8.5 enabled us to obtain production titers supporting proof-of-concept for *S. cerevisiae* fermentation-based production of **2**.

## Discussion

By co-expression of *T. wilfordii* biosynthetic genes in two orthogonal heterologous host organisms, we demonstrate that four genes from the *CYP71BE* and the *CYP82D* subfamilies constitute a minimal set of genes required for formation of triptonide (**2**) from miltiradiene (**3**). The distinct nature of the two chosen production systems minimizes the likelihood of endogenous enzyme activities in the heterologous hosts being contributors to the formation of **2**. Production in *S. cerevisiae* was more easily scaled and offered a cleaner background for purification of the intermediates selected for NMR analysis and structure elucidation.

In prior work, a majority of the functionally characterized genes from the *CYP82D* subfamily have been associated with enzymes catalyzing steps in flavonoid biosynthesis^11^ (Fig. 1b). No gene from this subfamily has to our knowledge been shown to be involved in oxygenation of diterpenoids^11^. A reaction similar to the one catalyzed by *Tw*CYP82D274 is carried out by *Sm*CYP76AH1 and homologs from Lamiaceae species resulting in formation of 11-hydroxy-dehydroabietadiene from **3** as demonstrated by expression studies in *S. cerevisiae*^34^. However, in many cases it remains to be determined whether conversion of **3** to dehydroabietadiene (**4**) is a spontaneous reaction or whether this happens in conjunction with oxidation of **3** resulting in aromatization of the C-ring (Fig. 2)^35^.

*Tw*CYP71BE86 was shown to catalyze an unprecedented 18(4→3) abeo-abietane methyl shift within the abietane type scaffold found in **3**, **4** and **5** when its encoding gene was expressed in *N. benthamiana* as well as in *S. cerevisiae*. This demonstrates that *Tw*CYP71BE86 catalyzes a carbon rearrangement of labdane type diterpenoids, a highly unusual CYP reaction not previously assigned to any identified plant CYP^36^. Interestingly, *El*CYP71D445 from the phylogenetically overlapping subfamily (Fig. 1b, Supplementary Fig. 1) has been shown in combination with *El*CYP726A27 and *El*ADH1 to catalyze oxygenations facilitating carbon rearrangement of its macrocyclic diterpenoid substrate possibly via an aldol reaction^37^. *Sm*CYP71D375 from the same CYP family has been shown to catalyze the formation of the heterocycle in Tanshinone IIA from miltirone possibly via a P450 mediated carbocation reaction mechanism^38^. Thus, three plant CYPs from the CYP71D/BE subfamily have been shown to be involved in rearrangement of diterpenoid core structures.

**Fig. 4:**
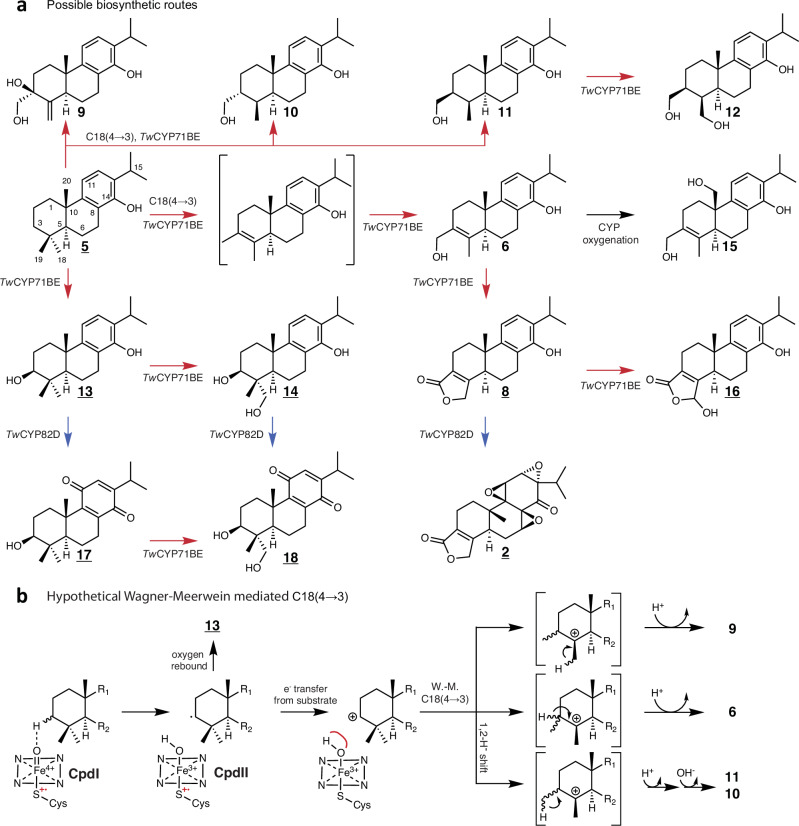
Proposed biosynthetic pathway of triptonide (2) from 14-hydroxy-dehydroabietadiene (5) in *S. cerevisiae*. **a** The biosynthetic pathway from **5** to **2** illustrating the formation of oxygenated miltiradiene derived intermediates catalyzed by the action of *Tw*CYP82’s (blue arrows) and *Tw*CYP71BE’s (red arrows) on the A- and C-ring of the abietane diterpene backbone, respectively. Underlined compounds or diastereomers hereof have previously been identified in plant extracts from *Tripterygium* species (Supplementary Table 1). **b** a hypothetical Wagner-Meerwein rearrangement reaction (W.-M.) to account for the methyl shift of C-18 to C-3 in the abietane carbon backbone. Cpd I and Cpd II represents states of the heme in the CYP catalytic cycle. Red line at heme bound hydroxyl symbolizes inhibition of oxygen rebound facilitating e^-^ transfer as proposed by^43^ for CYP mediated carbocation formation. Compound **13** and oxygenated compounds hereof including **14**, **17**, and **18**, would be products caused by the failure to inhibit oxygen rebound and additional oxygenation likely to be catalyzed by the *TwCYP*s heterolgously expressed in *S. cerevisiae*. Alternatively, the C18(4→3) could be mediated via a mechanism relying on the radical formed by the Compound II (CpdII) state of the involved CYP enzyme (Supplementary Fig. 7).

Carbocation mediated carbon rearrangements are the common mechanism in terpenoid synthase (TPS) catalyzed formation of cyclic terpenoids from prenyldiphosphates^39^. In contrast, the reported additional ability of plant CYPs to catalyze the formation of carbocations mediating terpenoid carbon rearrangements opens up a new route to the formation of complex natural products. Thus, the formation of 5(12)-oxa-3(11)-cyclotaxane upon heterologous expression of *TbCYP725A* from *Taxus brevifolia* in *N. benthamiana*, has been argued to be the result of a carbocation mediated carbon rearrangement of taxadiene^40^. *Tb*CYP725A is part of the CYP85 clan. From the same clan, detailed studies of members of the CYP88A subfamily support that they catalyze ring-contraction of *ent*-kaurenoic acid via formation of a carbocation and a pinacol type rearrangement^41^. The bacterium *Streptomyces arenae* synthesizes the terpenoid pentanolactone. The final step of its biosynthesis proceeds via a CYP161C2 catalyzed formation of a carbocation, resulting in a Wagner-Meerwein type methyl shift and formation of a cyclo-pentene moiety^42,43^. A similar catalytic mechanism has to our knowledge never been reported for plant CYPs. CYP161C2 is distantly related to *Tw*CYP71BE86 but renders it possible that *Tw*CYP71BE86 catalyzes the 18(4→3) abeo-abietane methyl shift using a Wagner-Meerwein type reaction mechanism (Fig. 4b). This is further supported by the observation that the compounds **13**, **6**, **8**, 18 *R*(4→3) abeo-abietatrien-19-ene-14,18,20-triol (**9)**, 18 *R*(4→3) abeo-abietatrien-14,18-diol (**10**), 18-*S-*(4→3) abeo-abietatrien-14,18-diol (**11**), 18 *S*(4→3) abeo-abietatrien-14,18,20-triol (**12**), 18(4→3) abeo-abietatrien-14,18,20-triol (**15**), (Fig. 4a, b) were identified in extracts of *S. cerevisiae* expressing the genes encoding *Tw*CYP82D274 and *Tw*CYP71BE86. Compounds **9-12** all lack the C-3(C-4) double bond which could possibly cause stereochemical constraints of the C-3 and C-4 methyl’s preventing lactone formation (Fig. 4a). Assuming that the 18(4→3) abeo-abietane methyl shift is facilitated by a *Tw*CYP71BE86 catalyzed formation of a carbocation, these compounds could be considered shunt products from an early quenching of the carbocation leading to a failure of establishing the C-3(C-4) double bond and the lactone (Fig. 4b). In this scenario *Tw*CYP71BE86 would go through multiple catalytic cycles, by initially catalyzing formation of the carbocation of C-3 causing the 18(4→3) abeo-abietane methyl shift and subsequent oxygenations of C-18 resulting in the formation of **6**, and possibly also **9**, **10**, **11**, and **12**. If *Tw*CYP71BE86 utilizes a mechanism similar to CYP161C2^43^, failure to inhibit the O-rebound at the *Tw*CYP71BE86 active site could explain the formation of **13**, that subsequently is oxygenated causing the accumulation of **14**, **17**, and **18** (Fig. 4a).

Cationic rearrangements facilitated by CYP catalysis have previously been suggested^44^ and substantial experimental support has been provided in^43^ to show that CYPs can catalyze a Wagner-Meerwein shift. Nevertheless, it cannot be completely excluded that the C18(4→3) shift proceeds via a free radical formed at CpdII (Supplementary Fig. 7), or that both cationic and free radical based reactions are occurring in parallel. If so, this may represent an intriguing evolutionary strategy offering the opportunity to increase the structural diversification of natural products in the plant kingdom by the ability of some enzymes to convert a specific substrate into different products by using different types of catalytic reaction mechanisms.

Out of the five *T. wilfordii* cytochrome *b*_5_ enzymes tested, only *Tw*cyt*b*_5_-A stimulated the activity of *Tw*CYP71BE86 in *S. cerevisiae*^45^. A similar cyt*b*_5_ mediated stimulation has been observed in artemisinic acid biosynthesis catalyzed by *Aa*CYP71AV1. While data on appropriate POR/cyt*b*_5_ expression ratios for heterologous biosynthesis of plant diterpenoids in *S. cerevisiae* has not been reported^45^, we hypothesize that differences in expression ratios could explain the disparate effects of *Tw*cyt*b*_5_-A expression in *S. cerevisiae* and *N. benthamiana*. In cyt*b*_5_ titration studies with mammalian CYPs, CYP activity was inhibited at specific ratios^46^. Alternatively, or in addition, differences in ER membrane properties, native POR and cyt*b*_5_ enzymes, or unknown factors in the two distinct hosts used for heterologous triptonide biosynthesis could influence *Tw*cyt*b*_5_-A functionality. The different effects of *Tw*cyt*b*_5_ co-expression show the advantage of using orthogonal systems for in vivo characterization of biosynthetic genes involved in plant specialized metabolism.

The *Tw*CYP71BE86 associated formation of **6** and **7** requires a multiplicity of consecutive catalytic cycles. To facilitate these, electrons from *Tw*POR and/or *Tw*cyt*b*_5_-A need to be readily available. Presence of CYP reducing partners oxidizing NADPH and NADH, respectively, could promote the availability of electrons for an orchestrated series of CYP oxygenations. Failure to complete these concerted oxygenations could account for the accumulation of a number of the diterpenoid products formed in the *S. cerevisiae* triptonide biosynthesis strain.

In this study we demonstrate that biosynthesis of **2** from **3** can be catalyzed by CYPs from the CYP71BE and CYP82D subfamilies involving unique reactions including a methyl shift on the A-ring and multiple epoxidations of the B/C-ring system, respectively. Epoxides are generally highly reactive and typically labile moieties. LC-qTOF-MS analysis of extracts and NMR analysis of purified diterpenoids from *S. cerevisiae* afforded **2** as the only epoxide carrying compound identified. A triepoxide with a conformation of epoxide rings identical to **2** is the only epoxide containing diterpenoid observed in *T. wilfordii* tissue cultures^18^. Thus, derivatives of **3** with one or two epoxide rings have neither been identified in the native plant nor in the heterologous expression hosts described here. Instead, two abietane quinones were identified, whereof **18** and **16** have previously been isolated from *Tripterygium hyoglaucoma* and *T. wilfordii*^32,47^. To achieve efficient formation of the unique triepoxide configuration, *Tw*CYP82D274 and *Tw*CYP82D213 could be required to pass through multiple highly coordinated consecutive catalytic cycles, possibly facilitated by metabolon formation^48^. Intermediates or products escaping the concerted oxygenations including diterpenoids with one or two epoxide rings are possibly less stable compounds that rearrange into quinone diterpenoids. Further studies including evaluation of the stability of mono- and diepoxide abeo-abietane diterpenoids possibly derived by chemical synthesis would be required to clarify some of these questions and hypotheses.

In addition to being organized in metabolons, orchestration of plant biosynthetic pathways may also be controlled at the genome level e.g. by localization of the biosynthetic genes in gene clusters^49^. The enzymes identified here, including close orthologs of *Tw*CYP82D274 and *Tw*CYP71BE86, were found in tandem repeats on the chromosomes CM023878, CM023886 and CM023888 (Fig. 1c and Supplementary Table 23). These tandem repeats could have emerged from gene duplication throughout the evolution of this plant species^50^. Multiple copies of these genes could enable *T. wilfordii* to achieve high levels of the CYP enzymes via simultaneous transcription from multiple biosynthetic genes in the genome. With the diTPS genes encoding the first committed step in biosynthesis of **2** being co-localized on the same chromosome as *TwCYP71BE86/85*, the biosynthetic pathway of **2** in *T. wilfordii* could be considered clustered^51^. Still, while *Tw*CYP71BE86 can accept **3** as substrate (Supplementary Fig. 3) *in planta*, it remains to be determined how the derived products contribute to the biosynthesis of **2** or whether miltiradiene derived products from *Tw*CYP82D274 catalysis are more appropriate substrates. Thus, it is unclear how and in which order the *Tw*CYP71BE86 and *Tw*CYP82D274 contribute to the biosynthetic step(s) following the formation of **3**.

Considering that co-expression of four CYPs in two orthologous host systems resulted in the formation of several miltiradiene derived compounds also observed in *Tripterygium* species, a linear pathway might be considered a too simplistic model for the biosynthesis of **2** in *T. wilfordii*. Instead, broad substrate and product promiscuity of the *Tw*CYPs could suggest that **2** is only one out of many miltiradiene derived products in *Tripterygium* species that emerges from a grid-type biosynthetic pathway with involvement of a limited number of biosynthetic enzymes (Fig. 4a).

To conclude, the identification of CYPs catalyzing unprecedented types of reactions in plant diterpenoid biosynthesis significantly advances our understanding about their diverse catalytic capacities. This finding will guide future efforts in assigning CYPs to biosynthetic pathways with reaction steps not easily explained as classical CYP catalyzed reactions. Diterpenoid scaffold diversity have until now mainly been associated with the catalytic capacity of diTPS while CYPs have been associated with further decoration of the core structures by monooxygenation reactions^52^. Our data demonstrate that plant CYPs may also play a key role in the modification of the basic diterpenoid core structures. In the case of the biosynthesis of **2**, these unique CYPs are harbored within the CYP71BE subfamily possibly catalyzing a Wagner-Meerwein reaction resulting in scaffold diversification of labdane diterpenoids, including formation of the 18(4→3) abeo-abietane backbone essential for biosynthesis of **2**.

By introducing the genes shown to be involved in formation of **2** into yeast, we have established proof-of-concept for an on-demand and scalable production platform for **2** as a replacement for the current plant extraction-based production of this high value triepoxide diterpenoid.

## Methods

### Establishing constructs for high yield miltiradiene biosynthesis in *N. benthamiana*

For enhanced biosynthesis of miltiradiene, and to employ a gene assembly system that was compatible with the EasyCloni system^53^, we designed a new plasmid system for transient expression in *N. benthamiana*. Promoters for the new vector were sourced from literature and obtained via DNA synthesis (pCm9, pSGT^54,55^), from Addgene (pSIM24 – pM24 promoter^56^) or from in-house DNA template (p35S - pLIFE33^57^). A synthetic DNA *Gb*lock was ordered from Integrated DNA Technologies (IDT, USA) containing the terminator tOCS^58^ and t3A terminator^59^ connected by the USER cassette 5´-CAACGGAATGCGTGCGATCGCGTGCATTC-3′. The new *N. benthamiana* expression vector was established by introducing the tOCS - USER cassette - t3A construct into the pLIFE33 backbone amplified with primer pairs *GB*A31 + *GB*A32, by InFusion cloning (Takara Bio, USA). Assembled plasmid was transformed into E. Cloni 10 G cells (Lucigen, USA). Plasmid sequence was verified by Sanger sequencing (Macrogen, South Korea) and named New_pLIFE. Genes selected for transient expression in *N. benthamiana* were cloned into the established plasmids by using methods described in the EasyCloni system. While working on the data presented here, it was shown that transient co-expression of GGPPS and diTPS encoding genes targeted to the cytosol, together with HMGR in *N. benthamiana* provides enhanced diterpenoid accumulation^16^. Employing a similar strategy, we constructed two dual expression constructs by cloning *ScHMGR*, together with a bidirectional promotor and *SpGGPPS* in the New_pLIFE plasmid. On another New_pLIFE plasmid t*CfTPS1*, was assembled with a bidirectional promotor and t*CfTPS3*^60^. The bidirectional promotor consisted of pM24 and Cm9. Combined the four genes in the two expression construct are denoted the miltiradiene biosynthetic genes.

### Isolation of *T. wilfordii* CYP genes and expression in *Nicotiana benthamiana*

*Tripterygium wilfordii* CYP genes (*TwCYP*s) were cloned from cDNA synthesized using the RevertAid First Strand cDNA Synthesis Kit (ThermoFisher) from RNA isolated from either root, stem or leaf material from *T. wilfordii* grown at the green house facilities at the University of Copenhagen. *TwCYP*s were cloned into pLIFE33 or New_pLIFE33 by USER cloning^57^. A complete list of tested *TwCYP*s is provided in Supplementary Table 21.

Full length *TwCYP* genes were transiently co-expressed with the miltiradiene biosynthetic genes in *N. benthamiana* using agrobacteria mediated transfection^13^. Briefly, binary vectors each containing the miltiradiene biosynthetic genes or *TwCYP*s were transformed by electroporation into agrobacteria. Liquid cultures of transformed agrobacteria each containing specific plasmids were mixed for co-expression. Leaf material of *N. benthamiana* co-expressing specific combinations of *TwCYP*s together with the miltiradiene biosynthetic genes was harvested 7 d after agrobacterial infiltration. 1 mL MeOH was added to two leaf disks (Ø = 2 cm). Extraction was done at room temperature at 200 rpm orbital shaking. 200 μL of extract were filtered by using a 0.22 μm 96-well filter plate (Merck Millipore, Darmstadt, Germany) and at stored at 4 °C prior to LC-MS analysis.

### Saccharomyces cerevisiae growth media

YPD media: 20 g/L Bacto™ Peptone, 10 g/L Bacto™ Yeast extract, 20 g/L glucose.

Synthetic complete (SC) meda without uracil: 1.92 g/L Yeast Synthetic Drop-out Media Supplements without uracil (Sigma-Aldrich Co. LLC. Catalog number Y1501), 6.7 g/L Yeast Nitrogen Base Without Amino Acids (Sigma-Aldrich Co. LLC. Catalog number Y0626), 20 g/L glucose. Feed-In-Time (FIT) was based on EnPump200 (Enpresso GmbH), and made according to protocol enclosed with the product. Agar plates: SC media including agar (15 g/L).

Uracil auxotrophy in parent strains was introduced by selecting for lack of URA3 function on agar plates of SC medium without uracil containing also 5-fluoroorotic acid (5-FOA, 0.74 g/L) and uracil (30 mg/L). Yeast transformants were isolated on SC without uracil agar plates.

### Assembly of genetic constructs for *S. cerevisiae* genome engineering

All plasmids were generated by USER cloning^57^. Also, parent vectors named assembler −1, −2 and −3, for simultaneous genome integration of up to six gene constructs, and harboring AsiSI/Nb.BsmI USER-cassettes, were prepared for USER cloning^53^. Primers used for PCR amplification with USER compatible PfuX7 polymerase^61^ are listed in Supplementary Table 25. Vectors used and generated in this work is listed in Supplementary Table 26.

### *S. cerevisiae* strain construction

Parent yeast strain was *S. cerevisiae* S288C (NCYC 3608; National Collection of Yeast Cultures, Norwich, UK). Genotypes and source of strains are listed in Supplementary Table 28. Biosynthesis of the triptonide precursor miltiradiene was established in *S. cerevisiae* as described in ref. 60. Codon optimized versions of *TwCYP71BE85*, *TwCYP71BE86* and *TwCYP82D213* (TWIST bioscience) were used (Supplementary Table 27). Other genes used for *S. cerevisiae* triptonide biosynthesis originated from *T. wilfordii* cDNA.

Constructed yeast strains were made using the lithium acetate transformation method^62^. Parent strains without functional URA3 were made competent by the following procedure: Inoculation from a glycerol stock into 5 ml YPD medium and growing at 30 °C O/N. Then, transfer of 3 mL of O/N culture to 50 mL YPD medium and continued growing for 4–5 h followed by centrifugation at 3500 × *g* for 10 min, then discarding the supernatant. Cells were then ready for transformation after 2 washes in sterile water (1st in 25 mL, 2nd in 1 mL) and resuspension in 0.4 mL of sterile water.

Transformation of competent yeast cells was carried out as follows: Mixes of designated NotI digested plasmids (2 µL of each) were each added 10 µL competent yeast cells and mixed with 60 µL PEG 3350 (50% w/v), 9 µL LiAc (1 M) and 12.5 µL preboiled salmon sperm DNA. The resulting mixes were next incubated at 42 °C for 40 min before cells were collected by centrifugation (2000 × *g* for 5 min) and removal of supernatant. Cells were then resuspended in 100 µL sterile water and spread on SC without uracil agar plates. Isolated transformants appeared as single colonies after 2 d of incubation at 30 °C. Insertion of gene constructs was confirmed by colony PCR, using the gene and construct specific primers found in Supplementary Table 25. For colony PCR, colonies were resuspended in 50 µL 20 mM NaOH and incubated at 99 °C for 15 min. 1 µL colony suspension was used for PCR.

### Extraction and metabolite analysis

Genetically engineered *S. cerevisiae* strain was transferred into 0.5 mL media in a 96-well plate and grown for 3 d at 30 °C with orbital shaking at 350 rpm. For extraction, 0.1 mL of *S. cerevisiae* culture was transferred to 1.5 mL glass vials. 0.4 mL MeOH uHPLC grade was added. *S. cerevisiae* extracts were filtered by using a 0.22 μm 96-well filter plate (Merck Millipore, Darmstadt, Germany) and at stored at 4 °C prior to LC-MS analysis.

### LC-MS analysis

MeOH extracts were analysed using an Ultimate 3000 UHPLC + Focused system (Dionex Corporation, Sunnyvale, CA) coupled to a Bruker Compact ESI-QTOF-MS (Bruker) system. Samples were separated on a Kinetex XB-C18 column (100 × 2.1 mm ID, 1.7 μm particle size, 100 Å pore size; Phenomenex Inc., Torrance, CA) maintained at 40 °C with a flow rate of 0.3 mL min^−1^ and mobile phase consisting of 0.05% (v/v) formic acid in water (solvent A) and 0.05% (v/v) formic acid in acetonitrile (solvent B).

Three LC protocols were used:

LC method 1: 0–0.5 min, 10 % B; 0.5–21 min, linear increase from 10 to 80% B; 21–31 min, to 90% B; 31–34 min, to 100% B; 34–39 min 100% B; 39–40 min linear decrease from 100 to 10% B. Isocratic 20%, B 41–45.5 min.

LC method 2: 0–0.5 min, 20% B; 0.5–11 min, linear increase from 20 to 80% B; 11–20 min, to 90% B; 20–22 min, to 100% B; 22–27 min 100% B; 27–28 min linear decrease from 100 to 20% B. Isocratic 20%, B 28–32 min.

LC method 3: 0–0.5 min, 20 % B; 0.5–9 min, linear increase from 20 to 100% B; 9–11 min, 100% B; 11–11.5 min, linear decrease from 100 to 20% B; 11.5–15 min, 20% B.

Mass spectra were acquired in positive ion mode over a scan range of *m*/*z* 50–1200 with the following ESI and MS settings: capillary voltage, 4000 V; end plate offset, 500 V; dry gas temperature, 220 °C; dry gas flow of 8 L min^−1^; nebulizer pressure, 2 bar; in source CID energy, 0 eV; hexapole RF, 50 Vpp; quadrupole ion energy, 4 eV; collision cell energy, 7 eV. Raw chromatogram data was calibrated using an internal sodium formate standard and subsequently exported as *.mzML format using DataAnalysis 4.3 (Bruker). MZmine ver 2.53 was used for visualizing the LC-MS chromatograms.

### GC-MS analysis

GC-MS analysis was carried out on a Shimadzu GCMS-QP2010 Ultra (Shimadzu Corp.) with an Agilent HP-5MS column (Agilent Technologies) 20 m × 0.18 mm i.d., 0.18 µm film thickness). Hydrogen was used as a carrier gas at a constant linear velocity of 50 cm s^−1^, and the injection volume was 1 µL at 250 °C (splitless mode). The oven program was 80 °C for 2 min, ramp at rate 20 °C/min to 180 °C, ramp at rate 10 °C/min to 300 °C, ramp at rate 20 °C/min to 310 °C, hold for 3 min. Data was stored in.CDF format and processed in MZmine2.

### Relative quantification of miltiradiene derived diterpenoids

Relative compound quantities in yeast cultures were based on normalized peak areas of characteristic ions (data obtained using targeted feature detection in the MZmine2 software). The signal for the following ions were quantified, **3**: *m*/*z* 255 (GC-MS), **5**: *m*/*z* 189 (GC-MS), **13**: *m*/*z* 303.2318, **6**: *m*/*z* 283.2059, **7**: *m*/*z* 299.2002, **8**: *m*/*z* 313.1794, **2**: *m*/*z* 359.1481. For LC-qTOF-MS and GC-MS data a mass deviation of 5 ppm and 100 ppm, respectively, was tolerated.

For LC-qTOF-MS, the peak area of the base peak ion (*m*/*z* 315.1947) for the internal standard andrographolide was used for normalization. Additional data analysis of normalized peak areas were done with Microsoft Excel for Mac Ver. 16.59 (Microsoft Inc.) and SigmaPlot Version 14.5 (Systat Software Inc.).

### Absolute quantifications of 2 and 6 from fed-batch fermentation

Absolute quantifications of **8** (FT65732, CarboSynth) and **2** (FT65197, CarboSynth) were carried out by co-analysis of authentic standards prepared in MeOH and a final concentration of 5 ppm internal standard (andrographolide). Quantification was based on normalized peak area and calculated from the slopes of linear extrapolations of the standards response curve (triptophenolide 0.05, 0.5, 1, 2 ppm; triptonide 0.5, 1, 2, 10, 20 ppm).

### Metabolomics, identification of peaks observed in *N. benthamiana* and *S. cerevisiae* expressing the triptonide biosynthetic genes

LC-MS data from duplicate samples of the negative control and triptonide biosynthesis samples were analyzed using MZmine ver2.53^63^. Briefly, noise level was set to 1.5E4, ADAP Chromatogram builder was used for feature detection, while chromatographic peaks were detected by Chromatogram deconvolution using the local minimum search algorithm. Monoisotopic masses were identified using the Isotope peak grouper with a tolerance of *m*/*z* 0.01 or 10 ppm, while peaks in the *N. benthamiana* and *S. cerevisiae* extract samples, respectively, were aligned using a *m*/*z* and retention time tolerance of 5 ppm and 0.15 min. All peaks not identified in both of the duplicate samples were removed. Peaks exclusively identified the triptonide biosynthesis samples while not appearing in the background samples were retained. Identification of oxygenated diterpenoids or conjugates thereof was based on the molecular formula predicted from the accurate mass of monoisotopic peaks (<5 ppm). Putative monoisotopic peaks resulting from in source fragmentation of diterpenoid conjugates were removed.

### Feed-batch fermentation of engineering *S. cerevisiae* strains for isolation of miltiradiene derived diterpenoids

All engineered *S. cerevisiae* strains were cultivated in 96-deepwell plates using a Feed-In-Time (FIT; m2p-labs)^60^. For isolation and purification of key intermediates in the triptonide pathway selected engineered *S. cerevisiae* strains were cultivated in feed batch fermentor using a 2 L Biostat® A bioreactor (Sartorius AG).

Batch media contained: 55 g/L glucose · H_2_O, 25 g/L (NH_4_)_2_SO_4_, 1.7 g/L MgSO_4_ · 7H_2_O, while feed media consisted of: 880 g/L glucose · H_2_O, 21.6 g/L KH_2_PO_4_, 24.24 g/L MgSO_4_ · 7H_2_O, 8.4 g/L K_2_SO_4_, and 0.672 g/L Na_2_HSO_4_. Batch- and feed salt mixes as well as batch and feed glucose were prepared separately by dissolving components in Milli-Q water and sterilizing by autoclavation. The feeding solution was made by mixing 500 mL of feed glucose with 500 mL of feed salt mix, 10 mL of vitamin mix (0.64 g/L D-biotin, 3 g/L nicotinic acid, 10 g/L thiamin HCl, 4 g/L D-pantothenic acid hemicalcium salt, 8 g/L myo-inositol, 2 g/L pyridoxine HCl), 10 mL microelements (6.7 g/L Titriplex III, 6.7 g/L (NH_4_)_2_Fe(SO_4_)_2_ · 6H_2_O, 0.55 g/L CuSO_4_ · 5H_2_O, 2 g/L ZnSO_4_ · 7H_2_O, and MnSO_4_ · H_2_O), and 1 mL of trace elements solution (1.25 g/L NiSO_4_ · 6H_2_O, 1.25 g/L CoCl_2_ · 6H_2_O, 1.25 g/L, boric acid, 1.25 g/L Kl, and 1.25 g/L Na_2_MoO_4_ · 2H_2_O).

Fed batch fermentation was initiated by addition of a 100 mL starter culture to the reactor tank (with impellers), which in turn was prepared earlier by autoclaving while containing 200 mL Batch glucose and 300 mL Batch salt mix. Also 5 mL vitamin mix, 5 mL micro elements and 0.5 mL trace elements, were added. Cultivation in the bioreactor was started under the following conditions (monitored and automatically controlled): pH = 5, temperature = 30 °C, dissolved oxygen (DO) = 20%. While pH was controlled by feeding of ammonium hydroxide (32%) and sulfuric acid (10 %), dissolved oxygen was controlled by air supply combined with stirring. Also foam levels were adjusted by addition of anti-foam emulsion (35119, Serva Electrophoresis GmbH). After 18 h of initial cultivation in the bioreactor, feeding with Feeding solution at a rate of 1.3% was started. The fermentation process continued for 7 d with daily sampling of the culture.

### Isolation and purification of miltiradiene derived compounds from engineered *S. cerevi*siae strain for NMR analysis

Compounds in this invention were isolated from bioreactor cultured yeast strains NVJ8.15, and NVJ3.10, and structurally elucidated by NMR. The combined ethyl acetate extracts of broth and MeOH-lysed cells (cells:MeOH = 1:4, v/v) were initially dried in presence of Celite S® (06858, Sigma-Aldrich) via rotary evaporation. Compounds were subsequently isolated by successive fractionations using a puriFlash® 5.250 (Interchim, Montluçon, France) instrument with detection by UV absorbance and Evaporative Light-Scattering Detection (ELSD). This was equipped with a s PF-15SIHP-F0025 (C1) column (OV002A, Interchim) for normal phase and a US5C18HQ-100/300 (C2) column (SSP750, Interchim) - for reverse phase separation.

An initial pre-fractionation of the dry mix of Celite S®/crude extract was achieved using column (C1) with loading from a manually packed dry-loading column. Separation was obtained using mobile phases hexane (A) and ethyl acetate (B), a constant flow rate of 15 mL/min, followed by a final washing step with 100% MeOH. Compounds of interest were detected by UV and ELSD and collected. Collected fractions were continuously evaluated by LCMS using LC-MS method 3 and TLC analysis prior to further fractionation or NMR studies. Additional purification of compounds of interest from fractions containing multiple compounds was carried out by an additional normal phase fractionation using C1 or a reverse phase column fractionation using C2.

For reverse phase purification with C2, sample solvents were evaporated using rotor evaporation and the residue resuspended in 2 mL MeOH. Sample was injected directly onto the pre conditioned column C2. Mobile phases for C2 consisted of solvent C: deionized water and solvent D acetonitrile each acidified with 0.05% (v/v) formic acid. A constant flow rate of 32 mL/min was used, with a linear solvent gradient with increasing concentration of solvent D. Compounds of interest were detected by ELSD and UV and collected.

Additional reverse phase purification was done by multiple injections of 100 μL onto a semi-prep Phenomenex Luna 5 µm C_18_(2) 100 Å 250 × 10 mm i.d. (fully porous) (Phenomenex, Inc., Torrance, CA, USA) column on a Shimadzu HPLC (SPD-M20A diode array detector, FRC-10A fraction collector, DGU-20A5 degasser, LC-20AT pump, CBM-20A System controller, CTO-10AS VP column oven, SIL-10AP autosampler). Mobile phase was a linear gradient between C and D with an increasing amount of D going from 50–100%. Compounds of interest were detected by UV absorbance at 210 nm and collected.

### NMR analysis

NMR experiments were acquired on a Bruker Avance III HD 600 MHz NMR spectrometer (^1^H operating frequency 599.85 MHz) equipped with a 5-mm cryogenically cooled DCH cryoprobe optimized for ^13^C and ^1^H or a Avance III 600 MHz spectrometer (^1^H operating frequency 600.13 MHz) equipped with a 1.7-mm TCI cryoprobe (Bruker Biospin, Karlsruhe, Germany). NMR data was recorded in 1.7- or 5 mm tubes in CDCl_3_ or CD_3_OD (Euriso-top, 99.8 atom % D) with temperature equilibration to 300 K, optimization of lock parameters, gradient shimming, and setting of receiver gain, all automatically controlled by Topspin ver. 3.2 or 3.5 and IconNMR ver. 4.7.5 or 5.0.7 (Bruker Biospin, Karlsruhe, Germany). ^1^H and ^13^C chemical shifts were referenced to the residual solvent signals at respectively *δ*_H_ 7.26 ppm and *δ*_C_ 77.16 ppm (CDCl_3_) and *δ*_H_ 3.31 ppm and *δ*_C_ 49.00 ppm (CD_3_OD). 1D ^1^H and ^13^C NMR spectra were acquired with 30° pulses and 64k data points and zero-filled to 128k data points, ^1^H spectra were acquired with a spectral width of 12 kHz, a relaxation delay of 1 s and an acquisition time of 2.7 s. ^13^C spectra were ^1^H-decoupled using the Waltz-16 composite pulse decoupling scheme. 2D homo- and heteronuclear experiments were acquired with 4096 (HMBC), 2048 (DQF-COSY and ROESY), or 1024 (multiplicity edited HSQC) data points in the direct dimension and 256 (DQF-COSY, HMBC and ROESY) or 256/128 (multiplicity edited HSQC) data points in the indirect dimension. 2D NMR data was zero-filled to 1k in F1 and zero-filled to twice the number of points in F2, employing forward linear prediction in F1 (LPBIN = 0). The 2D experiments supported the results from the 1D-experiments and structures of the identified molecules in Supplementary Figs. 8–53 and Supplementary Table 1–19. Processing of NMR data was done using Topspin ver. 4.0.9 (Bruker Biospin, Karlsruhe, Germany).

Optical rotation was conducted on a ADP410 from Bellingham and Stanley, using MeOH as a reference. Column length was 20 cm and pure compound was dissolved in 10 mL of MeOH. See Supplementary Note 4 for optical rotation measurement values and the comments.

### Reporting summary

Further information on research design is available in the Nature Research Reporting Summary linked to this article.

## Supplementary information

Supplementary Information

Reporting Summary

## Source data

Source Data

## Data Availability

Sequence information data for TwCYP71BE85 (accession ON375998), TwCYP71BE86 (accession ON375999), TwCYP82D213 (accession ON376000), TwCYP82D274 (accession ON376001), Twb5-A (accession ON376002), Twb5-B (accession ON376003), Twb5-C (accession ON376004), Twb5-D (accession ON376005), Twb5-E (accession ON376006), Twb5-F (accession ON376007), and TwPOR1 (accession ON376008) are available in the NCBI GenBank. Source data are provided with this paper.
