# Author Correction: *Tripterygium wilfordii* cytochrome P450s catalyze the methyl shift and epoxidations in the biosynthesis of triptonide

**DOI:** 10.1038/s41467-025-62209-8

**Published:** 2025-07-24

**Authors:** Kenneth T. Kongstad, Nikolaj Lervad Hansen, Louise Kjaerulff, Quinn Kalby Heck, Victor Forman, Dan Staerk, Birger Lindberg Møller, Johan Andersen-Ranberg

**Affiliations:** 1 Faculty of Health and Medical Sciences, University of Copenhagen, Copenhagen, Denmark; 2 Plant Biochemistry Laboratory, Department of Plant and Environment Sciences, University of Copenhagen, Thorvaldsensvej 40, DK-1871 Frederiksberg C, Denmark; 3 Department of Drug Design and Pharmacology, Faculty of Health and Medical Sciences, University of Copenhagen, Universitetsparken 2, DK-2100 Copenhagen, Denmark

**Keywords:** Natural product synthesis, Secondary metabolism, Metabolic engineering

Correction to: *Nature Communications* 10.1038/s41467-022-32667-5, published online 25 August 2022

In the version of the article initially published, in Fig. 1, there were errors in the stereochemistry of the epoxides of triptonide (**2**), and the carbonyl in the lactone was misassigned as C-19 instead of C-18. These errors, and the stereochemistry of Coleon O, have now been corrected.

In Fig. 2, the numbering of compounds in the headings above the bar plots in panels b and c was not correct. The compound numbers should correspond to the numbers shown below the structures in the adjacent column. The compound numbers, plus the stereochemistry of triptonide (**2**), have now been corrected.

In Fig. 4, there were errors in the stereochemistry of the epoxides of triptonide (**2**). To ensure the validity of the stereochemistry of triptonide (**2**) in the corrected figure, the compound has been isolated from the genetically engineered yeast established in our publication, enabling acquisition of the 1D and 2D NMR spectra seen in this amendment’s Supplementary Data 1.1–1.4 (1D 1H, 1D 13C, HSQC, HMBC, ROESY) and summarized in Supplementary Data 1.5, confirming the correct stereochemistry.

Misassignment of the methyl groups C-18 and C-19 in Fig. 4 was also corrected to reflect the C-18(4→3) methyl shift. For compound **16** shown in Fig. 4, a stereocenter was incorrectly introduced at C-13. The correct representation of (**2**), (**5**) and (**16**) has been made. Based on the stereochemistry established from the NMR analysis, the stereochemistry of triptolide (**1**) and triptonide (**2**) has also been corrected in Fig. 4.

For comparison, the original Figs. 1, 2 and 4 are available in the Supplementary Information in this amendment. The figures have been updated in the HTML and PDF versions of the article.

## Supplementary information

Supplementary Data 1.1–1.5

Original, uncorrected Figs. 1, 2, 4

