# Supplementary material for: Tripterygium wilfordii cytochrome P450s catalyze the methyl shift and epoxidations in the biosynthesis of triptonide
Source: Nat Commun. 2022 Aug 25;13:5011. doi: 10.1038/s41467-022-32667-5 (PMC9411204; doi:10.1038/s41467-022-32667-5)
Supplement: Supplementary file 1 — Supplementary Information [file 41467_2022_32667_MOESM1_ESM.docx]

Supplementary material for

*Tripterygium wilfordii cytochrome P450s catalyze the methyl shift and epoxidations in the biosynthesis of triptonide*

Hansen et al.

**Supplementary note 1: Identification of 14-hydroxy-dehydroabietadiene (5)**

Co-expression of miltiradiene biosynthetic genes and *TwCYP82D274* in both *S. cerevisiae* and *N. benthamiana* resulted in the accumulation of **5**. In previous studies, ferruginol (11-hydroxy-dehydroabietadiene) has been identified in *T. wilfordii* root extracts by GC-MS analysis ^1^. We noted that the mass spectrum of ferruginol shares extensive similarity with the mass spectrum of **5** (Supplementary fig. 2). Therefore, it is possible that the previous identification of ferruginol is not correct and that the previously identified compound was instead **5**. Similarly, the identification of the compound hinokione in *T. wilfordii* root extracts in the same study is uncertain, since it cannot be excluded that the reported hinokione peak represents 3-oxo-dehydroabietadien-14-ol.

**Supplementary note 2: Lactam-diterpenoid (23) compounds from extract of engineered *S. cerevisiae***

Purification of triptonide biosynthetic intermediates from *S. cerevisiae* resulted in the identification of compound **23**, with its identity confirmed by NMR analysis. **23** was the only nitrogen containing compound isolated. A possible route for the formation of **23** could be through a reaction involving an abeo-abietane derived diterpenoid and ethanolamine, a common metabolite of eukaryotic cells for the biosynthesis phosphatidylethanol

amine (PE) the most common non-bilayer membrane phospholipid ^2^. Interestingly, we were not able to identify **23** in *N. benthamiana* extracts. In *S. cerevisiae* ethanolamine is the substrate of ethanolamine kinase (EKI1) catalyzing the formation of phosphoethanolamine that via the Kennedy pathway ^3,4^, results in the biosynthesis of PE. In **23**, ethanolamine is part of the of the γ-lactam, and it is unclear whether the formation of **23** is catalyzed by an enzyme native to the *S. cerevisiae* expression host or through a spontaneous reaction.

Due to the identification of **23**, we speculate that other diterpenoid-ethanolamine (possibly propanolamine) compounds could be observed in the extracts from *S. cerevisiae* strain NVJ11_11. In total, five compounds not observed in negative control NVJ11_0 with a monoisotopic mass corresponding to the molecular formula (< 5 ppm) of diterpenoid-ethanol(propanol)amine compounds were detected (Supplementary figure 6, and supplementary table 24).

**Supplementary note 3: Isolated diterpenoids compounds not derived from miltiradiene (3)**

Out of 16 compounds that were isolated, purified and identified by NMR analysis, compound **19**-**21** were considered not to be derived from **3** due to lack of the characteristic abietane core structure. Similar to labdane-type diterpenoids, **19** was shown to encompass a tricyclic core structure^5^ featuring an unusual configuration of C-15, C-16, and C-17 on the C-ring (Supplementary figure 41-43 and supplementary table 15). We suggest that **19** is derived from GGPP and is the product of the catalytic capacity of the diTPS and *Tw*CYPs heterologous expressed in NVJ11.11. In contrast, NMR analysis showed that **20**, and **22** had a bicyclic and a monocyclic structure, respectively, while **21** is a dephosphorylated GGPP molecule with an epoxide at C-13 and C-14 (Supplementary figure 47 and supplementary table 17). Formation of the tricyclic triptonide precursor **3** from GGPP in the NVJ11_11 strain is governed by CfTPS1 and CfTPS3 ^6^. Thus, a possible cause for the accumulation of **19-22** could be insufficient capacity of the two diTPS enzymes to complete the cyclization of the available GGPP *in vivo*. The oxygenations, such as the hydroxylation at C-3 in **19-20** are unlikely to be attributed to the reaction mechanism of the diTPSs and likely a product of an oxygenation by the heterologously expressed *TwCYP*s, endogenous *S. cerevisiae* enzymes or a spontaneous reaction. It is however noted that C-3 hydroxylation of **5** is attributed to *Tw*CYP71BE86, but additional experiments are required to determine the substrate promiscuity of this enzymes.

**Supplementary note 4: Optical rotation of purified compounds**

An attempt was made to record optical rotations based on the minute amounts (<1 mg) of diterpenoids purified from yeast expressing the triptonide biosynthetic genes. The amounts isolated of **9** and **16** were 0.15 mg and 0.20 mg, respectively. The optical rotations of **9** and **16** were determined to be -1500° and -2000°, respectively. The low compound amounts available for the measurements, reduce/lower our confidence in the values obtained. The quantity of the other isolated compounds was insufficient for any attempts to determine their optical rotation. The absolute stereochemical configurations determined by the NMR analyses of the miltiradiene derived compounds (Supplementary figure 9-53, and supplementary table 2-19) are supported by the known configuration of **3** when biosynthesized by CfTPS1 and CfTPS3.

#

**Supplementary figure 1: Phylogeny of characterized plant CYPs involved in diterpenoid biosynthesis.** Phylogenic analysis combining isolated *T. wilfordii* CYPs and plant CYPs involved in diterpenoid biosynthesis as described in Bathe et al 2019 ^7^. CYPs assumed to act on similar substrates have been labeled in the same color. The simplified structures of corresponding substrates and the positions of oxygenation (asterisks) are displayed. Positions labeled with asterisks in brackets are oxidized by distant CYPs (*Os*KOL4-5[*Os*CYP701A8-9], *Sp*CYP71BE52 and *Cf*CYP71D381). The recently characterized *Os*CYP71Z2,21-22^8^ and *Zm*CYP71Z16,18^9^ were added to the phylogeny together with CYPs isolated from *T. wilfordii* cDNA (black) (Supplementary table 22). *Tw*CYPs shown to utilize **3** and oxygenated derives hereof as substrate in this work are marked (black, bold).

**Supplementary figure 2: GC-MS analysis of extracts from *N. benthamiana* expressing *Tw*CYP82D274.** GC-MS and LC-qTOF-MS analysis of extracts from *N. benthamiana* expressing miltiradiene biosynthetic genes without (-) and with co-expression of (*TwCYP82D274*). **a**: GC-MS chromatogram. Compound **4** and **3** were detected in the (-), while only **5** and an unknown constituent were detected in the *Tw*CYP82D274 sample. **b**: MS spectra of **4** and unknown constituent/metabolite. Identity of **5** was confirmed by NMR (Supplementary fig. 9-10, and table 2) **c**: LC-qTOF-MS chromatogram [LC-MS method 1]. **d**: Quantification of **13** in *N. benthamiana* and *S. cerevisiae* expressing different combinations of the triptonide biosynthesis genes. Quantification based on normalized peak areas of **13** from LC-qTOF-MS analysis [LC-MS method 3] of *N. benthamiana* and *S. cerevisiae* extracts respectively.

Bars represent the average of n = 3 [*N. benthamiana*] or n= 4 [*S. cerevisiae*] biological replicates. Values from each replicate is marked by black diamond squares. Bars represent the standard error mean +/- SEM of the level of **13** in the biological replicates with either no expression (grey) or co-expression (white) of *Twcyt b_5_-A*, respectively. Source data are provided as a Source Data file.

**Supplementary figure 3: LC-qTOF-MS analysis of extracts from *N. benthamiana* co-expressing *CfCYP71D381*, *TwCYP71BE83*, *TwCYP71BE84*, *TwCYP71BE85*, and *TwCYP71BE86* together with miltiradiene biosynthetic genes.** LC-qTOF-MS analysis of *N. benthamiana* extracts. LC-MS method 1 used. Peaks marked with grey are peaks not observed in the negative control (-). The mass spectra representing marked peaks contains a parental ion corresponding to the monoisotopic mass of an oxygenated diterpenoid molecule.

**Supplementary figure 4: LC-qTOF-MS analysis of extracts from miltiradiene (3) producing *N. benthamiana* co-expressing combinations of *Tw*CYPs from the CYP82D and CYP71BE subfamilies.** LC-qTOF-MS analysis of extracts from *N. benthamiana* expressing combinations of *Tw*CYPs. LC-MS method 1 used. In all combinations, miltiradiene biosynthetic genes were co-expressed with the specific *Tw*CYPs. Compounds **5**, **6**, **7**, **8**, and **13** were identified by their characteristic *m*/*z* [M+H] at retention time 23.1 min, 13.0 min, 15.3 min, 14.8 min and 16.7min, respectively (grey fill). Compound **5** and **13** were detected in all samples except the negative control, while **6-8** only were identified in the *N. benthamiana* expressing *TwCYP82D274* and *TwCYP71BE86*. Identity of **5**, **13**, **6**, **7** was confirmed by NMR (Supplementary figures 9-10, 29, 11-13, 14-16 and Supplementary tables 2, 9, 3, and 4) while **8** was confirmed by an authentic standard.

**Supplementary figure 5: Level of triptophenolide (8) from *S. cerevisiae* strains expressing *Twcyt b_5_-*(*A-F***). LC-qTOF-MS analysis of extracts from *S. cerevisiae* co-expressing *TwCYP82D274*, TwCYP71BE86, and *TwCYP71BE85* together with *Twcyt b_5_*-(*A-F*) identified in *T. wilfordii* transcriptomes (Supplementary table 20). *Twcyt b_5_*-*B*, not included in this analysis. Chromatogram is based on the sum of parental ions specific for internal standard (IS, 5 ppm andrographolide, m/z 351.2125) and **8** (triptophenolide, *m*/*z* 313.1794).

**Supplementary figure 6: LC-MS analysis of *N. benthamiana* and *S. cerevisiae* expressing the triptonide biosynthetic genes.** LC-qTOF-MS analysis [LC- method 1] of extracts from *N. benthamiana* and *S. cerevisisae* expressing the miltiradiene biosynthetic genes (-) and the triptonide biosynthetic genes (*TwCYP82D274* + *TwCYP82D213* +*TwCYP71BE86* + *TwCYP71BE85*), respectively. (**a**), (**c**) Comparison of the extracted ion (*m*/*z* 250.0 - *m*/*z* 800.0) chromatograms from (-) and triptonide biosynthesis samples. Identity of **5** was confirmed by NMR analysis (Supplementary figure 9-10, and supplementary table 2) while **2** and **8** were confirmed by authentic standards. Compounds marked with an asterisk were detected in trace amounts not observable as a resolved peak in the EIC (*m*/*z* 250.000-*m*/*z* 800.000) trace. (**b**), (**d**) 2-D representation of peaks identified in biological replicates of the triptonide biosynthesis sample (n = 2), while not observed in (-) (n = 2). Only peaks represented by mass spectra with monoisotopic *m*/*z* [M+H] corresponding to the molecular mass of C20 or C20 conjugate molecules (>5ppm) are shown (Supplementary table 24). Each putative compound is represented by a grey circle and a number according to its RT (x-axis) and *m*/*z* value (y-axis).

**Supplementary figure 7: Alternative proposal for a free radical mediated mechanism facilitating the C18(4🡪3) methyl shift.** A radical is formed by Compound I (CpdI) state of the CYP. Alkyl migration mediated by a radical is unlikely (See chapter 13 in Nonhebel et al 1973 ^10^). Nevertheless, based on the current knowledge about CYP catalysis such a mechanism can not be completely ruled out. Formation of the 19-ene observed in **9** could be the result of a second CYP catalytic cycle, forming a 2^nd^ radical that when paired with the 1^st^ radical results in double bond formation. Similarly, the C3(4)-ene in **6** and the final product **2** could emerge from a similar mechanism with the 2^nd^ free radical being formed on C3. A possible mechanism for biosynthesis of **11**, and **10**, include a 1,2-H^-^ shift. In organic chemistry, radical 1,2-H^-^ shifts have been observed in aryl compounds, but remain rare ^10,11^. Following the mechanism from **9** and **6**, a double bond could be formed between C3 and C18, which would then be hydrated for the formation of **11** and **10**. Based on the many reactions that are prohibited and rare in radical chemistry, we consider it lees probable that C18(C4🡪C3) is mediated through radical reactions rather than a carbo-cation mediated reaction. Further experimentation is required to determine whether such a mechanism can be completely disregarded for CYP catalyzed methyl shifts.

**Supplementary figure 8. Overview of identified diterpenoids.**

Compound info found in supplementary table 1. NMR data supporting the molecular structures are found in supplementary figure 9-53 and table 2-19.


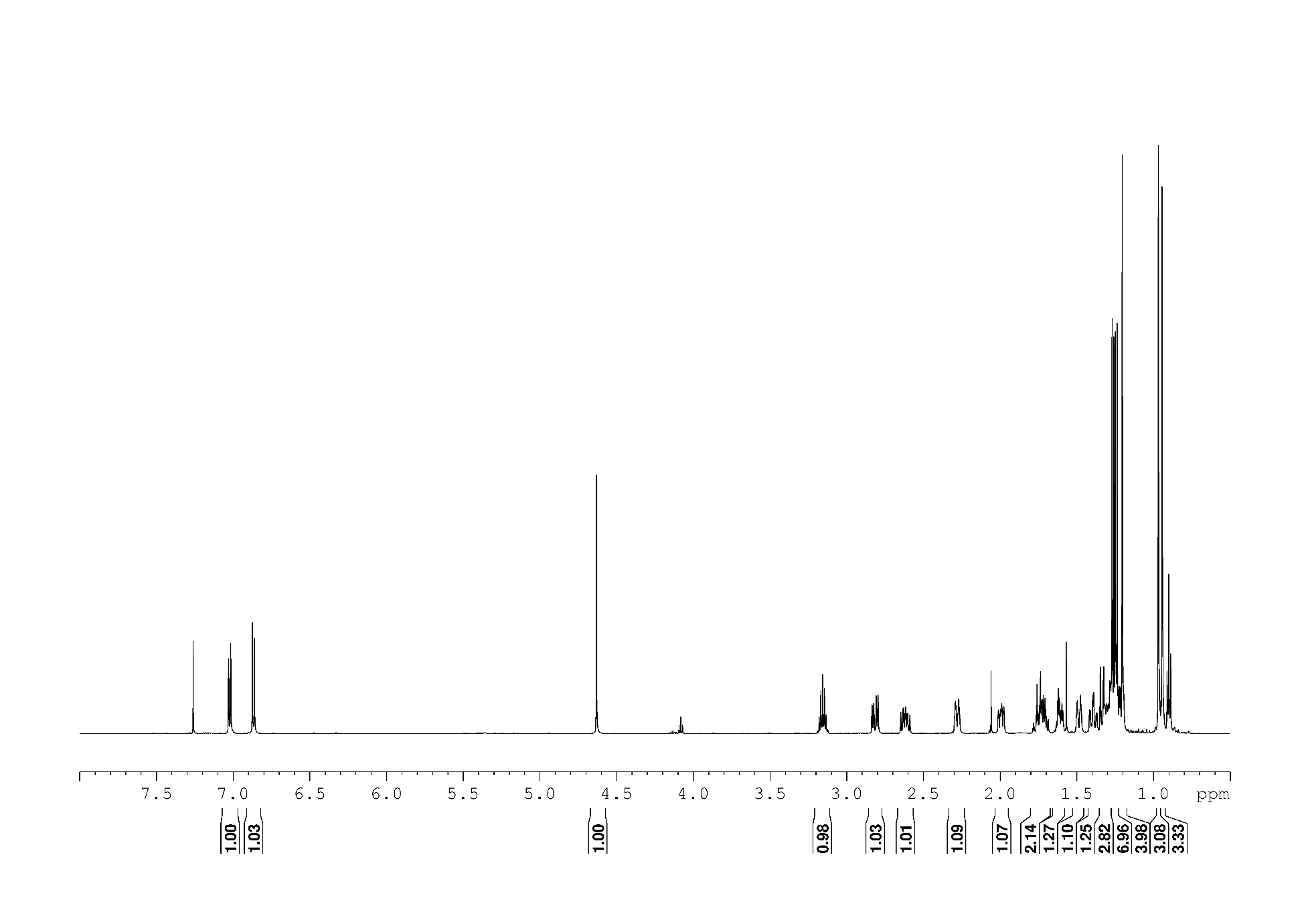


**Supplementary figure 9. NMR analysis of 14-hydroxy-dehydroabietadiene (5):** ^1^H NMR spectrum (599.85 MHz, CDCl_3_). Couplings; see Supplementary table 2.


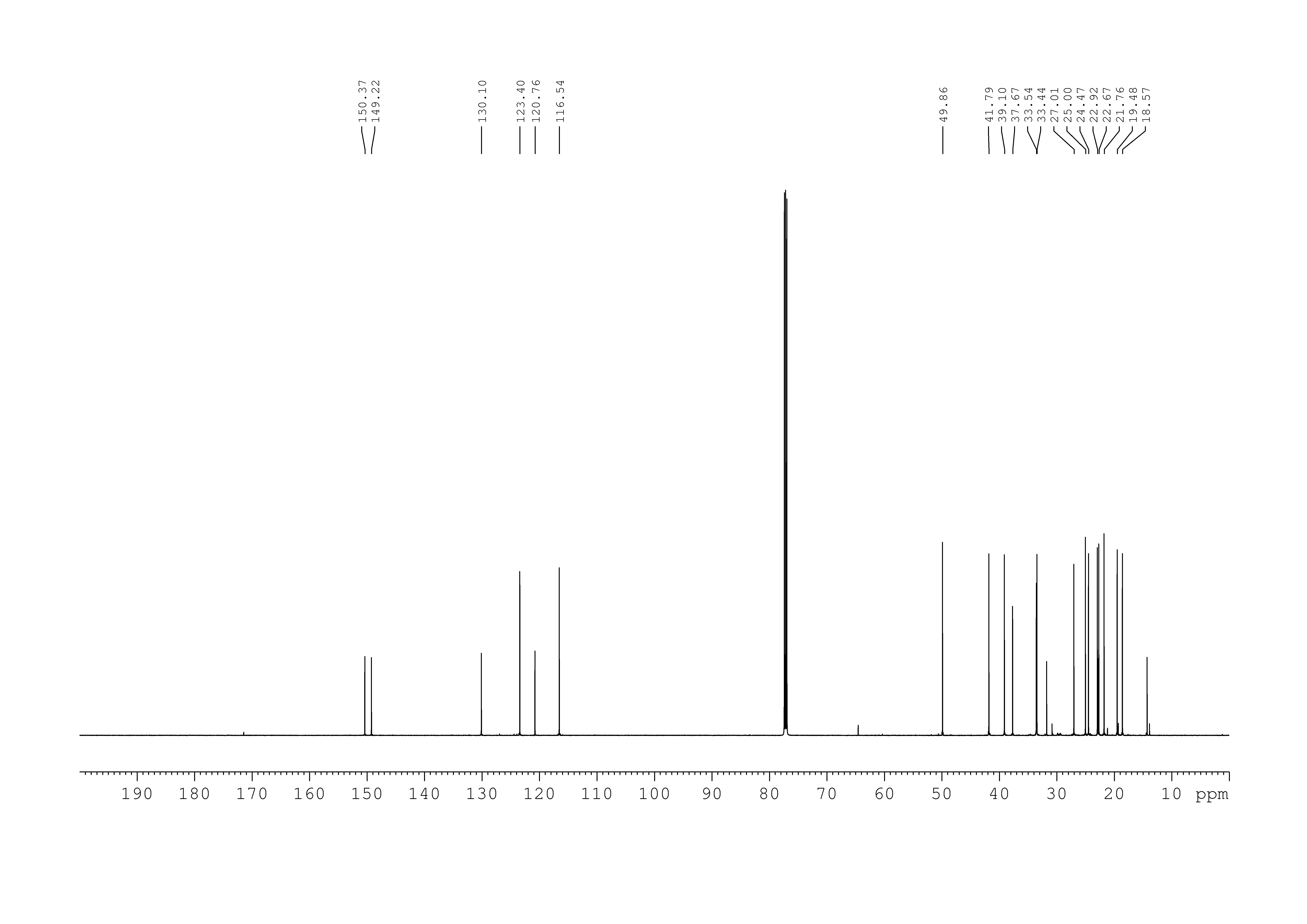


**Supplementary figure 10. NMR analysis of 14-hydroxy-dehydroabietadiene (5):** ^13^C NMR spectrum (599.85 MHz, CDCl_3_). Couplings; see Supplementary table 2. Ref: ^12^

**
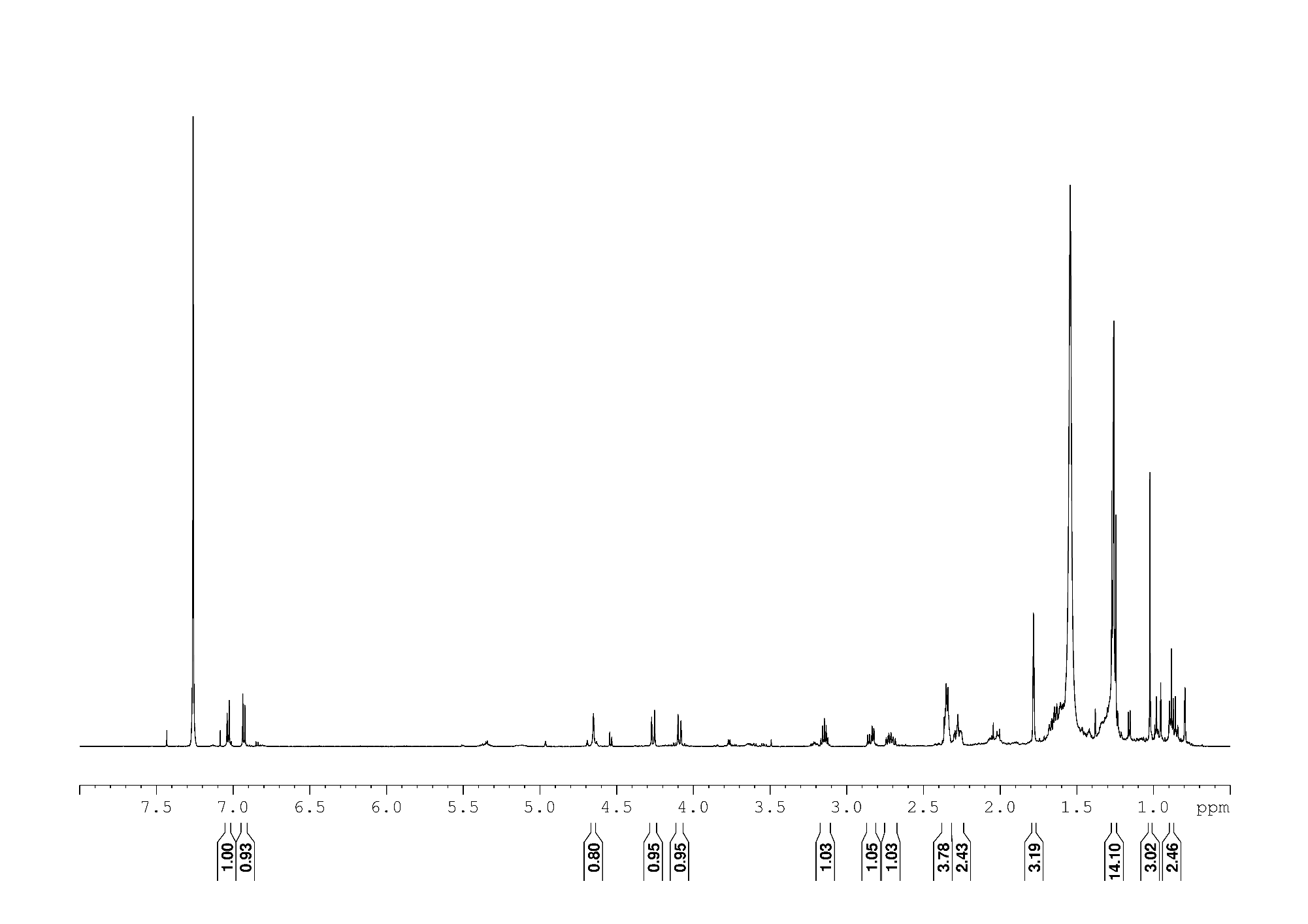
**

**Supplementary figure 11. NMR analysis of 18(4→3)abeo-abietatrien-14,18-diol (6):** ^1^H NMR spectrum (600.13 MHz, CDCl_3_). Couplings; see Supplementary table 3. Ref.: This work


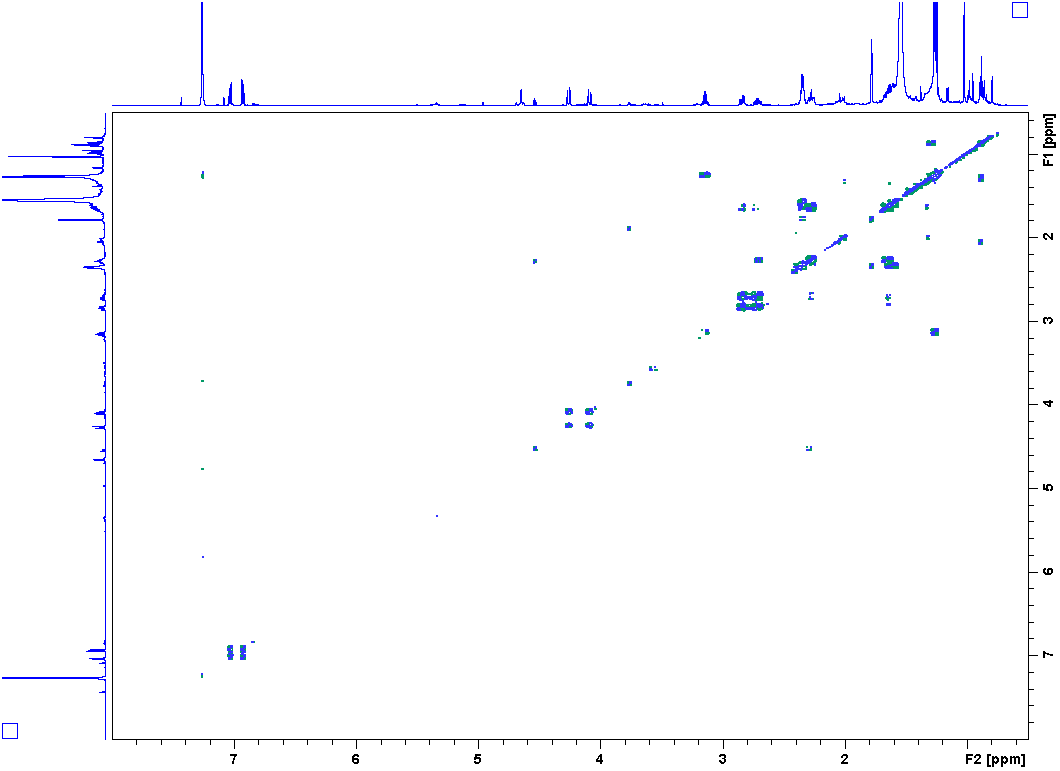


**a**


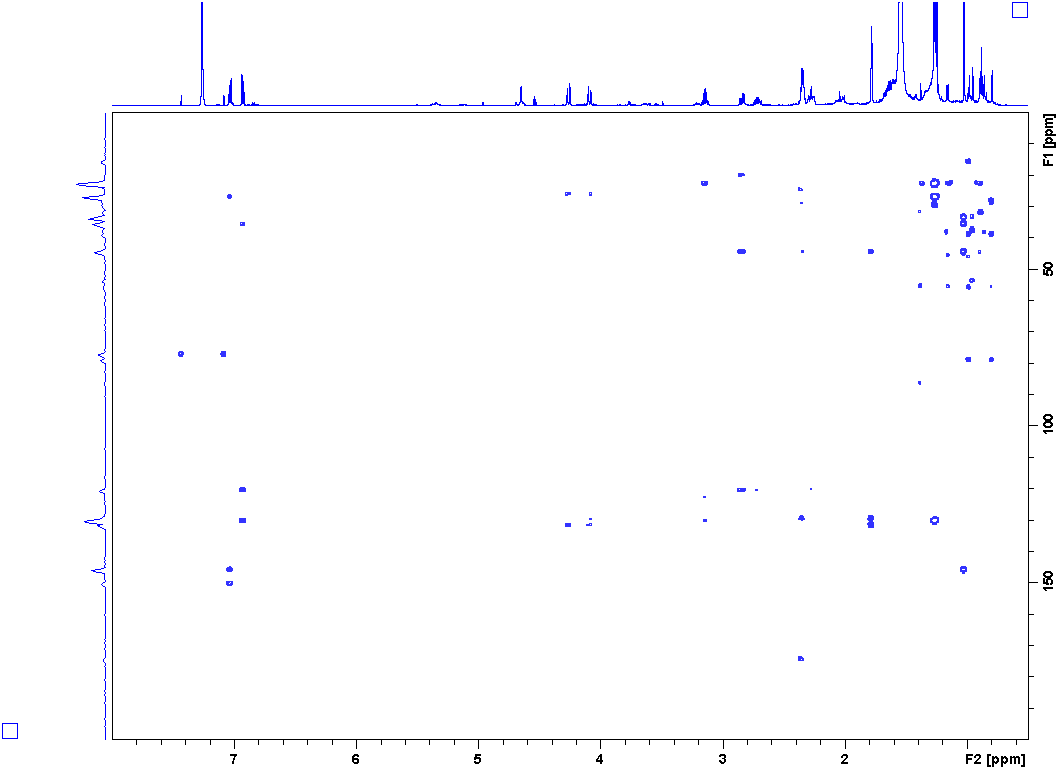


**b**

**Supplementary figure 12. NMR analysis of 18(4→3)abeo-abietatrien-14,18-diol (6):** (**a**) COSY and (**b**) HMBC, spectra of **6** Couplings; see Supplementary table 3. Ref.: This work

##
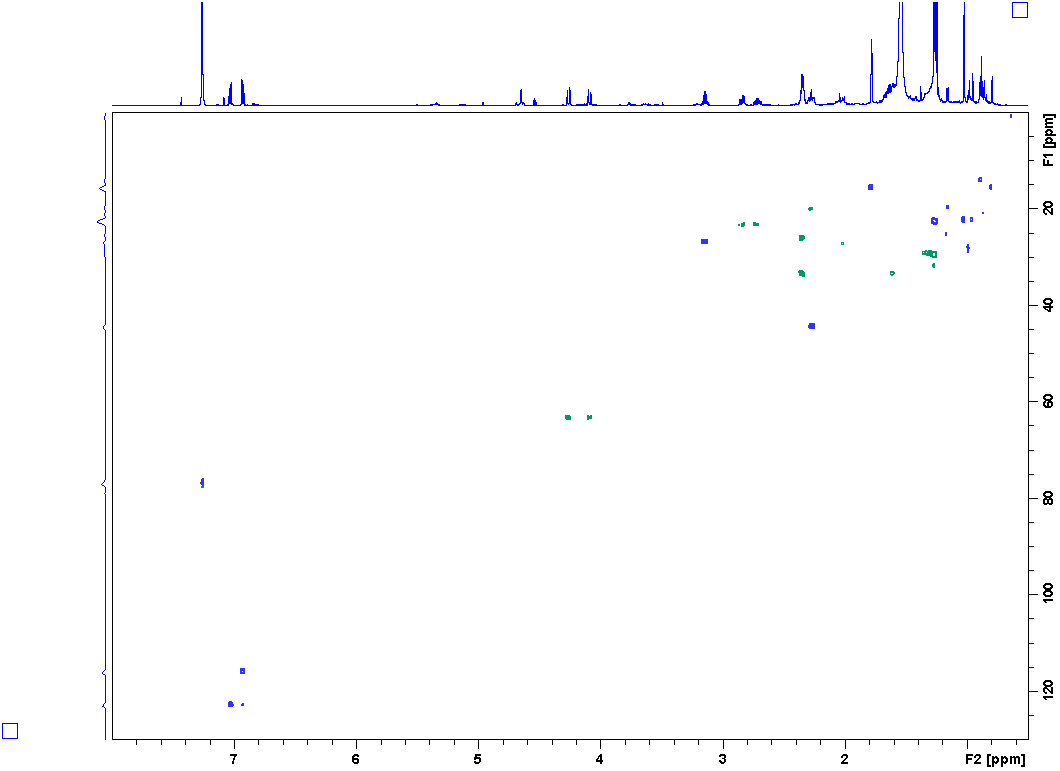


**a**


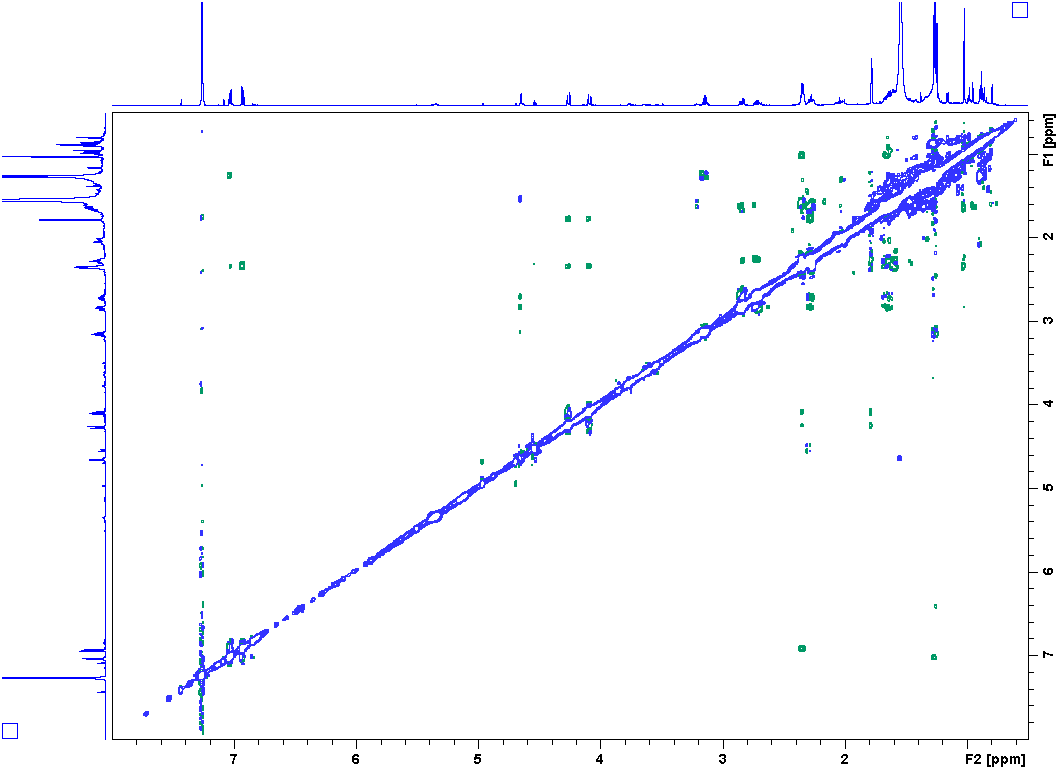


**b**

**Supplementary figure 13. NMR analysis of 18(4→3)abeo-abietatrien-14,18-diol (6):**  (**a**) HSQC and (**b**) ROESY spectra of **6**. Couplings; see Supplementary table 3. Ref.: This work

**
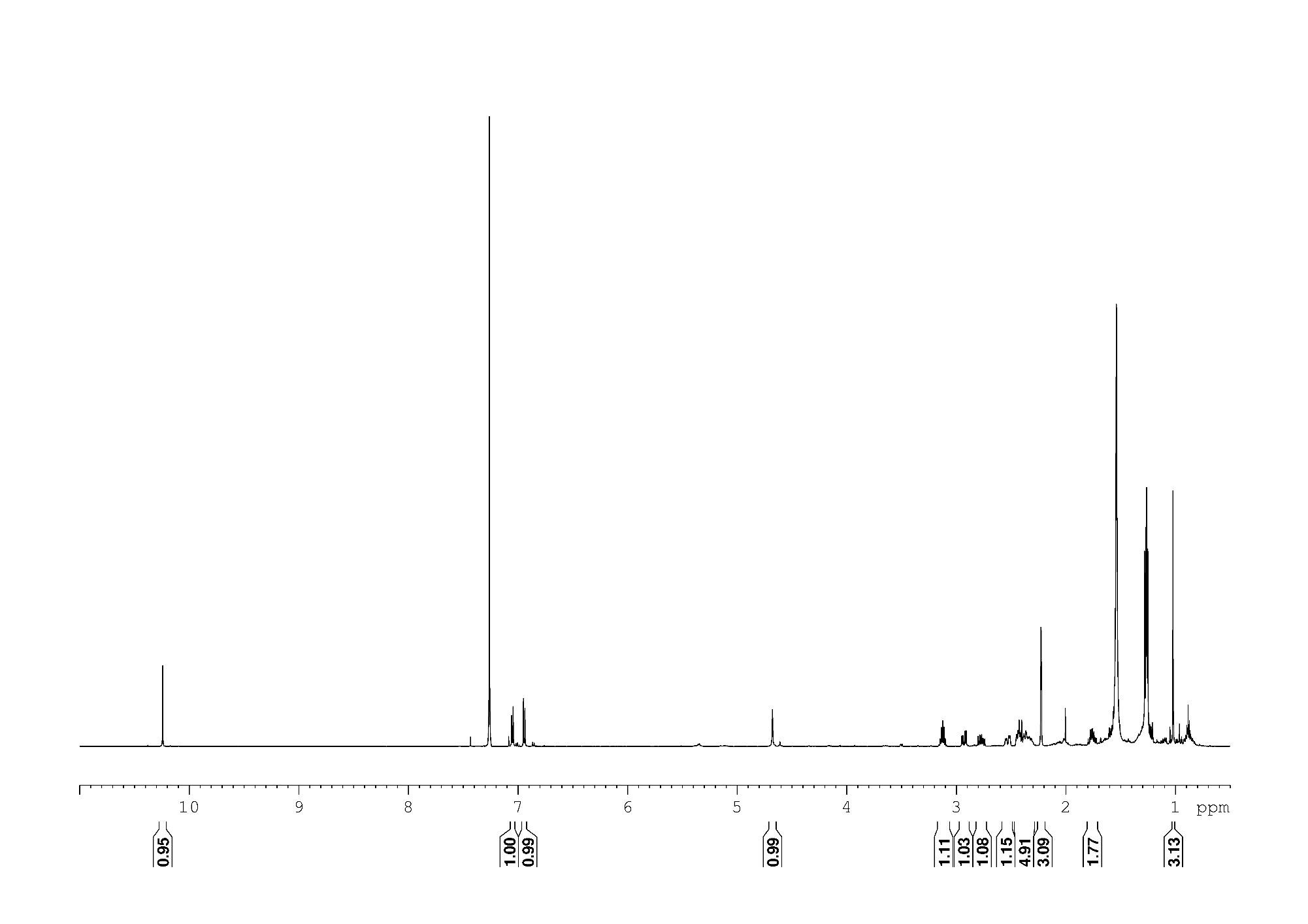
**

**c**

**Supplementary figure 14 of 14-hydroxy-18(4→3)abeo-abietatrien-18-al (7):** (**a**) ^1^H NMR spectrum (600.13 MHz, CDCl_3_). Couplings; see Supplementary table 4. Ref.: This work


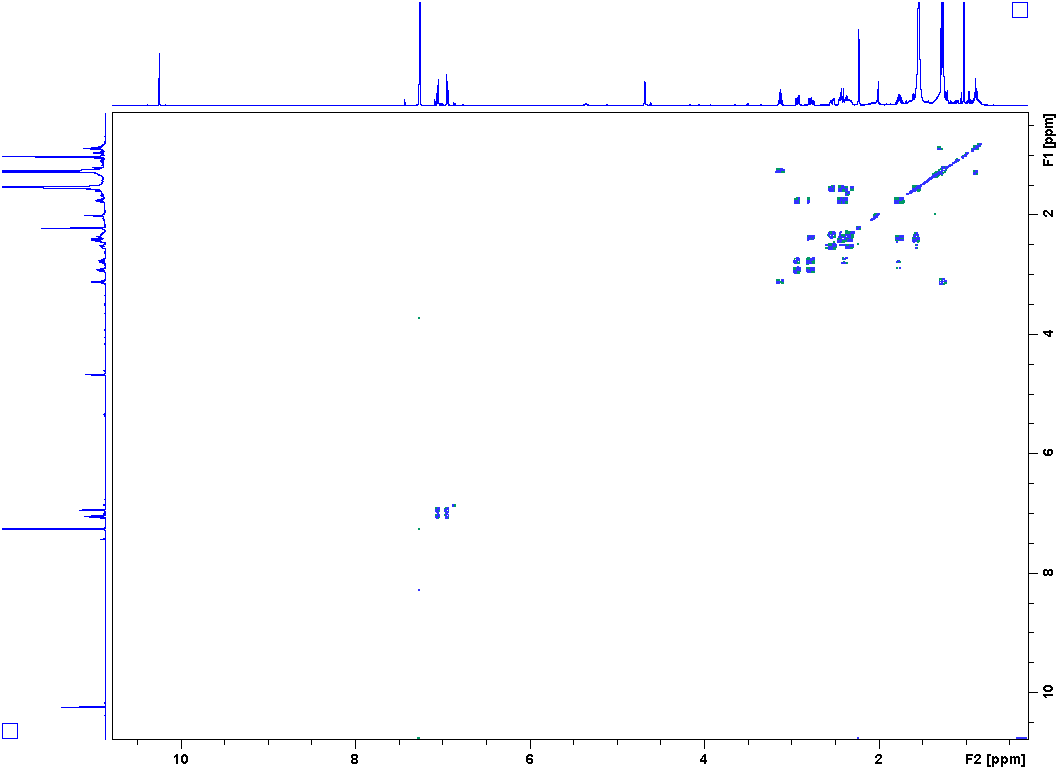


**a**


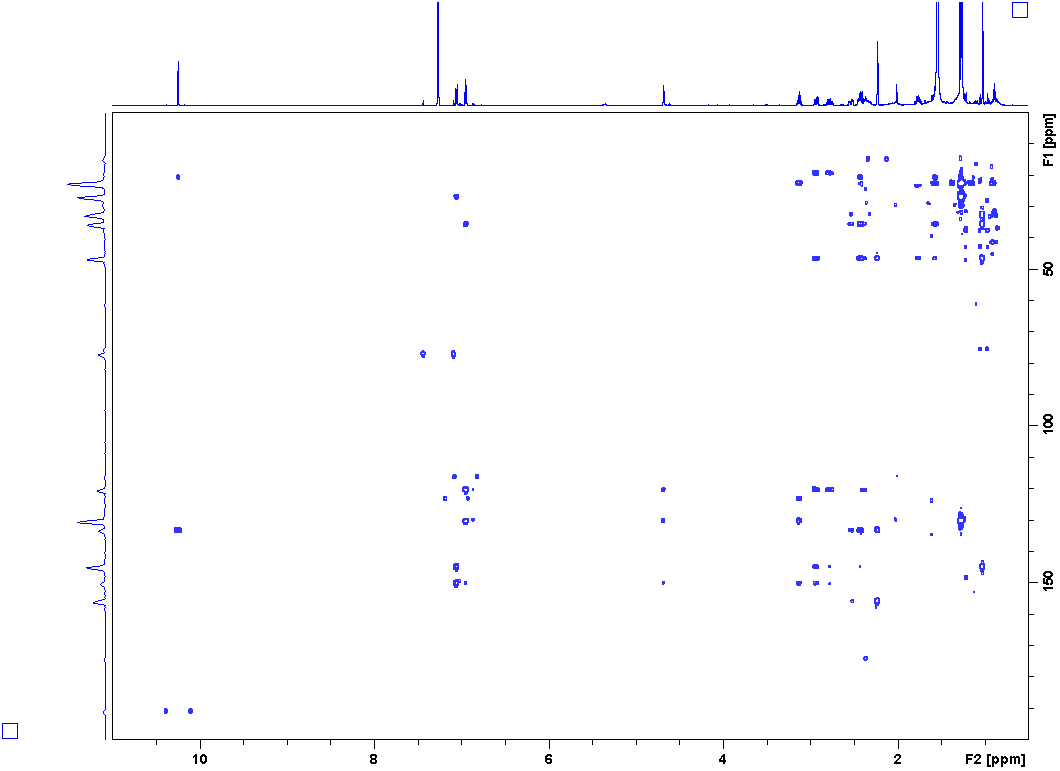


**b**

**Supplementary figure 15 of 14-hydroxy-18(4→3)abeo-abietatrien-18-al (7):** (**a**) COSY, (**b**) HMBC spectra of **7.** Couplings; see Supplementary table 4. Ref.: This work


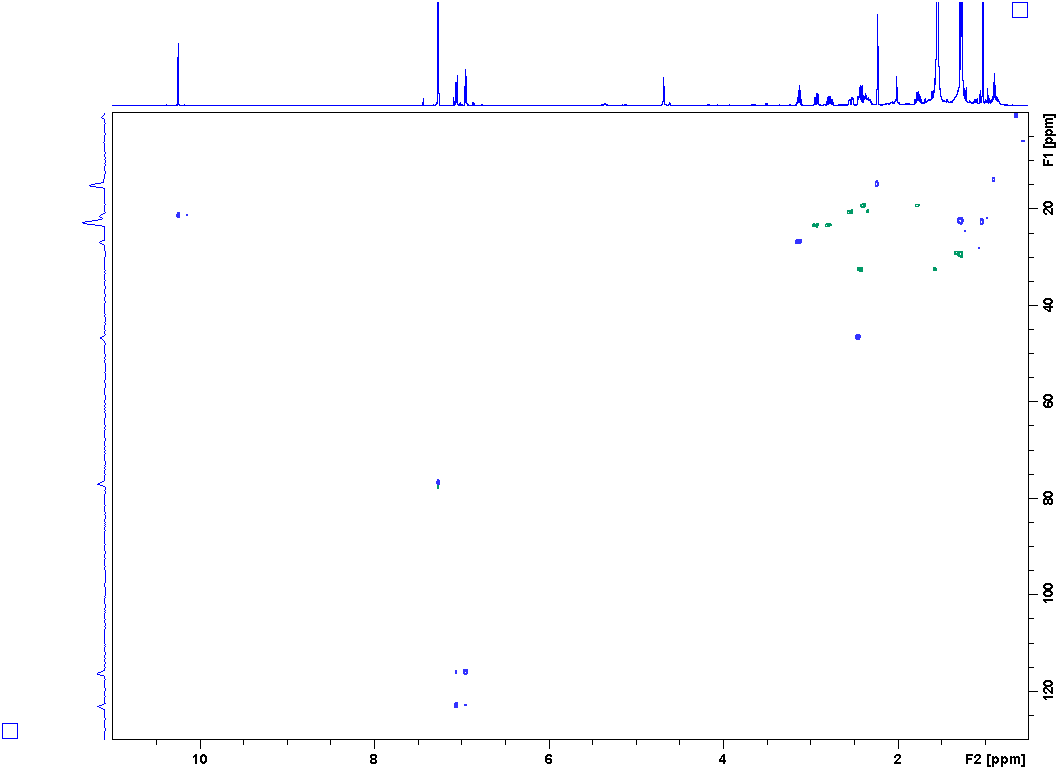


**a**


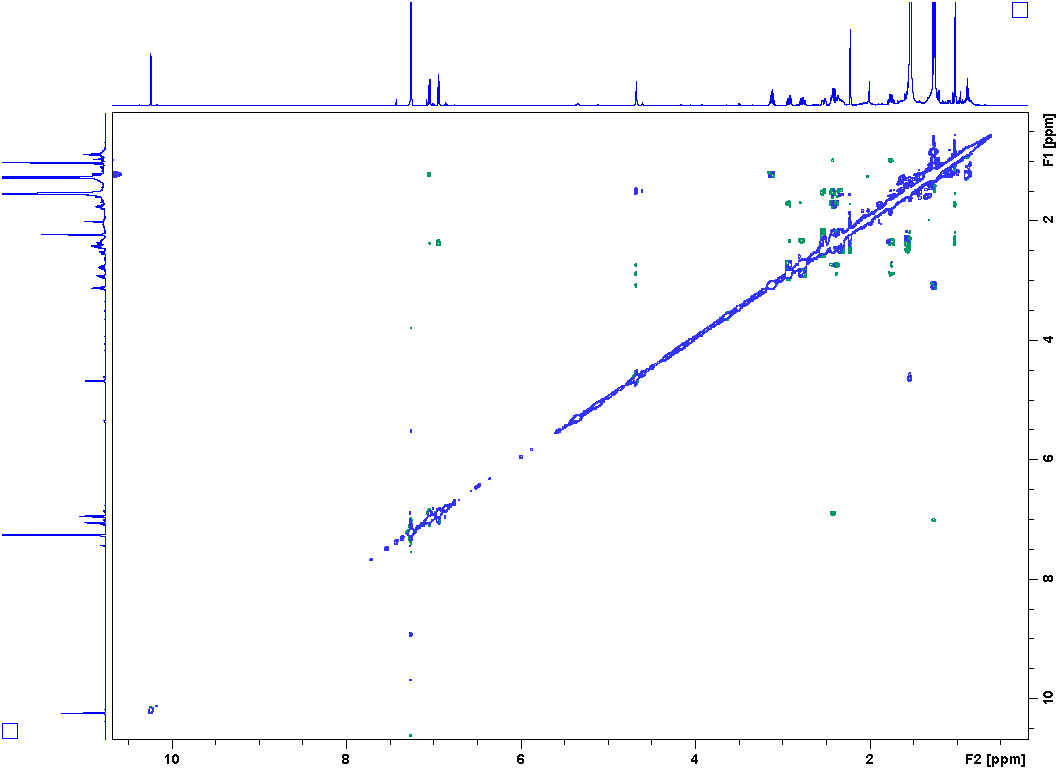


**b**

**Supplementary figure 16 of 14-hydroxy-18(4→3)abeo-abietatrien-18-al (7):** (**a**) HSQC and (**b**) ROESY spectra of **7.** Couplings; see Supplementary table 4. Ref.: This work

**
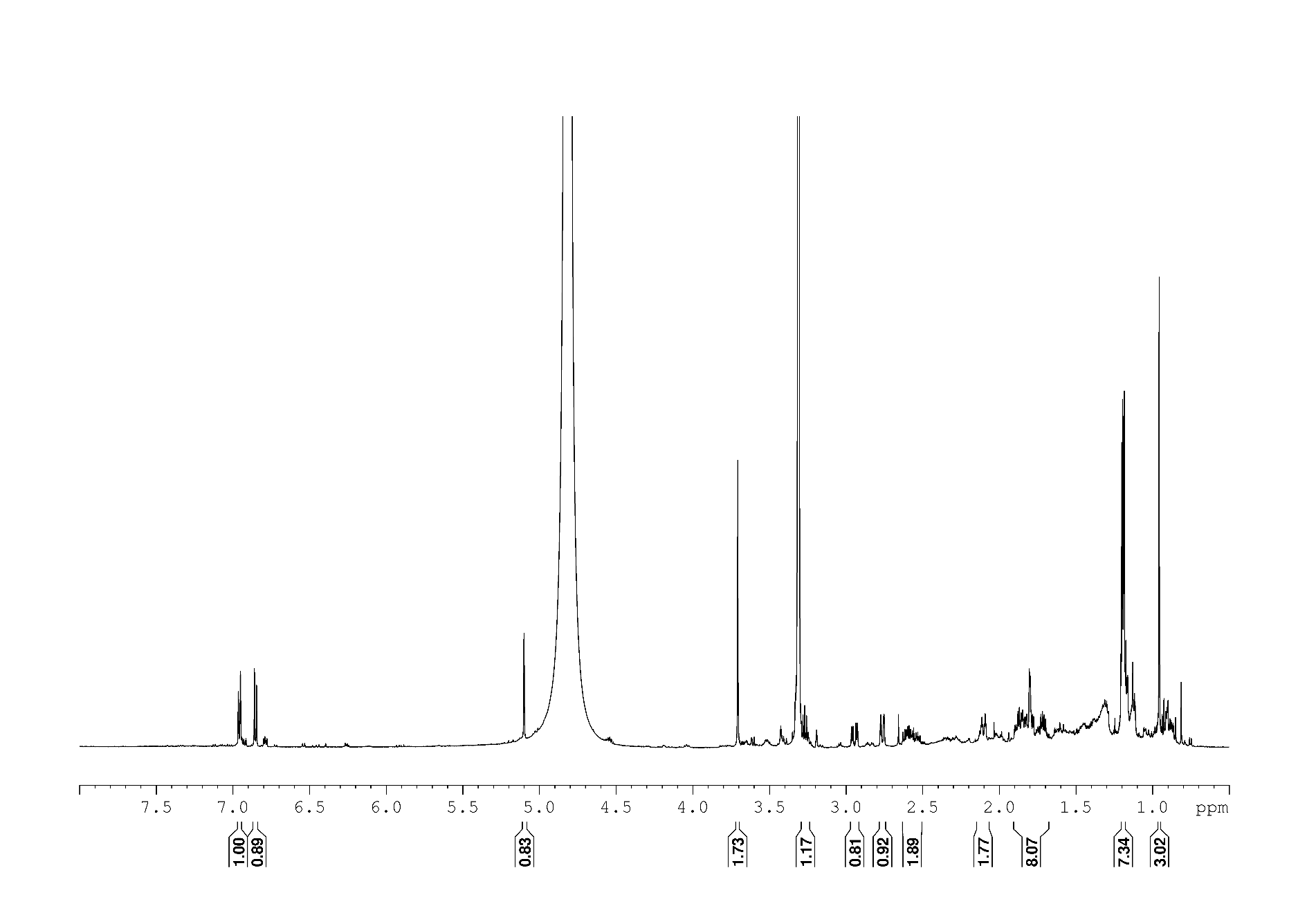
**

**Supplementary figure 17 NMR analysis of 18*R*(4→3)abeo-abietatrien-19-ene-14,18,20-triol (9):** ^1^H NMR spectrum (600.13 MHz, methanol-*d*_4_). Couplings; see Supplementary table 5. Ref.: This work


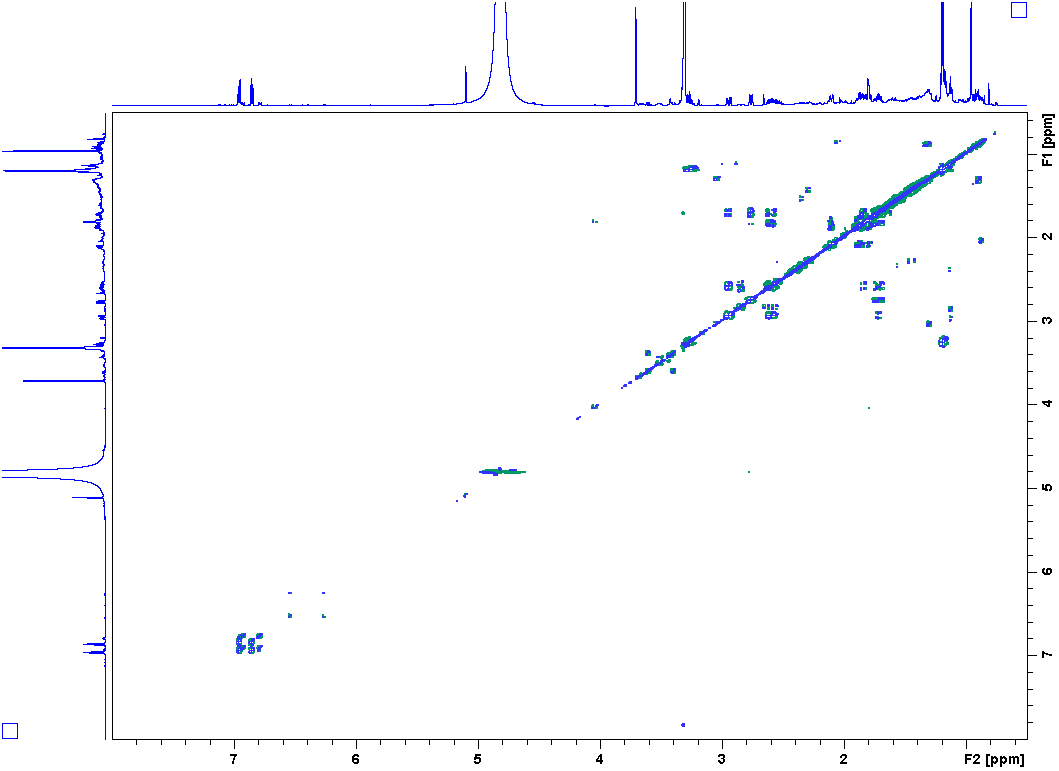

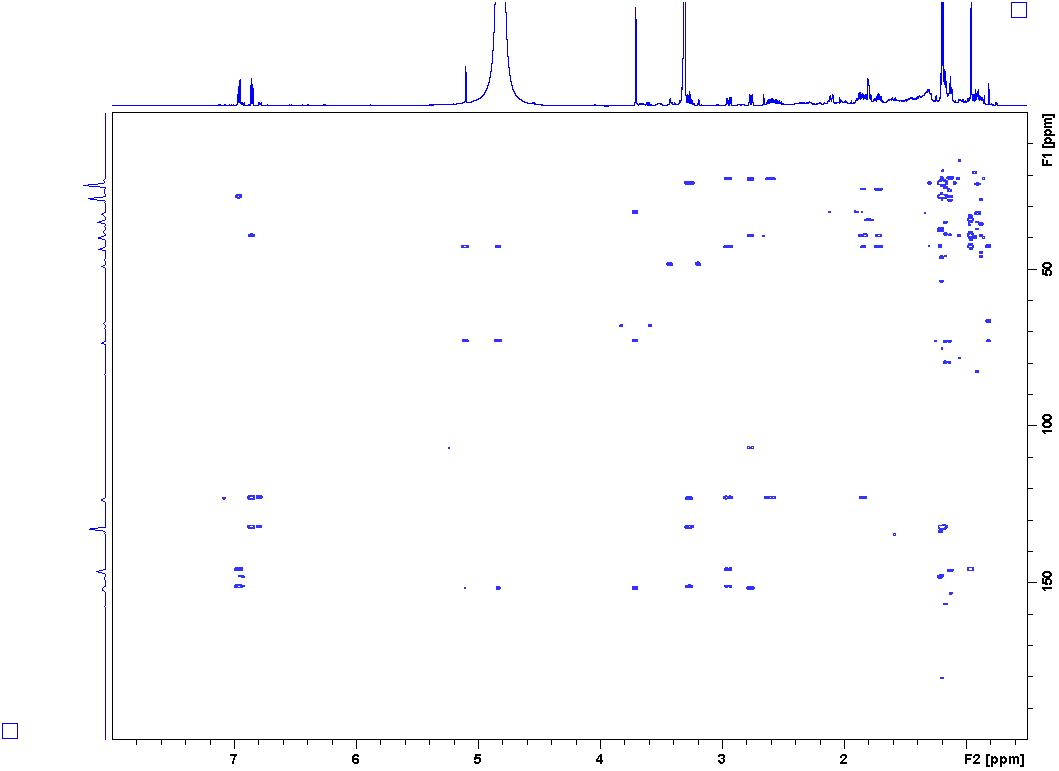


**b**

**a**

**Supplementary figure 18 NMR analysis of 18*R*(4→3)abeo-abietatrien-19-ene-14,18,20-triol (9):** (**a**) COSY, (**b**) HMBC spectra of **9.** Couplings; see Supplementary table 5. Ref.: This work


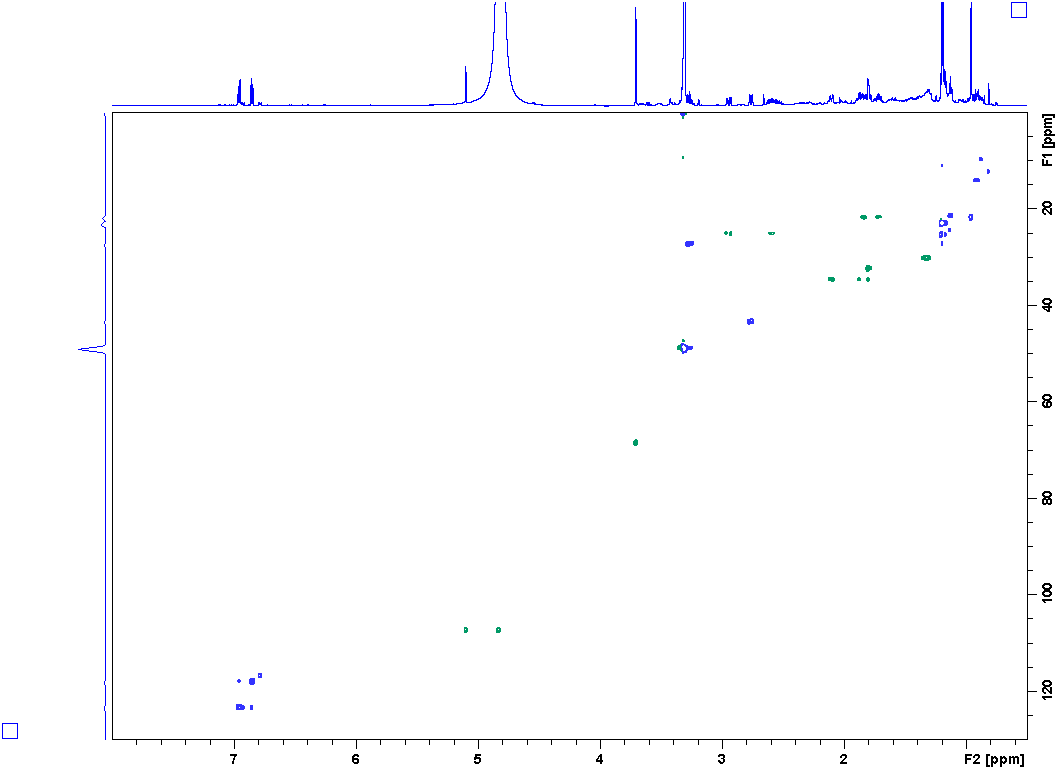

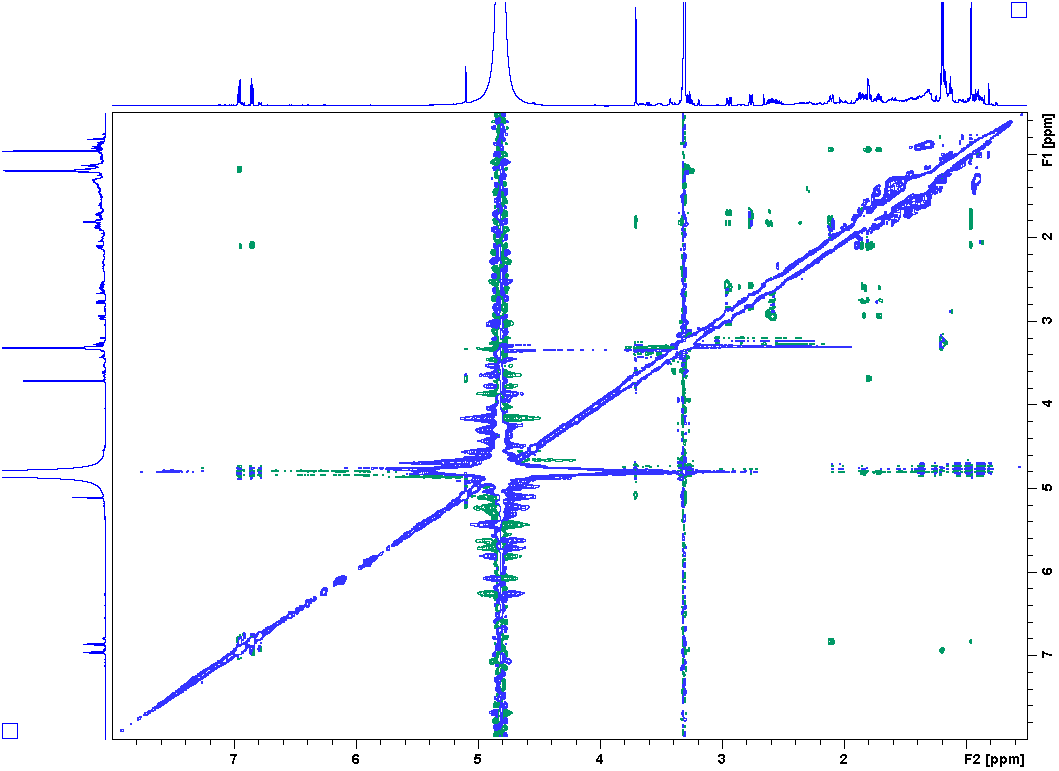


**b**

**a**

**Supplementary figure 19 NMR analysis of 18*R*(4→3)abeo-abietatrien-19-ene-14,18,20-triol (9):** (a) HSQC and (**b**) ROESY spectra of **9.** Couplings; see Supplementary table 5. Ref.: This work

**
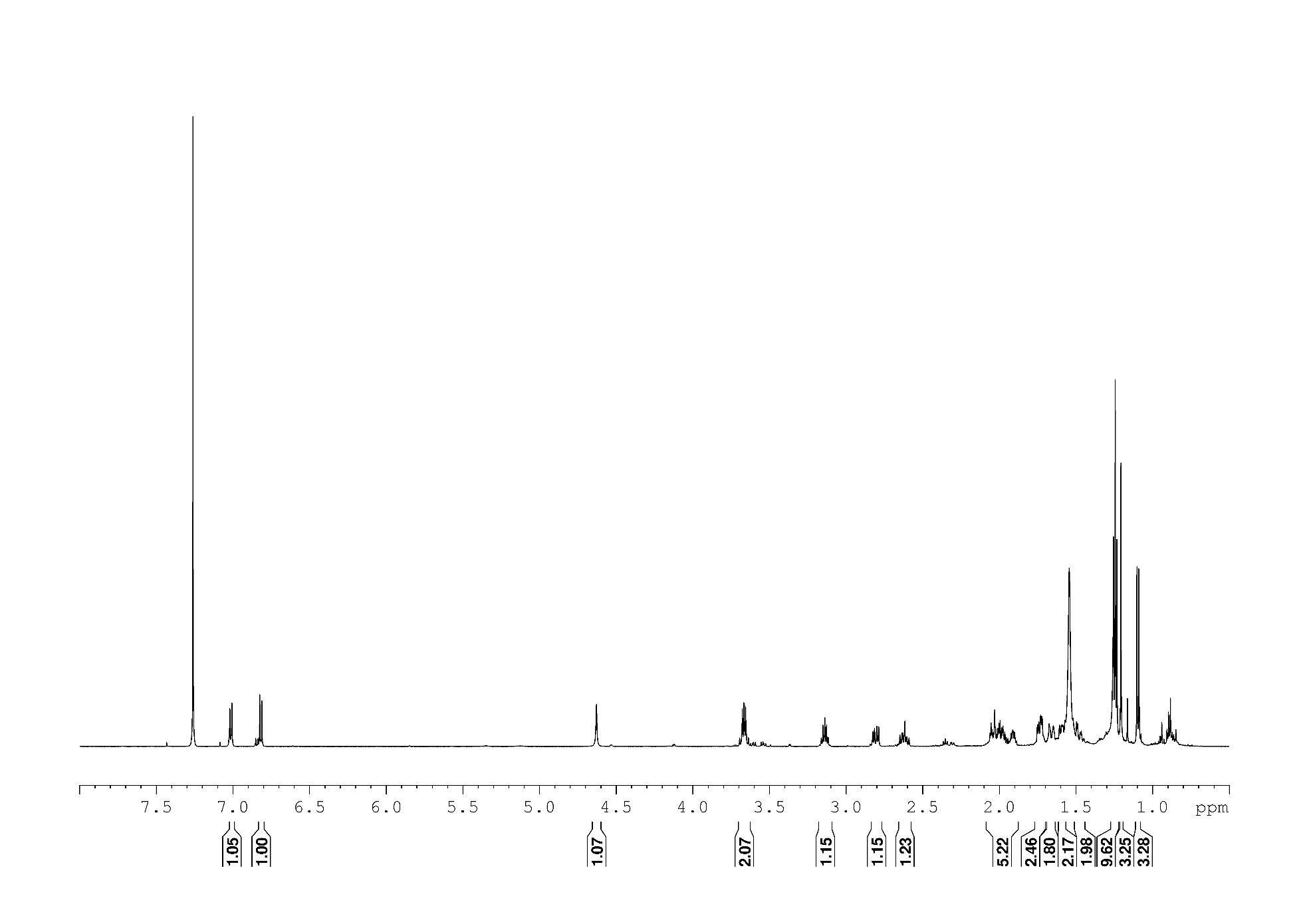
**

**Supplementary figure 20 NMR analysis of 18*R*(4→3)abeo-abietatrien-14,18-diol (10):** (**a**)^1^H NMR spectrum 600.13 MHz, CDCl_3_. Couplings; see Supplementary table 6. Ref.: This work


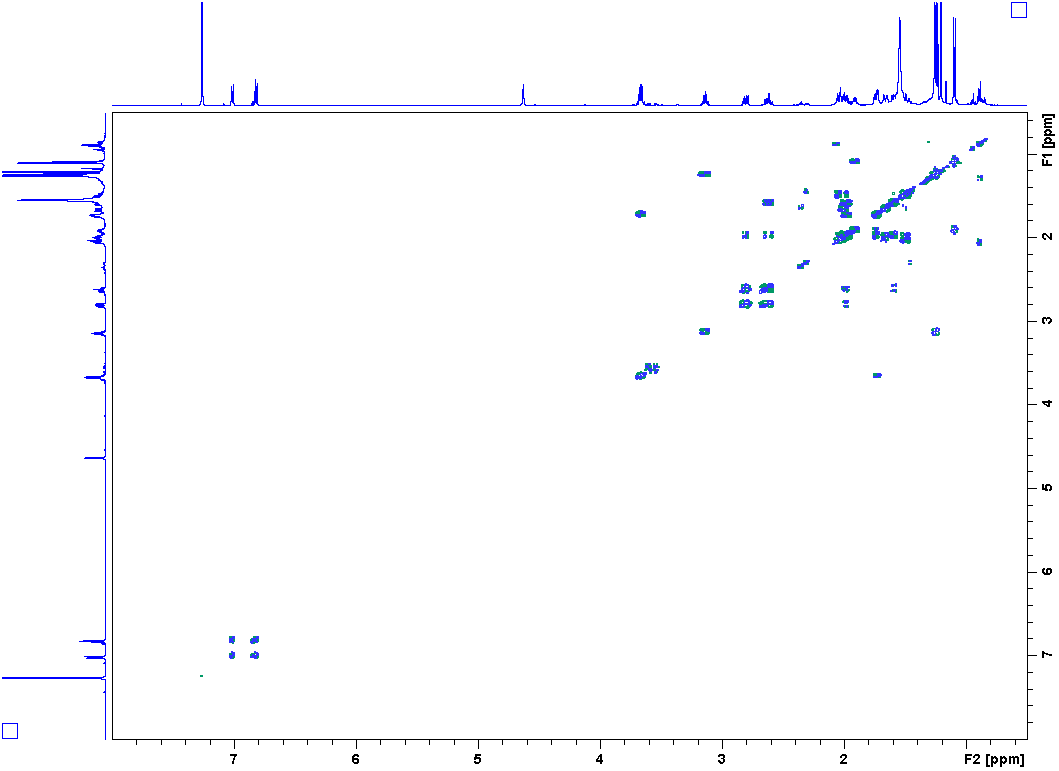


**a**


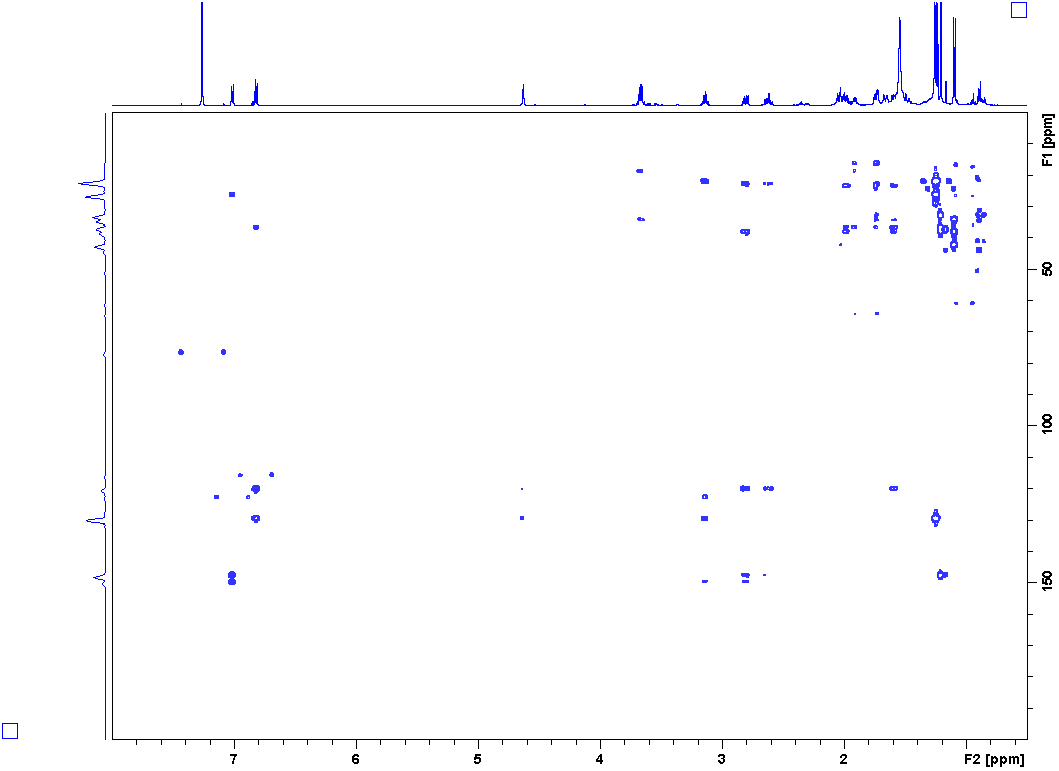


**b**

**Supplementary figure 21 NMR analysis of 18*R*(4→3)abeo-abietatrien-14,18-diol (10):** (**a**) COSY (**b**) HMBC spectra of **10**. Couplings; see Supplementary table 6. Ref.: This work


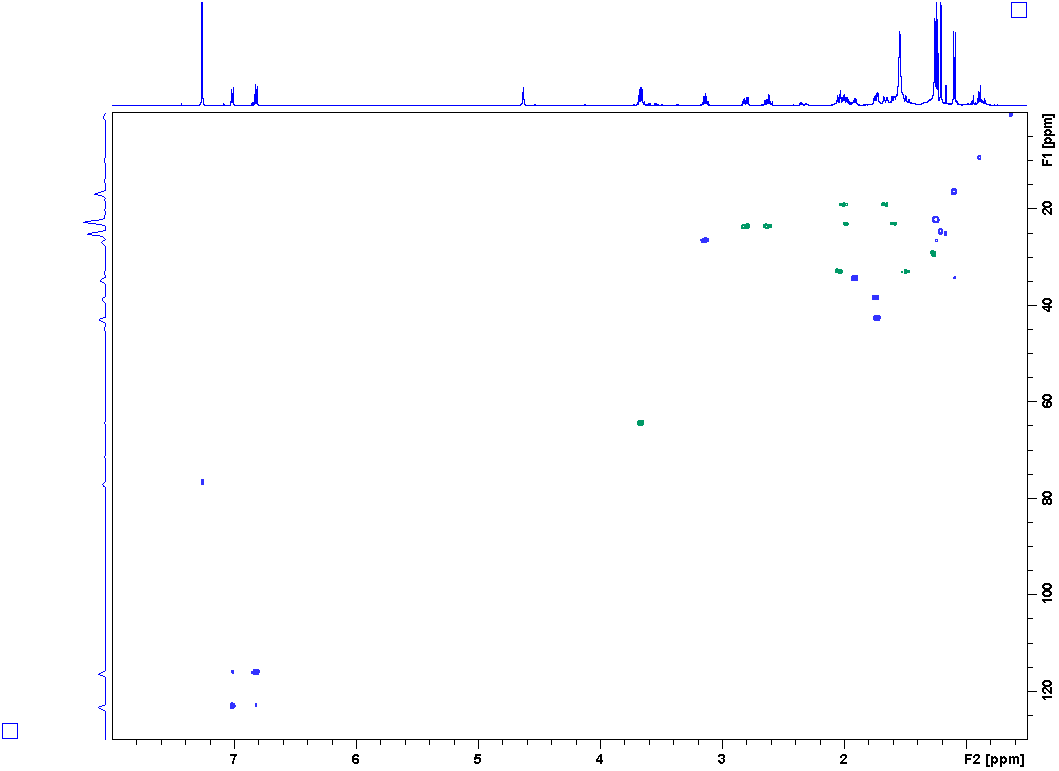


**a**


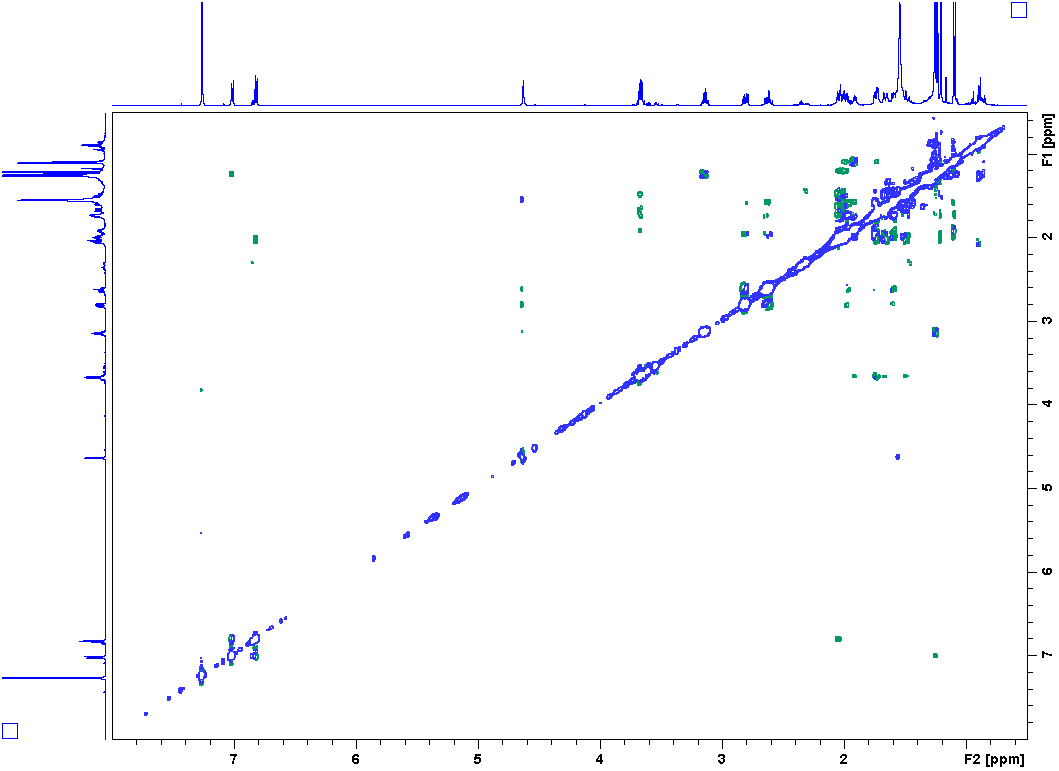


**b**

**Supplementary figure 22 NMR analysis of 18*R*(4→3)abeo-abietatrien-14,18-diol (10):** (**a**) HSQC and (**b**) ROESY spectra of **10**. Couplings; see Supplementary table 6. Ref.: This work

**
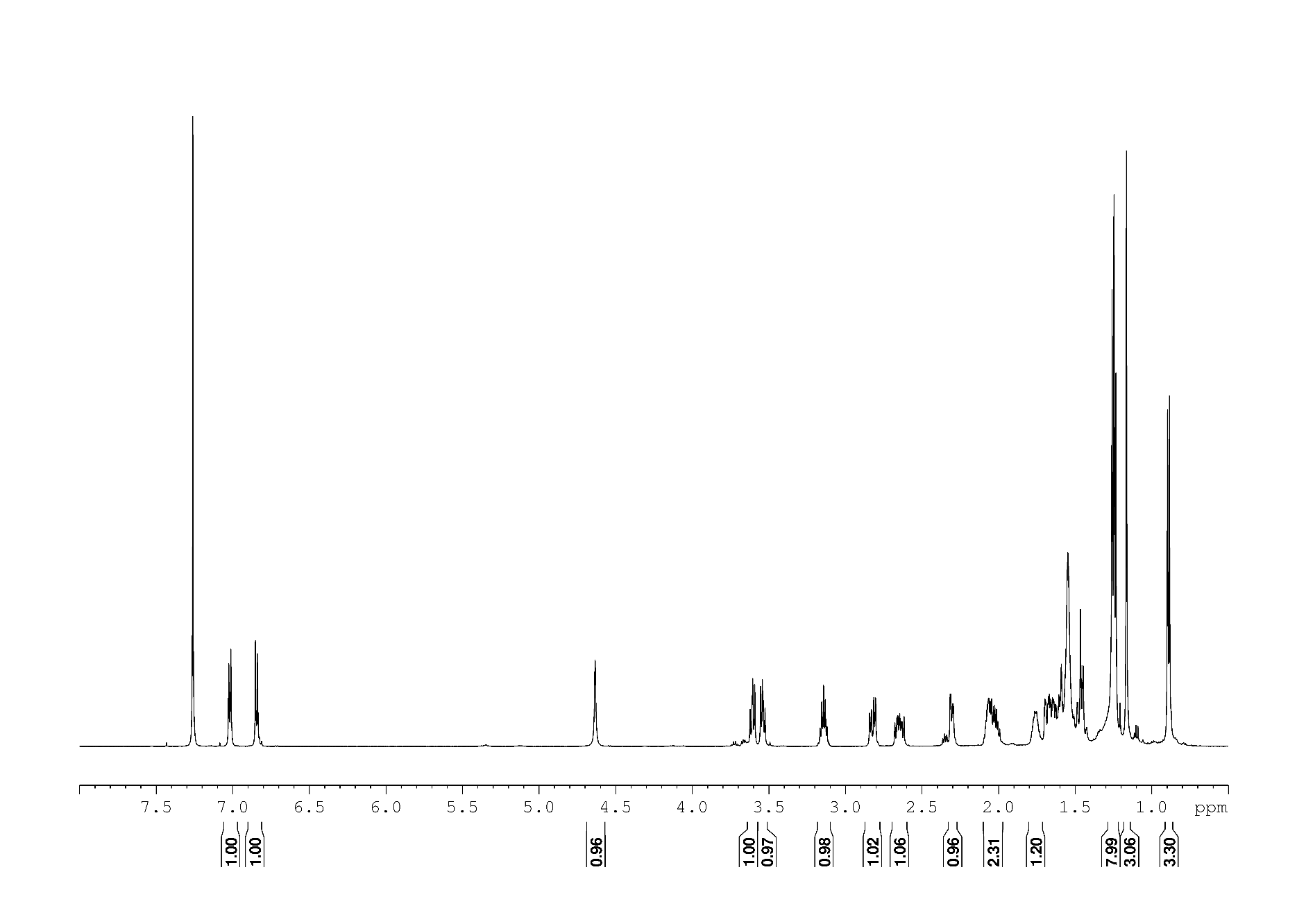
**

**Supplementary figure 23 NMR analysis of** 18*S*(4→3)abeo-abietatrien-14,18-diol (**11**): (**a**) ^1^H NMR spectrum (600.13 MHz, CDCl_3_). Couplings; see Supplementary table 7, Ref.: This work


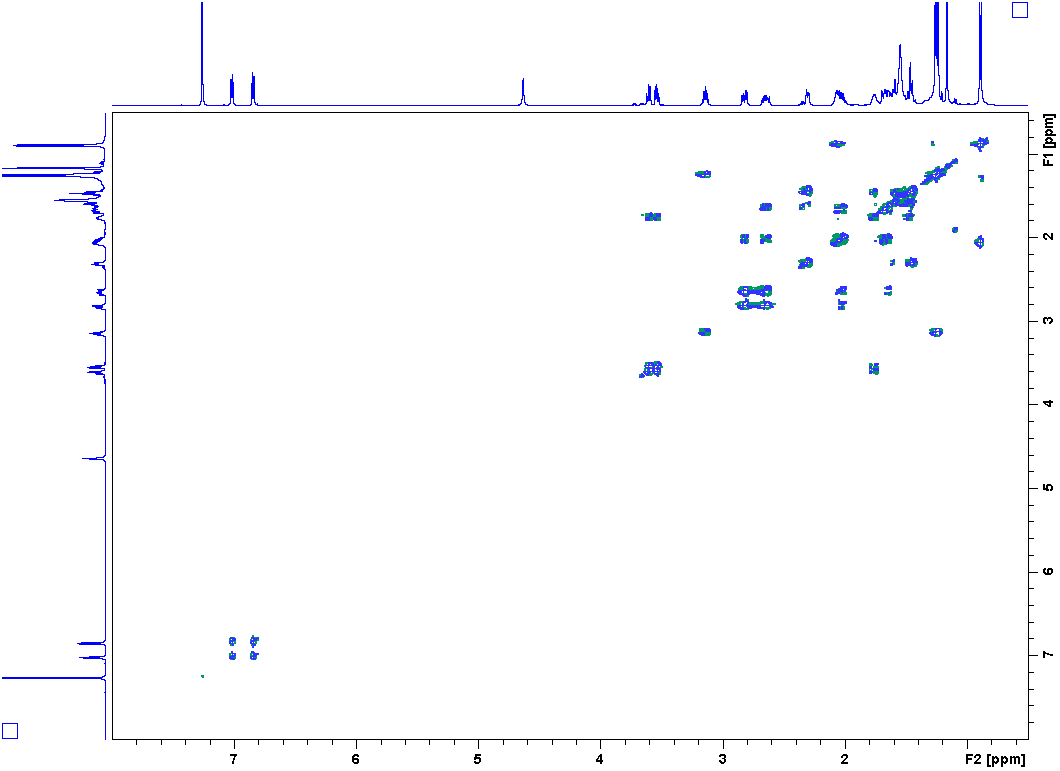

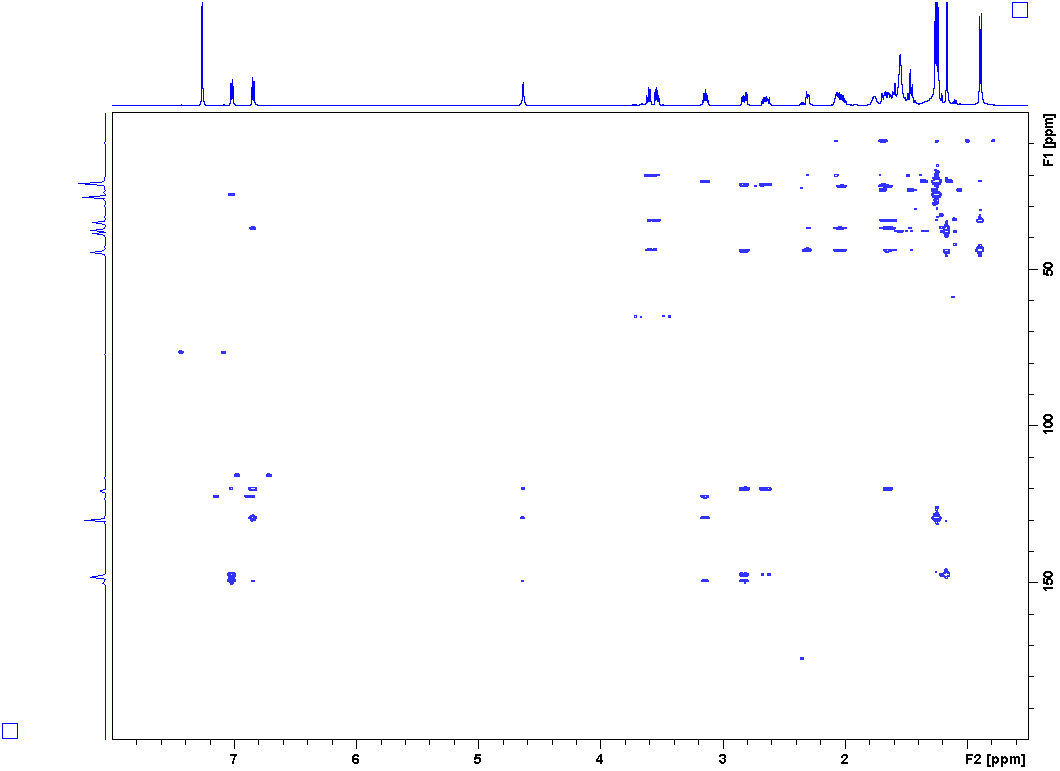


**b**

**a**

**Supplementary figure 24 NMR analysis of** 18*S*(4→3)abeo-abietatrien-14,18-diol (**11**): (**a**) COSY, and (**b**) HMBC spectra of **11.** Couplings; see Supplementary table 7. Ref.: This work


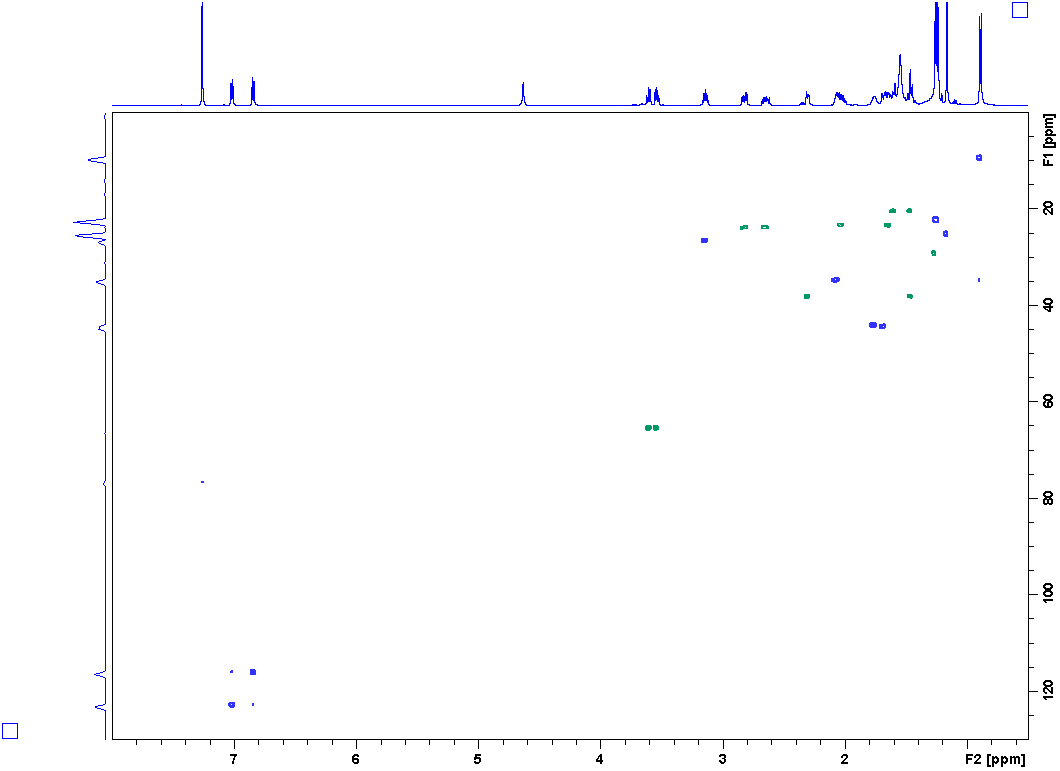


**a**

**
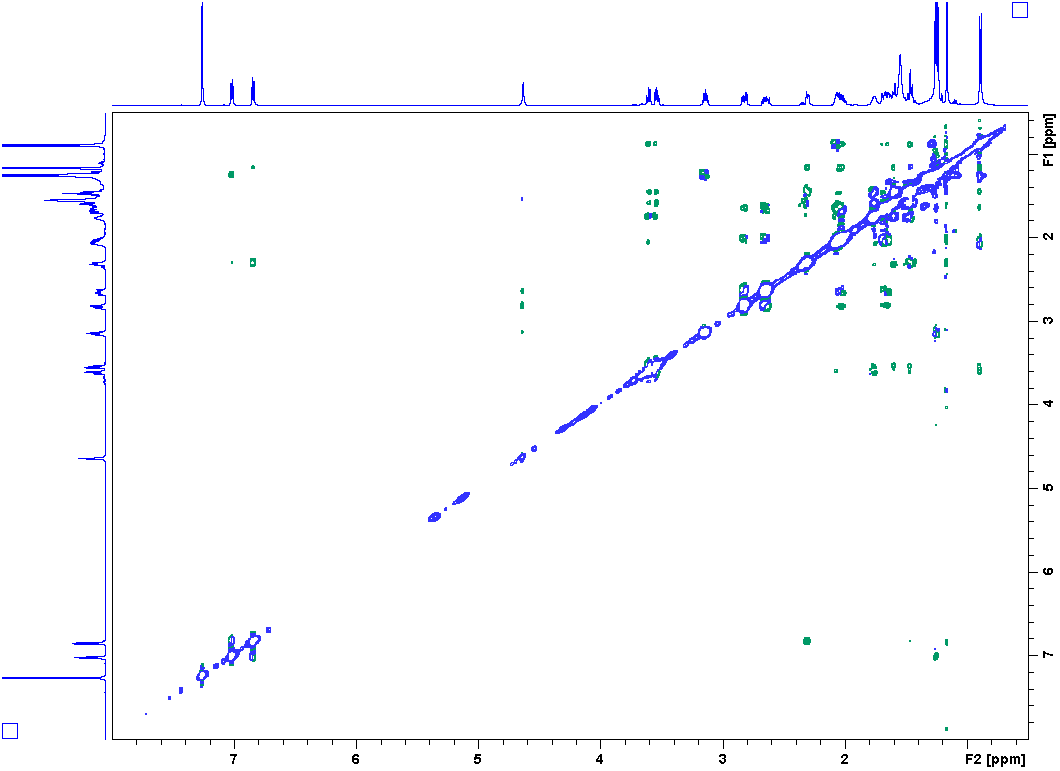
**

**b**

**Supplementary figure 25 NMR analysis of 18*S*(4→3)abeo-abietatrien-14,18-diol** (**11**): (**a**) HSQC and (**b**) ROESY spectra of **11.** Couplings; see Supplementary table 7. Ref.: This work

**
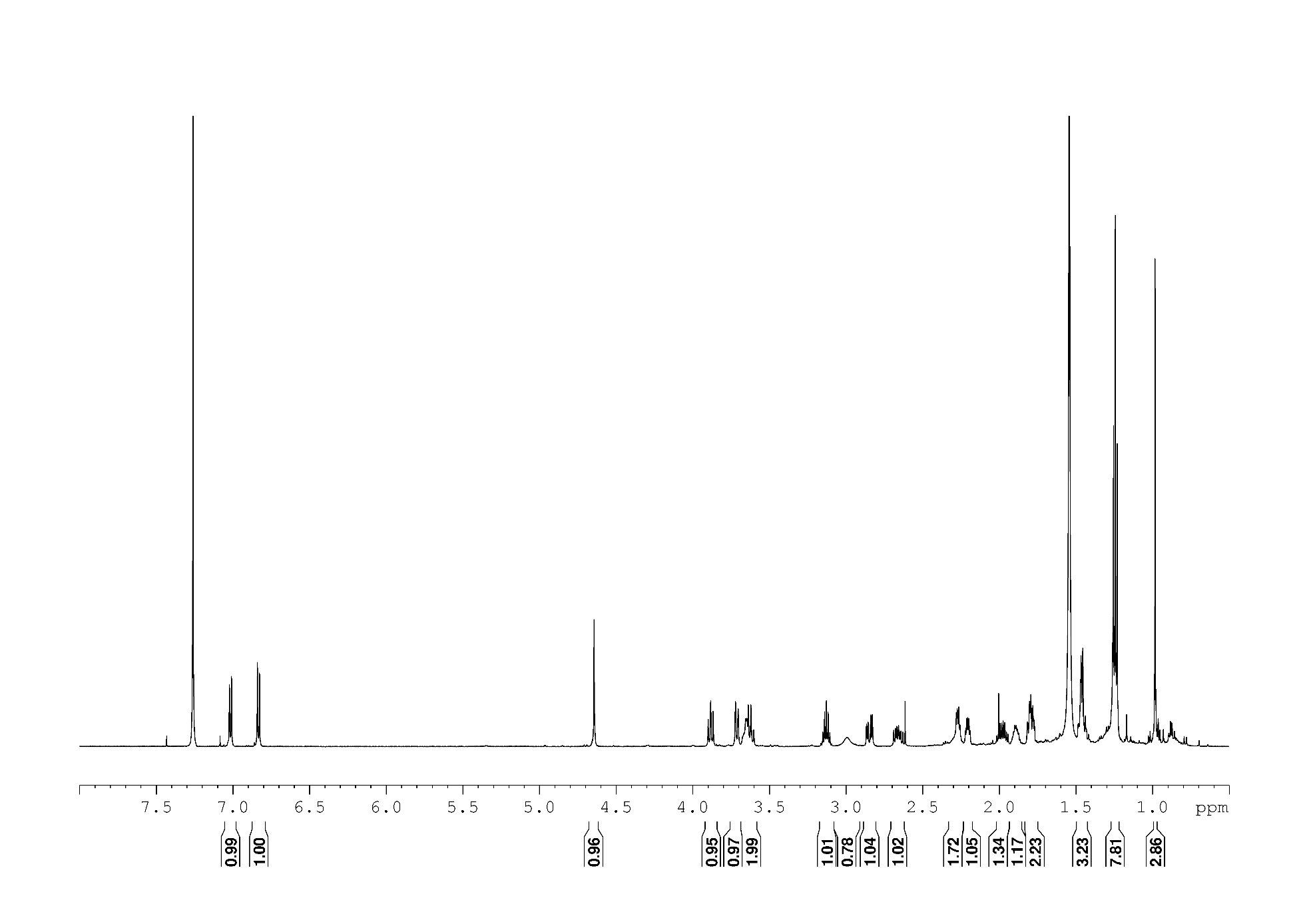
**

**Supplementary figure 26 NMR analysis of 18*S*(4→3)abeo-abietatrien-14,18,20-triol (12**): ^1^H NMR spectrum (600.13 MHz, CDCl_3_). Couplings; see Supplementary table 8. Ref.: This work


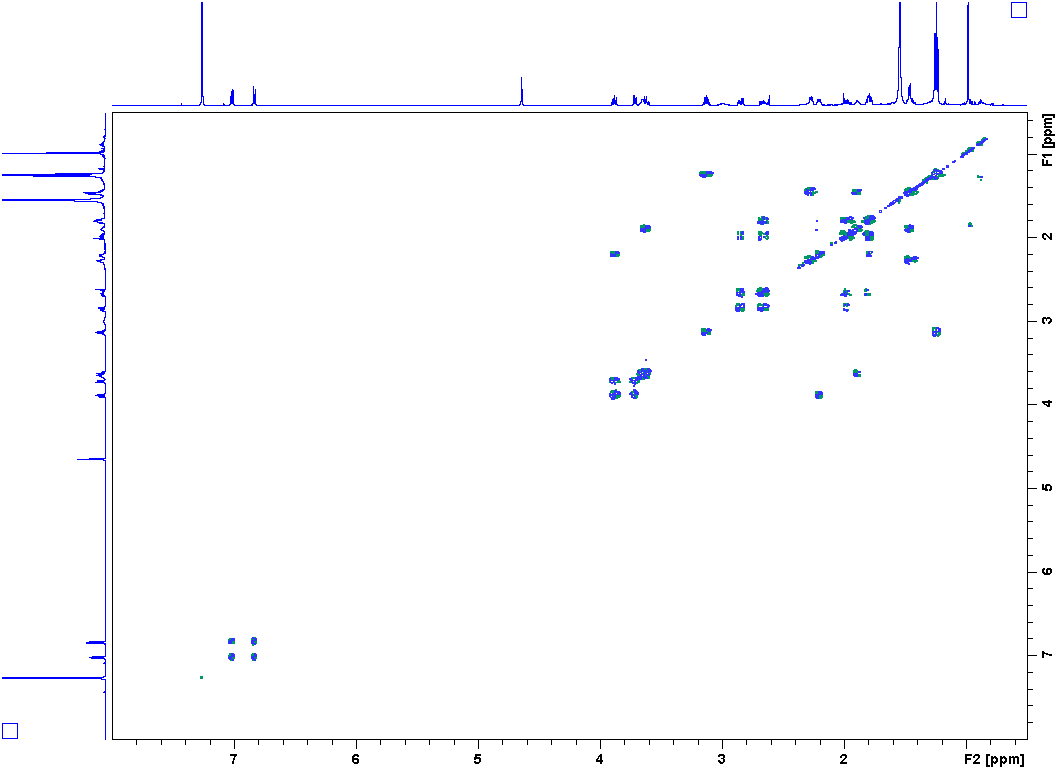


**a**


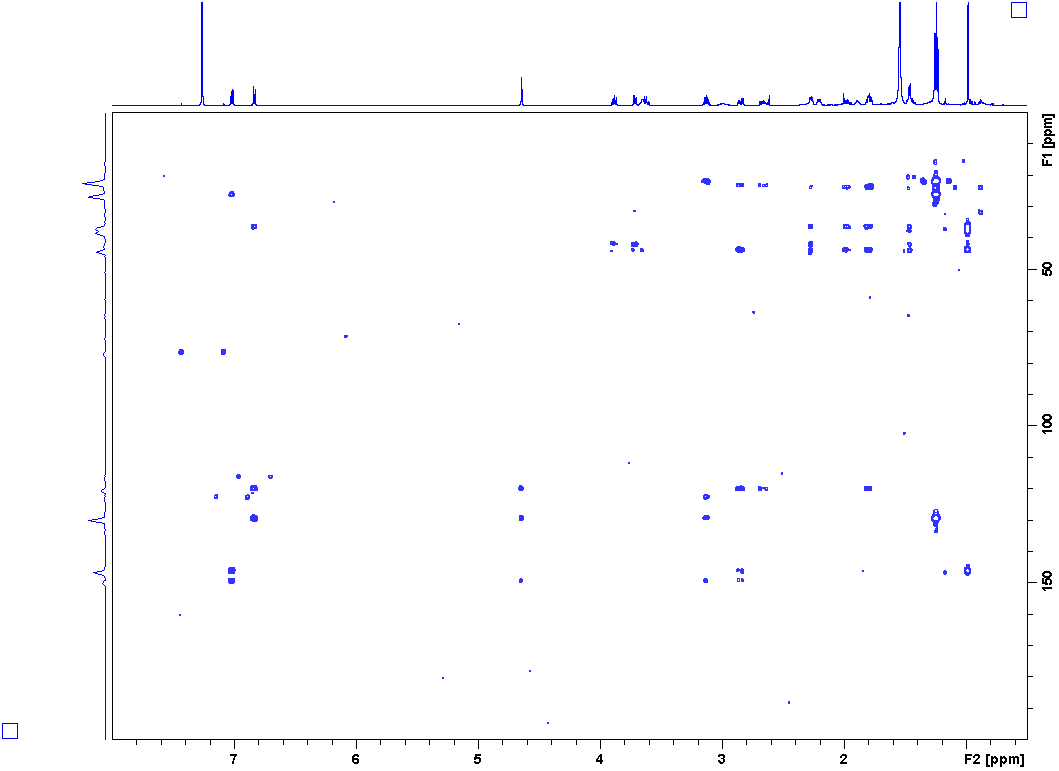


**b**

**Supplementary figure 27 NMR analysis of 18*S*(4→3)abeo-abietatrien-14,18,20-triol (12**): (**a**) COSY, and (**b**) HMBC spectra of **12.** Couplings; see Supplementary table 8. Ref.: This work


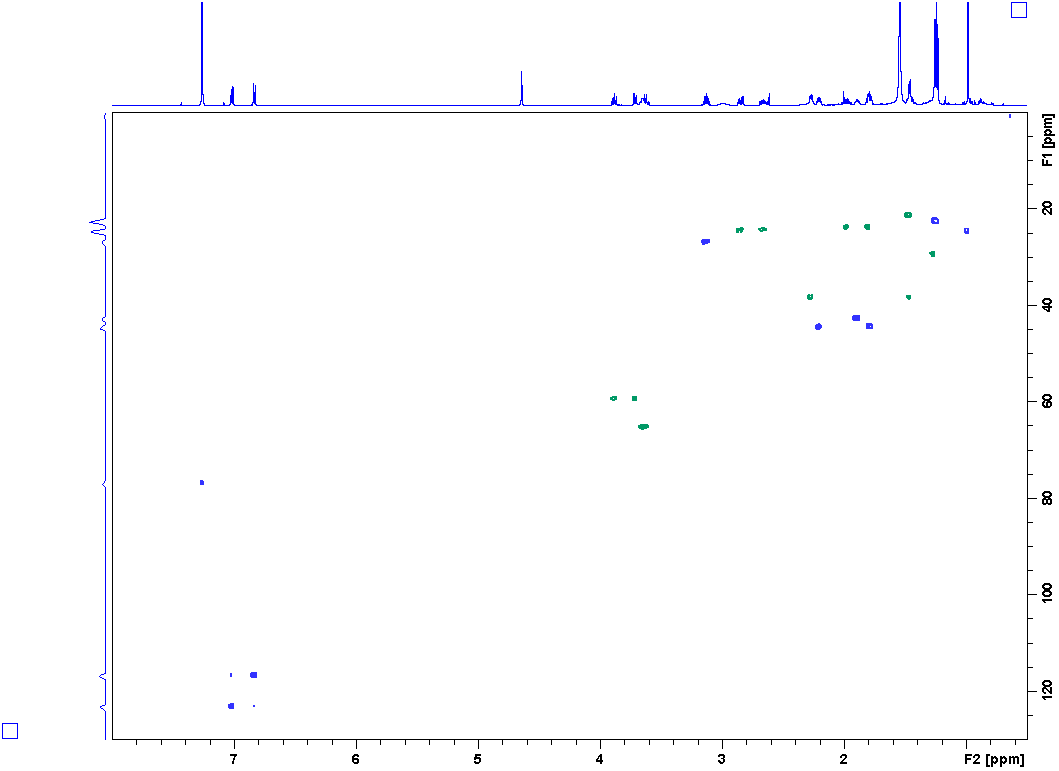


**a**


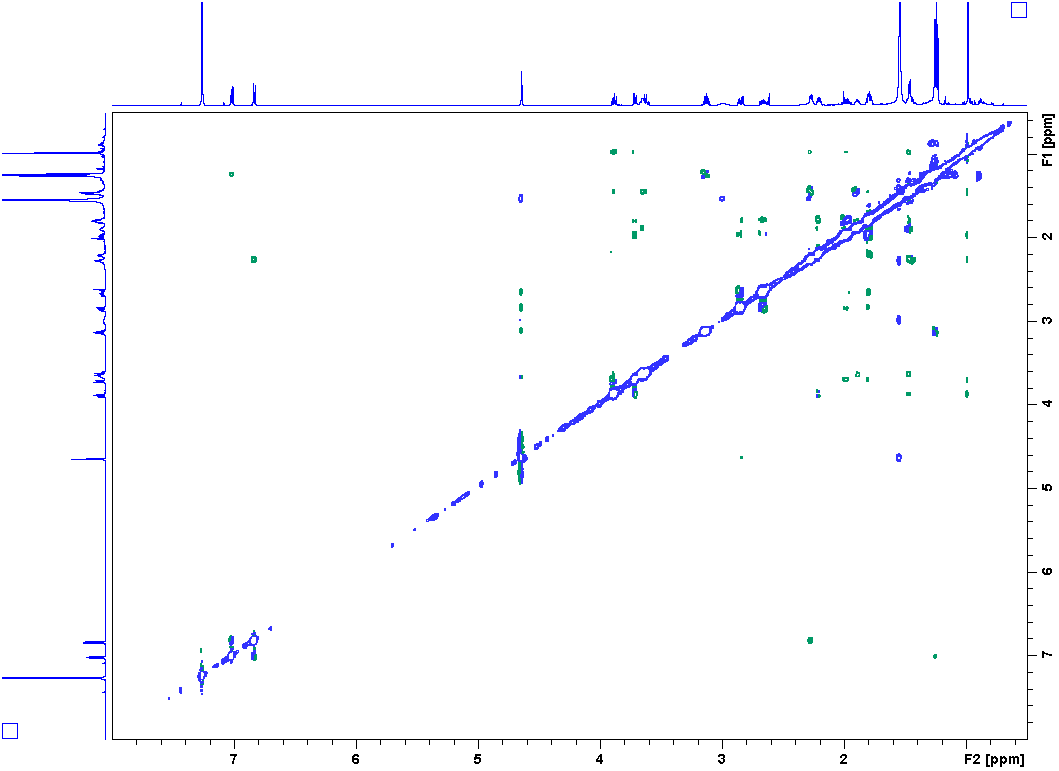


**b**

**Supplementary figure 28 NMR analysis of 18*S*(4→3)abeo-abietatrien-14,18,20-triol (12**): (**a**) HSQC, and (**b**) ROESY spectra of **12.** Couplings; see Supplementary table 8. Ref.: This work

**
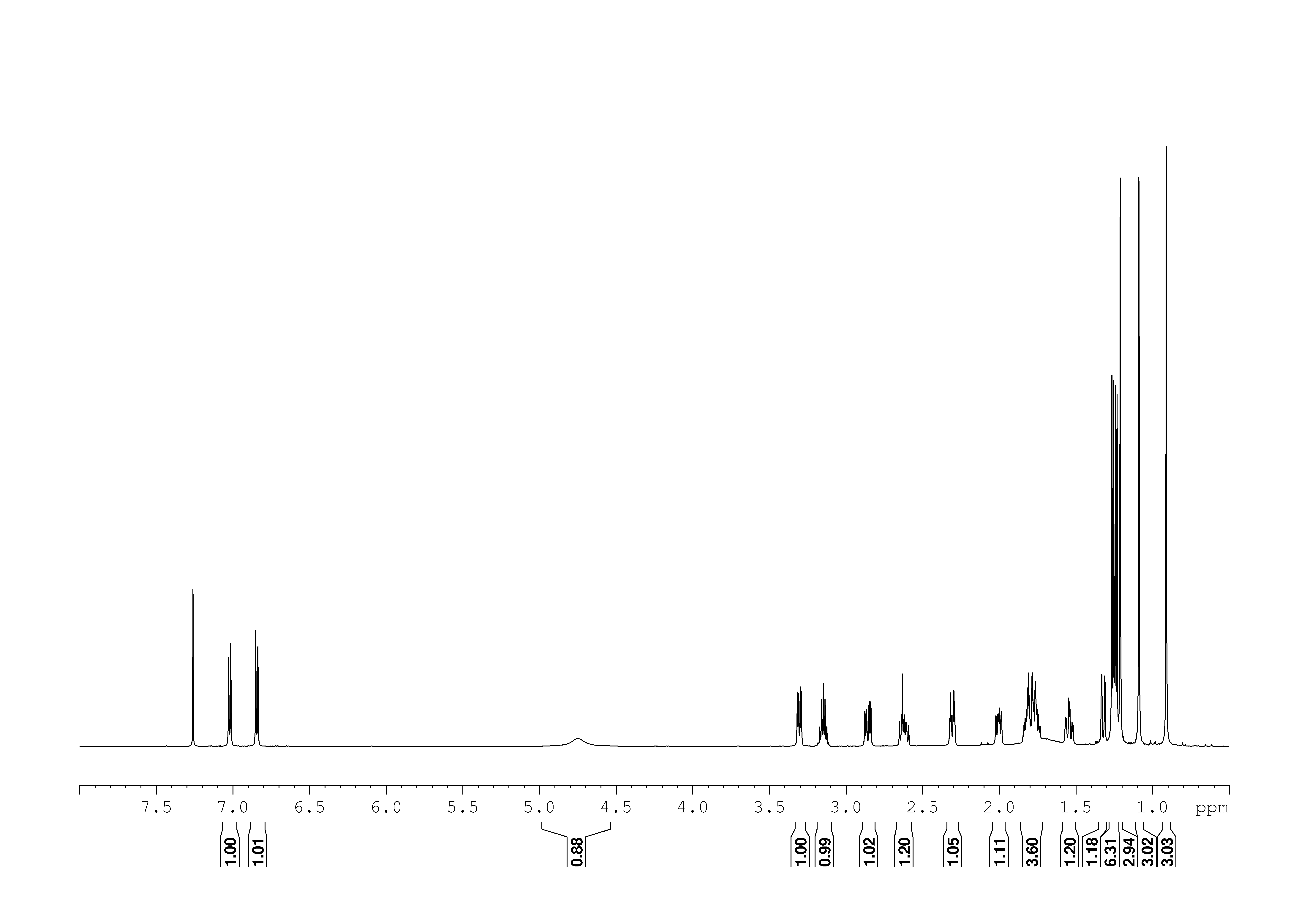
 Supplementary figure 29. NMR analysis of 3-epi-triptobenzene B (13):** 1H NMR spectrum (600.13 MHz, CDCl3). Couplings; see Supplementary table 9. Ref:^13^ & ^14^

**
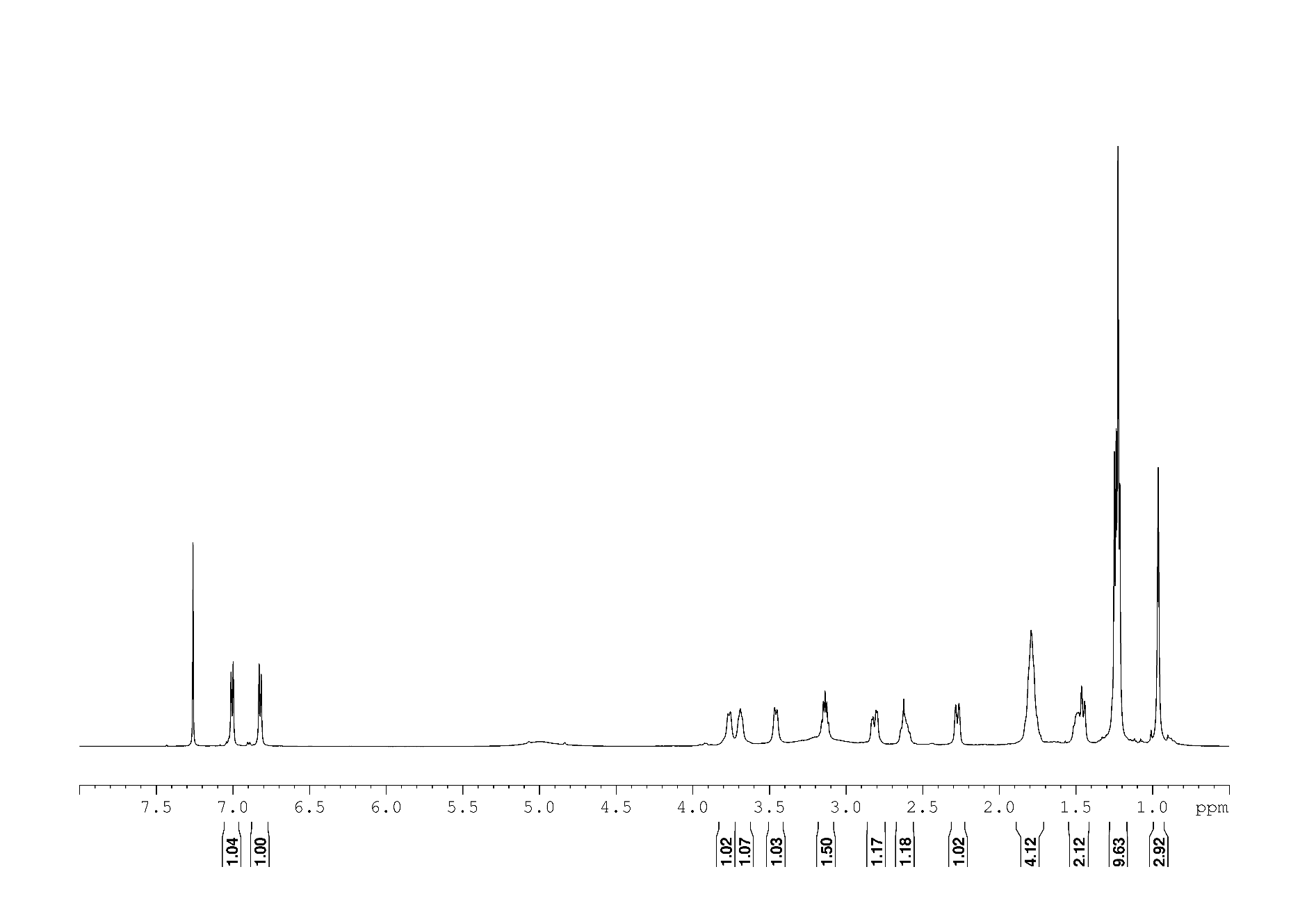
**

**Supplementary figure 30 NMR analysis of 4-epi-triptobenzene J (14**): ^1^H NMR spectrum (600.13 MHz, CDCl_3_). Couplings; see Supplementary table 10. Ref: This work, and ^15^


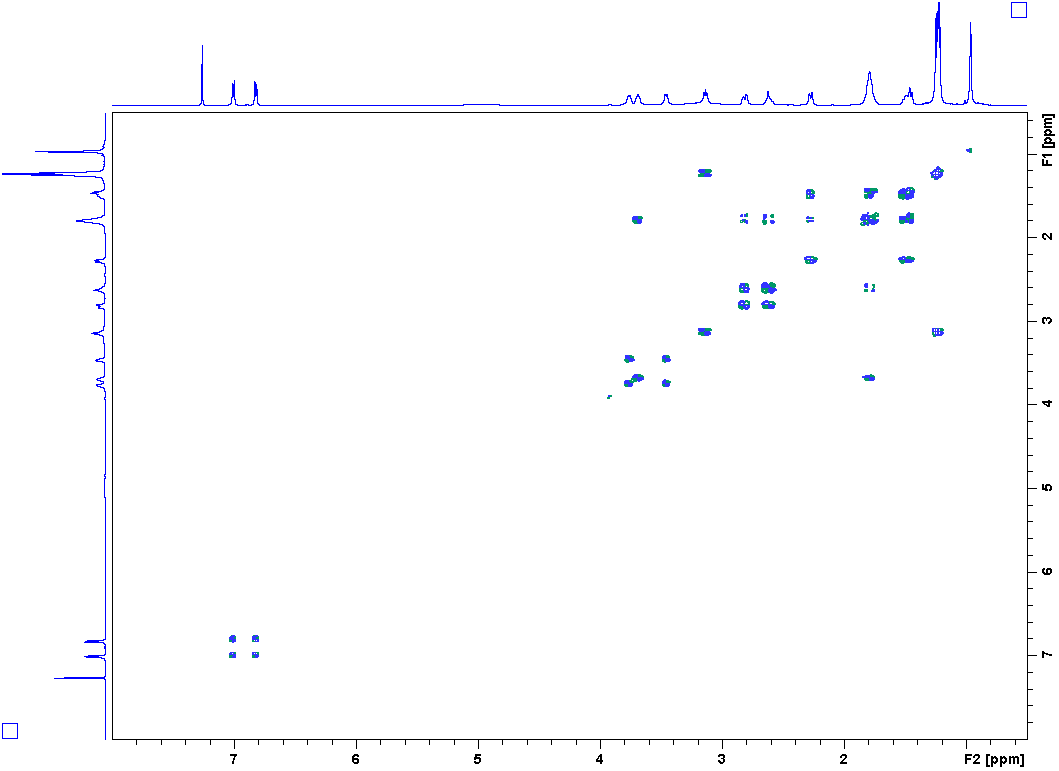
**
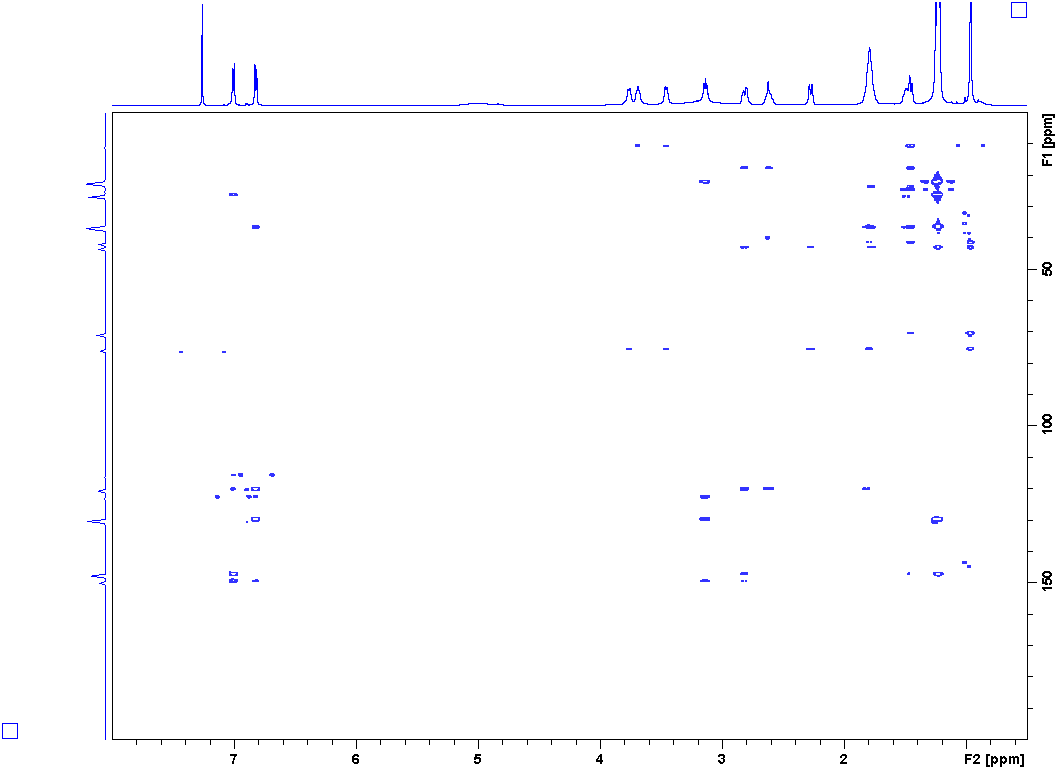
 Supplementary figure 31 NMR analysis of 4-epi-triptobenzene J (14**): (**a**) COSY, (**b**) HMBC. Couplings; see Supplementary table 10. Ref: This work, and ^15^

**b**

**a**


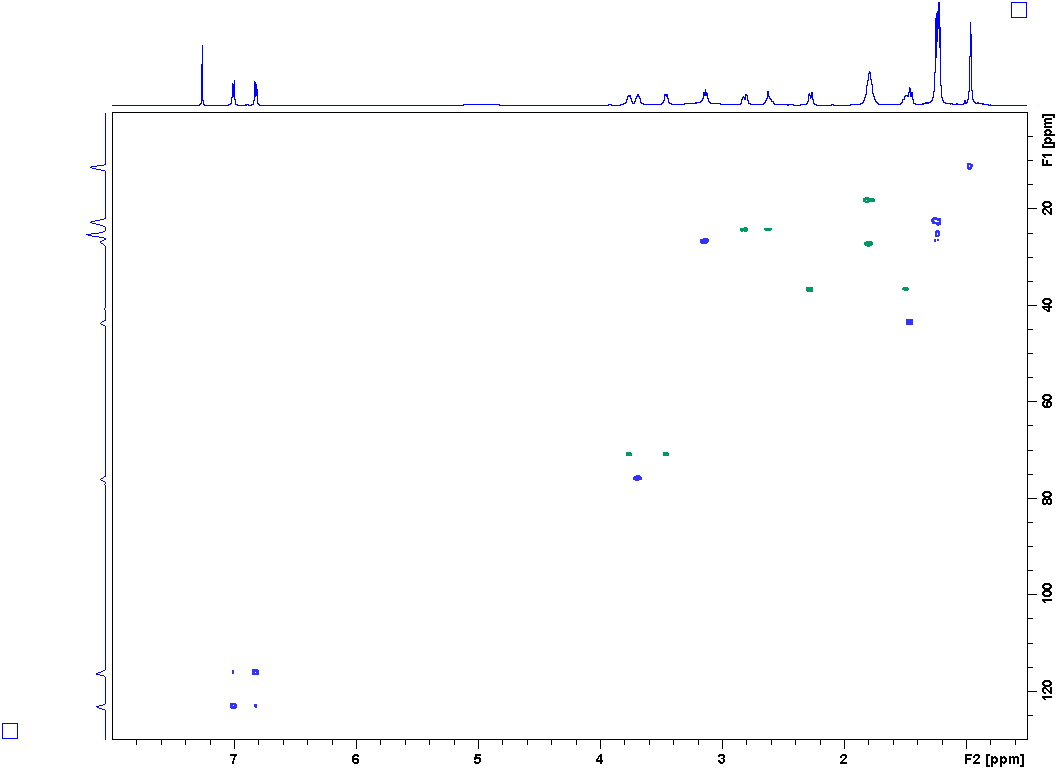


**a**


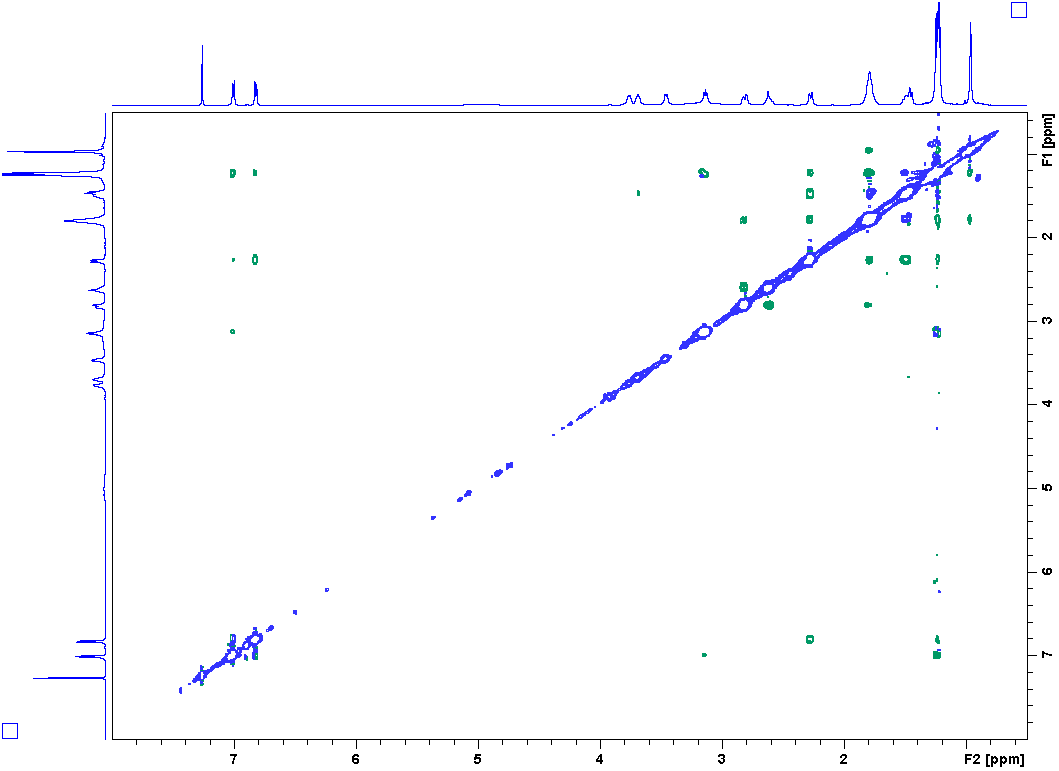


**b**

**Supplementary figure 32 NMR analysis of 4-epi-triptobenzene J (14**): (**a**) HSQC and (b) ROESY spectra of **14**; see Supplementary table 10. Ref: This work, and ^15^


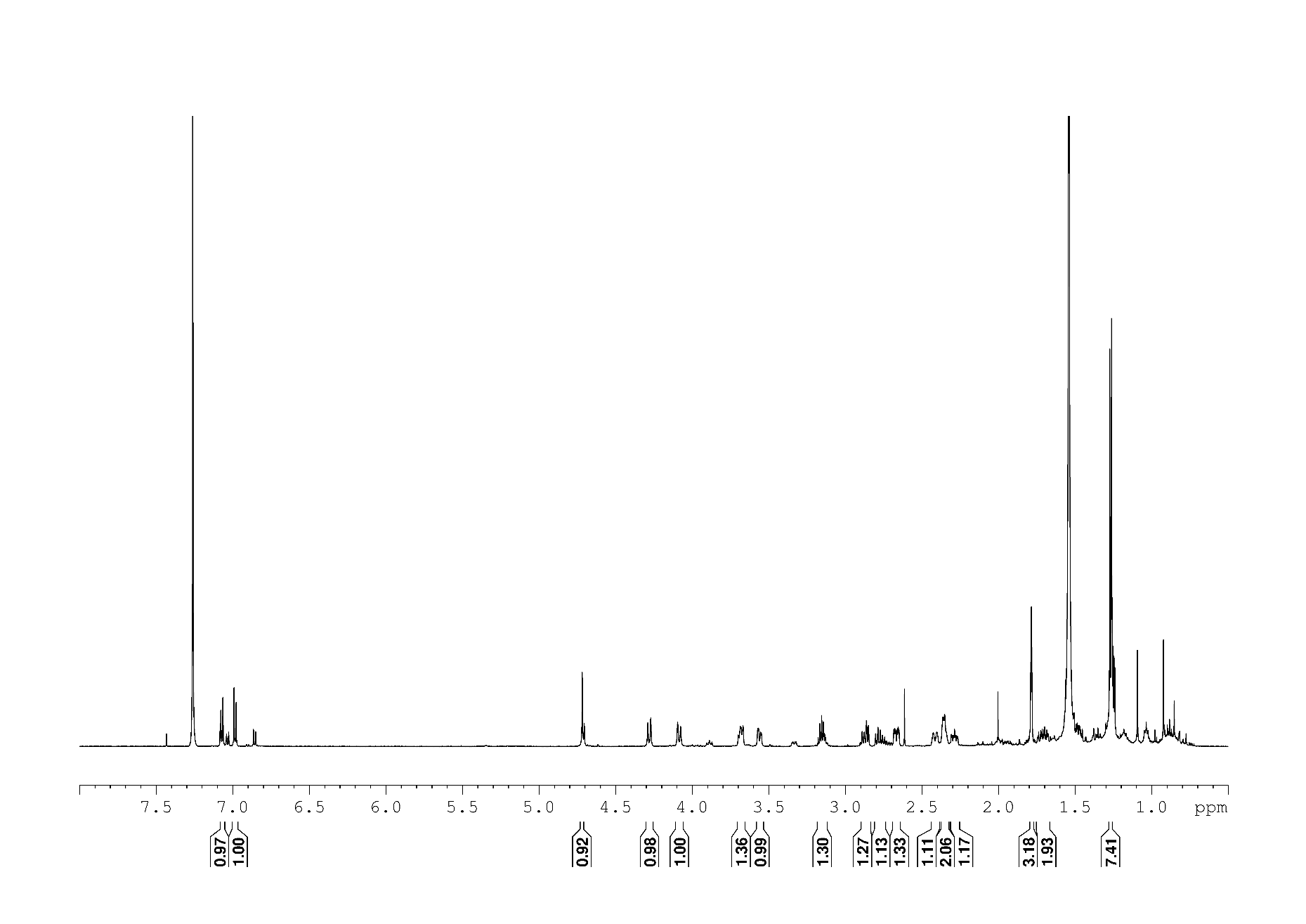
 **Supplementary figure 33 NMR analysis of 18(4→3)abeo-abietatrien-14,18,20-triol (15):** ^1^H NMR spectrum (600.13 MHz, CDCl_3_) ; see Supplementary table 11**.** Ref.: This work


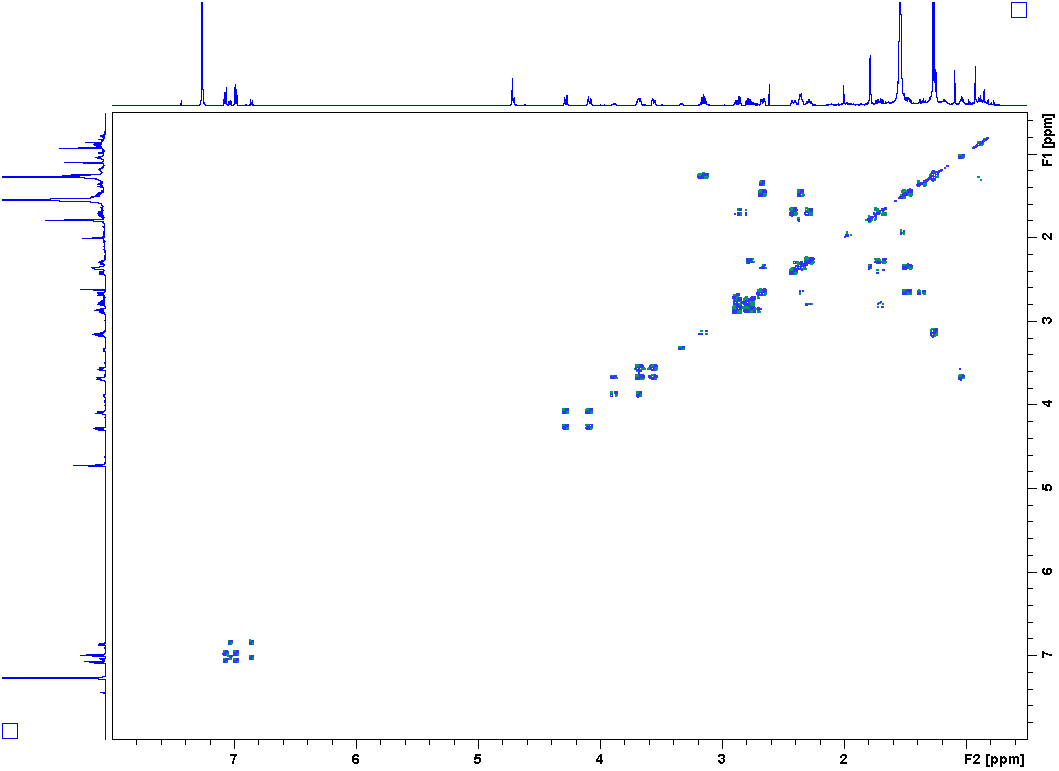


**a**


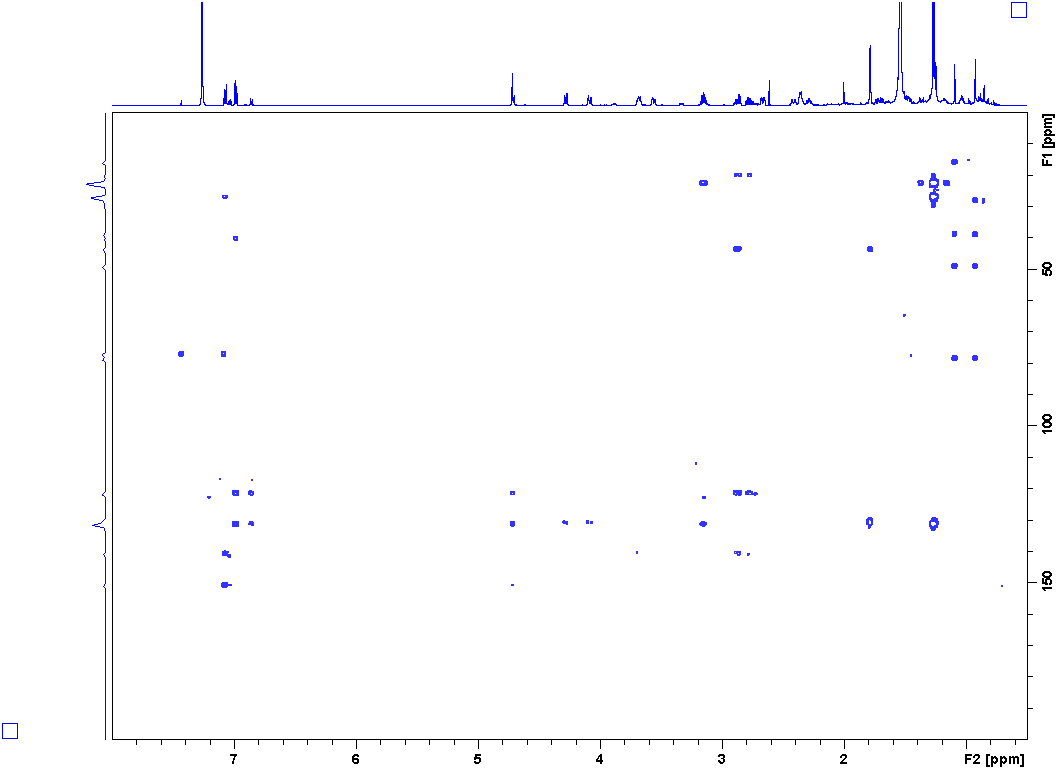


**b**

**Supplementary figure 34 NMR ananlysis of 18(4→3)abeo-abietatrien-14,18,20-triol (15):** (**a**) COSY, (**b**) HMBC spectra of **15**. Couplings; see Supplementary table 11. Ref.: This work


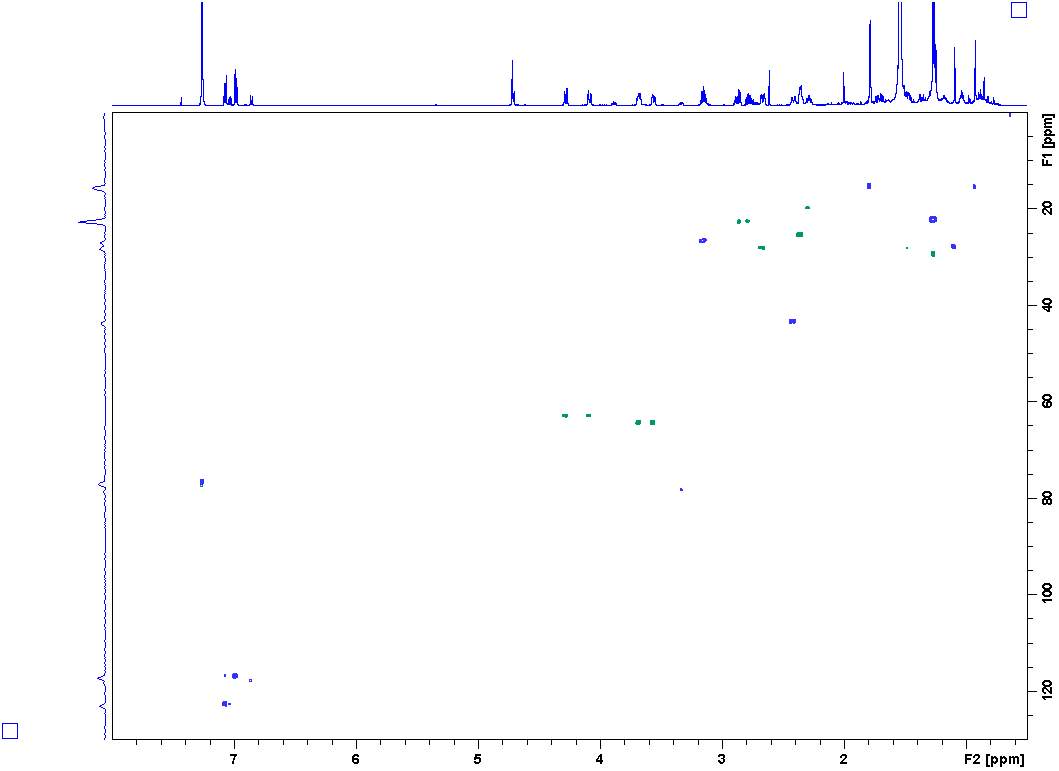

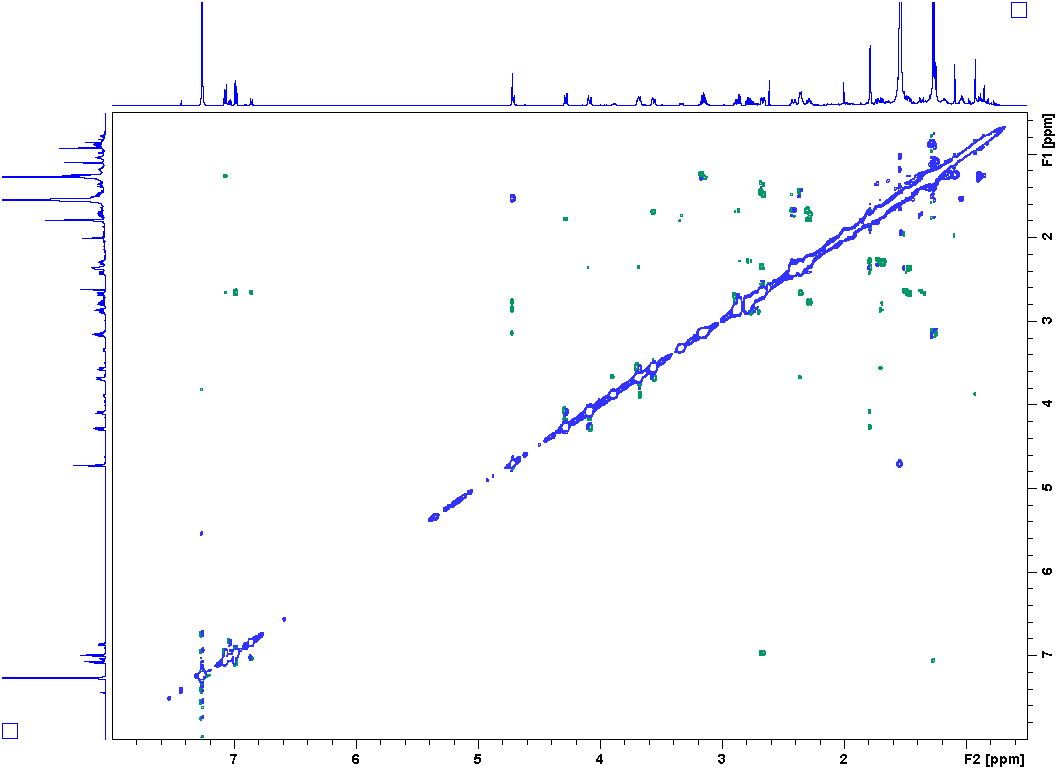


**b**

**a**

**Supplementary figure 35 NMR ananlysis of 18(4→3)abeo-abietatrien-14,18,20-triol (15):** (**a**) HSQC and (**b**) ROESY spectra of **15**. Couplings; see Supplementary table 11. Ref.: This work

**
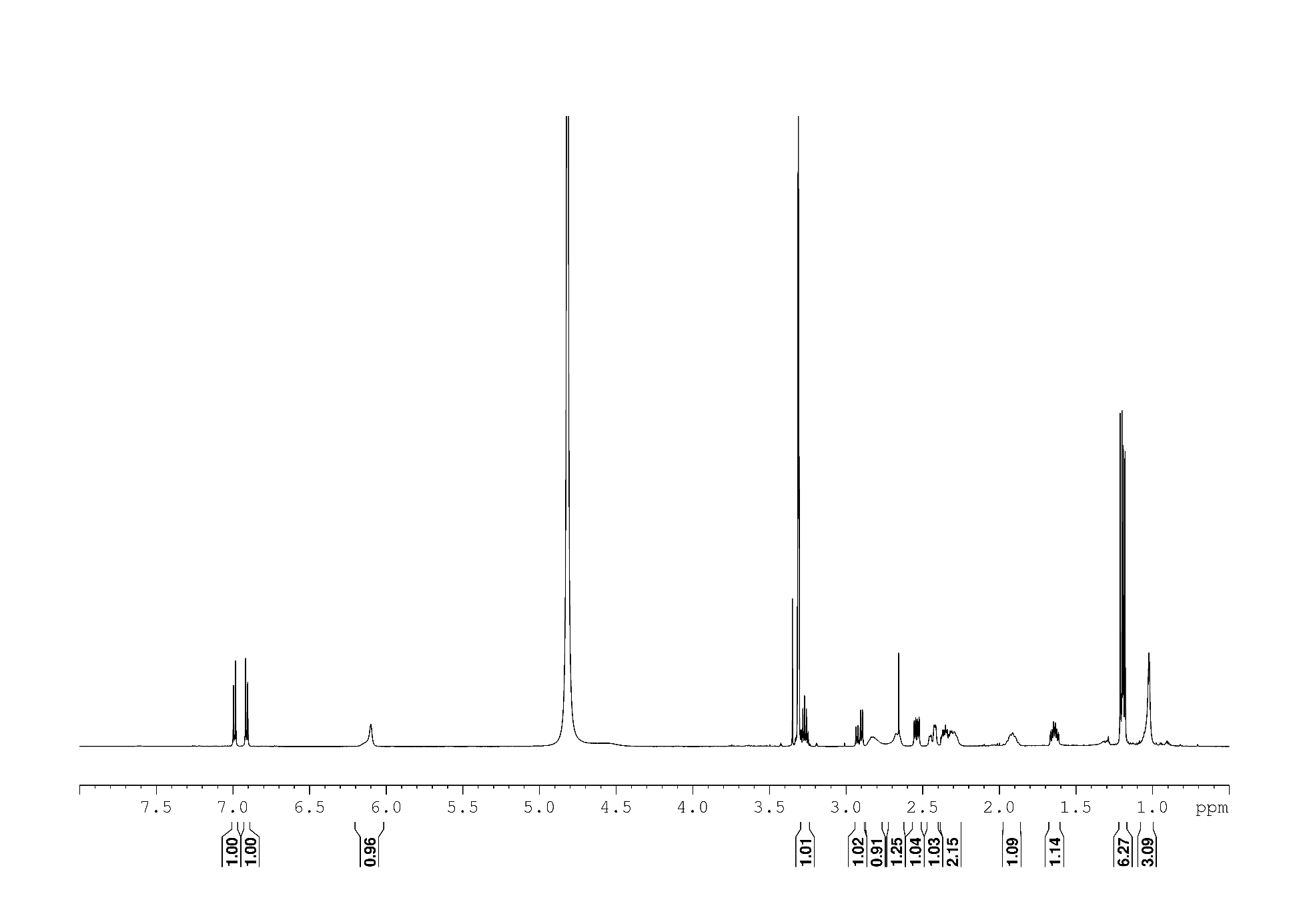
**

**Supplementary figure 36 of triptobenzene I (16**): ^1^H NMR spectrum (600.13 MHz, methanol-*d*_4_) of triptobenzene I **16**. Couplings; see supplementary table 12. Ref.: ^16^


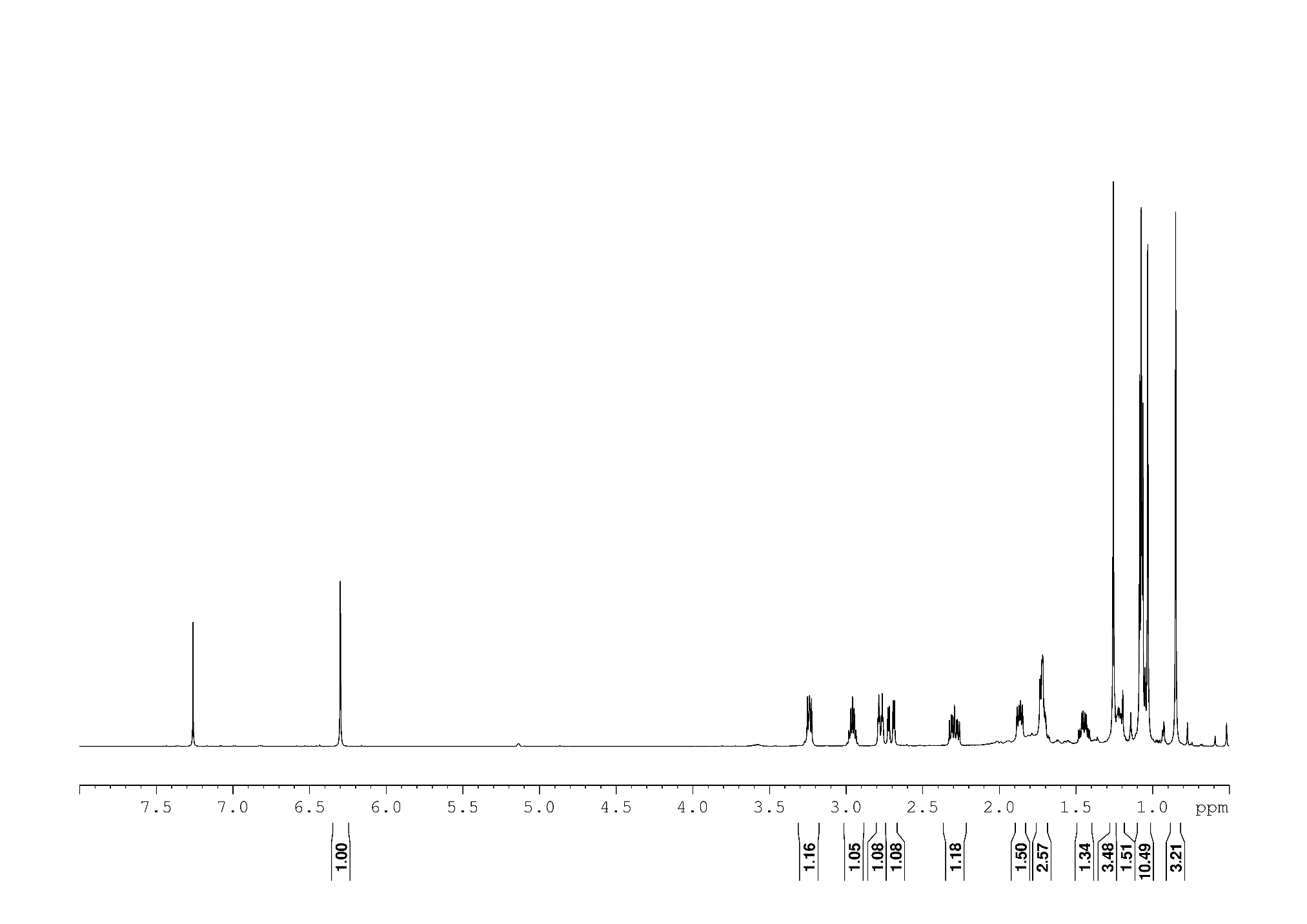


**Supplementary figure 37 of 3β-hydroxy-11,14-oxo-abieta-8,12-diene (17):** ^1^H NMR spectrum (600.13 MHz, CDCl_3_). Couplings; see supplementary table 13. Ref.: ^17^


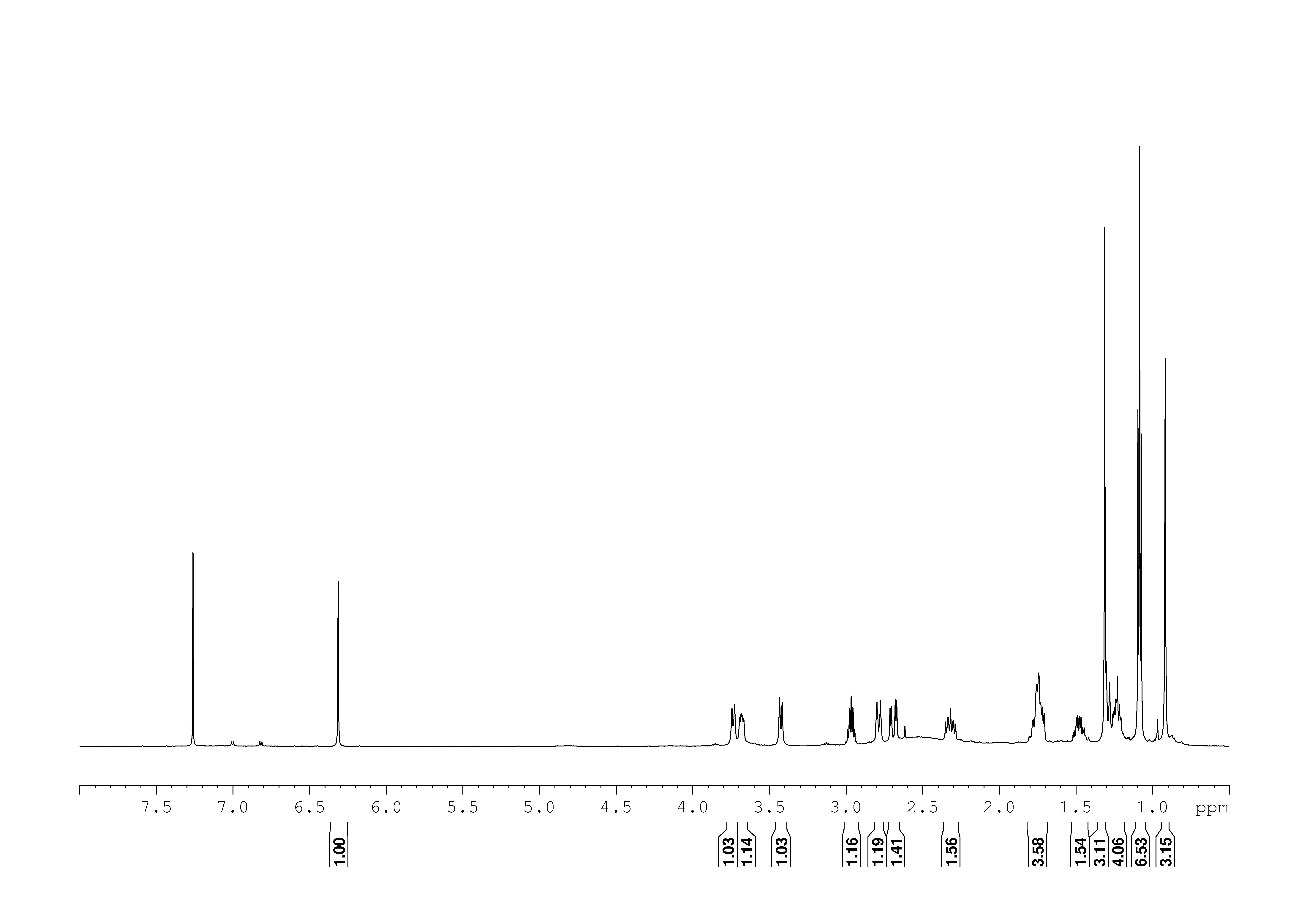
 **Supplementary figure 38 NMR analysis of 4-epi-triptoquinone C (18):** ^1^H NMR spectrum (600.13 MHz, CDCl_3_) of **18**. Couplings; see supplementary table 14. Ref.: This work and ^18^.


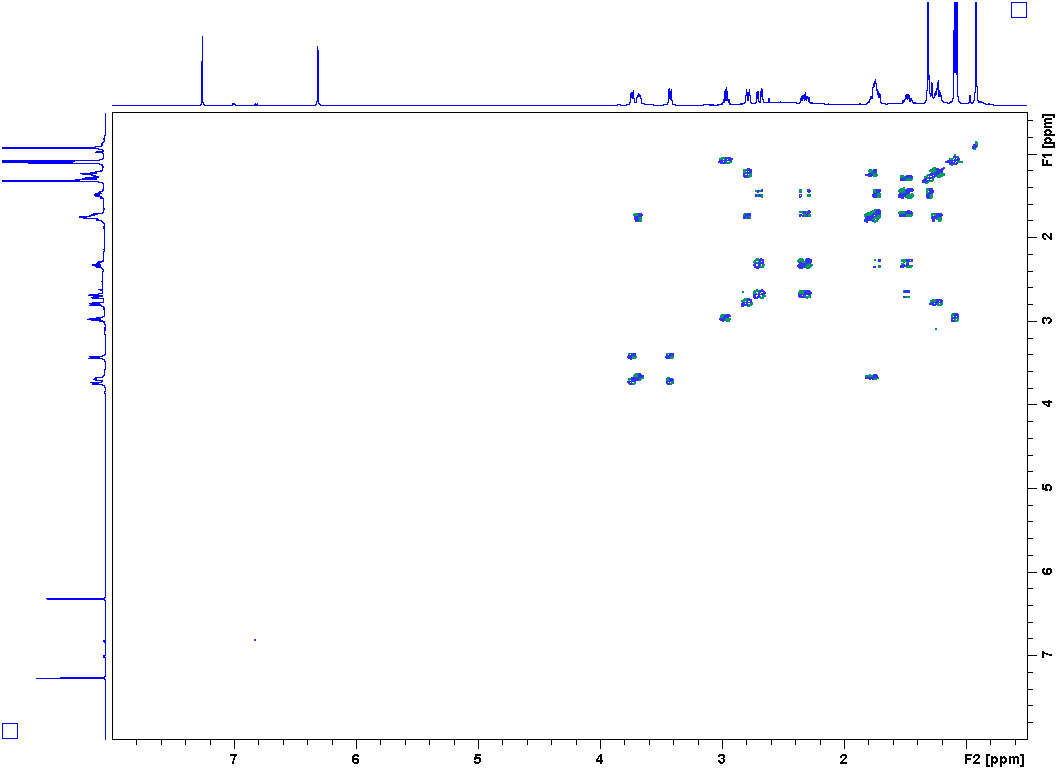

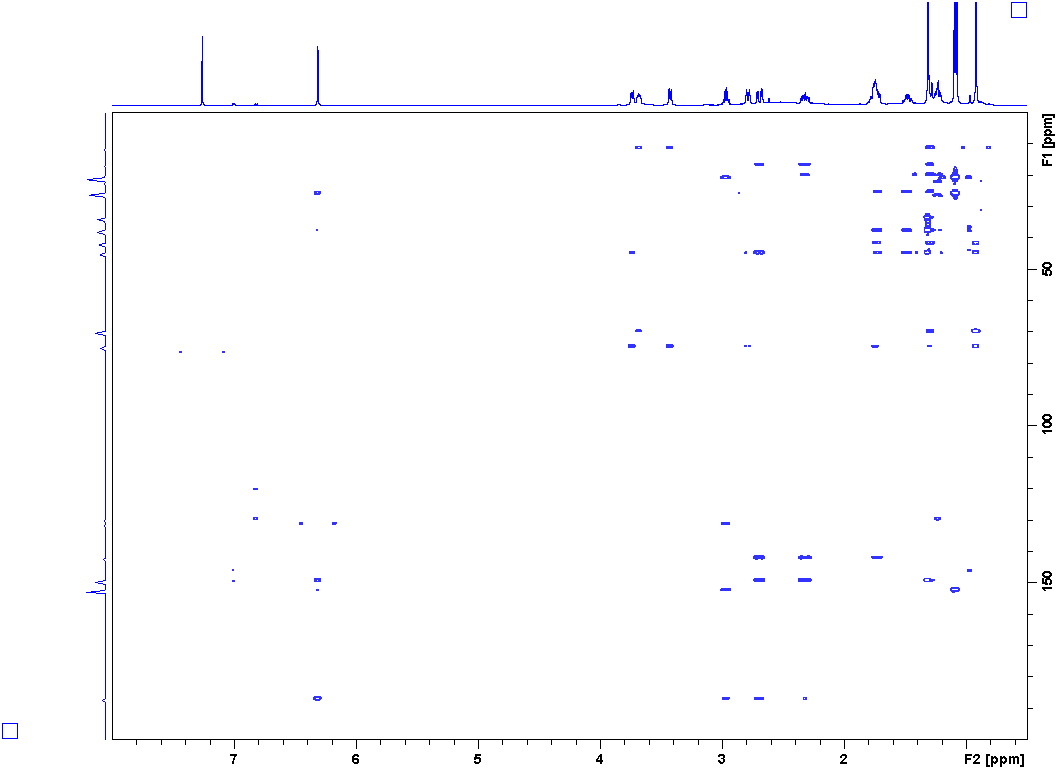


**b**

**a**

**Supplementary figure 39 NMR analysis of 4-epi-triptoquinone C (18):** (**a**) COSY, and (**b**) HMBC spectra of **18**. Couplings; see supplementary table 14. Ref.: This work and ^18^


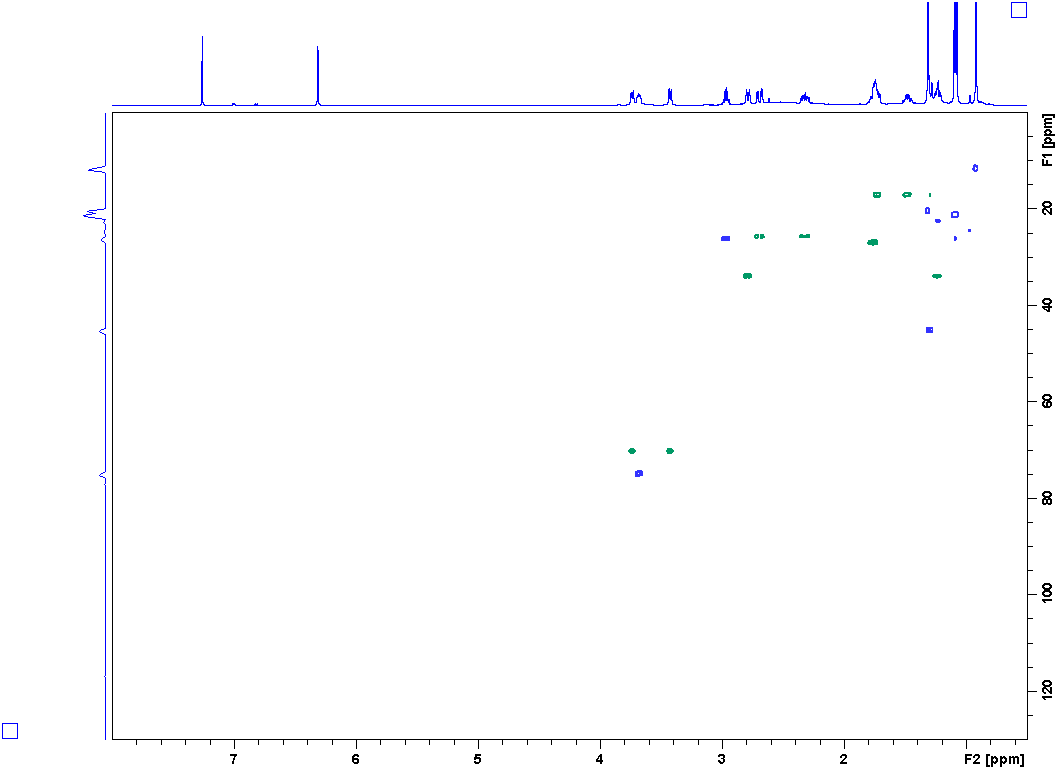


**a**


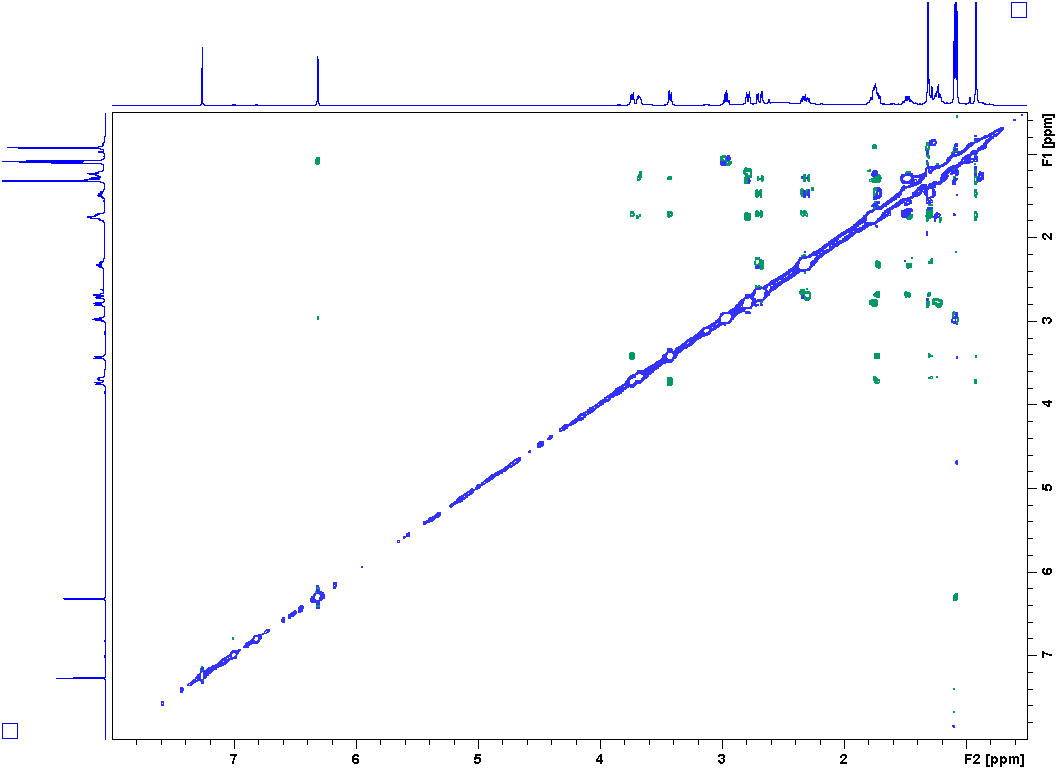


**b**

**Supplementary figure 40 NMR analysis of of 4-epi-triptoquinone C (18):** (**a**) HSQC and (b) ROESY spectra of **18**. Couplings; see supplementary table 14. Ref.: This work and ^18^

**
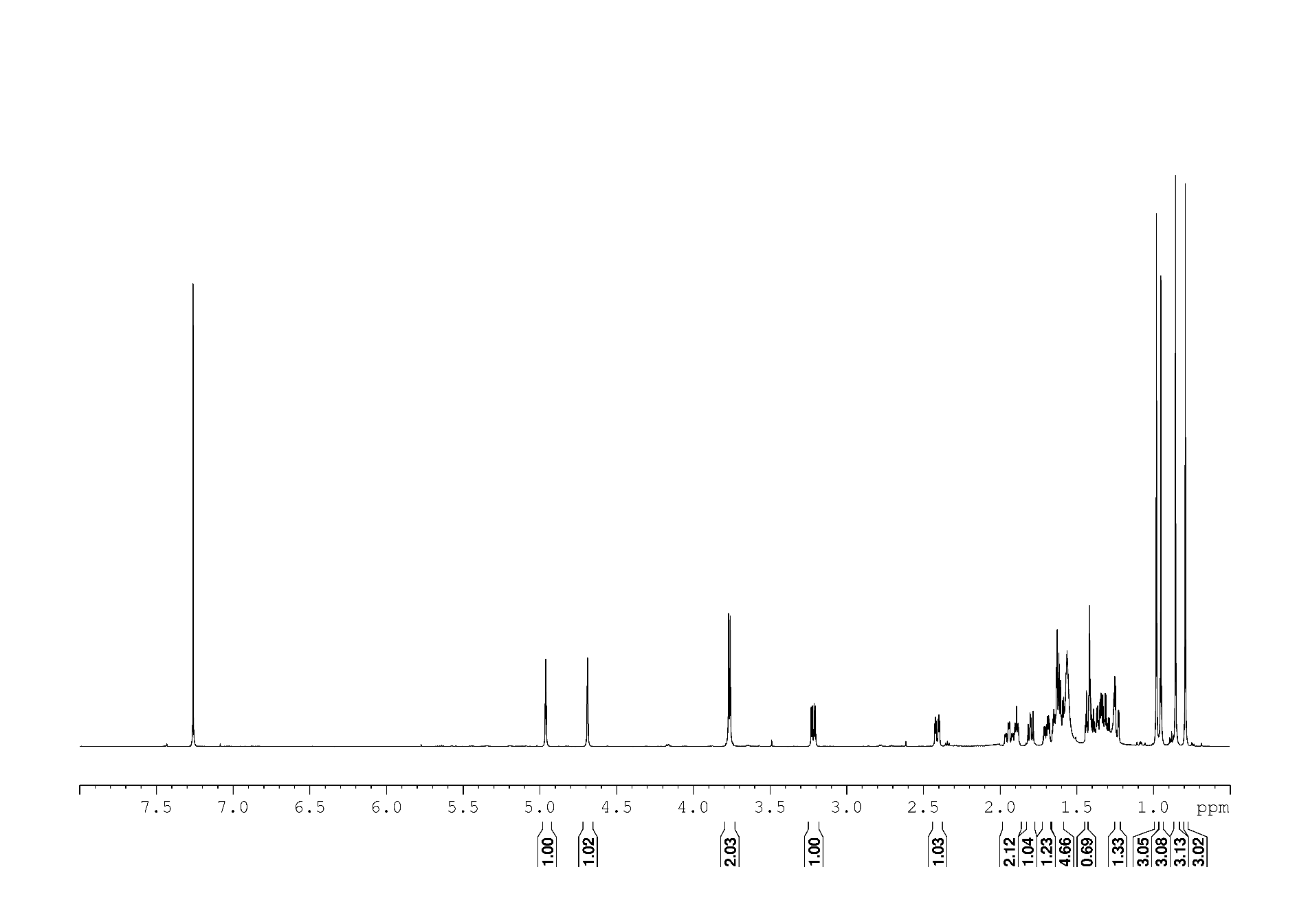
**

**Supplementary figure 41 NMR analysis of (+)isocopal-13(16)-en-3,14-diol (19):** ^1^H NMR spectrum (600.13 MHz, CDCl_3_) of **19**. Couplings; see supplementary table 15. Ref.: This work


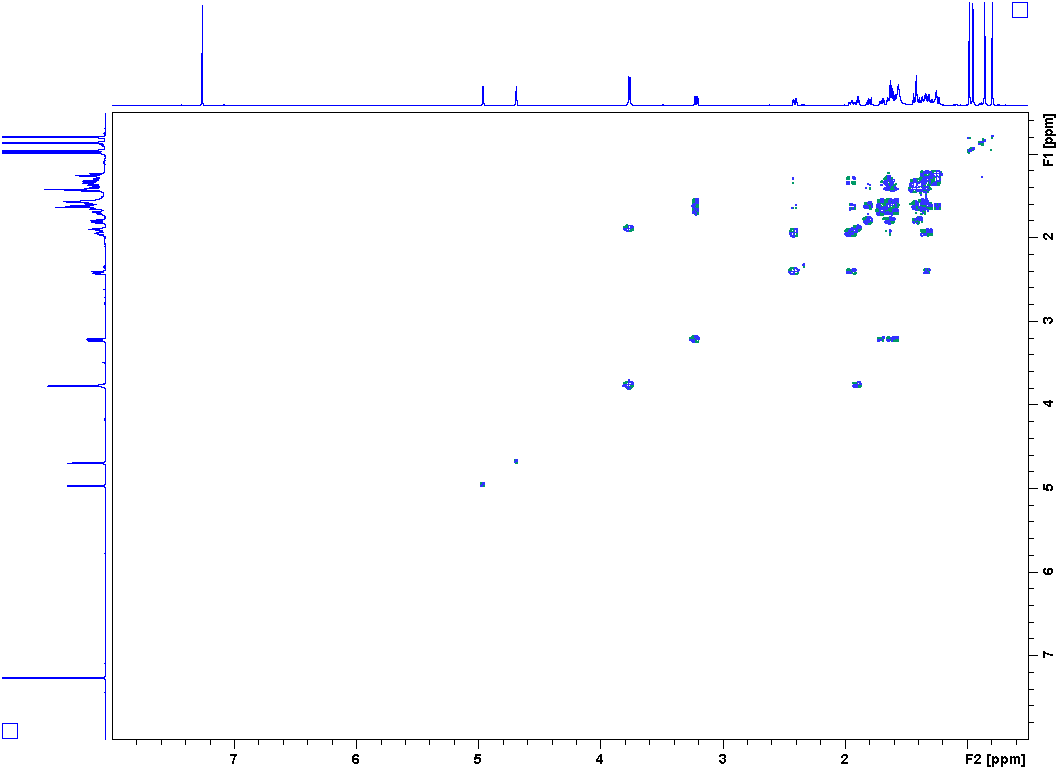


**a**


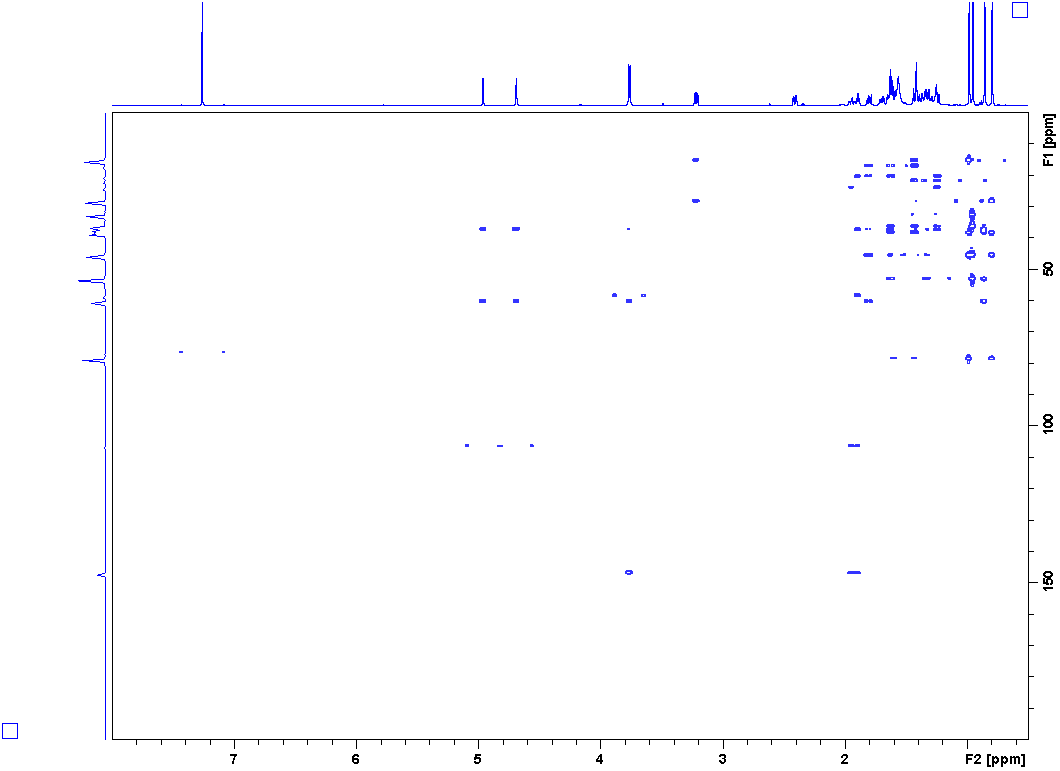


**b**

**Supplementary figure 42 NMR analysis of (+)isocopal-13(16)-en-3,14-diol (19):** (**a**) COSY, and (**b**) HMBC data of **19.** Couplings; see supplementary table 15. Ref.: This work


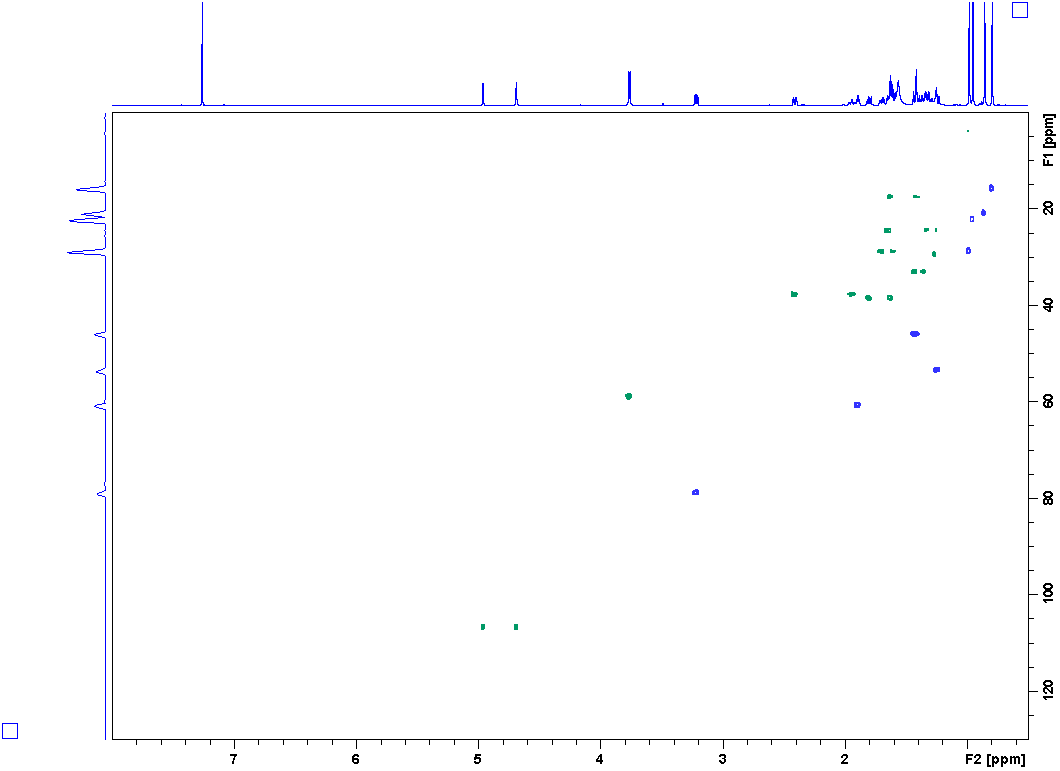

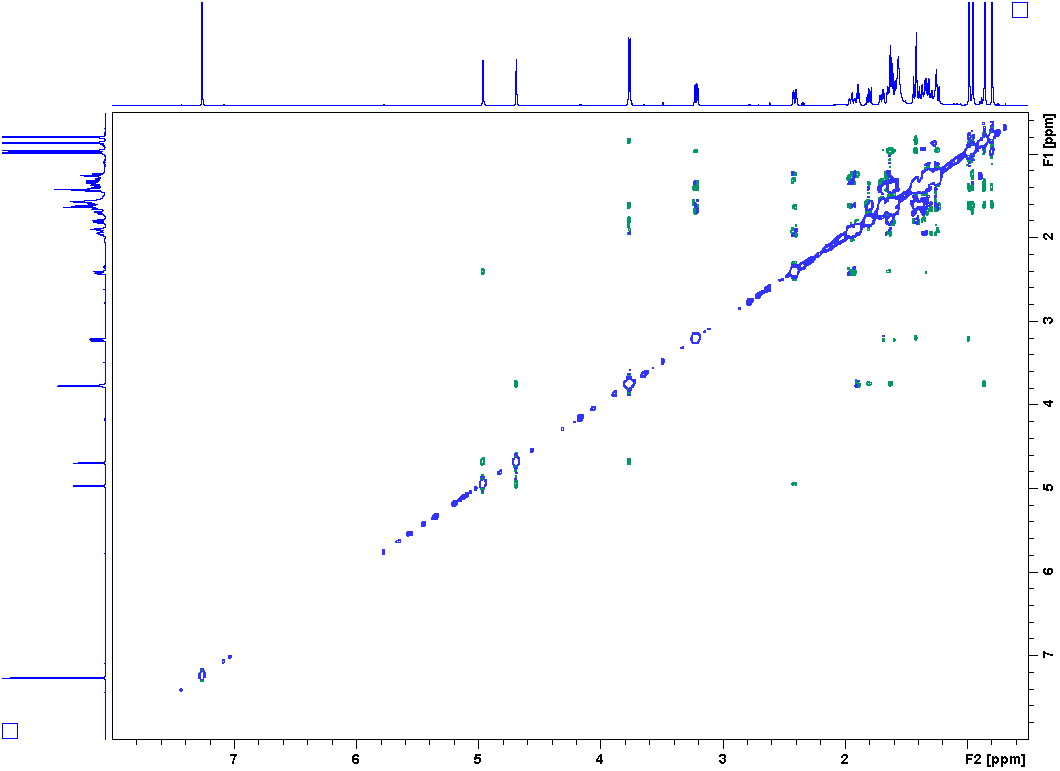


**b**

**a**

**Supplementary figure 43 NMR analysis of (+)isocopal-13(16)-en-3,14-diol (19):** (**a**) HSQC and (**b**) ROESY data of **19.** Couplings; see supplementary table 15. Ref.: This work

**
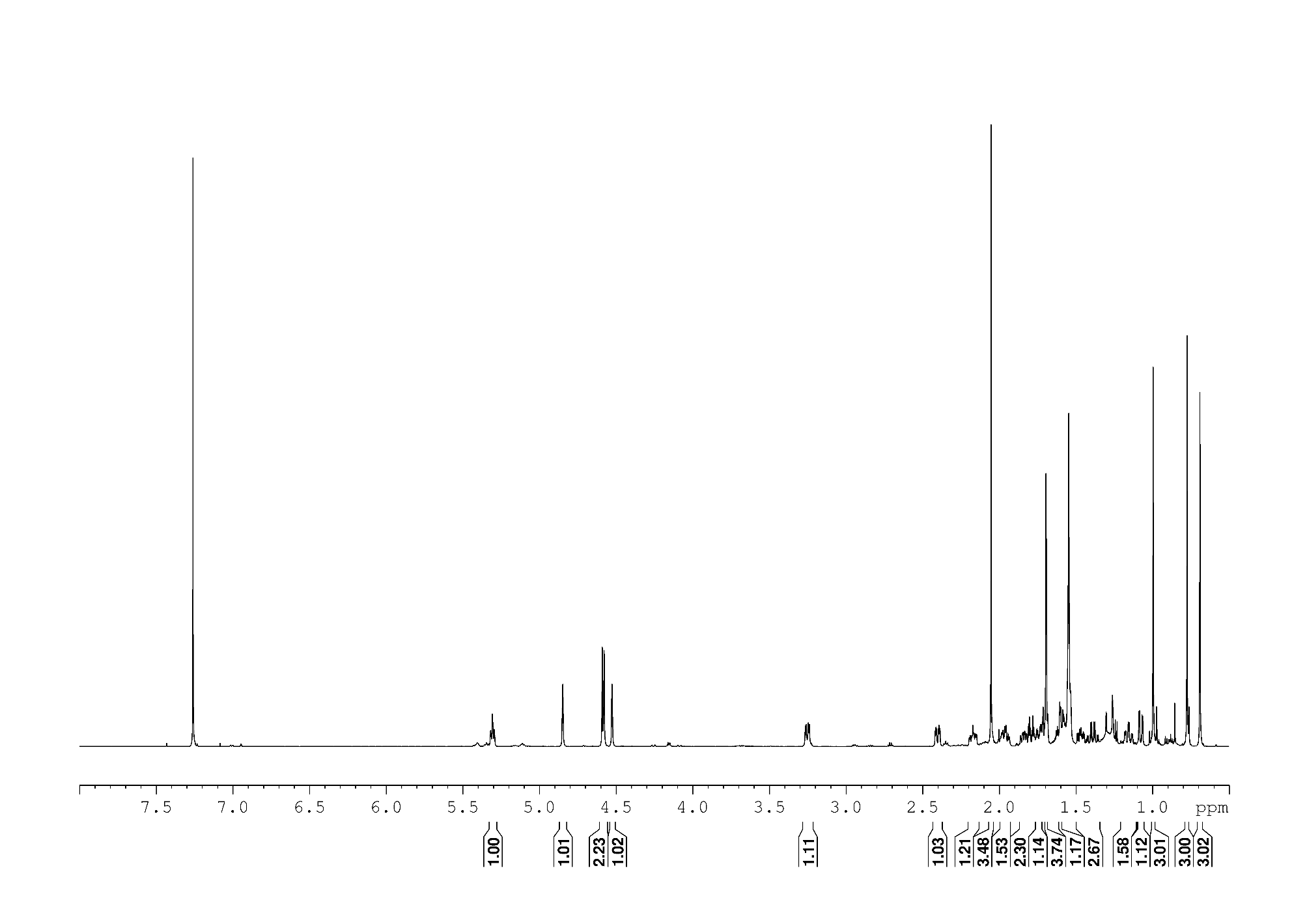
 Supplementary figure 44 NMR analysis of labda-8(17)-13(14)en-15-O-acetate-3-ol (20):** ^1^H NMR spectrum (600.13 MHz, CDCl_3_) spectra of **20.** Couplings; see supplementary table 16. Ref.: This work


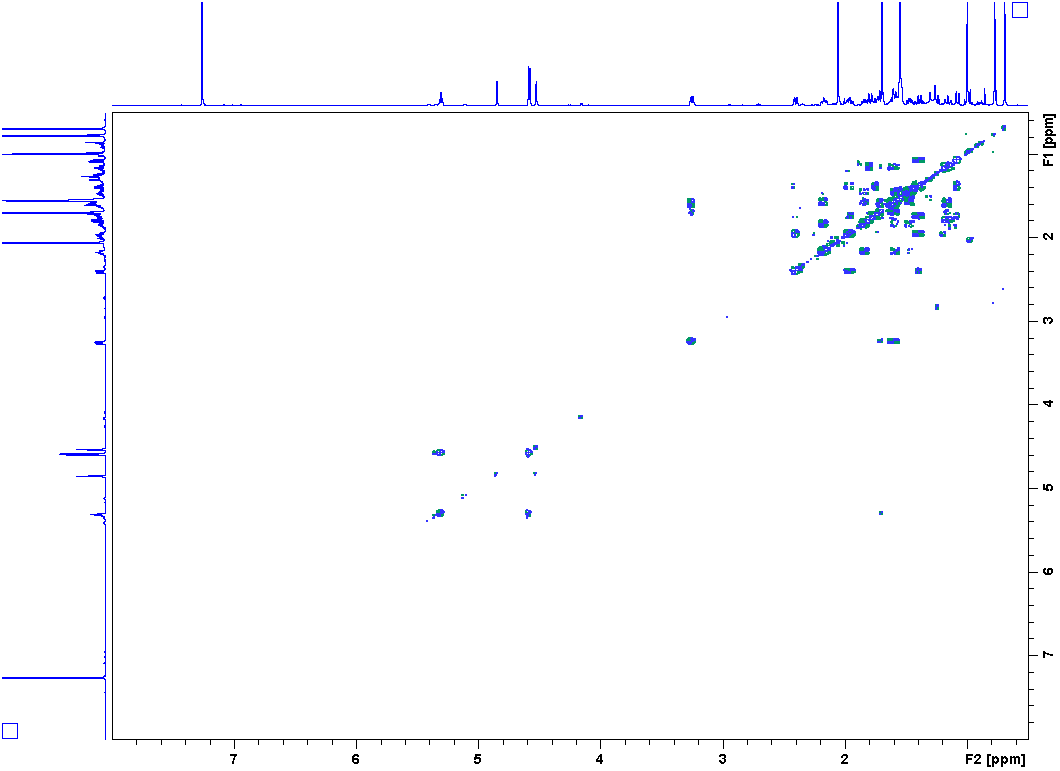

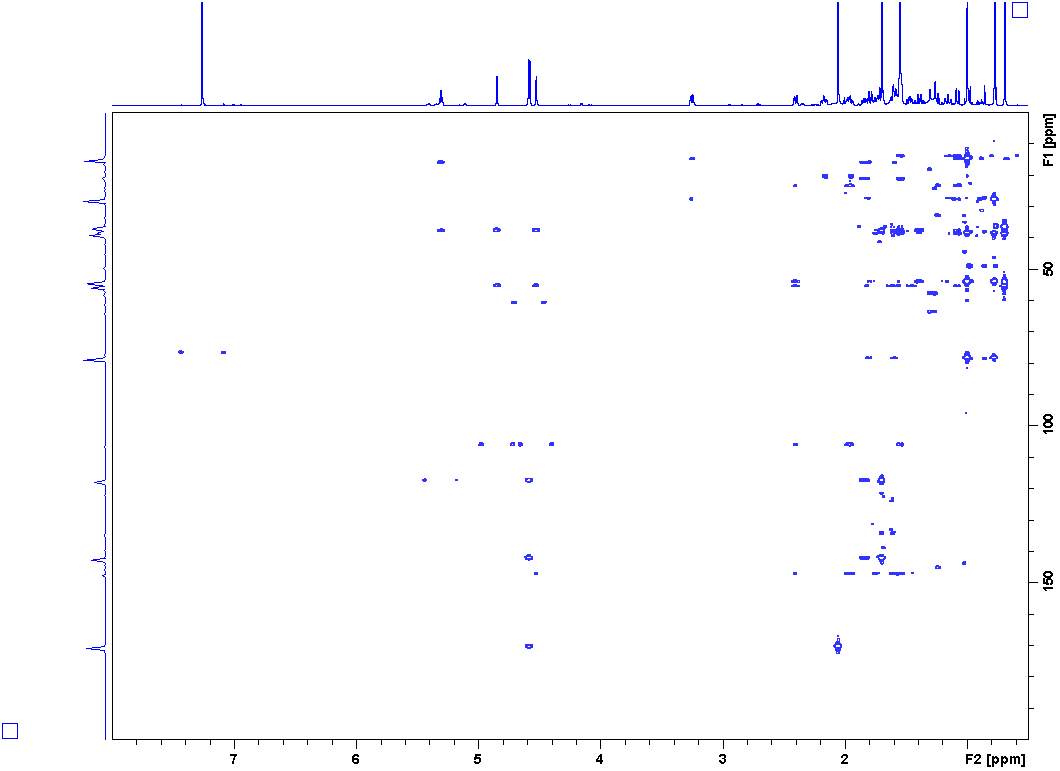


**b**

**a**

**Supplementary figure 45 NMR analysis of labda-8(17)-13(14)en-15-O-acetate-3-ol (20): (a**) COSY, and (**b**) HMBC spectra of **20.** Couplings; see supplementary table 16. Ref.: This work


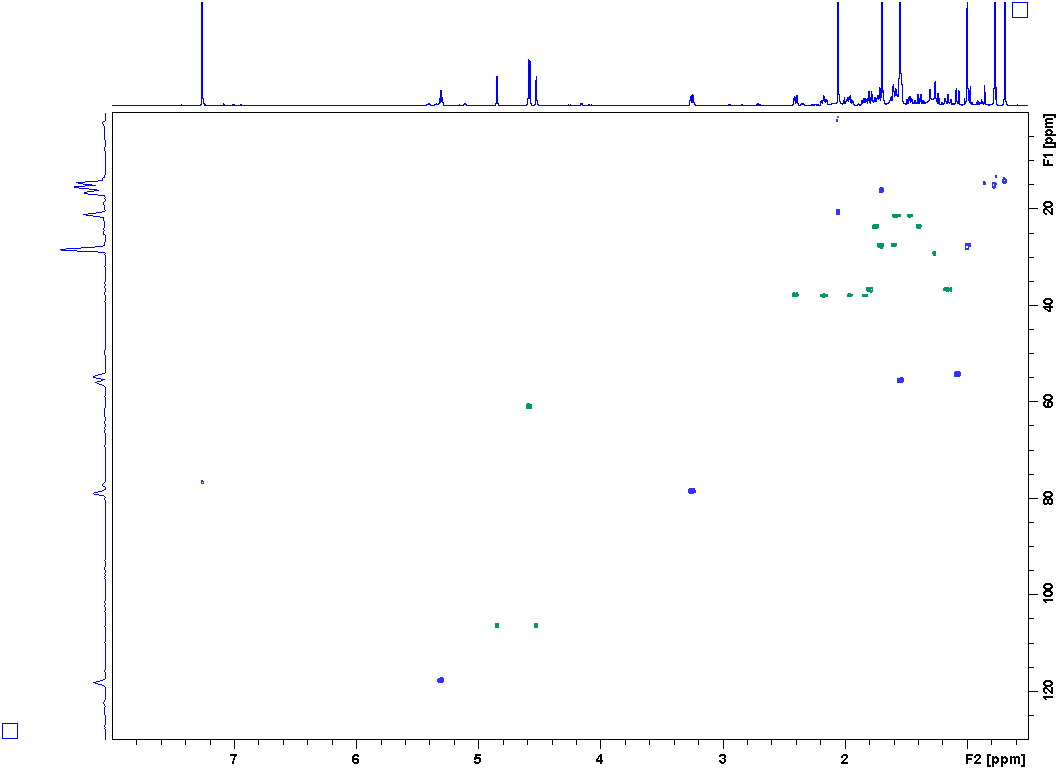

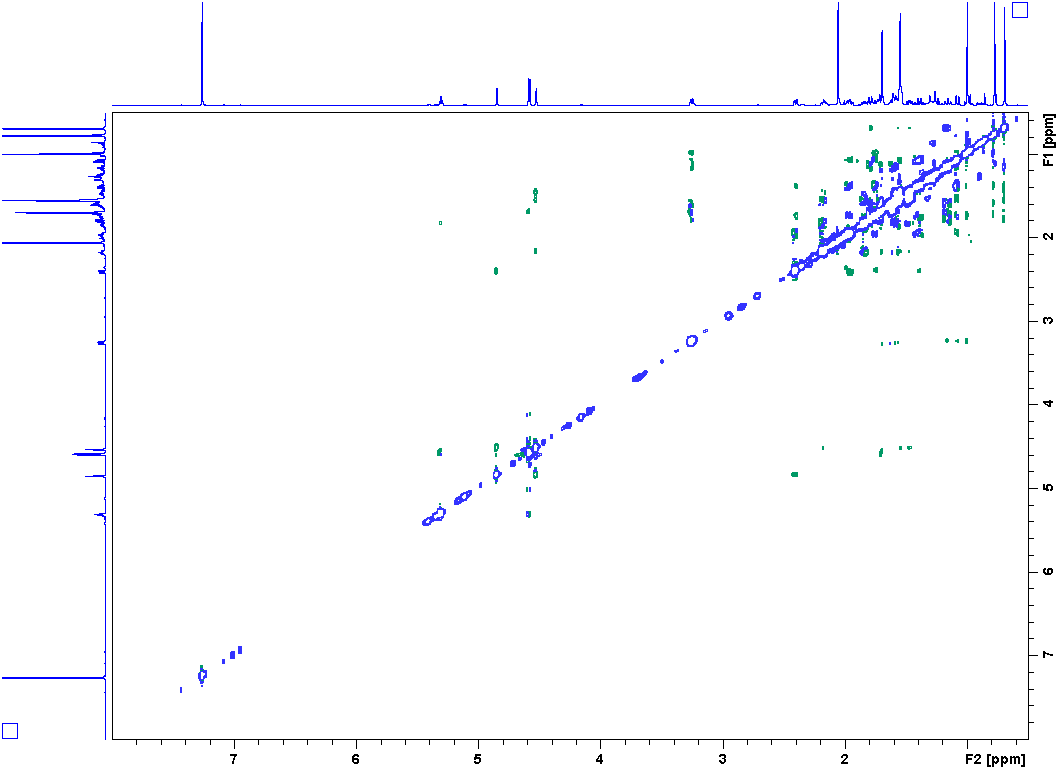
 **Supplementary figure 46 NMR analysis of labda-8(17)-13(14)en-15-O-acetate-3-ol (20): (a**) HSQC and (**b**) ROESY spectra of **20**. Couplings; see supplementary table 16. Ref.: This work

**b**

**a**

**
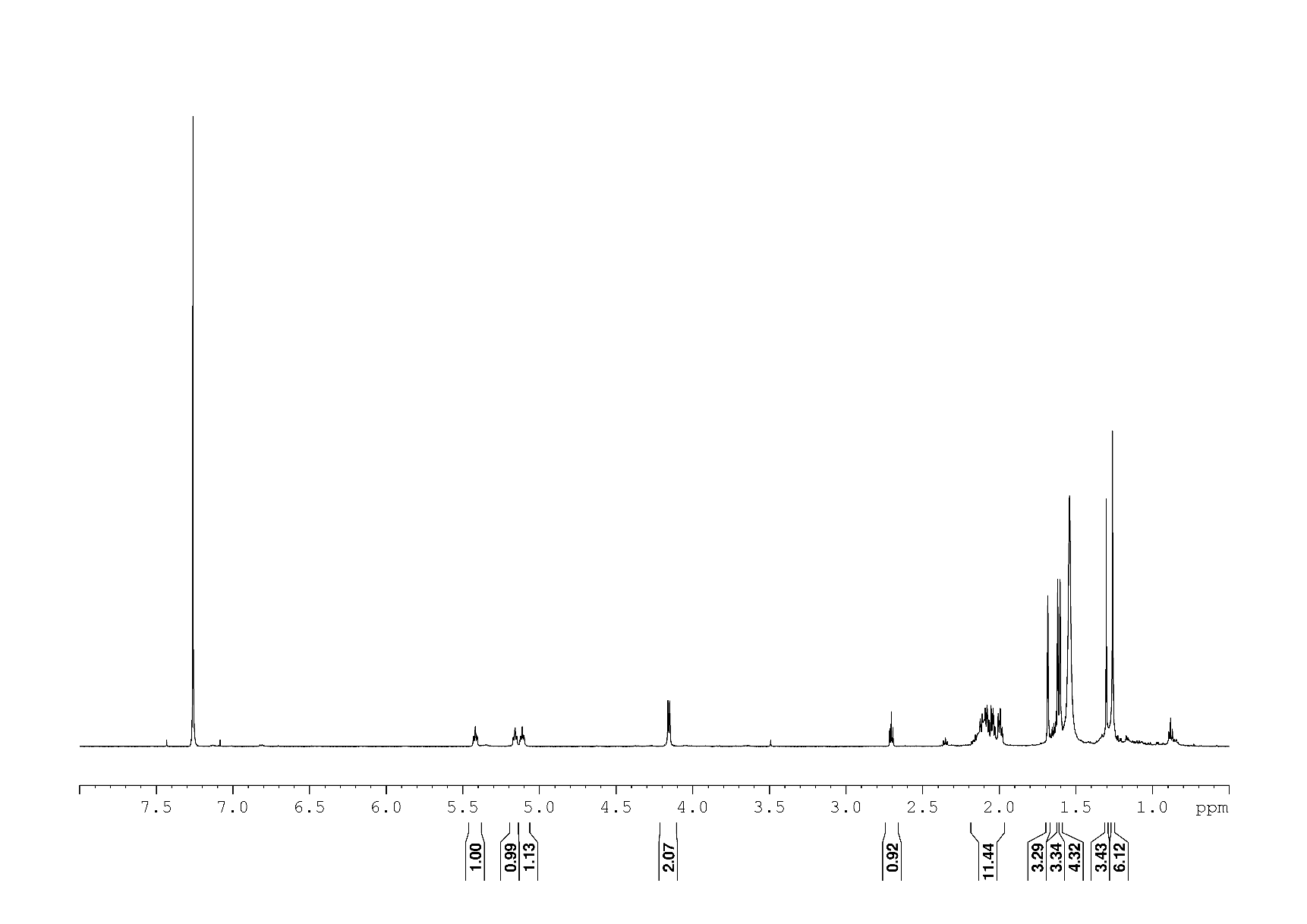
**

**Supplementary figure 47** ^1^H NMR spectrum (600.13 MHz, CDCl_3_) of 14,15-epoxygeranylgeraniol **21**. Couplings; see supplementary table 17. Ref.: ^19,20^

**
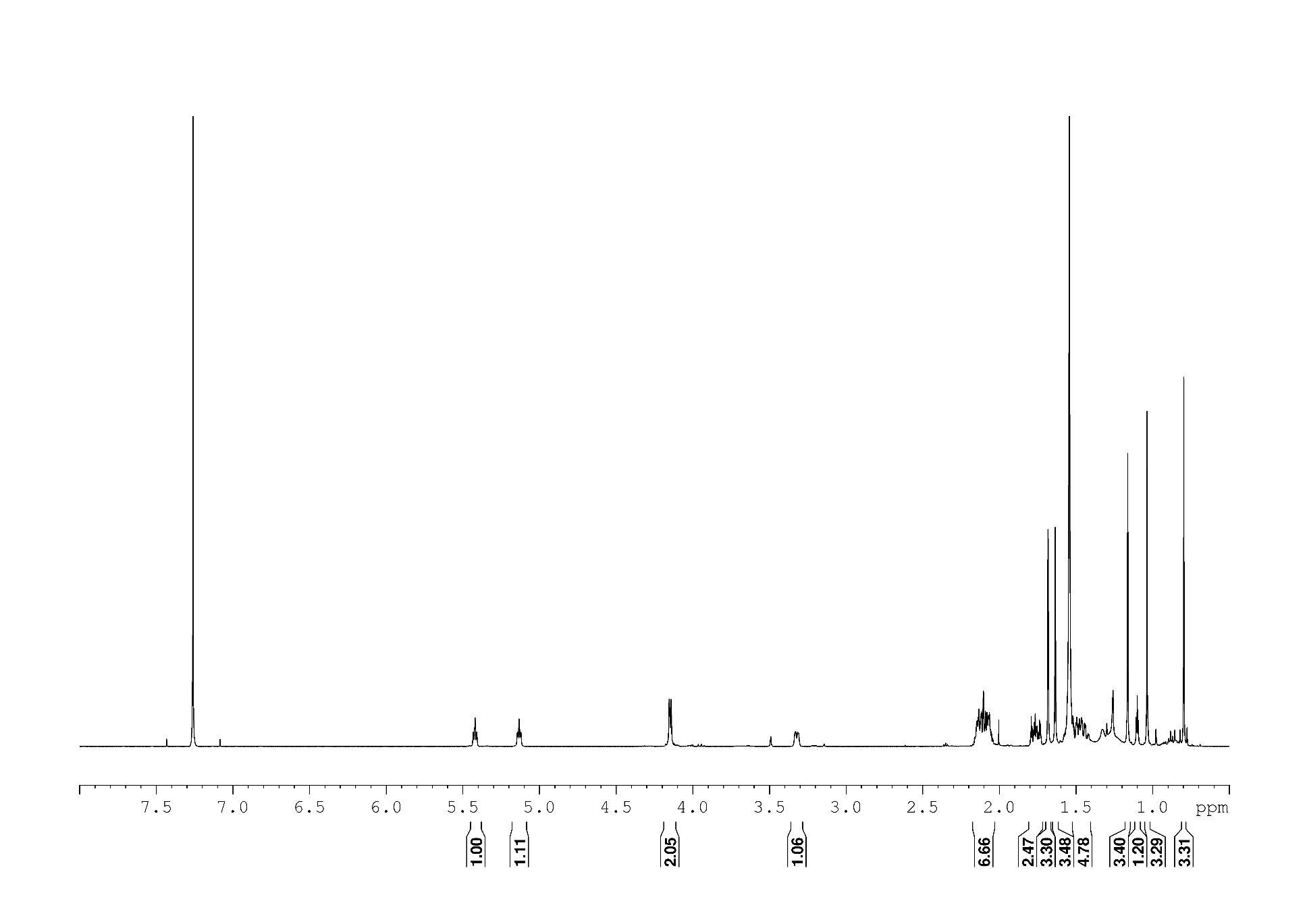
 Supplementary figure 48 NMR analysis of trimethylcyclohexane-11,14-diolgeraniol (22):** ^1^H NMR spectrum (600.13 MHz, CDCl_3_) spectra of **22**. Couplings; see supplementary table 18. Ref.: This work


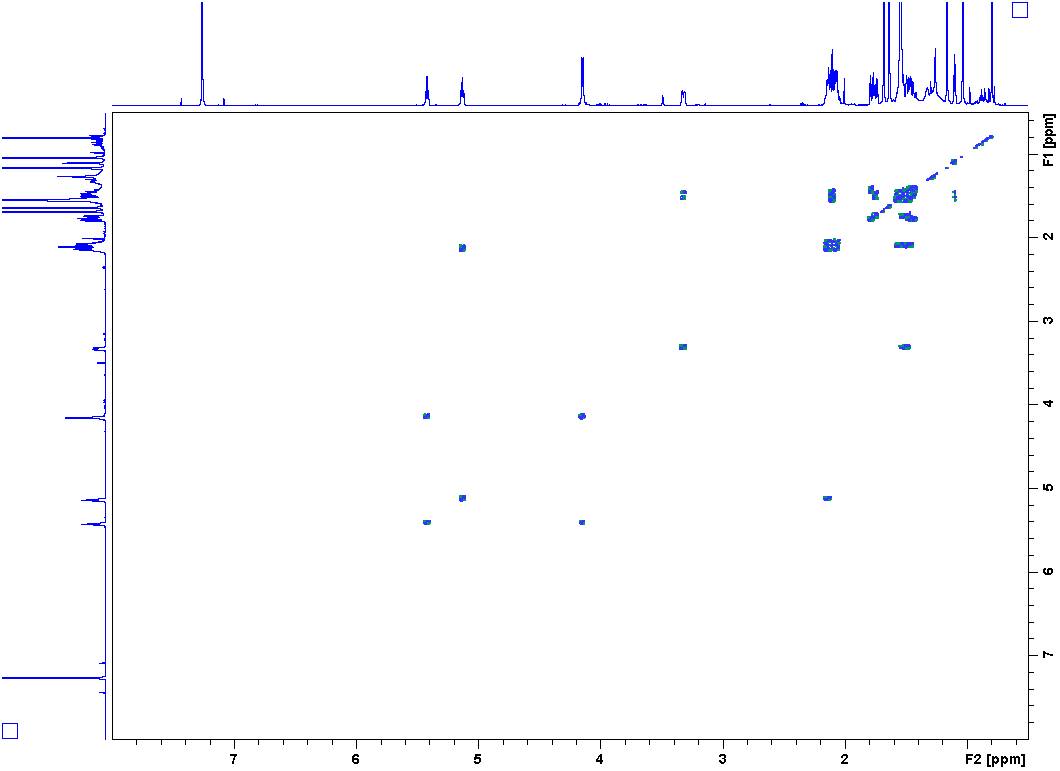

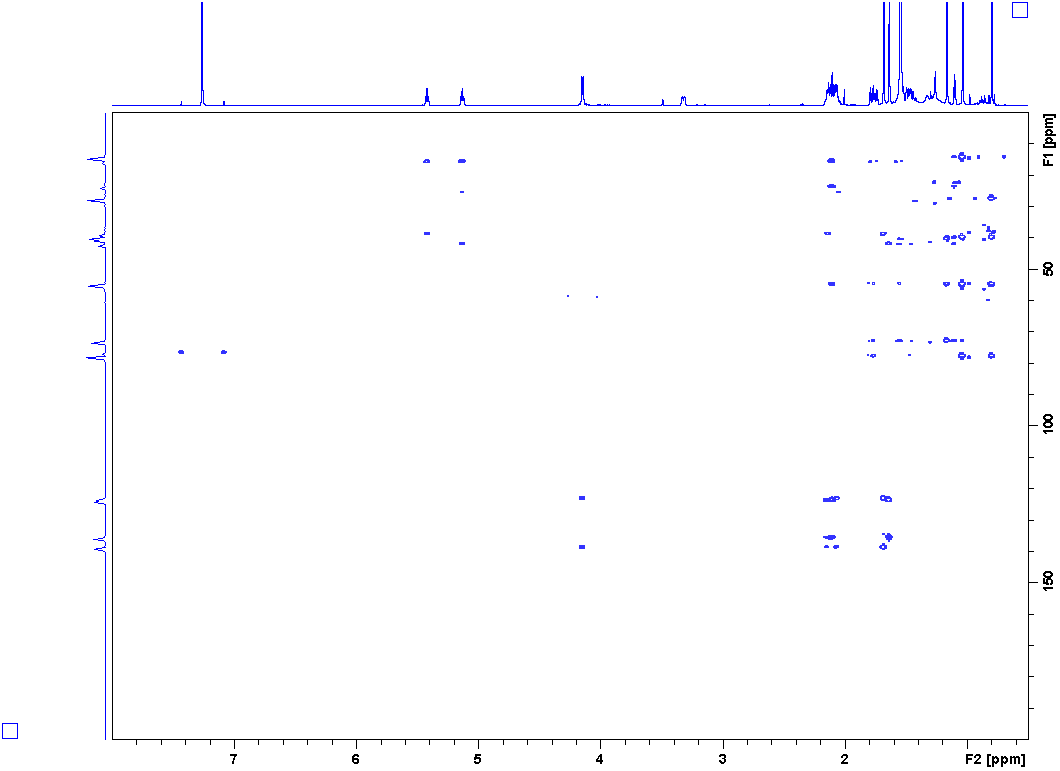


**b**

**a**

**Supplementary figure 49 NMR analysis of trimethylcyclohexane-11,14-diolgeraniol (22):** (**a**) COSY, and (**b**) HMBC spectra of **22**. Couplings; see supplementary table 18. Ref.: This work


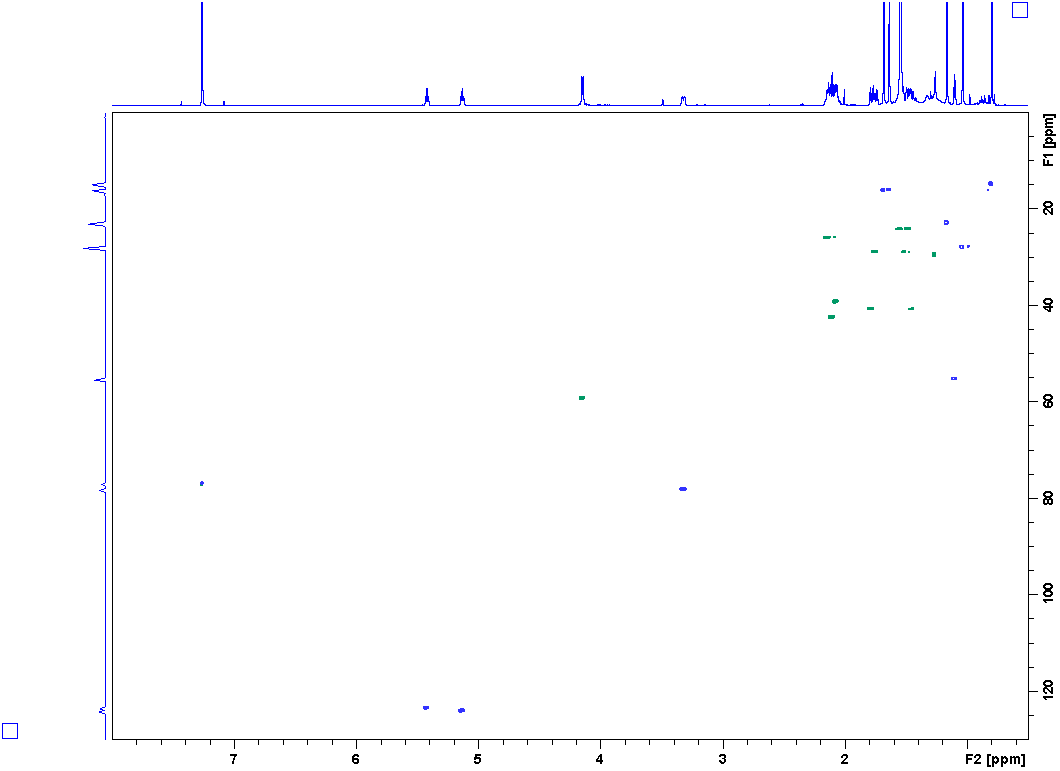

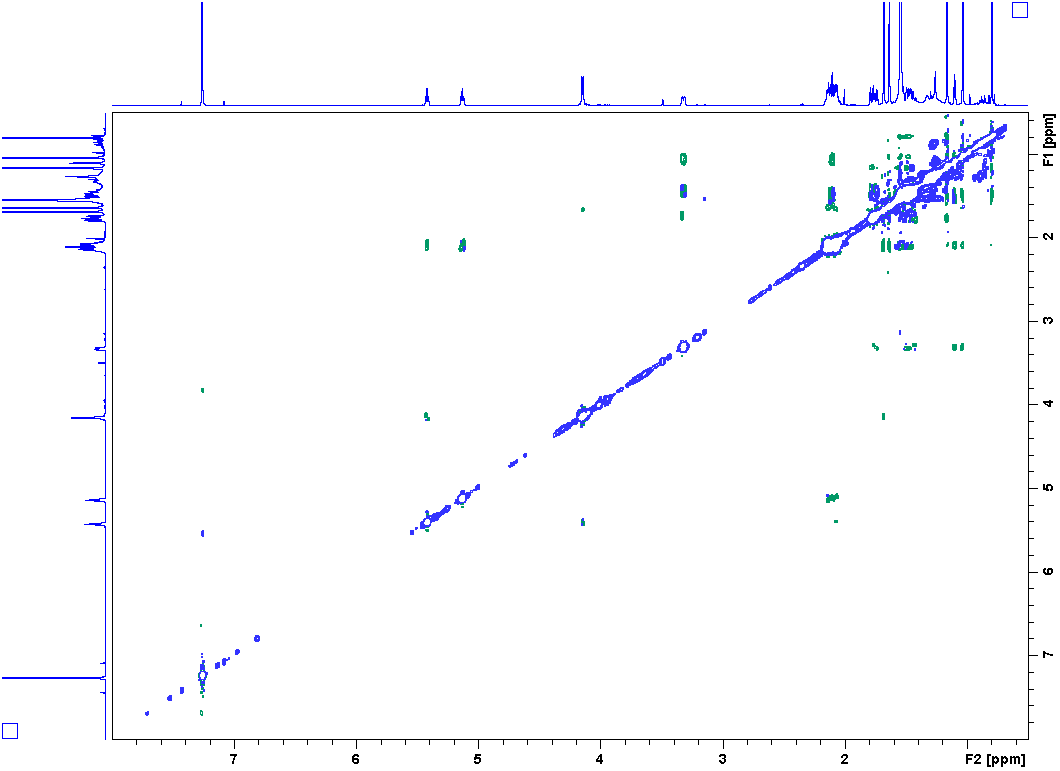


**b**

**a**

**Supplementary figure 50 NMR analysis of trimethylcyclohexane-11,14-diolgeraniol (22):** (**a**) HSQC, and (**b**) ROESY spectra of **22**. Couplings; see supplementary table 18. Ref.: This work

**
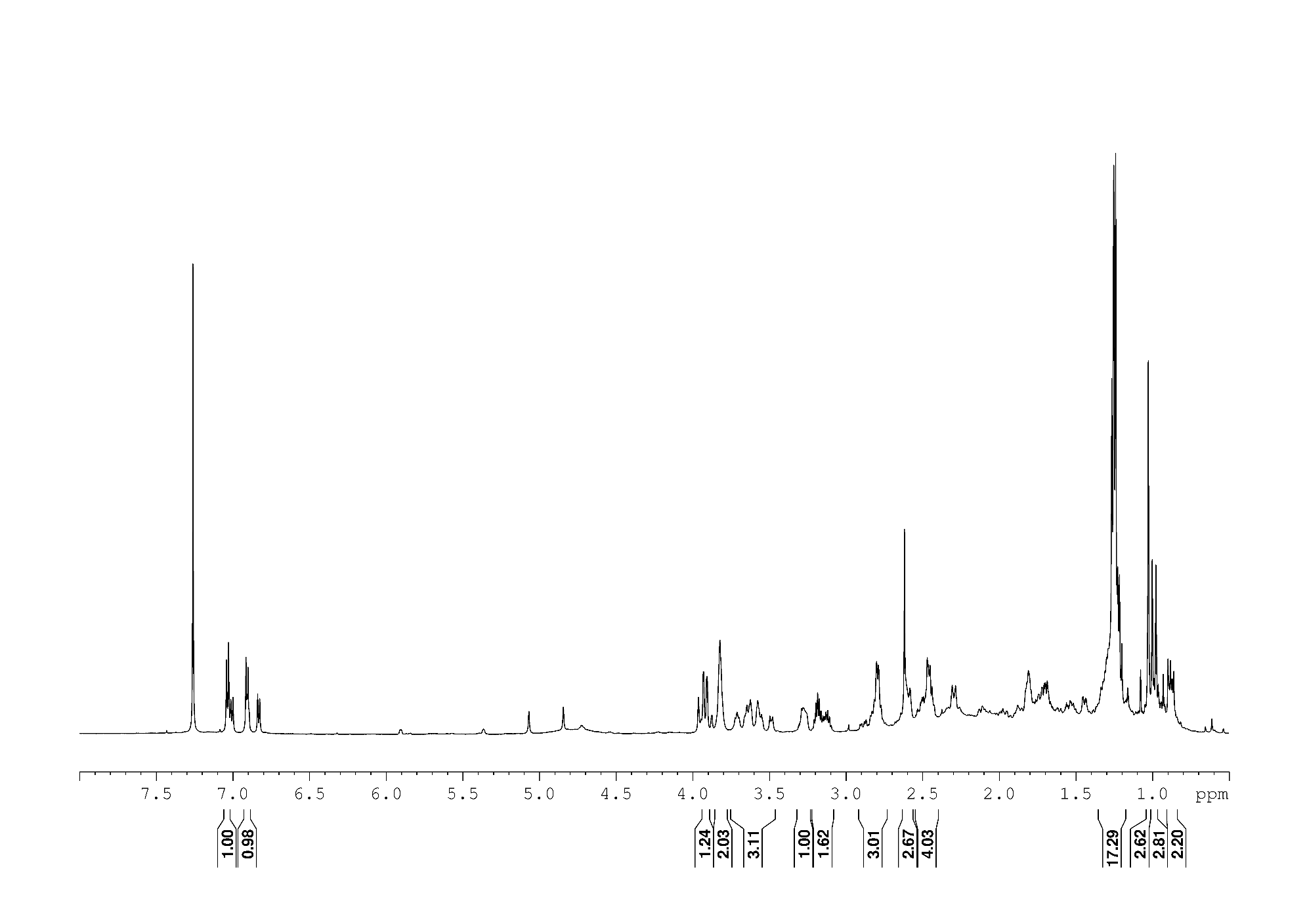
**

**Supplementary figure 51 NMR analysis of diterpenoid lactam (23):** (**a**) ^1^H NMR spectrum (600.13 MHz, CDCl_3_) spectra of **23**. Couplings; see supplementary table 19 Ref.: This work


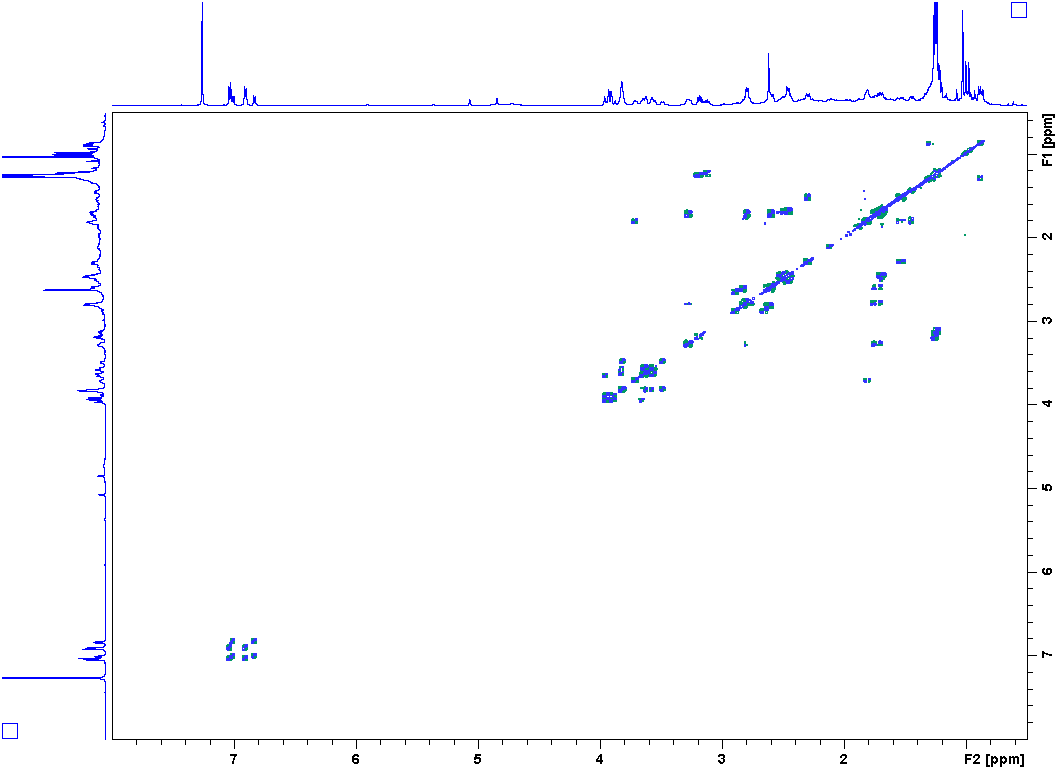


**a**


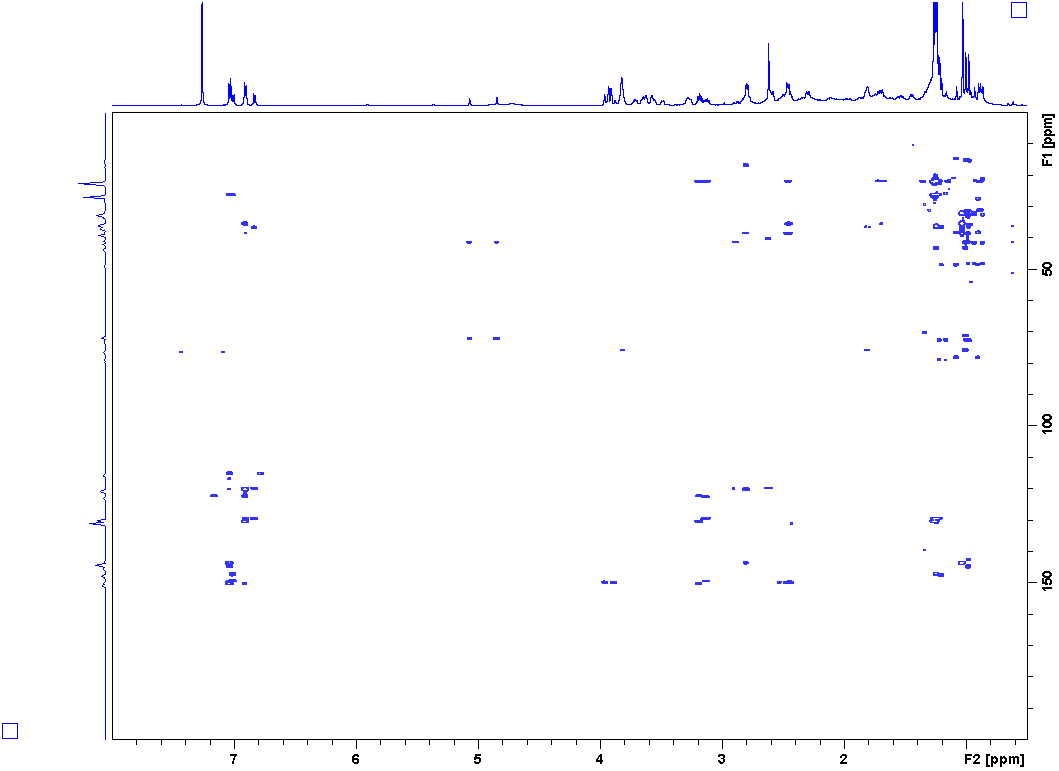


**b**

**Supplementary figure 52 NMR analysis of diterpenoid lactam (23):** (**a**) COSY, and (**b**) HMBC spectra of **23**. Couplings; see supplementary table 19 Ref.: This work


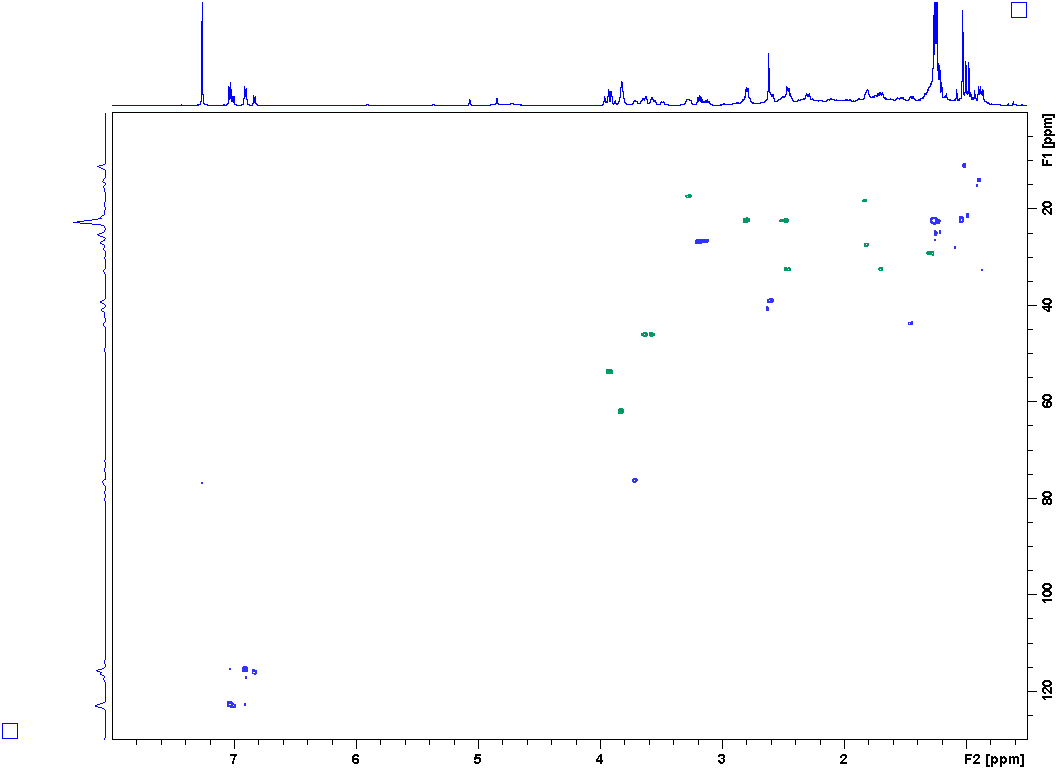


**a**


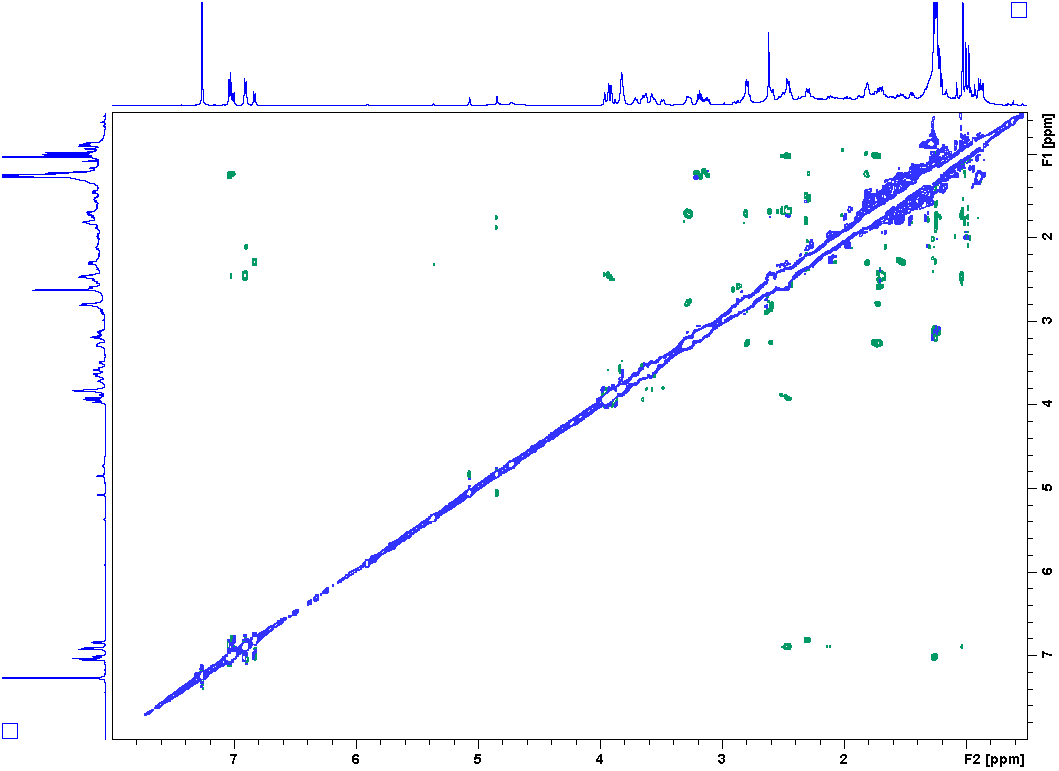


**b**

**Supplementary figure 53 NMR analysis of diterpenoid lactam (23):** (**a**) HSQC, and (**b**) ROESY spectra of **23**. Couplings; see supplementary table 19 Ref.: This work

**Supplementary table 1** List of compounds identified in *S. cerevisiae* and *N. benthamiana* extracts.

| # | Compound name | Identification method | Reference | Supplementary material # |
| --- | --- | --- | --- | --- |
| (1) | Triptolide | Auth. Standard | 12 |  |
| (2) | Triptonide | Auth. Standard | 12 |  |
| (3) | miltiradiene | Reference GC-MS spectra | 6 |  |
| (4) | dehydroabietadiene | Reference GC-MS spectra | 12 |  |
| (5) | 14-hydroxy-dehydroabietadiene | NMR analysis | 12 | Supplementary Fig. 9-10, table 2 |
| (6) | 18(4-->3)abeo-abietatrien-14,18-diol | NMR analysis | This work | Supplementary Fig. 11-13, table 3 |
| (7) | 14-hydroxy-18(4-->3)abeo-abietatrien-18-al | NMR analysis | This work | Supplementary Fig. 14-16, table 4 |
| (8) | triptophenolide | Auth. Standard | 12 |  |
| (9) | 18R(4-->3)abeo-abietatrien-19-ene-14,18,20-triol | NMR analysis | This work | Supplementary Fig. 17-19, table 5 |
| (10) | 18R(4-->3)abeo-abietatrien-14,18-diol | NMR analysis | This work | Supplementary Fig. 20-22, table 6 |
| (11) | 18S(4-->3)abeo-abietatrien-14,18-diol | NMR analysis | This work | Supplementary Fig. 23-25, table 7 |
| (12) | 18S(4-->3)abeo-abietatrien-14,18,20-triol | NMR analysis | This work | Supplementary Fig. 26-28, table 8 |
| (13) | 3-epi-triptobenzene B | NMR analysis | 14 13 | Supplementary Fig. 29 table 9 |
| (14) | 4-epi-triptobenzene J | NMR analysis | This work & 15 (triptobenzene J) | Supplementary Fig. 30-32, table 10 |
| (15) | 18(4-->3)abeo-abietatrien-14,18,20-triol | NMR analysis | This work | Supplementary Fig. 33-35, table 11 |
| (16) | Triptobenzene I | NMR analysis | 16 | Supplementary Fig. 36, table 12 |
| (17) | 3β-hydroxy-11,14-oxo-abieta-8,12-diene | NMR analysis | 21 | Supplementary Fig. 37, table 13 |
| (18) | 4-epi-triptoquinone C | NMR analysis | This work & 18 (triptoquinone C) | Supplementary Fig. 38-40, table 14 |
| (19) | (+)isocopal-13(16)-en-3,14-diol | NMR analysis | This work | Supplementary Fig. 41-43, table 15 |
| (20) | labda-8(17)-13(14)en-15-O-acetate-3-ol | NMR analysis | This work | Supplementary Fig. 44-46, table 16 |
| (21) | 14,15-Epoxygeranylgeraniol | NMR analysis | 19,20 | Supplementary Fig. 47, table 17 |
| (22) | trimethylcyclohexane-11,14-diolgeraniol | NMR analysis | This work | Supplementary Fig. 48-50, table 18 |
| (23) | lactam-diterpenoid | NMR analysis | This work | Supplementary Fig. 51-53, table 19 |

**Supplementary table 2** ^1^H and ^13^C NMR data and 2D HMBC and ROESY correlations for **5**

| Pos. | *δ*_C_, type*^a,b^* | *δ*_H_, nH, multiplicity (*J* in Hz)*^a,c^* | HMBC | ROESY |
| --- | --- | --- | --- | --- |
| 1 | 39.1, CH_2_ | α: 1.39 (1H, td, 12.9, 3.5) | C-2, C-3, C-9, C-10, C-20 | H-1β |
|  |  | β: 2.28 (1H, br d, 12.9) | C-2, C-3, C-5, C-10, C-20 | H-1α, H-2β, H-2β, H-11, H-20 |
| 2 | 19.5, CH_2_ | α: 1.75 (1H, m) | C-1, C-3, C-4 | H-5 |
|  |  | β: 1.61 (1H, m) | C-1, C-3, C-4, C-10 | H-1β, H-20 |
| 3 | 41.8, CH_2_ | α: 1.22 (1H, m) | C-1, C-2, C-4/18, C-19 | H-18 |
|  |  | β: 1.49 (1H, dtd, 13.2, 3.2, 1.5) | C-1, C-2, C-4/18, C-5, C-19 | H-19 |
| 4 | 33.5, C | - |  |  |
| 5 | 49.9, CH | 1.34 (1H, dd, 12.7, 2.1) | C-4/18, C-6, C-7, C-9, C-10, C-19, C-20 | H-2α, H-6α, H-7α, H-18 |
| 6 | 18.6, CH_2_ | α: 1.99 (1H, br dd, 13.1, 7.9) | C-4, C-5, C-7, C-8, C-10 | H-5, H-7α, H-7β, H-18 |
|  |  | β: 1.72 (1H, m) | C-5, C-7, C-10 | H-7β, H-19, H-20 |
| 7 | 24.5, CH_2_ | α: 2.62 (1H, ddd, 16.5, 11.4, 7.9) | C-6, C-8, C-9, C-14 | H-5, H-6α, 14-OH |
|  |  | β: 2.82 (1H, dd, 16.5, 6.7) | C-5, C-6, C-8, C-9, C-14 | H-6α, H-6β, 14-OH |
| 8 | 120.8, C | - |  |  |
| 9 | 149.2, C | - |  |  |
| 10 | 37.7, C | - |  |  |
| 11 | 116.5, CH | 6.87 (1H, d, 8.2) | C-8, C-10, C-12, C-13, C-14 | H-1β, H-20 |
| 12 | 123.4, CH | 7.02 (1H, d, 8.2) | C-8, C-9, C-11, C-14, C-15 | H-16, H-17 |
| 13 | 130.1, C | - |  |  |
| 14 | 150.4, C | - |  |  |
| 14-OH | - | 4.63 (1H, s) | C-8, C-13, C-14 | H-7α, H-7β, H-15, H-16/17 |
| 15 | 27.0, CH | 3.16 (1H, sep, 6.9) | C-12, C-13, C-14, C-16, C-17 | 14-OH |
| 16 | 22.9, CH_3_ | 1.24 (3H, d, 6.9) | C-13, C-15, C-17 | H-12, 14-OH |
| 17 | 22.7, CH_3_ | 1.26 (3H, d, 6.9) | C-13, C-15, C-16 | H-12, 14-OH |
| 18 | 33.4, CH_3_ | 0.97 (3H, s) | C-3, C-4, C-5, C-19 | H-3α, H-5, H-6α |
| 19 | 21.8, CH_3_ | 0.94 (3H, s) | C-3, C-4, C-5, C-18 | H-3β, H-6β, H-20 |
| 20 | 25.0, CH_3_ | 1.20 (3H, s) | C-1, C-5, C-9, C-10 | H-1β, H-2β, H-6β, H-11, H-19 |

*^a^* ^1^H NMR (600.13) and ^13^C NMR (150.90 MHz) data obtained with samples in CDCl_3_. *^b^* Assignments based on HSQC and HMBC experiments.
*^c^* Multiplicities reported as apparent splittings: s = singlet, d = doublet, t = triplet, sep = septet, m = multiplet (incl. overlapping resonances), br = broad. α denotes hydrogen pointing into the plane and β denotes hydrogen pointing out of the plane.

**Supplementary table 3** ^1^H and ^13^C NMR data and 2D HMBC and ROESY correlations for **6**

| Pos. | *δ*_C_, type*^a,b^* | *δ*_H_, nH, multiplicity (*J* in Hz)*^a,c^* | HMBC | ROESY |
| --- | --- | --- | --- | --- |
| 1 | 33.4, CH_2_ | α: 1.60 (1H, m) |  | H-1β, H-5 |
|  |  | β: 2.35 (1H, m) | C-5, C-10 | H-1α, H-11, H-20 |
| 2 | 26.0, CH_2_ | 2.35 (2H, m) | C-3, C-4 | H-18A, H-18B |
| 3 | 131.8, C | - |  |  |
| 4 | 129.7, C | - |  |  |
| 5 | 44.5, CH | 2.27 (1H, m) |  | H-1α |
| 6 | 20.0, CH_2_ | α: 2.28 (1H, m) | C-8, C-10 | H-7A, H-7B, H-19 |
|  |  | β: 1.64 (1H, m) | (C-5) | H-7B, H-20 |
| 7 | 23.3, CH_2_ | A: 2.71 (1H, ddd, 16.8, 10.9, 8.4) | C-6, C-8 | H-6α, 14-OH |
|  |  | B: 2.84 (1H, dd, 16.8, 7.1) | C-5, C-6, C-8, C-9, C-14 | H-6α, H-6β, 14-OH |
| 8 | 120.6, C | - |  |  |
| 9 | 146.0, C | - |  |  |
| 10 | 35.6, C | - |  |  |
| 11 | 116.2, CH | 6.93 (1H, d, 8.1) | C-8, C-10, C-13 | H-1β |
| 12 | 123.1, CH | 7.03 (1H, d, 8.1) | C-9, C-14, C-15 | H-16, H-17 |
| 13 | 130.3, C | - |  |  |
| 14 | 150.4, C | - |  |  |
| 14-OH | - | 4.65 (1H, br s) |  | H-7α, H-7β, H-15 |
| 15 | 26.9, CH | 3.14 (1H, sep, 6.9) | C-12, C-13, C-14, C-16, C-17 | 14-OH |
| 16 | 22.6, CH_3_ | 1.25 (3H, d, 6.9) | C-13, C-15, C-17 | H-12 |
| 17 | 22.6, CH_3_ | 1.27 (3H, d, 6.9) | C-13, C-15, C-16 | H-12 |
| 18 | 63.4, CH_2_ | A: 4.09 (1H, d, 11.6) | C-2, C-3, C-4 | H-2, H-19 |
|  |  | B: 4.26 (1H, d, 11.6) | C-2, C-3, C-4 | H-2, H-19 |
| 19 | 15.6, CH_3_ | 1.78 (3H, br s) | C-3, C-4, C-5 | H-6α, H-18A, H-18B |
| 20 | 22.4, CH_3_ | 1.02 (3H, s) | C-1, C-5, C-9, C-10 | H-1β, H-6β |

*^a^* ^1^H NMR (600.13) and ^13^C NMR (150.90 MHz) data obtained with samples in CDCl_3_. *^b^* Assignments based on HSQC and HMBC experiments.
*^c^* Multiplicities reported as apparent splittings: s = singlet, d = doublet, t = triplet, sep = septet, m = multiplet (incl. overlapping resonances), br = broad. α denotes hydrogen pointing into the plane and β denotes hydrogen pointing out of the plane. A denotes the lowest chemical shift value and B denotes the highest chemical shift value.

**Supplementary table 4** ^1^H and ^13^C NMR data and 2D HMBC and ROESY correlations for **7**

| Pos. | *δ*_C_, type*^a,b^* | *δ*_H_, nH, multiplicity (*J* in Hz)*^a,c^* | HMBC | ROESY |
| --- | --- | --- | --- | --- |
| 1 | 32.7, CH_2_ | α: 1.56 (1H, m) | C-2, C-5, C-10, C-20 | H-2α |
|  |  | β: 2.42 (1H, m) | C-2, C-3, C-5, C-10, C-20 | H-11, H-20 |
| 2 | 20.7, CH_2_ | α: 2.53 (1H, m) | C-1, C-3, C-4, C-10 | H-1α, H-19 |
|  |  | β: 2.33 (1H, m) |  | H-20 |
| 3 | 133.5, C | - |  |  |
| 4 | 156.1, C | - |  |  |
| 5 | 46.7, CH | 2.44 (1H, m) |  | H-7α |
| 6 | 19.4, CH_2_ | α: 2.38 (1H, m) | C-5, C-8, C-10 | H-7α |
|  |  | β: 1.76 (1H, tdd, 13.2, 10.9, 7.2) | C-5, C-7, C-10 | H-7β, H-20 |
| 7 | 23.4, CH_2_ | α: 2.77 (1H, ddd, 17.0, 10.9, 8.2) | C-6, C-8, C-9, C-14 | H-5, H-6α, 14-OH |
|  |  | β: 2.93 (1H, dd, 17.0, 7.2) | C-5, C-6, C-8, C-9, C-14 | H-6β, 14-OH |
| 8 | 120.6, C | - |  |  |
| 9 | 145.1, C | - |  |  |
| 10 | 35.8, C | - |  |  |
| 11 | 116.4, CH | 6.94 (1H, d, 8.2) | C-8, C-10, C-13 | H-1β |
| 12 | 123.3, CH | 7.05 (1H, d, 8.2) | C-9, C-14, C-15 | H-16, H-17 |
| 13 | 130.6, C | - |  |  |
| 14 | 150.4, C | - |  |  |
| 14-OH | - | 4.68 (1H, s) | C-8, C-13, C-14 | H-7α, H-7β, H-15 |
| 15 | 26.9, CH | 3.12 (1H, sep, 6.9) | C-12, C-13, C-14, C-16, C-17 | 14-OH |
| 16 | 22.6, CH_3_ | 1.26 (3H, d, 6.9) | C-13, C-15, C-17 | H-12 |
| 17 | 22.5, CH_3_ | 1.27 (3H, d, 6.9) | C-13, C-15, C-16 | H-12 |
| 18 | 191.3, CH | 10.24 (1H, s) | C-2, C-3 | H-19 |
| 19 | 15.0, CH_3_ | 2.22 (3H, ddd, 1.8, 1.6, 1) | C-3, C-4, C-5 | H-2α, H-18 |
| 20 | 22.7, CH_3_ | 1.02 (3H, s) | C-1, C-5, C-9, C-10 | H-1β, H-2β, H-6β |

*^a^* ^1^H NMR (600.13) and ^13^C NMR (150.90 MHz) data obtained with samples in CDCl_3_. *^b^* Assignments based on HSQC and HMBC experiments.
*^c^* Multiplicities reported as apparent splittings: s = singlet, d = doublet, t = triplet, sep = septet, m = multiplet (incl. overlapping resonances), br = broad. α denotes hydrogen pointing into the plane and β denotes hydrogen pointing out of the plane. A denotes the lowest chemical shift value and B denotes the highest chemical shift value.

**Supplementary table 5** ^1^H and ^13^C NMR data and 2D HMBC and ROESY correlations for **9**

| Pos. | *δ*_C_, type*^a,b^* | *δ*_H_, nH, multiplicity (*J* in Hz)*^a,c^* | HMBC | ROESY |
| --- | --- | --- | --- | --- |
| 1 | 34.8, CH_2_ | α: 1.87 (1H, td, 12.7, 5.2) | C-2, C-10, C-20 | H-5, H-18 |
|  |  | β: 2.10 (1H, dt, 12.7, 3.6) | C-2, C-3, C-5 | H-2, H-11, H-20 |
| 2 | 32.4, CH_2_ | 1.80 (2H, m) | C-1, C-3, C-4, C-10 | H-1β, H-5, H-18, H-20 |
| 3 | 73.4, C | - |  |  |
| 4 | 152.5, C | - |  |  |
| 5 | 43.4, CH | 2.76 (1H, br d, 12.3) | C-4, C-6, C-10, C-19 | H-1α, H-2, H-7α |
| 6 | 21.7, CH_2_ | α: 1.84 (1H, m) | C-5, C-7, C-8, C-10 | H-7α, H-7β, H-19A |
|  |  | β: 1.71 (1H, m) | C-5, C-7, C-10 | H-7β, H-19A, H-20 |
| 7 | 25.2, CH_2_ | α: 2.60 (1H, m) | C-6, C-8, C-9, C-14 | H-5, H-6α, H-6β, H-7β |
|  |  | β: 2.94 (1H, dd, 17.2, 5.8) | C-5, C-6, C-8, C-9, C-14 | H-6α, H-6β, H-7α |
| 8 | 123.5, C | - |  |  |
| 9 | 146.4, C | - |  |  |
| 10 | 39.8, C | - |  |  |
| 11 | 118.2, CH | 6.85 (1H, d, 8.2) | C-8, C-10, C-13 | H-1β |
| 12 | 123.6, CH | 6.96 (1H, d, 8.2) | C-9, C-14, C-15 | H-16/17 |
| 13 | 132.9, C | - |  |  |
| 14 | 151.8, C | - |  |  |
| 15 | 27.3, CH | 3.27 (1H, sep, 6.9) | C-12, C-13, C-14, C-16/17 |  |
| 16 | 23.0, CH_3_ | 1.19 (3H, d, 6.9) | C-13, C-15, C-17 | H-12 |
| 17 | 23.0, CH_3_ | 1.19 (3H, d, 6.9) | C-13, C-15, C-16 | H-12 |
| 18 | 68.6, CH_2_ | 3.71 (2H, s) | C-2, C-3, C-4 | H-1α, H-2, H-19B |
| 19 | 107.5, CH_2_ | A: 4.83 (1H, br s) | C-3, C-4, C-5 | H-6α, H-6β |
|  |  | B: 5.10 (1H, br s) | C-3, C-4, C-5 | H-18 |
| 20 | 21.8, CH_3_ | 0.96 (3H, s) | C-1, C-5, C-9, C-10 | H-1β, H-2, H-6β |

*^a^* ^1^H NMR (600.13) and ^13^C NMR (150.90 MHz) data obtained with samples in methanol-*d*_4_. *^b^* Assignments based on HSQC and HMBC experiments.
*^c^* Multiplicities reported as apparent splittings: s = singlet, d = doublet, t = triplet, sep = septet, m = multiplet (incl. overlapping resonances), br = broad. α denotes hydrogen pointing into the plane and β denotes hydrogen pointing out of the plane. A denotes the lowest chemical shift value and B denotes the highest chemical shift value.

**Supplementary table 6** ^1^H and ^13^C NMR data and 2D HMBC and ROESY correlations for **10**

| Pos. | *δ*_C_, type*^a,b^* | *δ*_H_, nH, multiplicity (*J* in Hz)*^a,c^* | HMBC | ROESY |
| --- | --- | --- | --- | --- |
| 1 | 33.3, CH_2_ | α: 1.49 (1H, m) | C-10, C-20 | H1β, H-18 |
|  |  | β: 2.04 (1H, m) | C-3, C-5 | H1α, H-11, H-20 |
| 2 | 19.5, CH_2_ | α: 1.66 (1H, m) |  | H-18 |
|  |  | β: 2.00 (1H, m) |  | H-19, H-20 |
| 3 | 42.9, CH | 1.72 (1H, m) | C-1, C-2, C-4, C-18, C-19 |  |
| 4 | 34.7, CH | 1.91 (1H, m) | C-2, C-3, C-5, C-10, C-18, C-19 | H-6α, H-18 |
| 5 | 38.7, CH | 1.74 (1H, ddd, 12.8, 5.1, 2.2) | C-4, C-6, C-7, C-10, C-19 |  |
| 6 | 23.4, CH_2_ | α: 1.59 (1H, br dd, 12.8, 7.8) | C-4, C-5, C-7, C-8, C-10 | H-4, H-7α, H-7β |
|  |  | β: 1.98 (1H, m) | C-5, C-7, C-10 | H-7β, 14-OH, H-19, H-20 |
| 7 | 24.0, CH_2_ | α: 2.62 (1H, ddd, 16.5, 11.4, 7.8) | C-6, C-8, C-9 | H-6α, 14-OH |
|  |  | β: 2.81 (1H, dd, 16.5, 6.7) | C-5, C-6, C-8, C-9, C-14 | H-6α, H-6β |
| 8 | 120.7, C | - |  |  |
| 9 | 148.2, C | - |  |  |
| 10 | 37.4, C | - |  |  |
| 11 | 116.4, CH | 6.82 (1H, d, 8.2) | C-8, C-10, C-13 | H-1β |
| 12 | 123.3, CH | 7.01 (1H, d, 8.2) | C-9, C-14, C-15 | H-16, H-17 |
| 13 | 130.0, C | - |  |  |
| 14 | 150.2, C | - |  |  |
| 14-OH | - | 4.63 (1H, br s) | H-8, H-13 | H-7α, H-7β, H-15 |
| 15 | 26.8, CH | 3.14 (1H, sep, 6.9) | C-12, C-13, C-14, C-16, C-17 | 14-OH |
| 16 | 22.7, CH_3_ | 1.24 (3H, d, 6.9) | C-13, C-15, C-17 | H-12 |
| 17 | 22.6, CH_3_ | 1.25 (3H, d, 6.9) | C-13, C-15, C-16 | H-12 |
| 18 | 64.7, CH_2_ | 3.66 (2H, m) | C-2, C-4 | H-1α, H-2α, H-4 |
| 19 | 16.7, CH_3_ | 1.09 (3H, d, 7.6) | C-3, C-4, C-5 | H-2β, H-6β, H-20 |
| 20 | 25.0, CH_3_ | 1.21 (3H, s) | C-1, C-5, C-9, C-10 | H-1β, H-2β, H-6β, H-19 |

*^a^* ^1^H NMR (600.13) and ^13^C NMR (150.90 MHz) data obtained with samples in CDCl_3_. *^b^* Assignments based on HSQC and HMBC experiments.
*^c^* Multiplicities reported as apparent splittings: s = singlet, d = doublet, t = triplet, sep = septet, m = multiplet (incl. overlapping resonances), br = broad. α denotes hydrogen pointing into the plane and β denotes hydrogen pointing out of the plane. A denotes the lowest chemical shift value and B denotes the highest chemical shift value.

**Supplementary table 7** ^1^H and ^13^C NMR data and 2D HMBC and ROESY correlations for **11**

| Pos. | *δ*_C_, type*^a,b^* | *δ*_H_, nH, multiplicity (*J* in Hz)*^a,c^* | HMBC | ROESY |
| --- | --- | --- | --- | --- |
| 1 | 38.5, CH_2_ | α: 1.46 (1H, m) | C-2, C-10, C-20 | H1β, H-3 |
|  |  | β: 2.30 (1H, m) | C-2, C-3, C-5, C-10 | H1α, H-2β, H-11, H-20 |
| 2 | 20.7, CH_2_ | α: 1.60 (1H, m) |  | H-18A, H-20 |
|  |  | β: 1.47 (1H, m) | C-1, C-3 | H1β, H-18B, H-19 |
| 3 | 44.4, CH | 1.76 (1H, m) |  | H1α, H-4, H-18A, H-18B |
| 4 | 35.0, CH | 2.07 (1H, m) | C-2, C-3, C-5, C-10, C-19 | H-3, H-5, H-6α, H-18B |
| 5 | 44.7, CH | 1.68 (1H, ddd, 12.8, 4.4, 2.1) | C-4, C-6, C-7, C-10, C-19, C-20 | H-4, H-7α |
| 6 | 23.7, CH_2_ | α: 1.65 (1H, br dd, 12.8, 8.2) | C-4, C-5, C-7, C-8, C-10 | H-4, H-7α, H-7β |
|  |  | β: 2.03 (1H, m) | C-5, C-7, C-10 | H-7β, 14-OH, H-19, H-20 |
| 7 | 24.1, CH_2_ | α: 2.65 (1H, ddd, 16.4, 11.5, 7.8) | C-6, C-8, C-9, C-14 | H-5, H-6α, 14-OH |
|  |  | β: 2.82 (1H, dd, 16.4, 6.6) | C-5, C-6, C-8, C-9, C-14 | H-6α, H-6β |
| 8 | 120.7, C | - |  |  |
| 9 | 148.2, C | - |  |  |
| 10 | 37.5, C | - |  |  |
| 11 | 116.6, CH | 6.84 (1H, d, 8.2) | C-8, C-10, C-13 | H-1β, H-20 |
| 12 | 123.4, CH | 7.02 (1H, d, 8.2) | C-9, C-14, C-15 | H-16, H-17 |
| 13 | 130.0, C | - |  |  |
| 14 | 150.2, C | - |  |  |
| 14-OH | - | 4.64 (1H, br s) | H-8, H-13, H-14 | H-7α, H-7β, H-15 |
| 15 | 26.9, CH | 3.14 (1H, sep, 6.9) | C-12, C-13, C-14, C-16, C-17 | 14-OH |
| 16 | 22.7, CH_3_ | 1.24 (3H, d, 6.9) | C-13, C-15, C-17 | H-12 |
| 17 | 22.6, CH_3_ | 1.25 (3H, d, 6.9) | C-13, C-15, C-16 | H-12 |
| 18 | 65.8, CH_2_ | A: 3.54 (1H, dd, 10.4, 6.8) | C-3, C-4, C-5 | H-2α, H-2β, H-3, H-19 |
|  |  | B: 3.60 (1H, dd, 10.4, 8.0) | C-3, C-4, C-5 | H-2β, H-3, H-4, H-19 |
| 19 | 9.7, CH_3_ | 0.89 (3H, d, 7.6) | C-3, C-4, C-5 | H-2β, H-6β, H-18A, H-18B, H-20 |
| 20 | 25.4, CH_3_ | 1.16 (3H, s) | C-1, C-5, C-9, C-10 | H-1β, H-2α, H-6β, H-11, H-19 |

*^a^* ^1^H NMR (600.13) and ^13^C NMR (150.90 MHz) data obtained with samples in CDCl_3_. *^b^* Assignments based on HSQC and HMBC experiments.
*^c^* Multiplicities reported as apparent splittings: s = singlet, d = doublet, t = triplet, sep = septet, m = multiplet (incl. overlapping resonances), br = broad. α denotes hydrogen pointing into the plane and β denotes hydrogen pointing out of the plane. A denotes the lowest chemical shift value and B denotes the highest chemical shift value.

**Supplementary table 8** ^1^H and ^13^C NMR data and 2D HMBC and ROESY correlations for **12**

| Pos. | *δ*_C_, type*^a,b^* | *δ*_H_, nH, multiplicity (*J* in Hz)*^a,c^* | HMBC | ROESY |
| --- | --- | --- | --- | --- |
| 1 | 38.4, CH_2_ | α: 1.45 (1H, m) | C-2, C-20 | H1β, H-3 |
|  |  | β: 2.28 (1H, m) | C-3, C-5, C-10, C-20 | H1α, H-11, H-20 |
| 2 | 21.3, CH_2_ | 1.46 (1H, m) | C-1, C-3, C-4, C-10 | H-18A, H-18B, H-19B, H-20 |
| 3 | 42.8, CH | 1.89 (1H, m) |  | H1α, H-4, H-18B |
| 4 | 44.6, CH | 2.21 (1H, dt, 9.0, 4.6) |  | H-3, H-5, H-6α, H-19A |
| 5 | 44.4, CH | 1.79 (1H, m) | C-6, C-7, C-19 | H-4, H-7α |
| 6 | 23.8, CH_2_ | α: 1.80 (1H, m) | C-4, C-5, C-7, C-8, C-10 | H-4, H-7α, H-7β, H-19A |
|  |  | β: 1.98 (1H, tdd, 13.3, 11.6, 6.2) | C-5, C-7, C-10 | H-7β, 14-OH, H-19A, H-20 |
| 7 | 24.4, CH_2_ | α: 2.66 (1H, ddd, 16.4, 11.6, 7.4) | C-6, C-8 | H-5, H-6α, 14-OH |
|  |  | β: 2.85 (1H, dd, 16.4, 6.2) | C-5, C-6, C-8, C-9, C-14 | H-6α, H-6β |
| 8 | 120.5, C | - |  |  |
| 9 | 146.9, C | - |  |  |
| 10 | 37.1, C | - |  |  |
| 11 | 116.9, CH | 6.83 (1H, d, 8.3) | C-8, C-10, C-13 | H-1β |
| 12 | 123.4, CH | 7.02 (1H, d, 8.3) | C-9, C-14, C-15 | H-16, H-17 |
| 13 | 130.1, C | - |  |  |
| 14 | 150.2, C | - |  |  |
| 14-OH | - | 4.64 (1H, s) | H-8, H-13, H-14 | H-7α, H-7β, H-15 |
| 15 | 26.8, CH | 3.13 (1H, sep, 6.9) | C-12, C-13, C-14, C-16, C-17 | 14-OH |
| 16 | 22.7, CH_3_ | 1.24 (3H, d, 6.9) | C-13, C-15, C-17 | H-12 |
| 17 | 22.5, CH_3_ | 1.25 (3H, d, 6.9) | C-13, C-15, C-16 | H-12 |
| 18 | 65.4, CH_2_ | A: 3.63 (1H, m) |  | H-2, H-19B |
|  |  | B: 3.65 (1H, m) | C-3, C-4 | H-2, H-3, H-19B |
| 19 | 59.5, CH_2_ | A: 3.71 (1H, d, 10.4) | C-3, C-4 | H-4, H-6α, H-6β, H-20 |
|  |  | B: 3.88 (1H, dd, 10.4, 9.2) | C-3, C-4 | H-2, H-18A/B, H-20 |
| 20 | 24.6, CH_3_ | 0.98 (3H, s) | C-1, C-5, C-9, C-10 | H-1β, H-2, H-6β, H-19A, H-19B |

*^a^* ^1^H NMR (600.13) and ^13^C NMR (150.90 MHz) data obtained with samples in CDCl_3_. *^b^* Assignments based on HSQC and HMBC experiments.
*^c^* Multiplicities reported as apparent splittings: s = singlet, d = doublet, t = triplet, sep = septet, m = multiplet (incl. overlapping resonances), br = broad. α denotes hydrogen pointing into the plane and β denotes hydrogen pointing out of the plane. A denotes the lowest chemical shift value and B denotes the highest chemical shift value.

**Supplementary table 9** ^1^H and ^13^C NMR data and 2D HMBC and ROESY correlations for **13**

| Pos. | *δ*_C_, type*^a,b^* | *δ*_H_, nH, multiplicity (*J* in Hz)*^a,c^* | HMBC | ROESY |
| --- | --- | --- | --- | --- |
| 1 | 37.1, CH_2_ | α: 1.55 (1H, td, 13.2, 4.2) | C-2, C-3, C-9, C-10, C-20 | H-3 |
|  |  | β: 2.31 (1H, dt, 13.2, 3.5) | C-2, C-3, C-5, C-10, C-20 | H-2, H-11, H-20 |
| 2 | 27.9, CH_2_ | 1.80 (2H, m) | C-1, C-3, C-4, C-10 | H-1β |
| 3 | 78.7, CH | 3.31 (1H, dd, 11.5, 4.7) | C-2, C-4, C-18, C-19 | H-1α, H-5, H-18 |
| 4 | 38.9, C | - |  |  |
| 5 | 49.2, CH | 1.32 (1H, dd, 12.5, 2.0) | C-3, C-4, C-6, C-7, C-9, C-10, C-18, C-19, C-20 | H-1α, H-3, H-7α, H-18 |
| 6 | 18.2, CH_2_ | α: 2.00 (1H, ddt, 13.3, 7.9, 2.0) | C-4, C-5, C-7, C-8, C-10 | H-7α, H-7β, H-18, H-19 |
|  |  | β: 1.77 (1H, m) | C-5, C-7, C-10 | H-7β, H-19 |
| 7 | 24.6, CH_2_ | α: 2.62 (1H, ddd, 16.7, 11.6, 7.9) | C-6, C-8, C-9, C-14 | H-5, H-6α |
|  |  | β: 2.86 (1H, dd, 16.7, 6.5) | C-5, C-6, C-8, C-9, C-14 | H-6α, H-6β |
| 8 | 120.6, C | - |  |  |
| 9 | 148.2, C | - |  |  |
| 10 | 37.3, C | - |  |  |
| 11 | 116.4, CH | 6.84 (1H, d, 8.3) | C-8, C-10, C-13 | H-1β |
| 12 | 123.3, CH | 7.02 (1H, d, 8.3) | C-9, C-14, C-15 | H-16, H-17 |
| 13 | 130.2, C | - |  |  |
| 14 | 150.2, C | - |  |  |
| 15 | 26.7, CH | 3.15 (1H, sep, 6.9) | C-12, C-13, C-14, C-16, C-17 |  |
| 16 | 22.7, CH_3_ | 1.24 (3H, d, 6.9) | C-13, C-15, C-17 | H-12 |
| 17 | 22.5, CH_3_ | 1.26 (3H, d, 6.9) | C-13, C-15, C-16 | H-12 |
| 18 | 28.1, CH_3_ | 1.09 (3H, s) | C-3, C-4, C-5, C-19 | H-3, H-5, H-6α |
| 19 | 15.3, CH_3_ | 0.91 (3H, s) | C-3, C-4, C-5, C-18 | H-6α, H-6β, H-20 |
| 20 | 24.8, CH_3_ | 1.21 (3H, s) | C-1, C-5, C-9, C-10 | H-1β, H-19 |

*^a^* ^1^H NMR (600.13) and ^13^C NMR (150.90 MHz) data obtained with samples in CDCl_3_. *^b^* Assignments based on HSQC and HMBC experiments.
*^c^* Multiplicities reported as apparent splittings: s = singlet, d = doublet, t = triplet, sep = septet, m = multiplet (incl. overlapping resonances), br = broad. α denotes hydrogen pointing into the plane and β denotes hydrogen pointing out of the plane. A denotes the lowest chemical shift value and B denotes the highest chemical shift value.

**Supplementary table 10** ^1^H and ^13^C NMR data and 2D HMBC and ROESY correlations for **14**

| Pos. | *δ*_C_, type*^a,b^* | *δ*_H_, nH, multiplicity (*J* in Hz)*^a,c^* | HMBC | ROESY |
| --- | --- | --- | --- | --- |
| 1 | 36.8, CH_2_ | α: 1.49 (1H, m) | C-2, C-3, C-9, C-10, C-20 | H-1β, H-3 |
|  |  | β: 2.27 (1H, dt, 13.3, 3.1) | C-2, C-3, C-5, C-10, C-20 | H-1α, H-2, H-11, H-20 |
| 2 | 27.3, CH_2_ | 1.79 (2H, m) | C-1, C-3, C-4, C-10 | H-1β, H-19, H-20 |
| 3 | 75.9, CH | 3.69 (1H, t, 7.5) | C-2, C-4, C-18, C-19 | H-1α, H-5 |
| 4 | 42.0, C | - |  |  |
| 5 | 43.5, CH | 1.45 (1H, br d, 11.8) | C-1, C-4, C-6, C-7, C-9, C-10, C-18, C-20 | H-3, H-7A, H-18A |
| 6 | 18.2, CH_2_ | A: 1.75 (1H, m) | C-5, C-7, C-10 |  |
|  |  | B: 1.80 (1H, m) | C-8, C-10 |  |
| 7 | 24.3, CH_2_ | A: 2.62 (1H, m) | C-6, C-8, C-9, C-14 | H-5 |
|  |  | B: 2.82 (1H, dd, 16.2, 5.1) | C-5, C-6, C-8, C-9, C-14 |  |
| 8 | 120.7, C | - |  |  |
| 9 | 147.9, C | - |  |  |
| 10 | 37.1, C | - |  |  |
| 11 | 116.4, CH | 6.82 (1H, d, 8.2) | C-8, C-10, C-13 | H-1β, H-20 |
| 12 | 123.3, CH | 7.01 (1H, d, 8.2) | C-9, C-14, C-15 | H-15, H-16, H-17 |
| 13 | 130.4, C | - |  |  |
| 14 | 150.2, C | - |  |  |
| 15 | 26.7, CH | 3.14 (1H, sep, 6.9) | C-12, C-13, C-14, C-16, C-17 | H-12 |
| 16 | 22.7, CH_3_ | 1.22 (3H, d, 6.9) | C-13, C-15, C-17 | H-12 |
| 17 | 22.6, CH_3_ | 1.24 (3H, d, 6.9) | C-13, C-15, C-16 | H-12 |
| 18 | 71.0, CH_2_ | A: 3.46 (1H, d, 8.9) | C-3, C-4, C-5, C-19 | H-5 |
|  |  | B: 3.76 (1H, d, 8.9) | C-3, C-4, C-5 |  |
| 19 | 11.2, CH_3_ | 0.96 (3H, s) | C-3, C-4, C-5, C-18 | H-2, H-20 |
| 20 | 25.2, CH_3_ | 1.22 (3H, s) | C-1, C-5, C-9, C-10 | H-1β, H-2, H-11, H-19 |

*^a^* ^1^H NMR (600.13) and ^13^C NMR (150.90 MHz) data obtained with samples in CDCl_3_. *^b^* Assignments based on HSQC and HMBC experiments.
*^c^* Multiplicities reported as apparent splittings: s = singlet, d = doublet, t = triplet, sep = septet, m = multiplet (incl. overlapping resonances), br = broad. α denotes hydrogen pointing into the plane and β denotes hydrogen pointing out of the plane. A denotes the lowest chemical shift value and B denotes the highest chemical shift value.

**Supplementary table 11** ^1^H and ^13^C NMR data and 2D HMBC and ROESY correlations for **15**

| Pos. | *δ*_C_, type*^a,b^* | *δ*_H_, nH, multiplicity (*J* in Hz)*^a,c^* | HMBC | ROESY |
| --- | --- | --- | --- | --- |
| 1 | 28.5, CH_2_ | α: 1.48 (1H, m) |  | H-1β, H-5 |
|  |  | β: 2.67 (1H, ddd, 13.1, 5.2, 2.9) |  | H-1α, H-2, H-11, H-20B |
| 2 | 25.7, CH_2_ | 2.36 (2H, m) |  | H-1β, H-18A, H-18B, H-20B |
| 3 | 130.6*^d^*, C | - |  |  |
| 4 | 131.0*^d^*, C | - |  |  |
| 5 | 43.6, CH | 2.41 (1H, br d, 14.2) |  | H-1α |
| 6 | 20.1, CH_2_ | α: 2.29 (1H, dddd, 13.6, 8.4, 3.3, ~1) |  | H-7A, H-7B, H-19 |
|  |  | β: 1.70 (1H, tdd, 13.6, 10.4, 8.0) |  | H-7B, H-20A, H-20B |
| 7 | 23.0, CH_2_ | A: 2.78 (1H, m) | C-6, C-8 | H-6α, 14-OH |
|  |  | B: 2.88 (1H, m) | C-5, C-6, C-8, C-9 | H-6α, H-6β, 14-OH |
| 8 | 121.7, C | - |  |  |
| 9 | 140.8, C | - |  |  |
| 10 | 40.3, C | - |  |  |
| 11 | 117.2, CH | 6.98 (1H, d, 8.2) | C-8, C-10, C-13 | H-1β |
| 12 | 123.0, CH | 7.07 (1H, d, 8.2) | C-9, C-14, C-15 | H-16/17 |
| 13 | 131.5, C | - |  |  |
| 14 | 150.9, C | - |  |  |
| 14-OH | - | 4.72 (1H, br s) | C-8, C-13 | H-7A, H-7B, H-15 |
| 15 | 27.0, CH | 3.15 (1H, sep, 6.9) | C-12, C-13, C-14, C-16, C-17 | 14-OH |
| 16 | 22.6, CH_3_ | 1.27 (3H, d, 6.9) | C-13, C-15, C-17 | H-12 |
| 17 | 22.6, CH_3_ | 1.27 (3H, d, 6.9) | C-13, C-15, C-16 | H-12 |
| 18 | 63.2, CH_2_ | A: 4.09 (1H, d, 11.6) | C-2, C-4 | H-2, H-19 |
|  |  | B: 4.28 (1H, d, 11.6) | C-2, C-4 | H-2, H-19 |
| 19 | 15.6, CH_3_ | 1.79 (3H, q, 1.9) | C-3, C-4, C-5 | H-6α, H-18A, H-18B |
| 20 | 64.7, CH_2_ | A: 3.56 (1H, dd, 10.8, 3.0) |  | H-6β |
|  |  | B: 3.68 (1H, dd, 10.8, 7.7) | C-9 | H-1β, H-2, H-6β |
| 20-OH | - | 1.03 (1H, m) |  |  |

*^a^* ^1^H NMR (600.13) and ^13^C NMR (150.90 MHz) data obtained with samples in CDCl_3_. *^b^* Assignments based on HSQC and HMBC experiments.
*^c^* Multiplicities reported as apparent splittings: s = singlet, d = doublet, t = triplet, q = quartet, sep = septet, m = multiplet (incl. overlapping resonances), br = broad. *^d^* Assignments of C-3 and C-4 may be interchanged. α denotes hydrogen pointing into the plane and β denotes hydrogen pointing out of the plane. A denotes the lowest chemical shift value and B denotes the highest chemical shift value.

**Supplementary figure 12** ^1^H and ^13^C NMR data and 2D HMBC and ROESY correlations for **16**

| Pos. | *δ*_C_, type*^a,b^* | *δ*_H_, nH, multiplicity (*J* in Hz)*^a,c^* | HMBC | ROESY |
| --- | --- | --- | --- | --- |
| 1 | 33.5, CH_2_ | α: 1.64 (1H, ddd, 13.2, 11.5, 6.5) | C-2, C-5, C-9, C-10, C-20 | H-1β, H-2B, H-5 |
|  |  | β: 2.54 (1H, dd, 13.2, 6.2) | C-2, C-3, C-5, C-9, C-10, (C-18), C-20 | H-1α, H-11, H-20 |
| 2 | 18.6, CH_2_ | A: 2.34 (1H, m) | C-1, C-3, C-4 | H-20 |
|  |  | B: 2.43 (1H, dd, 18.1, 5.4) | C-1, C-3, C-4, C-10 | H-1α, H-20 |
| 3 | 128.6, C | - |  |  |
| 4 | 164.0, C | - |  |  |
| 5 | 41.4, CH | 2.67 (1H, br s) |  | H-1α, H-6α |
| 6 | 19.8, CH_2_ | α: 2.30 (1H, m) |  | H-5, H-6β, H-7A, H-7B, H-19 |
|  |  | β: 1.91 (1H, m) |  | H-6α, H-7B, H-19, H-20 |
| 7 | 23.8, CH_2_ | A: 2.83 (1H, m) |  | H-6α |
|  |  | B: 2.91 (1H, dd, 17.8, 7.5) | C-5, C-6, C-8, C-9, C-14 | H-6α, H-6β |
| 8 | 123.2, C | - |  |  |
| 9 | 144.7, C | - |  |  |
| 10 | 37.0, C | - |  |  |
| 11 | 116.8, CH | 6.91 (1H, d, 8.2) | C-8, C-10, C-13 | H-1β |
| 12 | 123.7, CH | 6.99 (1H, d, 8.2) | C-9, C-14, C-15 | H-16, H-17 |
| 13 | 133.7, C | - |  |  |
| 14 | 152.4, C | - |  |  |
| 15 | 27.4, CH | 3.27 (1H, sep, 6.9) | C-12, C-13, C-14, C-16, C-17 |  |
| 16 | 23.1, CH_3_ | 1.18 (3H, d, 6.9) | C-13, C-15, C-17 | H-12 |
| 17 | 23.0, CH_3_ | 1.20 (3H, d, 6.9) | C-13, C-15, C-16 | H-12 |
| 18 | 173.4, C | - |  |  |
| 19 | 99.4, CH | 6.10 (1H, br s) |  | H-6α, H-6β |
| 20 | 22.6, CH_3_ | 1.02 (3H, br s) | C-1, C-5, C-9, C-10 | H-1β, H-2A, H-2B, H-6β |

*^a^* ^1^H NMR (600.13) and ^13^C NMR (150.90 MHz) data obtained with samples in methanol-*d*_4_. *^b^* Assignments based on HSQC and HMBC experiments.
*^c^* Multiplicities reported as apparent splittings: s = singlet, d = doublet, sep = septet, m = multiplet (incl. overlapping resonances), br = broad. α denotes hydrogen pointing into the plane and β denotes hydrogen pointing out of the plane. A denotes the lowest chemical shift value and B denotes the highest chemical shift value.

**Supplementary table 13** ^1^H and ^13^C NMR data and 2D HMBC and ROESY correlations for **17**

| Pos. | *δ*_C_, type*^a,b^* | *δ*_H_, nH, multiplicity (*J* in Hz)*^a,c^* | HMBC | ROESY |
| --- | --- | --- | --- | --- |
| 1 | 34.4, CH_2_ | α: 1.22 (1H, m) | C-2, C-3, C-9, C-10, C-20 | H-3 |
|  |  | β: 2.78 (1H, dt, 13.5, 3.6) | C-3, C-5, C-10 | H-2, H-20 |
| 2 | 27.7, CH_2_ | 1.72 (2H, m) | C-1, C-3, C-4, C-10 | H-1β, H-19 |
| 3 | 78.3, CH | 3.24 (1H, m) | C-2, C-4, C-18, C-19 | H-1α, H-18 |
| 4 | 39.0, C | - |  |  |
| 5 | 51.0, CH | 1.06 (1H, m) | C-4, C-6, C-10, C-19, C-20 | H-3, H-6α, H-7B, H-7A |
| 6 | 17.1, CH_2_ | α: 1.87 (1H, br dd, 13.5, 7.5) | C-4, C-5, C-7, C-8, C-10 | H-5, H-7B, H-18 |
|  |  | β: 1.45 (1H, dtd, 13.5, 11.7, 5.7) | C-5, C-7, C-10 | H-7B, H-19 |
| 7 | 26.1, CH_2_ | A: 2.30 (1H, ddd, 20.2, 11.7, 7.5) | C-6, C-8, C-9, C-14 | H-5, H-6α |
|  |  | B: 2.71 (1H, br dd, 20.2, 5.7) | C-5, C-6, C-8, C-9, C-14 | H-5, H-6α, H-6β |
| 8 | 142.7, C | - |  |  |
| 9 | 150.0, C | - |  |  |
| 10 | 38.1, C | - |  |  |
| 11 | 187.78, C | - |  |  |
| 12 | 131.8, CH | 6.30 (1H, d, 1.0) | C-9, C-11/C-14, C-15 | H-16/17 |
| 13 | 152.9, C | - |  |  |
| 14 | 187.83, C | - |  |  |
| 15 | 26.2, CH | 2.96 (1H, sep d, 6.9, 1.0) | C-12, C-13, C-14, C-16, C-17 |  |
| 16 | 21.3, CH_3_ | 1.07 (3H, d, 6.9) | C-13, C-15, C-17 | H-12 |
| 17 | 21.3, CH_3_ | 1.08 (3H, d, 6.9) | C-13, C-15, C-16 | H-12 |
| 18 | 28.2, CH_3_ | 1.03 (3H, s) | C-3, C-4, C-5, C-19 | H-3, H-6α |
| 19 | 15.7, CH_3_ | 0.85 (3H, s) | C-3, C-4, C-5, C-18 | H-2, H-6β, H-20 |
| 20 | 20.1, CH_3_ | 1.26 (3H, s) | C-1, C-5, C-9, C-10 | H-1β, H-19 |

*^a^* ^1^H NMR (600.13) and ^13^C NMR (150.90 MHz) data obtained with samples in CDCl_3_. *^b^* Assignments based on HSQC and HMBC experiments.
*^c^* Multiplicities reported as apparent splittings: s = singlet, d = doublet, t = triplet, sep = septet, m = multiplet (incl. overlapping resonances), br = broad. α denotes hydrogen pointing into the plane and β denotes hydrogen pointing out of the plane. A denotes the lowest chemical shift value and B denotes the highest chemical shift value.

**Supplementary table 14** ^1^H and ^13^C NMR data and 2D HMBC and ROESY correlations for **18**

| Pos. | *δ*_C_, type*^a,b^* | *δ*_H_, nH, multiplicity (*J* in Hz)*^a,c^* | HMBC | ROESY |
| --- | --- | --- | --- | --- |
| 1 | 34.0, CH_2_ | α: 1.23 (1H, m) | C-2, C-10, C-20 | H-1β, H-3 |
|  |  | β: 2.79 (1H, dt, 13.5, 3.3) | C-3, C-5, C-10 | H-1α, H-2, H-20 |
| 2 | 27.1, CH_2_ | 1.76 (2H, m) | C-1, C-3, C-4, C-10 | H-1β, H-20 |
| 3 | 75.1, CH | 3.68 (1H, dd, 10.3, 5.8) | C-2, C-4, C-18, C-19 | H-1α, H-5, H-18A |
| 4 | 42.0, C | - |  |  |
| 5 | 45.2, CH | 1.29 (1H, d, 12.5) | C-4, C-6, C-7, C-10, C-18, C-19, C-20 | H-3, H-7B, H-18A |
| 6 | 17.1, CH_2_ | α: 1.73 (1H, m) | C-5, C-7, C-8, C-10 | H-7B, H-18A, H-18B |
|  |  | β: 1.48 (1H, qd, 12.5, 5.7) | C-5, C-7, C-10 | H-7B, H-19 |
| 7 | 25.8, CH_2_ | A: 2.32 (1H, ddd, 20.2, 11.6, 7.4) | C-6, C-8, C-9, C-14 | H-7B, H-6α |
|  |  | B: 2.69 (1H, dd, 20.2, 5.5) | C-5, C-6, C-8, C-9, C-14 | H-5, H-6α, H-6β, H-7A |
| 8 | 142.6, C | - |  |  |
| 9 | 149.9, C | - |  |  |
| 10 | 38.0, C | - |  |  |
| 11 | 187.7, C | - |  |  |
| 12 | 131.8, CH | 6.31 (1H, s) | C-9, C-11, C-14, C-15 | H-15, H-16/17 |
| 13 | 152.9, C | - |  |  |
| 14 | 187.6, C | - |  |  |
| 15 | 26.2, CH | 2.97 (1H, sep, 6.9) | C-12, C-13, C-14, C-16, C-17 | H-12 |
| 16 | 21.2, CH_3_ | 1.08 (3H, d, 6.9) | C-13, C-15, C-17 | H-12 |
| 17 | 21.2, CH_3_ | 1.09 (3H, d, 6.9) | C-13, C-15, C-16 | H-12 |
| 18 | 70.4, CH_2_ | A: 3.42 (1H, d, 10.3) | C-3, C-4, C-5, C-19 | H-3, H-5, H-6α, H-19 |
|  |  | B: 3.74 (1H, d, 10.3) | C-3, C-4, C-5, C-19 | H-6α, H-19 |
| 19 | 11.6, CH_3_ | 0.92 (3H, s) | C-3, C-4, C-5, C-18 | H-6β, H-18A, H-18B, H-20 |
| 20 | 20.4, CH_3_ | 1.31 (3H, s) | C-1, C-5, C-9, C-10 | H-1β, H-2, H-19 |

*^a^* ^1^H NMR (600.13) and ^13^C NMR (150.90 MHz) data obtained with samples in CDCl_3_. *^b^* Assignments based on HSQC and HMBC experiments.
*^c^* Multiplicities reported as apparent splittings: s = singlet, d = doublet, t = triplet, sep = septet, m = multiplet (incl. overlapping resonances), br = broad. α denotes hydrogen pointing into the plane and β denotes hydrogen pointing out of the plane. A denotes the lowest chemical shift value and B denotes the highest chemical shift value.

**Supplementary table 15** and ^13^C NMR data and 2D HMBC and ROESY correlations for **19**

| Pos. | *δ*_C_, type*^a,b^* | *δ*_H_, nH, multiplicity (*J* in Hz)*^a,c^* | HMBC | ROESY |
| --- | --- | --- | --- | --- |
| 1 | 33.1, CH_2_ | A: 1.35 (1H, m) | C-2, C-3, C-5, C-10, C-20 | H-2B |
|  |  | B: 1.427 (1H, m) |  | H-3, H-17, H-18 |
| 2 | 28.9, CH_2_ | A: 1.60 (1H, m) | C-1, C-3 |  |
|  |  | B: 1.69 (1H, m) |  | H-1A |
| 3 | 79.0, CH | 3.22 (1H, dd, 11.8, 5.0) | C-2, C-4, C-18, C-19 | H-1B, H-5, H-18 |
| 4 | 38.9, C | - |  |  |
| 5 | 46.0, CH | 1.415 (1H, m) | C-3, C-4, C-6, C-7, C-10, C-18, C-19, C-20 | H-3, H-18 |
| 6 | 17.5, CH_2_ | A: 1.407 (1H, m) | C-5 | H-19 |
|  |  | B: 1.62 (1H, m) | C-4, C-5, C-7, C-8, C-10 | H-19 |
| 7 | 38.6, CH_2_ | A: 1.62 (1H, m) | C-6, C-9 |  |
|  |  | B: 1.80 (1H, dd, 11.5, 7.6) | C-5, C-6, C-8, C-14, C-17 |  |
| 8 | 37.9, C | - |  |  |
| 9 | 53.6, CH | 1.24 (1H, dd, 12.9, 3.0) | C-1, C-8, C-10, C-11, C-12, C-17, C-20 | H-14, H-20 |
| 10 | 36.9, C | - |  |  |
| 11 | 24.5, CH_2_ | A: 1.32 (1H, qd, 12.9, 4.0) | C-9, C-12 | H-12A, H-12B, H-17 |
|  |  | B: 1.64 (1H, m) | C-9 | H-12A, H-12B, H-20 |
| 12 | 37.8, CH_2_ | A: 1.94 (1H, tdt, 12.8, 4.6, ~1) | C-11, C-13, C-15 | H-11A, H-11B |
|  |  | B: 2.41 (1H, ddd, 12.8, 4.0, 2.6) | C-9, C-11, C-13, C-14, C-15 | H-11A, H-11B, H-15B |
| 13 | 147.5, C | - |  |  |
| 14 | 60.8, CH | 1.89 (1H, t, 6.5) | C-8, C-9, C-13, C-15, C-16, C-17 | H-9 |
| 15 | 107.0, CH_2_ | A: 4.69 (1H, dt, 1.4, 1.2) | C-12, C-13, C-14 | H-15B, H-16 |
|  |  | B: 4.96 (1H, dt, 1.4, 1.2) | C-12, C-13, C-14 | H-12B, H-15A |
| 16 | 59.0, CH_2_ | 3.76 (2H, d, 6.5) | C-8, C-13, C-14 | H-7A, H-7B, H-15A, H-17 |
| 17 | 21.0, CH_3_ | 0.85 (3H, s) | C-7, C-8, C-9, C-14 | H-1B, H-11A, H-16 |
| 18 | 29.0, CH_3_ | 0.98 (3H, s) | C-3, C-4, C-5, C-19 | H-1B, H-3, H-5, H-6B |
| 19 | 15.8, CH_3_ | 0.79 (3H, s) | C-3, C-4, C-5, C-18 | H-6A, H-6B |
| 20 | 22.3, CH_3_ | 0.95 (3H, s) | C-1, C-5, C-9, C-10 | H-9, H-11B |

*^a^* ^1^H NMR (600.13) and ^13^C NMR (150.90 MHz) data obtained with samples in CDCl_3_. *^b^* Assignments based on HSQC and HMBC experiments.
*^c^* Multiplicities reported as apparent splittings: s = singlet, d = doublet, t = triplet, q = quartet, m = multiplet (incl. overlapping resonances), br = broad. α denotes hydrogen pointing into the plane and β denotes hydrogen pointing out of the plane. A denotes the lowest chemical shift value and B denotes the highest chemical shift value.

**Supplementary table 16** ^1^H and ^13^C NMR data and 2D HMBC and ROESY correlations for **20**

| Pos. | *δ*_C_, type*^a,b^* | *δ*_H_, nH, multiplicity (*J* in Hz)*^a,c^* | HMBC | ROESY |
| --- | --- | --- | --- | --- |
| 1 | 37.0, CH_2_ | α: 1.16 (1H, td, 13.2, 3.7) | C-2, C-5, C-10, C-20 | H-3 |
|  |  | β: 1.79 (1H, dt, 13.2, 3.5) | C-3, C-5 | H-20 |
| 2 | 27.9, CH_2_ | A: 1.60 (1H, m) | C-1, C-3 | H-19 |
|  |  | B: 1.71 (1H, m) | C-3 |  |
| 3 | 78.8, CH | 3.25 (1H, dd, 11.8, 4.3) | C-4, C-18, C-19 | H-1α, H-5, H-18 |
| 4 | 39.1, C | - |  |  |
| 5 | 54.6, CH | 1.08 (1H, dd, 12.5, 2.7) | C-4, C-6, C-7, C-9, C-10, C-18, C-19, C-20 | H-3, H-7α, H-9, H-18 |
| 6 | 24.0, CH_2_ | α: 1.74 (1H, dddd, 13.0, 5.0, 2.7, 2.5) | C-5, C-7, C-8, C-10 | H-7β, H-18 |
|  |  | β: 1.39 (1H, tdd, 13.0, 12.5, 4.2) | C-5, C-7, C-10 | H-7β, H-19 |
| 7 | 38.1, CH_2_ | α: 1.96 (1H, ddd, 13.0, 12.8, 5.0) | C-6, C-8, C-17 | H-5 |
|  |  | β: 2.40 (1H, ddd, 12.8, 4.2, 2.5) | C-5, C-6, C-8, C-9, C-17 | H-6α, H-6β, H-17B |
| 8 | 147.8, C | - |  |  |
| 9 | 55.9, CH | 1.54 (1H, m) | C-7, C-8, C-10, C-11, C-12, C-17, C-20 | H-5, H-12B, H-14, H-17A, H-20 |
| 10 | 39.2, C | - |  |  |
| 11 | 21.8, CH_2_ | A: 1.47 (1H, m) | C-8, C-9, C-12, C-13 | H-12B, H-14, H-17A, H-20 |
|  |  | B: 1.59 (1H, m) | C-8, C-9, C-12 |  |
| 12 | 38.3, CH_2_ | A: 1.83 (1H, ddd, 14.0, 9.4, 6.6) | C-9, C-11, C-13, C-14, C-15 | H-14 |
|  |  | B: 2.17 (1H, ddd, 14.0, 10.0, 3.8) | C-11, C-13, C-14 | H-9, H-11A, H-14, H-15, H-17A |
| 13 | 142.7, C | - |  |  |
| 14 | 118.0, CH | 5.31 (1H, t sext, 7.1, 1.2) | C-12, C-15, C-16 | H-9, H-11A, H-12A, H-12B |
| 15 | 16.5, CH_3_ | 1.69 (3H, br s) | C-12, C-13, C-14 | H-12B, H-16 |
| 16 | 61.4, CH_2_ | 4.58 (2H, d, 7.1) | C-13, C-14, C-1' | H-15 |
| 17 | 106.0, CH_2_ | A: 4.53 (1H, q, ~1) | C-7, C-8, C-9 | H-9, H-11A, H-12A, H-12B, H-17B |
|  |  | B: 4.85 (1H, q, ~1.4) | C-7, C-9 | H-7β, H-17A |
| 18 | 28.3, CH_3_ | 1.00 (3H, s) | C-3, C-4, C-5, C-19 | H-3, H-5, H-6α |
| 19 | 15.3, CH_3_ | 0.78 (3H, s) | C-3, C-4, C-5, C-18 | H-2A, H-6β |
| 20 | 14.5, CH_3_ | 0.69 (3H, s) | C-1, C-5, C-9, C-10 | H-9, H-11A |
| 1' | 171.0, C | - |  |  |
| 2' | 21.1, CH_3_ | 2.06 (3H, s) | C-1' |  |

*^a^* ^1^H NMR (600.13) and ^13^C NMR (150.90 MHz) data obtained with samples in CDCl_3_. *^b^* Assignments based on HSQC and HMBC experiments.
*^c^* Multiplicities reported as apparent splittings: s = singlet, d = doublet, t = triplet, sext = sextet, m = multiplet (incl. overlapping resonances), br = broad. α denotes hydrogen pointing into the plane and β denotes hydrogen pointing out of the plane. A denotes the lowest chemical shift value and B denotes the highest chemical shift value.

**Supplementary table 17** ^1^H and ^13^C NMR data and 2D HMBC and ROESY correlations for **21**

| Pos. | *δ*_C_, type*^a,b^* | *δ*_H_, nH, multiplicity (*J* in Hz)*^a,c^* | HMBC | ROESY |
| --- | --- | --- | --- | --- |
| 1 | 59.4, CH_2_ | 4.16 (2H, d, 6.9) | C-2, C-3 | H-3-Me |
| 2 | 123.4, CH | 5.42 (1H, t sext, 6.9, 1.3) | C-3-Me, C-4 | H-4 |
| 3 | 139.6, C | - |  |  |
| 3-Me | 16.3, CH_3_ | 1.68 (3H, br s) | C-2, C-3, C-4 | H-1 |
| 4 | 39.5, CH_2_ | 2.04 (2H, m) | C-2, C-3, C-3-Me, C-5 | H-2 |
| 5 | 26.3, CH_2_ | 2.11 (2H, m) | C-4, C-6, C-7 |  |
| 6 | 123.9, CH | 5.11 (1H, t sext, 6.9, 1.2) | C-5, C-7-Me, C-8 |  |
| 7 | 135.1, C | - |  |  |
| 7-Me | 15.9, CH_3_ | 1.60 (3H, br s) | C-6, C-7, C-8 |  |
| 8 | 39.6, CH_2_ | 1.99 (2H, m) | C-6, C-7, C-7-Me, C-9, C-10 |  |
| 9 | 26.5, CH_2_ | 2.08 (2H, m) | C-7, C-8, C-10, C-11 |  |
| 10 | 124.8, CH | 5.16 (1H, t sext, 6.9, 1.2) | C-9, C-11-Me, C-12 | H-12B, H-13 |
| 11 | 134.0, C | - |  |  |
| 11-Me | 16.0, CH_3_ | 1.62 (3H, br s) | C-10, C-11, C-12 |  |
| 12 | 36.3, CH_2_ | A: 2.08 (1H, m) | C-10, C-11, C-11-Me, C-13, C-14 | H-14 |
|  |  | B: 2.16 (1H, m) | C-10, C-11, C-11-Me, C-13, C-14 | H-10, H-14 |
| 13 | 27.4, CH_2_ | 1.63 (2H, m) | C-11, C-12, C-14, C-15 | H-10 |
| 14 | 64.1, CH | 2.70 (1H, t, 6.3) | C-12, C-13, C-15, C-15-Me' | H-12A, H-12B, H-15-Me, H-15-Me' |
| 15 | 58.2, C | - |  |  |
| 15-Me | 18.7, CH_3_ | 1.26 (3H, s) | C-14, C-15, C-15-Me' | H-14 |
| 15-Me' | 24.8, CH_3_ | 1.30 (3H, s) | C-14, C-15, C-15-Me | H-14 |

*^a^* ^1^H NMR (600.13) and ^13^C NMR (150.90 MHz) data obtained with samples in CDCl_3_. *^b^* Assignments based on HSQC and HMBC experiments.
*^c^* Multiplicities reported as apparent splittings: s = singlet, d = doublet, t = triplet, sext = sextet, m = multiplet (incl. overlapping resonances), br = broad. α denotes hydrogen pointing into the plane and β denotes hydrogen pointing out of the plane. A denotes the lowest chemical shift value and B denotes the highest chemical shift value.

**Supplementary table 18** ^1^H and ^13^C NMR data and 2D HMBC and ROESY correlations for **22**

| Pos. | *δ*_C_, type*^a,b^* | *δ*_H_, nH, multiplicity (*J* in Hz)*^a,c^* | HMBC | ROESY |
| --- | --- | --- | --- | --- |
| 1 | 59.3, CH_2_ | 4.15 (2H, br d, 6.9) | C-2, C-3 | H-3-Me |
| 2 | 123.7, CH | 5.42 (1H, t sext, 6.9, 1.2) | C-3-Me, C-4 | H-4 |
| 3 | 139.3, C | - |  |  |
| 3-Me | 16.2, CH_3_ | 1.68 (3H, br s) | C-2, C-3, C-4 | H-1, H-4 |
| 4 | 39.3, CH_2_ | 2.07 (2H, m) |  | H-2, H-3-Me |
| 5 | 25.9, CH_2_ | 2.14 (2H, m) | C-4, C-6, C-7 |  |
| 6 | 124.3, CH | 5.13 (1H, t sext, 6.8, 1.2) | C-4, C-5, C-7-Me, C-8 |  |
| 7 | 136.2, C | - |  |  |
| 7-Me | 16.0, CH_3_ | 1.64 (3H, br s) | C-6, C-7, C-8 | H-8 |
| 8 | 42.5, CH_2_ | 2.10 (2H, m) | C-6, C-7, C-7-Me, C-9, C-10 | H-7-Me, H-10, H-15-αMe |
| 9 | 24.2, CH_2_ | A: 1.48 (1H, m) | C-8, C-10, C-15 |  |
|  |  | B: 1.55 (1H, m) | C-8, C-10, C-11 | H-11-Me |
| 10 | 55.3, CH | 1.10 (1H, t, 4.3) | C-8, C-9, C-11, C-11-Me, C-15 | H-8, H-14 |
| 11 | 73.4, C | - |  |  |
| 11-Me | 23.0, CH_3_ | 1.16 (3H, s) | C-10, C-11, C-12 | H-9B, H-12B, H-13A, H-15-βMe |
| 12 | 40.9, CH_2_ | A: 1.45 (1H, m) | C-13 |  |
|  |  | B: 1.78 (1H, m) | C-10, C-11, C-14 | H-11-Me |
| 13 | 28.9, CH_2_ | A: 1.49 (1H, m) | C-14 | H-11-Me |
|  |  | B: 1.75 (1H, m) | C-10, C-11, C-14 | H-14 |
| 14 | 78.3, CH | 3.31 (1H, dd, 10.6, 2.8) |  | H-10, H-13B, H-15-αMe |
| 15 | 40.4, C | - |  |  |
| 15-αMe | 28.0, CH_3_ | 1.04 (3H, s) | C-10, C-14, C-15, C-15-βMe | H-8, H-14 |
| 15-βMe | 14.8, CH_3_ | 0.80 (3H, s) | C-10, C-14, C-15, C-15-αMe | H-11-Me |

*^a^* ^1^H NMR (600.13) and ^13^C NMR (150.90 MHz) data obtained with samples in CDCl_3_. *^b^* Assignments based on HSQC and HMBC experiments.
*^c^* Multiplicities reported as apparent splittings: s = singlet, d = doublet, t = triplet, sext = sextet, m = multiplet (incl. overlapping resonances), br = broad. α denotes Me pointing into the plane and β denotes Me pointing out of the plane. A denotes the lowest chemical shift value and B denotes the highest chemical shift value.

**Supplementary table 19** ^1^H and ^13^C NMR data and 2D HMBC and ROESY correlations for **23**

| Pos. | *δ*_C_, type*^a,b^* | *δ*_H_, nH, multiplicity (*J* in Hz)*^a,c^* | HMBC | ROESY |
| --- | --- | --- | --- | --- |
| 1 | 32.7, CH_2_ | α: 1.69 (1H, m) | C-2, C-10, C-20 | H-1β, H-2B, H-5 |
|  |  | β: 2.46 (1H, m) | C-2, C-5, C-9, C-20 | H-1α, H-11, H-20 |
| 2 | 22.5, CH_2_ | A: 2.45 (1H, m) | C-1, C-3, C-4, C-10 | H-19B |
|  |  | B: 2.52 (1H, m) | C-1, C-3, C-4 | H-1α, H-19A |
| 3 | 132.0, C | - |  |  |
| 4 | 150.7, C | - |  |  |
| 5 | 39.2, CH | 2.60 (1H, m) |  | H-1α, H-6α, H-7B |
| 6 | 17.5, CH_2_ | α: 3.28 (1H, m) | C-8, C-10 | H-5, H-7B |
|  |  | β: 1.73 (1H, m) | C-5, C-7, C-10 | H-20 |
| 7 | 22.5, CH_2_ | A: 2.46 (1H, m) |  |  |
|  |  | B: 2.80 (1H, td, 9.5, 7.6) | C-5, C-6, C-8, C-9, C-14 | H-6α, H5 |
| 8 | 120.8, C | - |  |  |
| 9 | 144.5, C | - |  |  |
| 10 | 36.1, C | - |  |  |
| 11 | 115.7, CH | 6.91 (1H, d, 8.1) | C-8, C-10, C-12, C-13 | H-1β, H-20 |
| 12 | 123.0, CH | 7.04 (1H, d, 8.1) | C-9, C-11, C-14, C-15 | H-16, H-17 |
| 13 | 131.2, C | - |  |  |
| 14 | 150.9, C | - |  |  |
| 15 | 26.9, CH | 3.18 (1H, sep, 7.0) | C-12, C-13, C-14, C-16, C-17 |  |
| 16 | 22.6, CH_3_ | 1.24 (3H, d, 7.0) | C-13, C-15, C-17 | H-12 |
| 17 | 22.6, CH_3_ | 1.26 (3H, d, 7.0) | C-13, C-15, C-16 | H-12 |
| 18 | 173.5, C | - |  |  |
| 19 | 53.9, CH_2_ | A: 3.90 (1H, dd, 18.7, 2.1) | C-3, C-4, C-18 | H-2B, H-1' |
|  |  | B: 3.94 (1H, dd, 18.7, 2.7) | C-3, C-4, C-18 | H-2A, H-1' |
| 20 | 22.4, CH_3_ | 1.03 (3H, s) | C-1, C-5, C-9, C-10 | H-1β, H-6β, H-11 |
| 1' | 46.1, CH_2_ | A: 3.56 (1H, m) | C-18, C-19, C-2' | H-19 |
|  |  | B: 3.63 (1H, m) | C-18, C-19, C-2' | H-19 |
| 2' | 62.2, CH_2_ | 3.82 (2H, m) | C-1' |  |

*^a^* ^1^H NMR (600.13) and ^13^C NMR (150.90 MHz) data obtained with samples in CDCl_3_. *^b^* Assignments based on HSQC and HMBC experiments.
*^c^* Multiplicities reported as apparent splittings: s = singlet, d = doublet, t = triplet, sep = septet, m = multiplet (incl. overlapping resonances), br = broad. α denotes hydrogen pointing into the plane and β denotes hydrogen pointing out of the plane. A denotes the lowest chemical shift value and B denotes the highest chemical shift value.

**Supplemental table 20. Overview of cDNA libraries used for CYP mining.** Trancriptomic dataset used for data mining of cytochrome P450 enzymes involved in triptonide biosynthesis.

| SRA accession / Sample ID | Tripterygium species and tissue | Sequencing technology | Date published | Reference |  |
| --- | --- | --- | --- | --- | --- |
| SRR3583049 / SAMN05172534 | *Tripterygium regelii*, adventitious root | Illumina HiSeq 3000 | 30/05/2016 | <https://www.ncbi.nlm.nih.gov/sra/SRR3583049/> |  |
| SRR708388 / SAMN01801623 | *Tripterygium wilfordii*, leaf | Illumina HiSeq 2000 | 22/07/2015 | <https://www.ncbi.nlm.nih.gov/sra/?term=SRR708388> |  |
| SRR1171189 / SAMN02351604 | *Tripterygium wilfordii*, leaf | Illumina HiSeq 2000 | 18/04/2014 | <https://www.ncbi.nlm.nih.gov/sra/?term=SRR1171189> |  |
| SRR4294733 / SAMN05757817 | *Tripterygium wilfordii*, root | Illumina HiSeq 2500 | 01/10/2016 | <https://www.ncbi.nlm.nih.gov/sra/?term=SRR4294733> | Ref: ^1^ |

**Supplementary table 21:** **List of CYP genes isolated from *T. wilfordii* cDNA**

List of genes in this paper. Names for CYP protein sequences was kindly provided by Prof. David Nelson according to established guidelines for naming^22^

| **Gene name** | **Comment** | **Reference** |
| --- | --- | --- |
| **TwCYP51G61** |  | This work |
| **TwCYP701A58** |  | ^23^ |
| **TwCYP706B23** |  | This work |
| **TwCYP706B24** |  | This work |
| **TwCYP711A61** |  | ^23^ |
| **TwCYP711A62** |  | ^23^ |
| **TwCYP712K1** |  | ^23^ |
| **TwCYP712K2** |  | ^23^ |
| **TwCYP712K3** |  | ^23^ |
| **TwCYP716C52** |  | This work |
| **TwCYP716C52v2** | N-trunc version of CYP716C52 | ^23^ |
| **TwCYP716C57** |  | This work |
| **TwCYP716E53** |  | ^23^ |
| **TwCYP716E66** |  | This work |
| **TwCYP71AN56** |  | ^23^ |
| **TwCYP71AP40** |  | ^23^ |
| **TwCYP71B102** |  | ^23^ |
| **TwCYP71BE83** |  | ^23^ |
| **TwCYP71BE84** |  | ^23^ |
| **TwCYP71BE85** |  | ^23^ genbank acc. number: ON375998 |
| **TwCYP71BE85v2** | 3 aa differences to CYP71BE85 | ^23^ |
| **TwCYP71BE86** |  | ^23^, genbank acc. number: ON375999 |
| **TwCYP71E23** |  | ^23^ |
| **TwCYP72A768v3** |  | This work |
| **TwCYP72A768v4** | 6 aa differences to CYP72A768v3 | This work |
| **TwCYP72A768v5** | 4 aa differences to CYP72A768v4 | This work |
| **TwCYP72D19** |  | ^23^ |
| **TwCYP749A88** |  | ^23^ |
| **TwCYP749A89** |  | ^23^ |
| **TwCYP76A55** |  | ^23^ |
| **TwCYP76T30** |  | ^23^ |
| **TwCYP76Y20** |  | ^23^ |
| **TwCYP81AM1** |  | ^23^, ^24^ |
| **TwCYP81AM2** |  | This work |
| **TwCYP81AN8** |  | This work |
| **TwCYP82AQ1v1** |  | ^23^ |
| **TwCYP82AQ1v2** | 1 aa difference to CYP82AQ1v1 | This work |
| **TwCYP82AS1** |  | ^23^ |
| **TwCYP82D166** |  | ^23^ |
| **TwCYP82D167** |  | ^23^ |
| **TwCYP82D168** |  | ^23^ |
| **TwCYP82D169** |  | ^23^ |
| **TwCYP82D212** |  | ^23^ |
| **TwCYP82D213** |  | This work, genbank acc. number: ON376000 |
| **TwCYP82D213v2** | 1 aa difference to CYP82D213 | This work |
| **TwCYP82D217** |  | This work |
| **TwCYP82D274** |  | This work, genbank acc. number: ON376001 |
| **TwCYP82D274v2** | 3 aa differences to CYP82D274 | This work |
| **TwCYP82D274v3** | 1 aa difference to CYP82D274 | This work |
| **TwCYP82D274v4** | 20 aa differences to CYP82D274 | This work |
| **TwCYP82D275** |  | This work |
| **TwCYP82J13** |  | ^23^ |
| **TwCYP86B39** |  | ^23^ |
| **CYP88A43** |  | ^23^ |
| **CYP88A44v1** | 7 aa differences to CYP88A43 | ^23^ |
| **CYP88A44v2** | 1 aa difference to CYP88A44v1 | ^23^ |
| **CYP88A88** | 9 aa difference to CYP88A44v1 | ^23^ |
| **CYP88A89** |  | ^23^ |
| **CYP88H1** |  | ^23^ |
| **CYP88H10** |  | ^23^ |
| **CYP88H11** | 11 aa differences to CYP88H10 | ^23^ |
| **CYP88H12** | 2 aa differences to CYP88H11 | ^23^ |
| **CYP88H2v2** |  | ^23^ |
| **CYP88H6** |  | ^23^ |
| **CYP88H7** |  | ^23^ |
| **CYP88H8** |  | ^23^ |
| **CYP88H9** |  | ^23^ |
| **CYP96A145** |  | ^23^ |
| **Twcytb5-F** |  | This work, , genbank acc. number: ON376007 |
| **Twcytb5-E** |  | This work, genbank acc. number: ON376006 |
| **Twcytb5-D** |  | This work, genbank acc. number: ON376005 |
| **Twcytb5-C** |  | This work, genbank acc. number: ON376004 |
| **Twcytb5-B** |  | This work, genbank acc. number: ON376003 |
| **Twcytb5-A** |  | This work, genbank acc. number: ON376002 |
| **TwPOR1** |  | This work,, genbank acc. number: ON376008 |
| **TwPOR2** |  | ^23^ |

**Supplementary table 22: CYP genes used in phylogeny fig. 1B.** Name and source of protein sequences. Remaining sequences can be found in Bathe et al 2019^7^.

| **Gene name** | **Comment** | **Source of sequence** |
| --- | --- | --- |
| **AtCYP82G1** | Steroid biosynthesis | BAB61873 |
| **GhCYP82D109** | Homoterpenoid biosynthesis | NP_001313857 |
| **SbCYP82D2** | Flavone hydroxylase | MF363008 |
| **ObCYP82D33** | flavone-6-hydroxylase and 7-O-demethylation | AGF30364 |
| **MpCYP82D62** | flavone-6-hydroxylase and 7-O-demethylation | JX162214 |
| **AtCYP82C2** | Xanthotoxin 5-hydroxylase | NP_194925 |
| **AtCYP82C4** | Xanthotoxin 5-hydroxylase | NP_194922 |
| **SmCYP71D411** | Diterpenoid | Ref: ^25^. chr6 (65,051,276 -> 65,050,400; 65,049,664 -> 65,049,042) |
| **SmCYP71D375** | Diterpenoid | Ref: ^25^. chr6 (65,230,679 -> 65,229,791, 65,228,929 -> 65,228,298) |
| **SmCYP71D464** |  | Ref: ^25^. chr6 (65,202,692 -> 65,201,960, 65,198,485 -> 65,197,863 |
| **SmCYP71D373** |  | Ref: ^25^. chr6 (65,067,928 -> 65,067,156, 65,066,655 -> 65,066,028) |

**Supplementary table 23 List of homologs of the triptonide biosynthetic genes identified in the the *T. wilfordii* genome^26^.** Homologs of the triptonide biosynthestic genes identified in the *T. wilfordii* genome. *T. wilfordii* genome was used as query for BLAST searches against the isolated cDNA sequences of the triptonide biosynthetic genes. Unannotated sequences in the genome aligning to the cDNA was extracted together with information about chromosome location, number of putative exons, length and direction of translation. Sequence identity for extracted genome sequences was based on multiple sequence alignment including the cDNA sequence having the highest BLAST hit score.

| **BLAST top hit** | **%ID** | **annotated**  **CDS** | ***T. Wilfordii* Chr.** | **comment** | **Length (bp)** | **# exons** | **gene 5'end on Tw chr.** | **gene 3'end on Tw chr.** |
| --- | --- | --- | --- | --- | --- | --- | --- | --- |
| TwTPS27 | 49,9% | KAF5728148.1 | CM023888.1 |  | 2031 | 12 | 1963535 | 1958926 |
|  | 98,4% | KAF5728169.1 | CM023888.1 |  | 1620 | 6 | 2129543 | 2132065 |
|  | 99,6% | KAF5728149.1 | CM023888.1 |  | 1770 | 6 | 1971984 | 1974655 |
| TwTPS9 | 79,8% | KAF5728168.1 | CM023888.1 | short anno. | 1527 | 9 | 2121803 | 2117172 |
| TwPOR3 | 98,0% | KAF5725931.1 | CM023890.1 |  | 2481 | 22 | 5396940 | 5384927 |
| TwPOR2 | 99,0% | KAF5746766.1 | CM023873.1 |  | 3957 | 24 | 9151195 | 9165045 |
| TwPOR1 | 99,7% | KAF5742890.1 | CM023876.1 |  | 2127 | 18 | 11354241 | 11348846 |
| TwCYP82D275 | 100% | NA | CM023879.1 |  | 1596 | 2 | 4370062 | 4368213 |
| TwCYP82D274 | 86,5% | NA | CM023879.1 |  | 1602 | 2 | 4480327 | 4482174 |
|  | 79,7% | NA | CM023879.1 |  | 1533 | 2 | 4397269 | 4395319 |
|  | 79,1% | NA | CM023879.1 |  | 1533 | 2 | 4382761 | 4380708 |
|  | 78,7% | NA | CM023879.1 |  | 1519 | 2 | 4375917 | 4373860 |
|  | 99,5% | NA | CM023879.1 |  | 1608 | 2 | 4362819 | 4360903 |
| TwCYP82D213 | 99,2% | NA | CM023875.1 |  | 1596 | 2 | 8979502 | 8977726 |
| TwCYP71BE86 | 85,7% | NA | CM023888.1 |  | 1518 | 2 | 5151682 | 5150087 |
|  | 63,4% | NA | CM023888.1 |  | 1527 | 2 | 5115988 | 5117820 |
|  | 79,7% | NA | CM023886.1 |  | 1513 | 2 | 643392 | 644975 |
|  | 81,8% | NA | CM023886.1 | put. pseudo gene | 1321 | 2 | 628749 | 630140 |
|  | 78,1% | NA | CM023886.1 |  | 1486 | 2 | 607787 | 609351 |
|  | 78,1% | NA | CM023886.1 |  | 1494 | 2 | 583135 | 584703 |
|  | 98,4% | NA | CM023888.1 |  | 1512 | 2 | 5140270 | 5138688 |
| TwCYP71BE85 | 80,2% | NA | CM023888.1 |  | 1477 | 2 | 3603292 | 3614820 |
|  | 98,2% | NA | CM023878.1 |  | 1540 | 2 | 395624 | 397226 |
| TwCYP71BE84 | 88,9% | NA | CM023886.1 |  | 1534 | 2 | 687815 | 689506 |
|  | 85,8% | NA | CM023889.1 |  | 1458 | 2 | 5422096 | 5423780 |
|  | 85,1% | NA | CM023886.1 |  | 1452 | 2 | 753209 | 754902 |
|  | 83,8% | NA | CM023886.1 |  | 1452 | 2 | 742855 | 744549 |
|  | 84,3% | NA | CM023886.1 |  | 1455 | 2 | 750101 | 751666 |
|  | 88,4% | NA | CM023886.1 |  | 1524 | 2 | 709469 | 711231 |
|  | 83,4% | NA | CM023886.1 |  | 1458 | 2 | 658255 | 659947 |
|  | 89,2% | NA | CM023886.1 |  | 1518 | 2 | 697573 | 699312 |
|  | 99,5% | NA | CM023886.1 |  | 1523 | 2 | 690913 | 692678 |
| TwCYP71BE83 | 88,0% | NA | CM023888.1 |  | 1530 | 2 | 5147824 | 5146107 |
|  | 86,8% | NA | CM023888.1 |  | 1527 | 2 | 5131377 | 5133192 |
|  | 98,1% | NA | CM023886.1 |  | 1536 | 2 | 652553 | 650924 |
| Twcytb5-F | 100% | KAF5745760.1 | CM023874.1 |  | 375 | 3 | 15721305 | 15719472 |
| Twcytb5-E | 73,0% | KAF5748544 | CM023871.1 |  | 354 | 5 | 3267321 | 3265113 |
| Twcytb5-D | 98,4% | KAF5733280.1 | CM023884.1 |  | 384 | 3 | 11629832 | 11628418 |
| Twcytb5-C | 99,1% | KAF5739455.1 | CM023879.1 |  | 432 | 2 | 4725017 | 4724316 |
| Twcytb5-B | 100% | KAF5741263.1 | CM023877.1 |  | 414 | 3 | 1631383 | 1629315 |
| Twcytb5-A | 89,1% | KAF5732444.1 | CM023885.1 |  | 405 | 3 | 13014195 | 13012997 |
|  | 99,5% | KAF5731294.1 | CM023886.1 |  | 405 | 3 | 14590859 | 14589502 |

**Supplementary table 24 Putative diterpenoid derived compounds identified in extracts of *N. benthamiana* and *S. cerevisiae* expressing triptonide biosynthetic genes.** Overview of compounds depicted in supplementary figure 4, including accurate mass (m/z [M+H]), retention time (RT), predicted molecular formula, and peak area (PA). Identification of 14-OH-dehydroabitediene, triptophenolide and triptonide was confirmed by authentic standards. Putative gluthatione(C10H16N3O6S)-, glucose(C6H11O5)-, and malonate-glucose(C9H13O8)-diterpenoid conjugates identified in the leaf material from *N. benthamiana* expressing the triptonide biosynthetic genes, are denoted in the Conjugate column. In *S. cerevisiae* extracts putative nitrogen containing miltiradiene derived compounds identified in the NJV11.11 strain, are denoted in the conjugate column.

| **Identified in *N. Benthamiana* extracts** | | | | | |
| --- | --- | --- | --- | --- | --- |
| **CI** | **m/z** | **RT** | **Pred. Formula** | **Conjugate** | **PA (n=2)** |
| **4** | 287,2369537 | 23,09 | C20H30O | 14-OH-dehydroabietadiene | 5,02E+06 |
| **Nb2** | 531,2585297 | 10,91 | C29H38O9 | C20H25O-C9H13O8 | 2,63E+06 |
| **Nb3** | 283,2055893 | 12,4 | C20H26O |  | 2,83E+06 |
| **Nb4** | 561,232605 | 10,67 | C29H36O11 | C20H23O3-C9H13O8 | 2,99E+06 |
| **Nb5** | 445,2576752 | 9,03 | C26H36O6 | C20H25O-C6H11O5 | 2,01E+06 |
| **Nb6** | 283,2056351 | 10,28 | C20H26O |  | 3,61E+06 |
| **Nb7** | 545,2371063 | 10,71 | C29H36O10 | C20H23O2-C9H13O8 | 1,69E+06 |
| **Nb8** | 533,2736664 | 12,18 | C29H40O9 | C20H27O-C9H13O8 | 1,58E+06 |
| **Nb9** | 531,258255 | 9,47 | C29H38O9 | C20H25O-C9H13O8 | 8,71E+05 |
| **Nb10** | 549,2695618 | 8,54 | C29H40O10 | C20H27O2-C9H13O8 | 1,33E+06 |
| **6** | 313,1803131 | 14,79 | C20H24O3 | triptophenolide | 8,04E+05 |
| **Nb12** | 475,2320557 | 10,08 | C26H34O8 | C20H23O3-C6H11O5 | 8,55E+05 |
| **Nb13** | 621,2913818 | 8,05 | C32H44O12 | C20H21O2-C6H11O5-C6H11O5 | 7,46E+05 |
| **Nb14** | 445,2582779 | 8,34 | C26H36O6 | C20H25O-C6H11O5 | 6,57E+05 |
| **Nb15** | 547,25354 | 8,73 | C29H38O10 | C20H25O2-C9H13O8 | 9,03E+05 |
| **Nb16** | 551,2845459 | 13,05 | C29H42O10 | C20H29O2-C9H13O8 | 9,03E+05 |
| **Nb17** | 463,2685623 | 7,84 | C26H38O7 | C20H27O2-C6H11O5 | 6,67E+05 |
| **Nb18** | 329,1749802 | 13,97 | C20H24O4 |  | 4,77E+05 |
| **Nb19** | 624,2961731 | 11,81 | C30H45N3O9S | C20H29O3-C10H16N3O6S | 5,28E+05 |
| **Nb20** | 445,2579269 | 7,6 | C26H36O6 | C20H25O-C6H11O5 | 6,07E+05 |
| **Nb21** | 317,2112274 | 8,88 | C20H28O3 |  | 3,82E+05 |
| **Nb22** | 624,2960052 | 11,25 | C30H45N3O9S | C20H29O3-C10H16N3O6S | 3,13E+05 |
| **Nb23** | 491,2271957 | 7,72 | C26H34O9 | C20H23O4-C6H11O5 | 4,58E+05 |
| **Nb24** | 529,2433472 | 9,32 | C29H36O9 | C20H23O-C9H13O8 | 3,80E+05 |
| **Nb25** | 549,2693176 | 11,79 | C29H40O10 | C20H27O2-C9H13O8 | 4,90E+05 |
| **Nb26** | 529,2437744 | 11,81 | C29H36O9 | C20H23O-C9H13O8 | 1,90E+05 |
| **Nb27** | 299,2007599 | 13,41 | C20H26O2 |  | 4,11E+05 |
| **Nb28** | 331,1907806 | 6,1 | C20H26O4 |  | 3,29E+05 |
| **Nb29** | 529,2428589 | 9,67 | C29H36O9 | C20H23O-C9H13O8 | 2,20E+05 |
| **Nb30** | 563,2491608 | 9,43 | C29H38O11 | C20H25O3-C9H13O8 | 2,21E+05 |
| **Nb31** | 567,2800293 | 10,73 | C29H42O11 | C20H25O3-C9H13O8 | 3,23E+05 |
| **Nb32** | 666,234024 | 4,8 | C30H39N3O12S | C20H23O7-C10H16N3O6S | 4,13E+05 |
| **Nb33** | 279,1748962 | 8,06 | C20H22O |  | 1,79E+05 |
| **Nb34** | 568,3114624 | 12,34 | C26H47O13 | C20H36O8-C6H11O5 | 2,87E+05 |
| **Nb35** | 577,2280426 | 12,01 | C29H36O12 | C20H23O4-C9H13O8 | 3,47E+05 |
| **Nb36** | 707,2936096 | 8,77 | C30H48N3O14S | C20H32O8-C10H16N3O6S | 1,64E+05 |
| **Nb37** | 585,290802 | 7,16 | C29H44O12 | C20H31O4-C9H13O8 | 1,44E+05 |
| **Nb38** | 563,2500458 | 8,59 | C29H38O11 | C20H25O3-C9H13O8 | 8,73E+04 |
| **Nb39** | 621,2912445 | 7,24 | C32H44O12 | C20H22O2-C6H11O5-C6H11O5 | 2,11E+05 |
| **Nb40** | 295,1695404 | 9,82 | C20H22O2 |  | 3,07E+05 |
| **Nb41** | 547,2528229 | 9,31 | C29H38O10 | C20H25O2-C9H13O8 | 9,25E+04 |
| **Nb42** | 479,2635193 | 7,33 | C26H38O8 | C20H27O3-C6H11O5 | 1,16E+05 |
| **Nb43** | 621,292099 | 9,95 | C32H44O12 | C20H22O2-C6H11O5-C6H11O5 | 2,46E+05 |
| **2** | 359,1487885 | 11,68 | C20H22O6 | triptonide | 6,77E+04 |
| **Nb45** | 563,2492676 | 7,71 | C29H38O11 | C20H25O3-C9H13O8 | 1,18E+05 |
| **Nb46** | 463,2684555 | 7,14 | C26H38O7 | C20H27O2-C6H11O5 | 1,01E+05 |
| **Nb47** | 515,2643433 | 8,71 | C29H38O8 | C20H25-C9H13O8 | 8,68E+04 |
| **Nb48** | 565,2636414 | 9,54 | C29H40O11 | C20H27O3-C9H13O8 | 2,19E+05 |
| **Nb49** | 481,2793732 | 7,83 | C26H40O8 | C20H29O3-C6H11O5 | 7,86E+04 |
| **Nb50** | 525,2346649 | 10,18 | C26H36O11 | C20H25O6-C6H11O5 | 6,25E+04 |
| **Identified in *S. cerevisiae* extracts** | | | | | |
| **CI** | **m/z** | **RT** | **Pred. Formula** | **Compound** | **Peak area (n=1)** |
| **Sc1** | 315,1955414 | 14,9135 | C20H26O3 |  | 1,15E+07 |
| **4** | 287,2374573 | 23,3618 | C20H30O | 14-OH-dehydroabietadiene | 9,10E+06 |
| **Sc3** | 329,1752625 | 13,5563 | C20H24O4 |  | 3,82E+06 |
| **6** | 313,180191 | 14,7948 | C20H24O3 | triptophenolide | 2,21E+06 |
| **Sc5** | 312,196106 | 12,1821 | C20H25NO2 |  | 1,52E+06 |
| **Sc6** | 299,2006226 | 12,0973 | C20H26O2 |  | 1,10E+06 |
| **Sc7** | 269,2267303 | 20,919 | C20H28 |  | 1,02E+06 |
| **Sc8** | 269,2267761 | 21,2074 | C20H28 |  | 5,33E+05 |
| **Sc9** | 356,2226868 | 11,724 | C22H29NO3 | C20H24O2-C2H5NO | 6,05E+05 |
| **Sc10** | 271,2421875 | 19,3243 | C20H30 |  | 4,34E+05 |
| **Sc11** | 319,2273712 | 13,3527 | C20H28O2 |  | 6,29E+05 |
| **Sc12** | 317,2116241 | 17,1359 | C20H28O3 |  | 5,05E+05 |
| **Sc13** | 271,2426758 | 22,0217 | C20H30 |  | 1,79E+05 |
| **Sc14** | 269,2272034 | 21,394 | C20H28 |  | 1,67E+05 |
| **Sc15** | 400,2129974 | 11,4695 | C23H29NO5 | C20H22O4-C3H7NO | 4,47E+05 |
| **Sc16** | 356,2227173 | 13,0643 | C22H29NO3 | C20H24O2-C2H5NO | 3,20E+05 |
| **Sc17** | 314,1875305 | 14,9305 | C20H25O3 |  | 6,56E+05 |
| **Sc18** | 302,2246094 | 15,5922 | C20H29O2 |  | 4,58E+05 |
| **Sc19** | 400,2124329 | 12,4366 | C23H29NO5 | C20H22O4-C3H7NO | 2,03E+05 |
| **Sc20** | 291,2695618 | 23,4636 | C20H34O |  | 2,13E+05 |
| **Sc21** | 283,205719 | 10,8418 | C20H26O |  | 7,91E+04 |
| **Sc22** | 318,2192078 | 12,759 | C20H29O3 |  | 1,79E+05 |
| **Sc23** | 307,2633057 | 18,7136 | C20H34O2 |  | 1,21E+05 |
| **Sc24** | 302,224472 | 16,8984 | C20H29O2 |  | 1,52E+05 |
| **Sc25** | 317,2112732 | 12,3178 | C20H28O3 |  | 5,42E+04 |
| **Sc26** | 346,2741241 | 8,95863 | C22H35NO2 | C20H30O-C2H5NO | 7,60E+04 |
| **Sc27** | 321,2427979 | 18,3234 | C20H32O3 |  | 9,72E+04 |
| **Sc28** | 319,2257385 | 12,742 | C20H30O3 |  | 5,99E+04 |
| **2** | 359,1493225 | 11,6901 | C20H22O6 | triptonide | 4,11E+04 |

**Supplementary table 25 Primer list.** List of primers used in this work. U denotes uracil, enabling USER cloning^27^ of gene-of-interest.

| **Name** | **Sequence** | **Target gene** | **Target vector** |
| --- | --- | --- | --- |
| **TwCYP51G61_USER_F** | GGCTTAAUATGGATACGGATAACAAGTTGTGGAATGTG | TwCYP51G61 | pLIFE33-USER |
| **TwCYP51G61_USER_R** | GGTTTAAUCTAACTAACAGACAGGGCTCGTCG | TwCYP51G61 | pLIFE33-USER |
| **TwCYP706B23_USER_F** | GGCTTAAUATGTTGAGCCAACTAAACTATGAGAAAGGT | TwCYP706B23 | pLIFE33-USER |
| **TwCYP706B23_USER_R** | GGTTTAAUTTAATACTCTGGATTGTGAAGCCGC | TwCYP706B23 | pLIFE33-USER |
| **TwCYP706B24_USER_F** | GGCTTAAUATGTTGAGCCAACTAAACTATGAGAAAGGT | TwCYP706B24 | pLIFE33-USER |
| **TwCYP706B24_USER_R** | GGTTTAAUTTAATACTCTGGATTGTGAAGCCGC | TwCYP706B24 | pLIFE33-USER |
| **TwCYP716C52_USER_F** | GGCTTAAUATGGCATTTTATCAAACACTTTCCGTGGATT | TwCYP716C52 | pLIFE33-USER |
| **TwCYP716C52_USER_R** | GGTTTAAUTTAAGCATAAAGGTGGGCAGGAAGG | TwCYP716C52 | pLIFE33-USER |
| **TwCYP716C57_USER_F** | ATCAACGGGUATGGATCCTCAAGTTCTGGTACCTG | TwCYP716C57 | pLIFE33-USER |
| **TwCYP716C57_USER_R** | CGTGCGAUCTAGTTTGAGTGAAGGCGAATTGGAA | TwCYP716C57 | pLIFE33-USER |
| **TwCYP716E66_USER_F** | GGCTTAAUATGTTAGCTATGTATCTACTCTCTCCCTATCT | TwCYP716E66 | pLIFE33-USER |
| **TwCYP716E66_USER_R** | GGTTTAAUTCAATTATTGTGAGGCTGTATTTGTATTGGAA | TwCYP716E66 | pLIFE33-USER |
| **TwCYP72A768v3_USER_F** | GGCTTAAUATGGAAGCATCAATGGTTTCCATTGC | TwCYP72A768v3 | pLIFE33-USER |
| **TwCYP72A768v3_USER_R** | GGTTTAAUCTAGGTTTCAAGGTTACGAATATTGATTTGAGC | TwCYP72A768v3 | pLIFE33-USER |
| **TwCYP72A768v4_USER_F** | GGCTTAAUATGGAAGCATCAATGGTTTCCATTGC | TwCYP72A768v4 | pLIFE33-USER |
| **TwCYP72A768v4_USER_R** | GGTTTAAUCTAGGTTTCAAGGTTACGAATATTGATTTGAGC | TwCYP72A768v4 | pLIFE33-USER |
| **TwCYP72A768v5_USER_F** | GGCTTAAUATGGAAGCATCAATGGTTTCCATTGC | TwCYP72A768v5 | pLIFE33-USER |
| **TwCYP72A768v5_USER_R** | GGTTTAAUCTAGGTTTCAAGGTTACGAATATTGATTTGAGC | TwCYP72A768v5 | pLIFE33-USER |
| **TwCYP81AM2_USER_F** | ATCAACGGGUATGGACACCCTTTACTACTTAGGTCTCTC | TwCYP81AM2 | pLIFE33-USER |
| **TwCYP81AM2_USER_R** | CGTGCGAUTTAGAGTTGGGAAAGTGCAGCAGT | TwCYP81AM2 | pLIFE33-USER |
| **TwCYP81AN8_USER_F** | GGCTTAAUATGGAAGACAACACTATCTACTACTCCG | TwCYP81AN8 | pLIFE33-USER |
| **TwCYP81AN8_USER_R** | GGTTTAAUTTAGAGAAAAAGCTTGTTCATGATGGGC | TwCYP81AN8 | pLIFE33-USER |
| **TwCYP82AQ1v2_USER_F** | GGCTTAAUATGGATTTTGCGCTACCATTGCCC | TwCYP82AQ1v2 | pLIFE33-USER |
| **TwCYP82AQ1v2_USER_R** | GGTTTAAUTTAAGCATAAAGGTGGGCAGGAAGGC | TwCYP82AQ1v2 | pLIFE33-USER |
| **TwCYP82D213_USER_F** | GGCTTAAUATGGAGTTTCTTCTTTCACTTCCAACAA | TwCYP82D213 | pLIFE33-USER |
| **TwCYP82D213_USER_R** | GGTTTAAUTTACCCCATATAGAGATGACTTGGAAGGCGTGG | TwCYP82D213 | pLIFE33-USER |
| **TwCYP82D217_USER_F** | GGCTTAAUATGGAGTTTCTTCTTTCACTCCCAACA | TwCYP82D217 | pLIFE33-USER |
| **TwCYP82D217_USER_R** | GGTTTAAUGCTATTGCAATTCACCCCATATAGACATG | TwCYP82D217 | pLIFE33-USER |
| **TwCYP82D274_USER_F** | GGCTTAAUATGGAGTTTCTTCTTTCACTCCCAACA | TwCYP82D274 | pLIFE33-USER |
| **TwCYP82D274_USER_R** | GGTTTAAUTCAGCCCATATAGAGATGAGCTGGGA | TwCYP82D274 | pLIFE33-USER |
| **TwCYP82D274v2_USER_F** | GGCTTAAUATGGAGTTTCTTCTTTCACTCCCAACA | TwCYP82D274v2 | pLIFE33-USER |
| **TwCYP82D274v2_USER_R** | GGTTTAAUTCAGCCCATATAGAGATGAGCTGGGA | TwCYP82D274v2 | pLIFE33-USER |
| **TwCYP82D274v3_USER_F** | GGCTTAAUATGGAGTTTCTTCTTTCACTTCCAACAA | TwCYP82D274v3 | pLIFE33-USER |
| **TwCYP82D274v3_USER_R** | GGTTTAAUTCAGCCCATATAGAGATGAGCTGGGA | TwCYP82D274v3 | pLIFE33-USER |
| **TwCYP82D274v4_USER_F** | GGCTTAAUATGGAGTTTCTTCTTTCACTTCCAACAA | TwCYP82D274v4 | pLIFE33-USER |
| **TwCYP82D274v4_USER_R** | GGTTTAAUTCAGCCCATATAGAGATGAGCTGGGA | TwCYP82D274v4 | pLIFE33-USER |
| **TwCYP82D275_USER_F** | GGCTTAAUCTCTCCTAACTAGCTCGATCGCCAA | TwCYP82D275 | pLIFE33-USER |
| **TwCYP82D275_USER_R** | GGTTTAAUGCTATTGCAATTCACCCCATATAGACATG | TwCYP82D275 | pLIFE33-USER |
| **TwPOR1_USER_F** | GGCTTAAUATGCAATCTTCTTCAAATTCTATGAAGG | TwPOR1 | pLIFE33-USER |
| **TwPOR1_USER_R** | GGTTTAAUTTACCACACATCCCGGAGATA | TwPOR1 | pLIFE33-USER |
| **Twcytb5-A_USER_F** | GGCTTAAUATGGCTTCGGATCGGAAGATA | Twcytb5-A | pLIFE33-USER |
| **Twcytb5-A_USER_R** | GGTTTAAUTTAAGCAGGAGGAGCTGATTTGGT | Twcytb5-A | pLIFE33-USER |
| **Twcytb5-B_USER_F** | GGCTTAAUATGGGTGGAGACGGAAAGGTT | Twcytb5-B | pLIFE33-USER |
| **Twcytb5-B_USER_R** | GGTTTAAUTTAAGCAGGAGGAGCTGATTTGGT | Twcytb5-B | pLIFE33-USER |
| **Twcytb5-C_USER_F** | GGCTTAAUATGGCTGGTCAGAGAGTTTTCAC | Twcytb5-C | pLIFE33-USER |
| **Twcytb5-C_USER_R** | GGTTTAAUTTAGAAGATCTGCTCAGGCCTTGTA | Twcytb5-C | pLIFE33-USER |
| **Twcytb5-D_USER_F** | ATCAACGGGUAAAATGGCTAAACTTCTTTCATTTGCTGAG | Twcytb5-D | pLIFE33-USER |
| **Twcytb5-D_USER_R** | CGTGCGAUTTAGAAAAGGTATCGCAAACCAAATGCC | Twcytb5-D | pLIFE33-USER |
| **Twcytb5-E_USER_F** | ATCAACGGGUAAAATGATTATTGTTGCGGTGGCTCTGA | Twcytb5-E | pLIFE33-USER |
| **Twcytb5-E_USER_R** | CGTGCGAUTTACTTCTCTAGATCCCCAATGTAAAAATCATCG | Twcytb5-E | pLIFE33-USER |
| **Twcytb5-F_USER_F** | ATCAACGGGUAAAATGCCGACTTTAACGAAGCTGCAC | Twcytb5-F | pLIFE33-USER |
| **Twcytb5-F_USER_R** | CGTGCGAUCTACTTCTTCCGCAAGTACAGGAGTC | Twcytb5-F | pLIFE33-USER |
| **Primers for building constructs for stable integration of triptonide biosynthetic into the *S. cerevisiae* genome** | | | |
| **CO_TwCYP71BE85_TEF-F** | AGCGATACGUAAAATGGACTTATTGCAATTTCCATCTG | CO_TwCYP71BE85 | pX-3-Ass1-KlURA3 |
| **CO_TwCYP71BE85_TEF-R** | CACGCGAUTCAGTTAAATGCGGGTGATGG | CO_TwCYP71BE85 | pX-3-Ass1-KlURA3 |
| **CO_TwCYP82D213_PGK-F** | ATCAACGGGUAAAATGGAATTCCTTCTGTCATTGC | CO_TwCYP82D213 | pX/pAss |
| **CO_TwCYP82D213_PGK-R** | CGTGCGAUCTAACCCATGTAAAGATGTGATGG | CO_TwCYP82D213 | pX/pAss |
| **CO_TwCYP71BE86_PGK-F** | ATCAACGGGUAAAATGGACTTACAATTACCTAGCTTCC | CO_TwCYP71BE86 | pX/pAss |
| **CO_TwCYP71BE86_PGK-R** | CGTGCGAUTTAACCAGATAAACTACGATATGGG | CO_TwCYP71BE86 | pX/pAss |
| **pLife_TEF-F** | AGCGATACGUTGCAGGCTGAGGCTTAATATG | TwCYP82D274 | pX/pAss |
| **pLife_TEF-R** | CACGCGAUCCCGGGGCTGAGGTTTAAT | TwCYP82D274 | pX/pAss |
| **pLife_PGK-F** | ATCAACGGGUTGCAGGCTGAGGCTTAATATG | TwPOR1 | pX/pAss |
| **pLife_PGK-R** | CGTGCGAUCCCGGGGCTGAGGTTTAAT | TwPOR1 | pX/pAss |
| **pLife_PGK-F** | ATCAACGGGUTGCAGGCTGAGGCTTAATATG | Twcytb5-A | pX/pAss |
| **pLife_PGK-R** | CGTGCGAUCCCGGGGCTGAGGTTTAAT | Twcytb5-A | pX/pAss |
| **Primers for construction of pLIFE33new** | | | |
| **GBA39** | AGCGAAGAGGCCCGCACCGATCGC | New_pLIFE | pLIFE_inPhusion |
| **GBA40** | CGTTAATTCAGTACATTAAAAACGTCCGCAATG | New_pLIFE | pLIFE_inPhusion |
| **GBA41** | TGTACTGAATTAACGGTTACCCGGCCGCCGTGCTGGAC | New_pLIFE | Termi_inPhusion |
| **GBA42** | GCGGGCCTCTTCGCTAAAAGCCTATACTGTACTTAACTTGATTGC | New_pLIFE | Termi_inPhusion |
| **GBA43** | CGTGCGAUTCTAGAGATCCGTCAACATGGTGGAGC | Single promoter TEF | p35S_enh (TEF site, single) |
| **GBA44** | ACGTATCGCUGGTCGTCCTCTCCAAATGAAATGAAC | Single promoter TEF | p35S_enh (TEF site, single) |
| **GBA45** | CACGCGAUTCTAGAGATCCGTCAACATGGTGGAGC | Single promoter PGK | p35S_enh (PGK site, single) |
| **GBA46** | ACCCGTTGAUGGTCGTCCTCTCCAAATGAAATGAAC | Single promoter PGK | p35S_enh (PGK site, single) |
| **GBA47** | AGACTTAGGUTCTAGAGATCCGTCAACATGGTGGAGC | Dual promoter TEF | p35S_enh (TEF site, Dual) |
| **GBA48** | ACCTAAGTCUTCTAGAGATCCGTCAACATGGTGGAGC | Dual promoter PGK | p35S_enh (PGK site, Dual) |
| **GBA49** | CGTGCGAUTTCGTCCACAGACATCAACATCTTATC | Single promoter TEF | pM24 (TEF site, single) |
| **GBA50** | ACGTATCGCUAGATGAGAGATTTCGATTCCGATTTTG | Single promoter TEF | pM24 (TEF site, single) |
| **GBA51** | CACGCGAUTTCGTCCACAGACATCAACATCTTATC | Single promoter PGK | pM24 (PGK site, single) |
| **GBA52** | ACCCGTTGAUAGATGAGAGATTTCGATTCCGATTTTG | Single promoter PGK | pM24 (PGK site, single) |
| **GBA53** | AGACTTAGGUTTCGTCCACAGACATCAACATCTTATC | Dual promoter TEF | pM24 (TEF site, Dual) |
| **GBA54** | ACCTAAGTCUTTCGTCCACAGACATCAACATCTTATC | Dual promoter PGK | pM24 (PGK site, Dual) |
| **GBA55** | CGTGCGAUGTTTTACAGTCAGGACAGATAATGTAAATC | Single promoter TEF | pSGT (TEF site, single) |
| **GBA56** | ACGTATCGCUCACTCCCCCTCTCTAAAAAATTTTG | Single promoter TEF | pSGT (TEF site, single) |
| **GBA57** | CACGCGAUGTTTTACAGTCAGGACAGATAATGTAAATC | Single promoter PGK | pSGT (PGK site, single) |
| **GBA58** | ACCCGTTGAUCACTCCCCCTCTCTAAAAAATTTTG | Single promoter PGK | pSGT (PGK site, single) |
| **GBA59** | AGACTTAGGUGTTTTACAGTCAGGACAGATAATGTAAATC | Dual promoter TEF | pSGT (TEF site, Dual) |
| **GBA60** | ACCTAAGTCUGTTTTACAGTCAGGACAGATAATGTAAATC | Dual promoter PGK | pSGT (PGK site, Dual) |
| **GBA61** | CGTGCGAUGTGGCAGACATACTGTCCCACAAATGAAG | Single promoter TEF | pCm9_11 (TEF site, single) |
| **GBA62** | ACGTATCGCUAAGCTTAGCTCTTACCTGTTTTCGTCG | Single promoter TEF | pCm9_11 (TEF site, single) |
| **GBA63** | CACGCGAUGTGGCAGACATACTGTCCCACAAATGAAG | Single promoter PGK | pCm9_11 (PGK site, single) |
| **GBA64** | ACCCGTTGAUAAGCTTAGCTCTTACCTGTTTTCGTCG | Single promoter PGK | pCm9_11 (PGK site, single) |
| **GBA65** | AGACTTAGGUGTGGCAGACATACTGTCCCACAAATGAAG | Dual promoter TEF | pCm9_11 (TEF site, Dual) |
| **GBA66** | ACCTAAGTCUGTGGCAGACATACTGTCCCACAAATGAAG | Dual promoter PGK | pCm9_11 (PGK site, Dual) |
| **GBA72** | AGCGATACGUAAAatgagtcgagttgcaagtctggatgc | USER TEF | tCfTPS1_WT_TEF |
| **GBA73** | CACGCGAUtcagaccactggttcaaatagtacttgg | USER TEF | tCfTPS1_WT_TEF |
| **GBA74** | ATCAACGGGUAAAatgatcacctctaaatcatctgcagc | USER PGK | tCfTPS3_WT_PGK |
| **GBA75** | CGTGCGAUctagttgctgacacaactcattttttctcc | USER PGK | tCfTPS3_WT_PGK |
| **Genotyping primers for engineeried *S. Cerevisiae* strains** | | | |
| **YEA85** | TCTCAGGTATAGCATGAGGTCGCTCAT | Genotyping | YEA85_UP_Genotyping_Fw |
| **YEA86** | CCTGCAGGACTAGTGCTGAGGCATTAAT | Genotyping | YEA86_DW_Genotyping_Fw |
| **YEA89** | TGACGAATCGTTAGGCACAG | Genotyping_siteX_3 | YEA89_X-3_Genotyping_UP |
| **YEA90** | CCGTGCAATACCAAAATCGAG | Genotyping_siteX_3 | YEA90_X-3_Genotyping_DW |
| **YEA98** | CGAAGAAGGCCTGCAATTC | Genotyping_siteXII_2 | YEA98_XII-2_Genotyping_UP |
| **YEA99** | GGCCCTGATAAGGTTGTTG | Genotyping_siteXII_2 | YEA99_X1I-2_Genotyping_DW |

**Supplementary table 26 List of plasmids generated for engineering *S. cerevisiae.*** pX/pAss Vectors for yeast genome-integration

| **Name** | **Description** | **Source** |
| --- | --- | --- |
| **p320** | pX-4-SI-PpTEF2::TwPOR1-TpTDH3::TwCYP82D274 | This study |
| **pVictor1** | pXI‐2‐Ass1‐pICL1::CO_SpGGPPS7 | This study |
| **pVictor2** | pAss2‐pPGK1::CO_CfTPS3 | This study |
| **pVictor3** | pXI‐2‐Ass3‐pTEF1::CO_CfTPS3 | This study |
| **pCYT85** | pAss2A-PpPGK1::CO_CfTPS1-TpTEF1::CO_CfTPS3 | This study |
| **pTRIP10** | pX-3-Ass3-PpSED1::TwCYP82D274-TpFBA1::Twcytb5-A | This study |
| **pTRIP108** | pAss2C-PpENO2::CO_TwCYP71BE85 | This study |
| **pTRIP110** | pX-3-Ass3-PpSED1::TwCYP82D274 | This study |
| **pTRIP14** | pSIXI-2-PpPGK1::TwPOR1-TpTPI1::Twcytb5-D | This study |
| **pTRIP4** | pAss2B-PpCCW12::CO_TwCYP82D213-TpSED1::CO_TwCYP71BE86 | This study |
| **pTRIP5** | pAss2C-PpENO2::CO_TwCYP71BE85-TpPDC1::CO_TwCYP82D213 | This study |
| **pTRIP50** | pXII-2-Ass1-PpPGK1::CO_TwCYP71BE86-TpTPI1::CO_TwCYP71BE85 | This study |
| **pTRIP52** | pAss2A-PpTDH3::CO_TwCYP82D213-TpSED1::CO_TwCYP82D213 | This study |
| **pTRIP53** | pAss2B-PpPDC1::TwCYP82D274-TpENO2::TwCYP82D274 | This study |
| **pTRIP54** | pAss2C-PpTEF1::TwCYP82D213-TpPGK1::TwCYP82D213 | This study |
| **pTRIP55** | pXII-2-Ass3-PpSED1::Twcytb5-A-TpFBA1::Twcytb5-D | This study |
| **pTRIP7** | pX-3-Ass1-pTEF2::TwPOR1-TpICL1::CO_SpGGPPS7 | This study |
| **pTRIP8** | pX-3-Ass1-pTEF2::TwPOR2-TpICL1::CO_SpGGPPS7 | This study |
| **pTRIP88** | pAss2B-TpSED1::CO_TwCYP71BE86 | This study |
| **pTRIP92** | pX-3-Ass3-PpSED1::TwCYP82D274v3-TpFBA1::Twcytb5-A | This study |
| **pTRIP95** | pX-3-Ass3-PpSED1::TwCYP82D274v4-TpFBA1::Twcytb5-A | This study |
| **pTRIP89** | pAss2C-PpENO2::CO_TwCYP71BE85-TpPDC1::CO_TwCYP82D213v2 | This study |
| **pTRIP3** | pAss2-PpFBA1::Twcytb5-A | This study |
| **pCYT185** | pX-3-Ass1-PpTDH3::CO_TwCYP71BE86-TpCCW12::CO_TwCYP71BE85 | This study |
| **pX-3-Ass3** | pX-3-Ass3 empty | This study |
| **pX-3-Ass1** | pX-3-Ass1 empty | This study |
| **pAss2A** | pAss2A empty | This study |
| **pAss2B** | pAss2B empty | This study |
| **pAss2C** | pAss2C empty | This study |
| **pTRIP149** | pX-3-Ass3-PpSED1::CO_TwCYP82D274-TpFBA1::Twcytb5-C | This study |
| **pTRIP150** | pX-3-Ass3-PpSED1::CO_TwCYP82D274-TpFBA1::Twcytb5-D | This study |
| **pTRIP151** | pX-3-Ass3-PpSED1::CO_TwCYP82D274-TpFBA1::Twcytb5-E | This study |
| **pTRIP152** | pX-3-Ass3-PpSED1::CO_TwCYP82D274-TpFBA1::Twcytb5-F | This study |

**Supplementary table 27** Codon optimized *Tw*CYP genes for *S. cerevisiae* expression

| **Gene** | **Sequence** |
| --- | --- |
| TwCYP71BE85 | ATGGACTTATTGCAATTTCCATCTGTTAGTATTTTGTTGGGTTTCGTTTTTTTTATGTTCATGGT CCTAAAGGTCTGGAAAAGGTTCGAAGCTAATGGTAGTACTTCGAATCTTCCACCGGGTCCATGGA AATTACCAATCATTGGAAACTTACACCAACTAGGCGGTAGTGACCCTCCGCATAGGGCATTAGGT GAATTAGCCAAAAAATATGGACCGTTGATGTTTTTACAATTAGGAGAGATACAAACGTTAGTTGT GTCCAGCGCAGAATACGCGGAAGAGGTTCTTAAGACCCACGACACCGTTTTTGCCTCCAGACCAC AAATGCATTCCCTTGAGATTATGTCGTACGACTATAAGGATATTACTTTTTCTCCGTCCGATGGT TCTTGGAGAAGAAGAAGAAAAATCTGCGTTCAAGAGTTATTATCAGCAAAACGTGTACAATCGTT CAGGTCTACAAGAGAAAAAGAGTTATCGAAATTGATTCAGTGGATCTTCAGCCAGGCTGGTACAA GTATAAATTTGACCACCAAGATATATTCATCAACTTGTACTCTGTCGTCAAGGATGGCTTTTAGC GATGAATGTAAGTATCAAGAAGAATTCATCTCTATTTTAAAGGACCTATTAAAGATCGCATCGGG TTTTAACATAGAAGATATGTTTCCTTCAATGAAATTCTTGCATTTGATATCTGGCGCTTCAAGCA AAATTGAAAAGTTGCACAAACAATTAGATAGAATTGTAGGGAGCATCATCGATGAGCATATAAAT CTAAATACTAGAAAATCAGAAGGTAATGAGGATTTGGTTGACGTTTTATTGAAGTACCATGAACA GGGGGACAGCGAATTTAGTTTGAGCATGGAGGAAATAAAAGCAATTATTTGTGACATCTACTTAG CAGGTACCGAAACAAGTAGCACAACAGTGGACTGGACAATGGCTGAGCTTATCAAGAACCCAAGA GTGATGAAAAAAGCGCAAGCAGAAGTGAGACAAGTTTTCGATTCAAGGGGAAGCGTAGATGAAAC TGGGATCCCTGAGCTGAAATACCTTAAATTAGTTATTAAGGAGACACTTAGGCTGCACCCTCCAG GACCACTATTGTTACCTAGAGAAAACGCAAAGTCATGTGAGATAAATGAATACGTCATCCCGGCC AAAACTCGTGTGATGGTTAACGGTTGGGCAATAGGCAGAGACCCAAAGTATTGGCCTAAAGAACC AGAGAAGTTTTACCCTGAGAGATTCATAGACAATCCAATTGACTATAAAGGTACTAACTTTGAAT ACATCCCATTTGGTGCAGGTCGTCGTATGTGCCCCGGCATGGCGTTCGGTCTAGCCAATGTGGAA TTGCCTTTGTCCCAGTTTTTATATTATTTTGATTGGAAATTAGCCGACGGAATGGTTCCCGAGAA TTTAAATATGGCTGAAGCTTTTGCTGCGACCGTGTGTCGTAAAGACGATTTATACCTAATTCCAA CCCCATATTGCCCATCACCCGCATTTAACTGA |
| TwCYP71BE86 | ATGGACTTACAATTACCTAGCTTCCCAATTTTATCCAGTATTATTCTATTAATATTAGTAGTTTT GAAATCGGTTTTAAGACCATCCAAATTGCCGCCGGGACCATGGAAGCTTCCTCTTATTGGAAACC TGCATCAGCTAGCCCAAGATTTACCGCACAGAGCATTGCAAAAATTGGCCAAAAAACATGGTCCT TTGATGCATTTACATTTTGGTGAAGTTCCAACGCTTGTCGTAACAAGTCCGGAGTACGCAAAGGA AGTCATGAAGACCCATGACATTACTTTTGCGTCGAGGCCGCTTCTTAATGCAATGAAAGTCATGA CTTATGATCATACGGATATAGCCTTCGCTCCTTATGGGGAATATTGGAGACAGCTTAGAAAGATT TGCACCATTGAACTTTTGTCCGTAAAAAGAGTCCAAAGTTTTAGACCGATTAGAGAGCAAGAAAC TAGTAACGTAATTGAATGGATCGGTAGTAACGCAGGATCATCTATTAATCTTACAGAGAGGTTGT ATACTACCATATATGCTTTGGTTTCAAAAGTGGCCTTCGGTAGAACTTGTGGTCGTGGAGAACAC GAGGAGTTTATTGAATACTCAAAAGCAAGTCAGAATAGGGCATCTGGTTTTAATATTGTGGATGT ATTTCCATCATTAAAATTGGTCCATTGGATTATGGGTGAGGGCAAGAAAACTGAAAGGTTGCATA AGCAAGGAGATATGCTTCTAGGAAACATTATAAATCAGCATGTTAAAAAGCCGGTTACCGGAAAG GGAGATGATGAGCATGAGGACTTAGTTGACGTATTATTAAAATTTCACGAAGAAGGTGACTTTCC GTTGACCATCAATAATATAAAGTCAGTTATCCAGGACATTTTTGTGGCAGGGGGGGAGACTAGTG CTACGACCATCGATTGGGCCATGAGAGAAATGATGAAAAACCCCAGAGTTATGAAGAAAGCACAA GCTGAAGTGAGACAAGTTTTTGACTCTAGGGGTAGGGTTGATGAAACCGCTGTTCCTGAACTGAA ATACTTGAAGTTAGTTTTGAAGGAAACGCTGAGGTTACATCCACCTTTACCCTTTCTTTTACCCA GAATAAATTGGGAAAGATGCGAAATTAACGGTTATGAGATTGCTGCTAATACTAAAGTTATCGTG AACGCTTGGGCTATCGGTCGTGACCCCAACTATTGGACGGAGGCCGAGAGATTTTATCCAGAAAG ATTCCTTGAAAAATCAGCCGATTATAAGGGCACGTCATTTGAATACACACCATTTGGGGCTGGGA GAAGATTATGTCCAGGGATGTCATTTGGTTTAGCAAATGTAGAGTTCCCATTATCTCAGTTACTG TATCATTTTGATTGGAATTTGACTGGCGGGATGAAACCAGAGGATCTGAATATGATCGAAAGTTT TGATGTGACCATGAGAGCCAAGGATGATTTACATTTAGTTCCCACCCCATATCGTAGTTTATCTG GTTAA |
| TwCYP82D213 | ATGGAATTCCTTCTGTCATTGCCTACAAACACAATAGCCACTAAAATCTTTGCAGTTTTGTTATT ATATTTGTTCTTAAGAATATTTACTAATGTATTAAAACCGAAGAAGTCAAAAACATCACCTCCAC AAGCAGGCGGGGCCTGGCCACTGATCGGTCACTTACACCTGCTTATTGGCCCACAAGCATCATAT ATTACATTAAGTAAAATGGCAGATAAATATGGACCTATCTTTAAAATCAAATTAGGTGTACATCC TACTTTAGTTATCTCAAATTCTGAAGTTGCGAAAGAATGTCTGACGACACACGATAAGGTGCTGG CTAATAGACCAGCCACAGTTGCAATGGAAATTATGGGCTATAACCACGCTATGTTTGGATGGTCT CCGTATGGTCCATATTGGAGGCAATTGAGAAAATTAGTGACGGTGGAATTGTTATCTAATCAACG TTTAAAGACATTTAAACACATCAGGGAGTCCGAAGTTAAGAATTCACTTAAGGAAATGTATCAGT CTTGGGTTCACAATAAGTCAGGAGATTCAAATCACGTTTCGGTAGATATGACCAGAATTTTTGGT GATATAACTGGTAATTTAATTTACAGAATTGTGGTCGGTAAAGTTTACGCACGTAAAGGAGAGGG TGTTGTTAGATGGAAACAGGTTGTAGGAGATTATATGAAACTTTTAACACATTTCAATGTTGGTG ATGCAATGCCGTTTATGAGGTGGTTTGATTTGGGAGGTTTAGAAAAGGCAATGAAAATAACTTTC AAAGAGCTTGACGGTTACGTGGAAGAGTGGCTGGAAGAACATAAAAAAAAACGTTCCAACAGTGG AGGCCATGGAATAGTTGAAGAAGATTTCATGGATGTCATGTTATCGATCTTTGATGATGGTGGTC AACAAGAATACTGTACAGATAATAGCACCCATACAACGAATAAAGCAATGTGTATGGCTCTAATT CTTGGTGCCTCTGAAACCACAAAAACTACTTTGACCTGGTCTTTGTCGCTGTTATTGAACAATTT AGACGTTTTGAAGAAGGTCAAACAAGAATTGGCTGCACATATAGGTCCAGAAACCTTGGTTACTG AATCAGACGTTAATTCATTGGTATATCTAGACGCAGTAATAACTGAAACTTTAAGACTGTATCCC TTGGGACCTCTTGGTTTGCCACATGAATCGATTGAAGATTGCACAATAGCTGGATATCATGTCCC AGCCAGAACTAGAATTTTATTTAATCTGTGGAAAATCCATCAAGACCCGAGAGTATGGGAAAACC CTCTAGAATTTAAGCCAGAAAGGTTTCTGAAAGAACATAACAATATAGACGTCAGAGGCGGGCAT TTTGAACTACTTCCTTTCGGTTCAGGTAGGCGTATGTGTCCGGGTGTCTCTTTTGCTTTACAAGT TTTAAAGTTGACCCTTGCTAATATGTTACACGGATTCGACTTTGCTACTCCAAATGATGAACCAG TTGATATGACAGAAGTGAATCATATGGCCACTACCAGGGCAACACCATTGGAGACCTTAATTTCT CCAAGGCTTCCATCACATCTTTACATGGGTTAG |

**Supplementary table 28** List of *S. cerevisiae* strain used and generated., include plasmid used for genome integration

| **Name** | **Genotype** | **Plasmid 1** | **Plasmid 2** | **Plasmid 3** | **Plasmid 4** | **Plasmid 5** | **Source** |
| --- | --- | --- | --- | --- | --- | --- | --- |
| **S288c** | MATα, *SUC2, gal2, mal2, mel, flo1, flo8-1, ho, bio1, bio6* |  |  |  |  |  | National Collection of Yeast Cultures (NCYC) |
| **NVJ0** | MATα, *SUC2, gal2, mal2, mel, flo1, flo8-1, ho, bio1, bio6, ura3*∆*::KanMX, XI-2::(pTEF1-CO_CfTPS1/pPGK1-CO_CfTPS3/pICL1-CO_SpGGPPS7/KlURA3)* | pVictor1 | pVictor2 | pVictor3 |  |  | This study |
| **NVJ1-3.5** | MATα, SUC2, gal2, mal2, mel, flo1, flo8-1, ho, bio1, bio6, ura3∆::KanMX, XI-2::(pTEF1-CO_CfTPS1/pPGK1-CO_CfTPS3/pICL1-CO_SpGGPPS7), X-4::(pTDH3-TwCYP82D274/pTEF2-TwPOR1/KlURA3) | 1st int.: pVictor1 2nd int.: p320 | 1st int.: pVictor2 | 1st int.: pVictor3 |  |  | This study |
| **NVJ11-0** | MATα, *SUC2, gal2, mal2, mel, flo1, flo8-1, ho, bio1, bio6, ura3*∆*::KanMX, X-3::(KlURA3)* | X3-Ass1 | Ass2A | Ass2B | Ass2C | X3-Ass3 | This study |
| **NVJ11-1** | MATα, SUC2, gal2, mal2, mel, flo1, flo8-1, ho, bio1, bio6, ura3∆::KanMX, X-3::(pTEF2-TwPOR1/pICL1-CO_SpGGPPS7/pPGK1-CO_CfTPS1/pTEF1-CO_CfTPS3/KlURA3) | pTRIP7 | pCYT85 | Ass2B | Ass2C | X3-Ass3 | This study |
| **NVJ11-2** | MATα, SUC2, gal2, mal2, mel, flo1, flo8-1, ho, bio1, bio6, ura3∆::KanMX, X-3::(pTEF2-TwPOR1/pICL1-CO_SpGGPPS7/pPGK1-CO_CfTPS1/pTEF1-CO_CfTPS3/pSED1-TwCYP82D274/KlURA3) | pTRIP7 | pCYT85 | Ass2B | Ass2C | pTRIP110 | This study |
| **NVJ11-3** | MATα, SUC2, gal2, mal2, mel, flo1, flo8-1, ho, bio1, bio6, ura3∆::KanMX, X-3::(pTEF2-TwPOR1/pICL1-CO_SpGGPPS7/pPGK1-CO_CfTPS1/pTEF1-CO_CfTPS3/pENO2-CO_TwCYP71BE85/pSED1-TwCYP82D274/KlURA3) | pTRIP7 | pCYT85 | Ass2B | pTRIP108 | pTRIP110 | This study |
| **NVJ11-4** | MATα, SUC2, gal2, mal2, mel, flo1, flo8-1, ho, bio1, bio6, ura3∆::KanMX, X-3::(pTEF2-TwPOR1/pICL1-CO_SpGGPPS7/pPGK1-CO_CfTPS1/pTEF1-CO_CfTPS3/pSED1-CO_TwCYP71BE86/pSED1-TwCYP82D274/KlURA3) | pTRIP7 | pCYT85 | pTRIP88 | Ass2C | pTRIP110 | This study |
| **NVJ11-5** | MATα, SUC2, gal2, mal2, mel, flo1, flo8-1, ho, bio1, bio6, ura3∆::KanMX, X-3::(pTEF2-TwPOR1/pICL1-CO_SpGGPPS7/pPGK1-CO_CfTPS1/pTEF1-CO_CfTPS3/pSED1-CO_TwCYP71BE86/pENO2-CO_TwCYP71BE85/pSED1-TwCYP82D274/KlURA3) | pTRIP7 | pCYT85 | pTRIP88 | pTRIP108 | pTRIP110 | This study |
| **NVJ11-6** | MATα, SUC2, gal2, mal2, mel, flo1, flo8-1, ho, bio1, bio6, ura3∆::KanMX, X-3::(pTEF2-TwPOR1/pICL1-CO_SpGGPPS7/pPGK1-CO_CfTPS1/pTEF1-CO_CfTPS3/pSED1-CO_TwCYP71BE86/pENO2-CO_TwCYP71BE85/pPDC1-CO_TwCYP82D213/pSED1-TwCYP82D274/KlURA3) | pTRIP7 | pCYT85 | pTRIP88 | pTRIP5 | pTRIP110 | This study |
| **NVJ11-7** | MATα, SUC2, gal2, mal2, mel, flo1, flo8-1, ho, bio1, bio6, ura3∆::KanMX, X-3::(pTEF2-TwPOR1/pICL1-CO_SpGGPPS7/pPGK1-CO_CfTPS1/pTEF1-CO_CfTPS3/pSED1-TwCYP82D274/pFBA1-Twcytb5-A/KlURA3) | pTRIP7 | pCYT85 | Ass2B | Ass2C | pTRIP10 | This study |
| **NVJ11-8** | MATα, SUC2, gal2, mal2, mel, flo1, flo8-1, ho, bio1, bio6, ura3∆::KanMX, X-3::(pTEF2-TwPOR1/pICL1-CO_SpGGPPS7/pPGK1-CO_CfTPS1/pTEF1-CO_CfTPS3/ pENO2-CO_TwCYP71BE85/pSED1-TwCYP82D274/pFBA1-Twcytb5-A/KlURA3) | pTRIP7 | pCYT85 | Ass2B | pTRIP108 | pTRIP10 | This study |
| **NVJ11-9** | MATα, SUC2, gal2, mal2, mel, flo1, flo8-1, ho, bio1, bio6, ura3∆::KanMX, X-3::(pTEF2-TwPOR1/pICL1-CO_SpGGPPS7/pPGK1-CO_CfTPS1/pTEF1-CO_CfTPS3/pSED1-CO_TwCYP71BE86/pSED1-TwCYP82D274/pFBA1-Twcytb5-A/KlURA3) | pTRIP7 | pCYT85 | pTRIP88 | Ass2C | pTRIP10 | This study |
| **NVJ11-10** | MATα, SUC2, gal2, mal2, mel, flo1, flo8-1, ho, bio1, bio6, ura3∆::KanMX, X-3::(pTEF2-TwPOR1/pICL1-CO_SpGGPPS7/pPGK1-CO_CfTPS1/pTEF1-CO_CfTPS3/pSED1-CO_TwCYP71BE86/pENO2-CO_TwCYP71BE85/pSED1-TwCYP82D274/pFBA1-Twcytb5-A/KlURA3) | pTRIP7 | pCYT85 | pTRIP88 | pTRIP108 | pTRIP10 | This study |
| **NVJ11-11** | MATα, SUC2, gal2, mal2, mel, flo1, flo8-1, ho, bio1, bio6, ura3∆::KanMX, X-3::(pTEF2-TwPOR1/pICL1-CO_SpGGPPS7/pPGK1-CO_CfTPS1/pTEF1-CO_CfTPS3/pSED1-CO_TwCYP71BE86/pENO2-CO_TwCYP71BE85/pPDC1-CO_TwCYP82D213/pSED1-TwCYP82D274/pFBA1-Twcytb5-A/KlURA3) | pTRIP7 | pCYT85 | pTRIP88 | pTRIP5 | pTRIP10 | This study |
| **NVJ8-15** | MATα, SUC2, gal2, mal2, mel, flo1, flo8-1, ho, bio1, bio6, ura3∆::KanMX, X-3::(pTEF2-TwPOR2/pICL1-CO_SpGGPPS7/pPGK1-CO_CfTPS1/pTEF1-CO_CfTPS3/pCCW12-CO_TwCYP82D213/pSED1-CO_TwCYP71BE86/pENO2-CO_TwCYP71BE85/pPDC1-TwCYP82D213/pSED1-TwCYP82D274/pFBA1-Twcytb5-A) XI-2::(pPGK1-TwPOR1/pTPI1-Twcytb5-D) XII-2::(pPGK1-CO_TwCYP71BE86/pTPI1-TwCYP71BE85/pTDH3-CO_TwCYP82D213/pSED1-CO_TwCYP82D213/pPDC1-TwCYP82D274/pENO2-TwCYP82D274/pTEF1-TwCYP82D213/pPGK1-TwCYP82D213/pSED1-Twcytb5-A/pFBA1-Twcytb5-D/KlURA3) | 1st int.: pJAR8 2nd int.: pTRIP14 3rd int.: pTRIP50 | 1st int.: pCYT85 3rd int.: pTRIP52 | 1st int.: pJAR4 3rd int.: pTRIP53 | 1st int.: pJAR5 3rd int.: pTRIP54 | 1st int.: pJAR10 3rd int.: pTRIP55 | This study |
| **NVJ18-0** | MATα, *SUC2, gal2, mal2, mel, flo1, flo8-1, ho, bio1, bio6, ura3*∆*::KanMX, X-3::(KlURA3)* | X3-Ass1 | Ass2A | Ass2B | Ass2C | X3-Ass3 | This study |
| **NVJ18-1** | MATα, SUC2, gal2, mal2, mel, flo1, flo8-1, ho, bio1, bio6, ura3∆::KanMX, X-3::(pTEF2-TwPOR1/pICL1-CO_SpGGPPS7/pPGK1-CO_CfTPS1/pTEF1-CO_CfTPS3/pSED1-CO_TwCYP71BE86/pENO2-CO_TwCYP71BE85/pSED1-CO_TwCYP82D274/pFBA1-Twcytb5-A/KlURA3) | pTRIP7 | pCYT85 | pTRIP88 | pTRIP108 | pTRIP10 | This study |
| **NVJ18-3** | MATα, SUC2, gal2, mal2, mel, flo1, flo8-1, ho, bio1, bio6, ura3∆::KanMX, X-3::(pTEF2-TwPOR1/pICL1-CO_SpGGPPS7/pPGK1-CO_CfTPS1/pTEF1-CO_CfTPS3/pSED1-CO_TwCYP71BE86/pENO2-CO_TwCYP71BE85/pSED1-CO_TwCYP82D274/pFBA1-Twcytb5-C/KlURA3) | pTRIP7 | pCYT85 | pTRIP88 | pTRIP108 | pTrip 149 | This study |
| **NVJ18-4** | MATα, SUC2, gal2, mal2, mel, flo1, flo8-1, ho, bio1, bio6, ura3∆::KanMX, X-3::(pTEF2-TwPOR1/pICL1-CO_SpGGPPS7/pPGK1-CO_CfTPS1/pTEF1-CO_CfTPS3/pSED1-CO_TwCYP71BE86/pENO2-CO_TwCYP71BE85/pSED1-CO_TwCYP82D274/pFBA1-Twcytb5-D/KlURA3) | pTRIP7 | pCYT85 | pTRIP88 | pTRIP108 | pTrip 150 | This study |
| **NVJ18-5** | MATα, SUC2, gal2, mal2, mel, flo1, flo8-1, ho, bio1, bio6, ura3∆::KanMX, X-3::(pTEF2-TwPOR1/pICL1-CO_SpGGPPS7/pPGK1-CO_CfTPS1/pTEF1-CO_CfTPS3/pSED1-CO_TwCYP71BE86/pENO2-CO_TwCYP71BE85/pSED1-CO_TwCYP82D274/pFBA1-Twcytb5-EKlURA3) | pTRIP7 | pCYT85 | pTRIP88 | pTRIP108 | pTrip 151 | This study |
| **NVJ18-6** | MATα, SUC2, gal2, mal2, mel, flo1, flo8-1, ho, bio1, bio6, ura3∆::KanMX, X-3::(pTEF2-TwPOR1/pICL1-CO_SpGGPPS7/pPGK1-CO_CfTPS1/pTEF1-CO_CfTPS3/pSED1-CO_TwCYP71BE86/pENO2-CO_TwCYP71BE85/pSED1-CO_TwCYP82D274/pFBA1-Twcytb5-F/KlURA3) | pTRIP7 | pCYT85 | pTRIP88 | pTRIP108 | pTrip 152 | This study |
| **NVJ18-7** | MATα, SUC2, gal2, mal2, mel, flo1, flo8-1, ho, bio1, bio6, ura3∆::KanMX, X-3::(pTEF2-TwPOR1/pICL1-CO_SpGGPPS7/pPGK1-CO_CfTPS1/pTEF1-CO_CfTPS3/pSED1-CO_TwCYP71BE86/pENO2-CO_TwCYP71BE85/pSED1-CO_TwCYP82D274/KlURA3) | pTRIP7 | pCYT85 | pTRIP88 | pTRIP108 | pTRIP110 | This study |

**Supplementary references**

1. Hansen, N.L. et al. The terpene synthase gene family in Tripterygium wilfordii harbors a labdane-type diterpene synthase among the monoterpene synthase TPS-b subfamily. *The Plant Journal* **89**, 429-441 (2017).

2. Kruijff, B.d. Lipid polymorphism and biomembrane function. *Current Opinion in Chemical Biology* **1**, 564-569 (1997).

3. Kennedy, E.P. & Weiss, S.B. THE FUNCTION OF CYTIDINE COENZYMES IN THE BIOSYNTHESIS OF PHOSPHOLIPIDES. *Journal of Biological Chemistry* **222**, 193-214 (1956).

4. Kim, K., Kim, K.-H., Storey, M.K., Voelker, D.R. & Carman, G.M. Isolation and Characterization of the Saccharomyces cerevisiae EKI1 Gene Encoding Ethanolamine Kinase*. *Journal of Biological Chemistry* **274**, 14857-14866 (1999).

5. Peters, R.J. Two rings in them all: The labdane-related diterpenoids. *Natural Product Reports* **27**, 1521-1530 (2010).

6. Pateraki, I. et al. Manoyl Oxide (13R), the Biosynthetic Precursor of Forskolin, Is Synthesized in Specialized Root Cork Cells in Coleus forskohlii. *Plant Physiology* **164**, 1222-1236 (2014).

7. Bathe, U. & Tissier, A. Cytochrome P450 enzymes: A driving force of plant diterpene diversity. *Phytochemistry* **161**, 149-162 (2019).

8. Zhan, C. et al. Selection of a subspecies-specific diterpene gene cluster implicated in rice disease resistance. *Nature Plants* **6**, 1447-1454 (2020).

9. Ding, Y. et al. Multiple genes recruited from hormone pathways partition maize diterpenoid defences. *Nature Plants* **5**, 1043-1056 (2019).

10. Nonhebel, D.C., Walton, J.C. & Nonhebel, D.C. *Free-radical chemistry : structure and mechanism*, (Cambridge University Press, London, 1974).

11. Brooks, M.A. & Scott, L.T. 1,2-Shifts of Hydrogen Atoms in Aryl Radicals. *Journal of the American Chemical Society* **121**, 5444-5449 (1999).

12. Kutney, J.P. & Han, K. Studies with plant-cell cultures of the Chinese herbal plant, Tripterygium wilfordii. Isolation and characterization of diterpenes. *Recueil des Travaux Chimiques des Pays-Bas* **115**, 77-93 (1996).

13. Takaishi, Y. et al. Phenolic diterpenes from Tripterygium wilfordii var. regelii. *Phytochemistry* **45**, 979-984 (1997).

14. Gao, C., Wang, D., Zhang, Y., Huang, X.-X. & Song, S.-J. Kaurane and abietane diterpenoids from the roots of Tripterygium wilfordii and their cytotoxic evaluation. *Bioorganic & Medicinal Chemistry Letters* **26**, 2942-2946 (2016).

15. Duan, H., Kawazoe, K., Bando, M., Kido, M. & Takaishi, Y. Di- and triterpenoids from Tripterygium hypoglaucum. *Phytochemistry* **46**, 535-543 (1997).

16. Li, K., Duan, H., Kawazoe, K. & Takaishi, Y. Terpenoids from Tripterygium wilfordii. *Phytochemistry* **45**, 791-796 (1997).

17. Chen, H.-L. et al. Terpenoids Induce Cell Cycle Arrest and Apoptosis from the Stems of Celastrus kusanoi Associated with Reactive Oxygen Species. *Journal of Agricultural and Food Chemistry* **58**, 3808-3812 (2010).

18. Shishido, K. et al. Tripterygium wilfordii var. regelii which are interleukin-1 inhibitors. *Phytochemistry* **35**, 731-737 (1994).

19. Mors, W.B., Fo., M.F.d.S., Monteiro, H.J., Gilbert, B. & Pellegrino, J. Chemoprophylactic Agent in Schistosomiasis: 14,15-Epoxygeranylgeraniol. *Science* **157**, 950-951 (1967).

20. Gnanadesikan, V. & Corey, E.J. A Strategy for Position-Selective Epoxidation of Polyprenols. *Journal of the American Chemical Society* **130**, 8089-8093 (2008).

21. Chen, Y., Li, F. & Wurtzel, E.T. Isolation and Characterization of the <em>Z-ISO</em> Gene Encoding a Missing Component of Carotenoid Biosynthesis in Plants. *Plant Physiology* **153**, 66-79 (2010).

22. Nelson, D. & Werck-Reichhart, D. A P450-centric view of plant evolution. *The Plant Journal* **66**, 194-211 (2011).

23. Hansen, N.L. et al. Integrating pathway elucidation with yeast engineering to produce polpunonic acid the precursor of the anti-obesity agent celastrol. *Microbial Cell Factories* **19**, 15 (2020).

24. Wang, J. et al. A cytochrome P450 CYP81AM1 from Tripterygium wilfordii catalyses the C-15 hydroxylation of dehydroabietic acid. *Planta* **254**, 95 (2021).

25. Ma, Y. et al. Expansion within the CYP71D subfamily drives the heterocyclization of tanshinones synthesis in Salvia miltiorrhiza. *Nature Communications* **12**, 685 (2021).

26. Tu, L. et al. Genome of Tripterygium wilfordii and identification of cytochrome P450 involved in triptolide biosynthesis. *Nature Communications* **11**, 971 (2020).

27. Nour-Eldin, H.H., Hansen, B.G., Nørholm, M.H.H., Jensen, J.K. & Halkier, B.A. Advancing uracil-excision based cloning towards an ideal technique for cloning PCR fragments. *Nucleic Acids Research* **34**, e122 (2006).
