# Supplementary material for: Tripterygium wilfordii cytochrome P450s catalyze the methyl shift and epoxidations in the biosynthesis of triptonide
Source: Nat Commun. 2022 Aug 25;13:5011. doi: 10.1038/s41467-022-32667-5 (PMC9411204; doi:10.1038/s41467-022-32667-5)
Supplement: Supplementary file 2 — Reporting Summary [file 41467_2022_32667_MOESM2_ESM.pdf]

nature portfolio

Corresponding author(s): Johan Andersen-Ranberg

Last updated by author(s): Aug 5, 2022

Reporting Summary

Nature Portfolio wishes to improve the reproducibility of the work that we publish. This form provides structure for consistency and transparency in reporting. For further information on Nature Portfolio policies, see our Editorial Policies and the Editorial Policy Checklist.

Statistics

For all statistical analyses, confirm that the following items are present in the figure legend, table legend, main text, or Methods section.

n/a	Confirmed	
☐	☒	The exact sample size (*n*) for each experimental group/condition, given as a discrete number and unit of measurement
☐	☒	A statement on whether measurements were taken from distinct samples or whether the same sample was measured repeatedly
☒	☐	The statistical test(s) used AND whether they are one- or two-sided<br>*Only common tests should be described solely by name; describe more complex techniques in the Methods section.*
☒	☐	A description of all covariates tested
☒	☐	A description of any assumptions or corrections, such as tests of normality and adjustment for multiple comparisons
☐	☒	A full description of the statistical parameters including central tendency (e.g. means) or other basic estimates (e.g. regression coefficient) AND variation (e.g. standard deviation) or associated estimates of uncertainty (e.g. confidence intervals)
☒	☐	For null hypothesis testing, the test statistic (e.g. *F*, *t*, *r*) with confidence intervals, effect sizes, degrees of freedom and *P* value noted<br>*Give P values as exact values whenever suitable.*
☒	☐	For Bayesian analysis, information on the choice of priors and Markov chain Monte Carlo settings
☒	☐	For hierarchical and complex designs, identification of the appropriate level for tests and full reporting of outcomes
☒	☐	Estimates of effect sizes (e.g. Cohen's *d*, Pearson's *r*), indicating how they were calculated

Our web collection on statistics for biologists contains articles on many of the points above.

Software and code

Policy information about availability of computer code

Data collection	Topspin ver. 3.2 or 3.5 and IconNMR ver. 4.7.5 or 5.0.7 (Bruker Biospin, Karlsruhe, Germany), Bruker Daltonics HyStar v.3.2 SR4 (Bruker, Karlsruhe, Germany)
Data analysis	Microsoft Excel for Mac Ver 16.59 (Microsoft inc.), Mzmine ver2.53, DataAnalysis 4.3 (Bruker, , Karlsruhe, Germany), CLC Genomics Workbench version 8.5.1, SigmaPlot Version 14.5 (Systat Software Inc.)

For manuscripts utilizing custom algorithms or software that are central to the research but not yet described in published literature, software must be made available to editors and reviewers. We strongly encourage code deposition in a community repository (e.g. GitHub). See the Nature Portfolio guidelines for submitting code & software for further information.

Data

Policy information about availability of data

All manuscripts must include a data availability statement. This statement should provide the following information, where applicable:

- Accession codes, unique identifiers, or web links for publicly available datasets
- A description of any restrictions on data availability
- For clinical datasets or third party data, please ensure that the statement adheres to our policy

All data used including gene sequences are available in publicly available databases. cDNA libraries used: https://www.ncbi.nlm.nih.gov/sra/SRR3583049/, https://www.ncbi.nlm.nih.gov/sra/?term=SRR708388, https://www.ncbi.nlm.nih.gov/sra/?term=SRR1171189, and https://www.ncbi.nlm.nih.gov/sra/?term=SRR4294733. T. wilfordii genome: https://www.ncbi.nlm.nih.gov/assembly/GCF_013401445.1/

Field-specific reporting

Please select the one below that is the best fit for your research. If you are not sure, read the appropriate sections before making your selection.

☒ Life sciences ☐ Behavioural & social sciences ☐ Ecological, evolutionary & environmental sciences

For a reference copy of the document with all sections, see nature.com/documents/nr-reporting-summary-flat.pdf

Life sciences study design

All studies must disclose on these points even when the disclosure is negative.

Sample size	Predetermination of sample size was not used in this work. Please refer to the section "replication" below for how samples were allocated and analyzed.
Data exclusions	No data was excluded in the study
Replication	Relative quantification of metabolomics data from heterolgous expression host was based on 3 and 4 biological replicates represented by individual transformants/lines of S. cerevisiae and N. benthamiana, respectively. The usual practice in the field is 3 biological replicates, and we consider the used sample size adequate to extract valid conclusions. As commented in the manuscript the compound levels from N. benthamiana was more variable than compounds levels in S. cerevisiae extracts. Including all replicates in the analysis allowed us to show this discrepancy between these two systems.
Randomization	Not relevant to this study. Based LC-MS analysis of blank/ref samples in between extract samples, it was observed that carryover between samples analyzed by LC-MS did not occur. Thus our experimental setup for LC-MS analysis did not require randomization of the sample analysis order.
Blinding	Blinding was not relevant to this study. No manual peak area determination was used. Peak area determined by the built-in algorithm in MZmine 2.53

Reporting for specific materials, systems and methods

We require information from authors about some types of materials, experimental systems and methods used in many studies. Here, indicate whether each material, system or method listed is relevant to your study. If you are not sure if a list item applies to your research, read the appropriate section before selecting a response.

Materials & experimental systems

n/a	Involved in the study	
☒	☐	Antibodies
☒	☐	Eukaryotic cell lines
☒	☐	Palaeontology and archaeology
☒	☐	Animals and other organisms
☒	☐	Human research participants
☒	☐	Clinical data
☒	☐	Dual use research of concern

Methods

n/a	Involved in the study	
☒	☐	ChIP-seq
☒	☐	Flow cytometry
☒	☐	MRI-based neuroimaging
