# Supplementary material for: Author Correction: Tripterygium wilfordii cytochrome P450s catalyze the methyl shift and epoxidations in the biosynthesis of triptonide
Source: Nat Commun. 2025 Jul 24;16:6811. doi: 10.1038/s41467-025-62209-8 (PMC12290090; doi:10.1038/s41467-025-62209-8)
Supplement: Supplementary file 1 — Supplementary Data 1.1–1.5 [file 41467_2025_62209_MOESM1_ESM.pdf]

Correction Supplementary Data 1.1. ^{1}H NMR spectrum (599.58 MHz, $CDCl_3$) of triptonide (**2**). Ref.: Fang et al. 2022

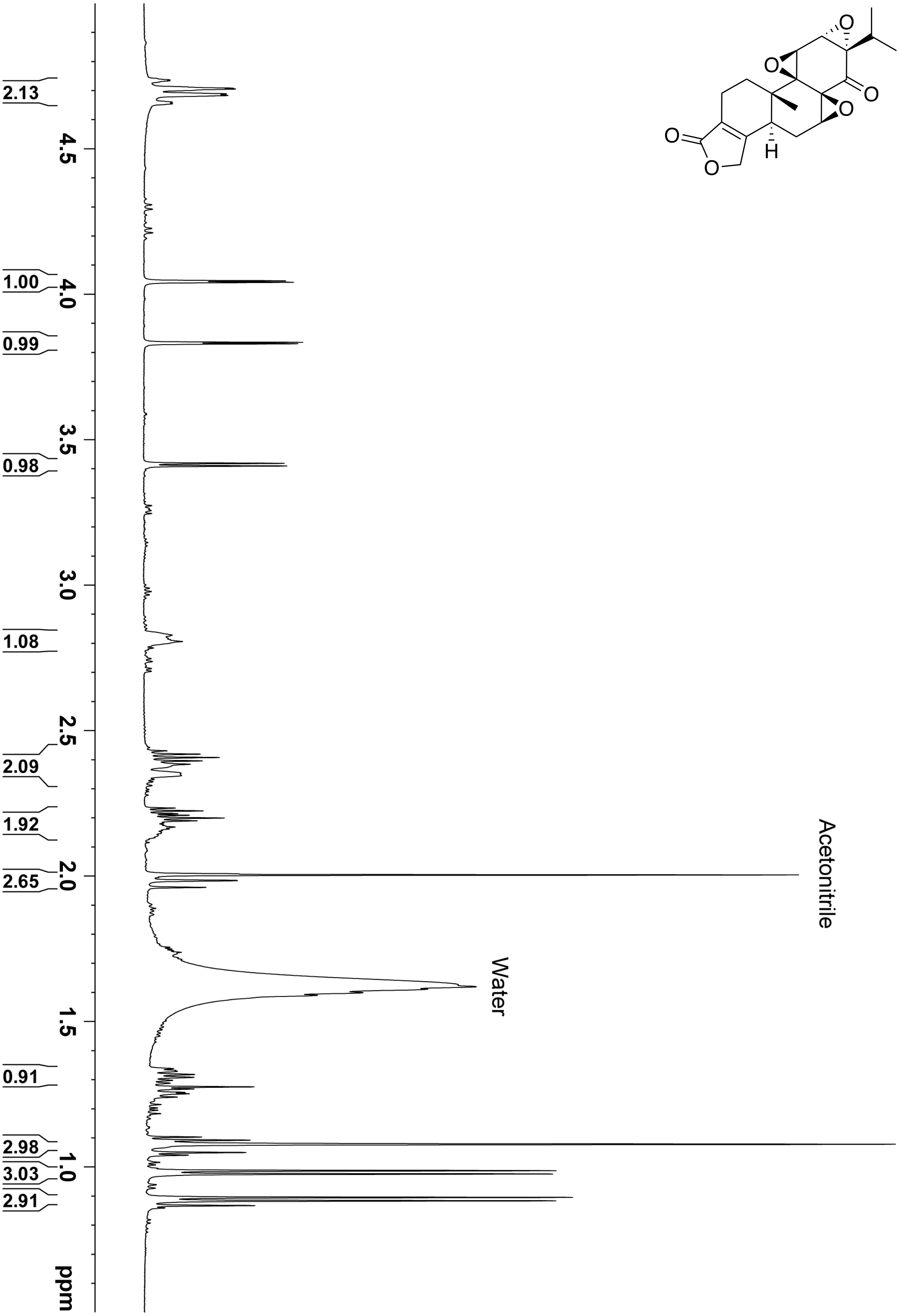

Correction Supplementary Data 1.2. ^{13}C NMR spectrum (150.76 MHz, $CDCl_3$) of triptonide (2). Ref.: Fang et al. 2022

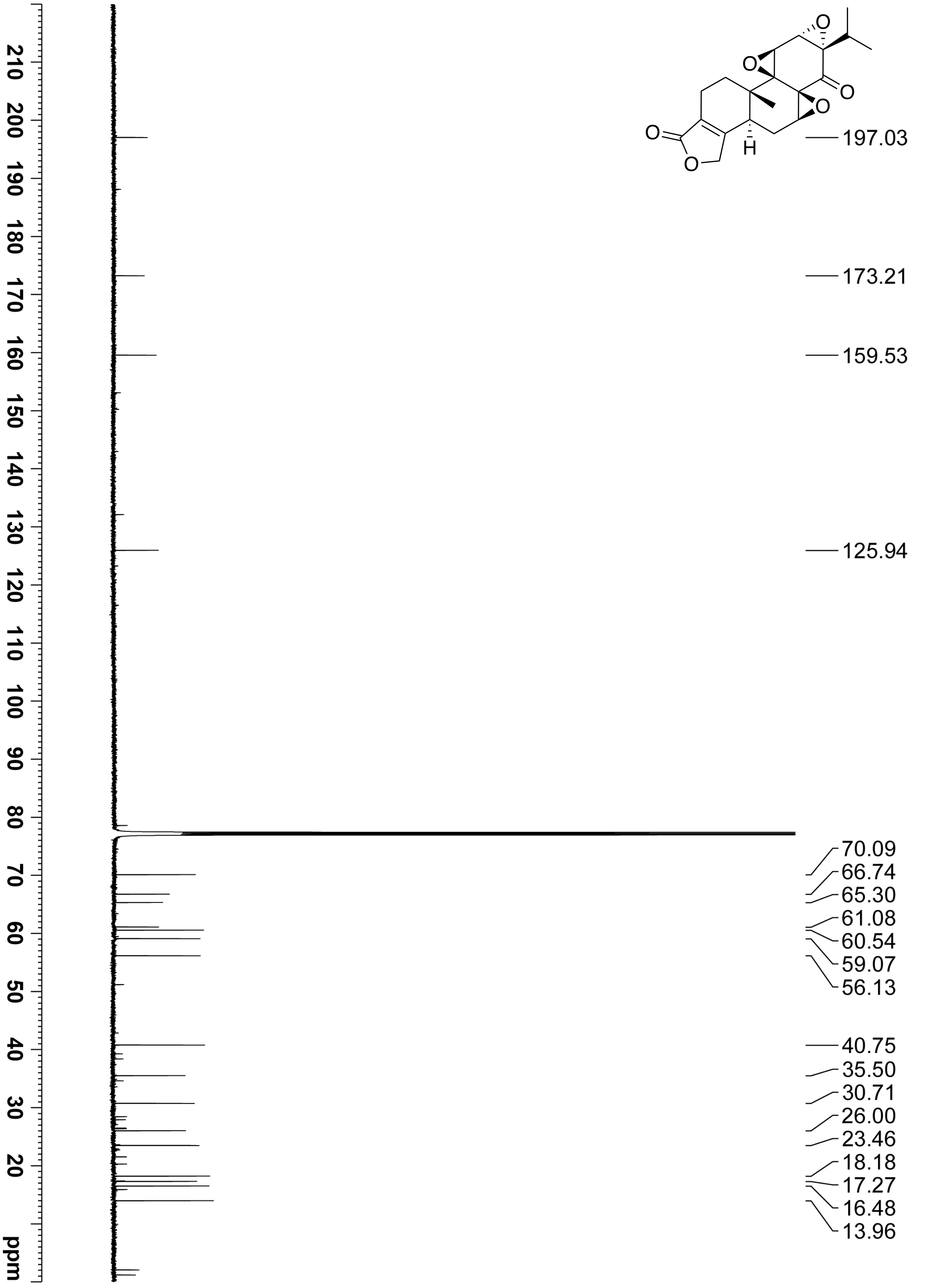

Correction Supplementary Data 1.3. HMBC spectrum of **2**

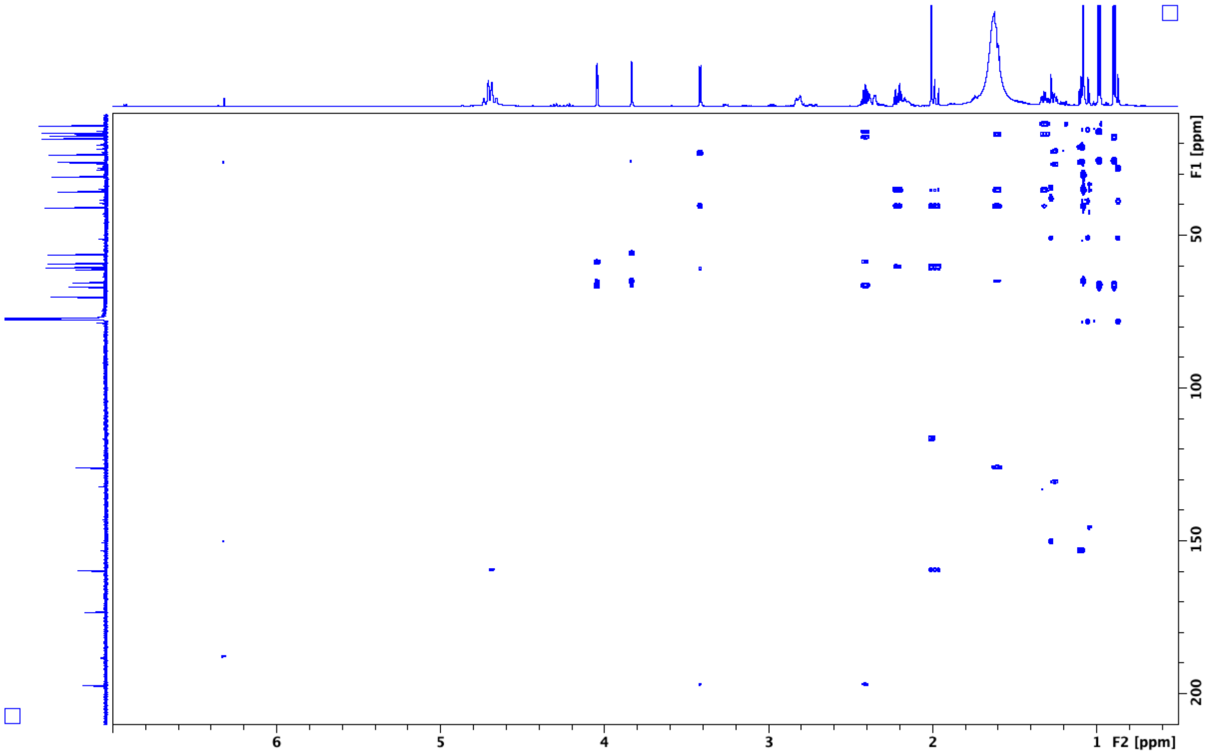

Correction Supplementary Data 1.4 HSQC and ROESY spectra of **2**

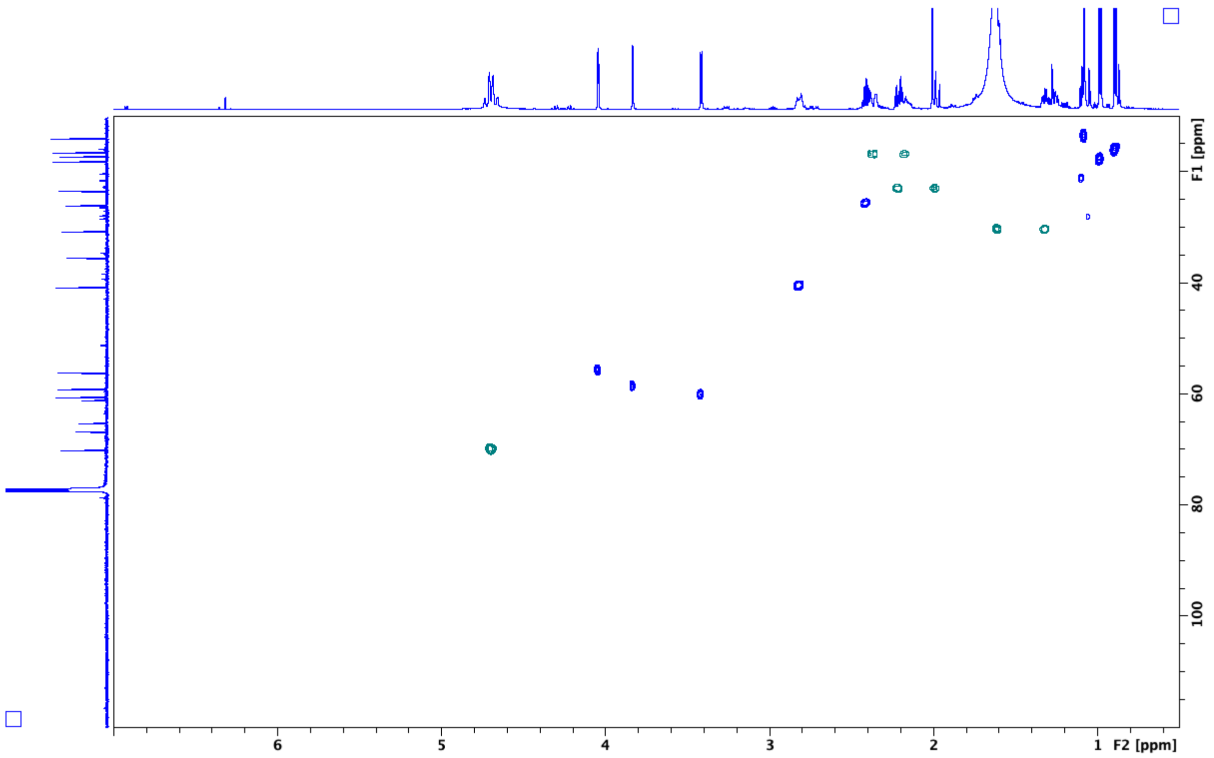

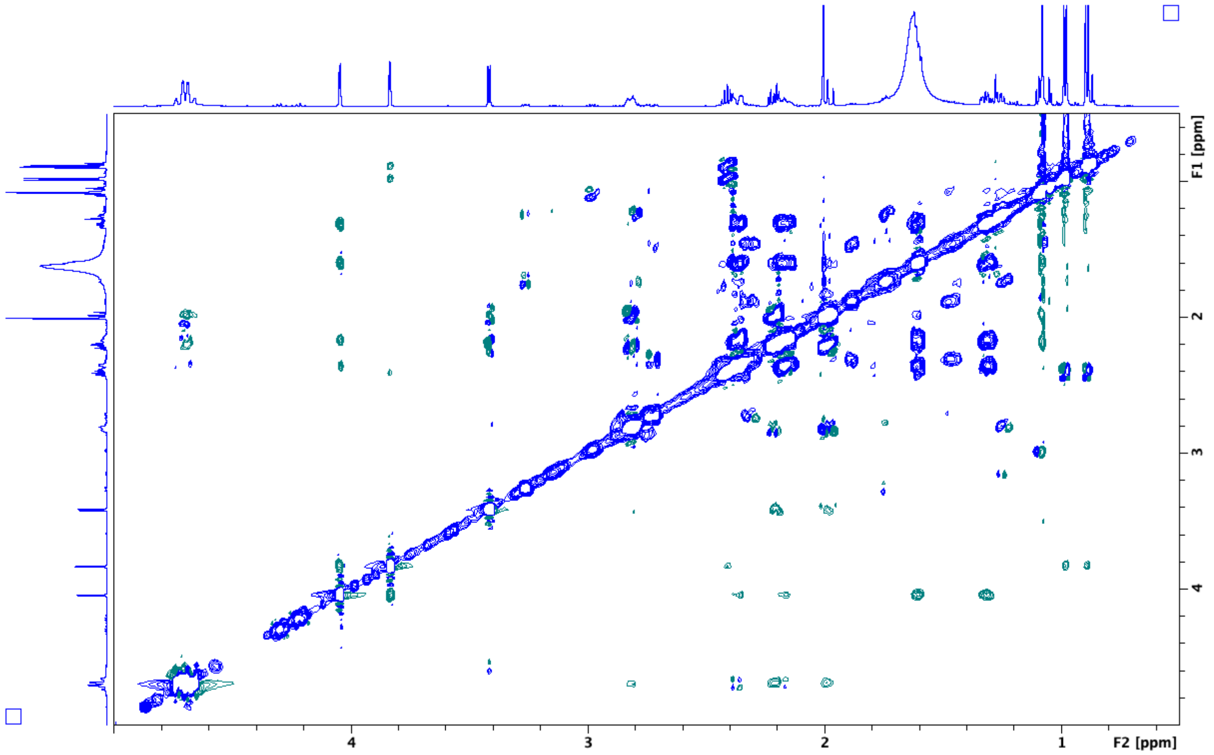

Correction Supplementary Data 1.5 ^{1}H and ^{13}C NMR data and 2D HMBC and ROESY correlations for 2

Pos.	δ_C, type[a,b]	δ_H, nH, multiplicity (*J* in Hz)[a,c]	HMBC	ROESY
1	30.7, CH_2	α: 1.31 (1H, m)	C-2, C-5, C-10, C-20	H-5, H-11
		β:1.61 (1H, m)	C-2, C-3, C-5, C-9, C-10, C-18	H-11, H-20
2	17.3, CH_2	α: 2.37 (1H, m)	C-3, C-4, C-10	H-11
		β: 2.17 (1H, m)		H-11, H-20
3	125.9, C	-		
4	159.5, C	-		
5	40.7, CH	2.82 (1H, m)		H-1α, H-7, H-11, H-19
6	23.5, CH_2	α: 2.21 (1H, dt, 14.5, 5.7)	C-4, C-5, C-8, C-10	H-7, H-19
		β: 1.98 (1H, dd, 14.5, 13.5)	C-4, C-5, C-8, C-10	H-7, H-19, H-20
7	60.5, CH	3.41 (1H, d, 5.4)	C-4, C-5, C-6, C-8, C-9, C-14	H-5, H-6α, H-6β
8	61.1, C	-		
9	65.3, C	-		
10	35.5, C	-		
11	56.1, CH	4.04 (1H, d, 2.9)	C-8, C-9, C-10, C-12, C-13	H-1α, H-1β, H-2α, H-2β, H-5
12	59.1, CH	3.83 (1H, d, 2.9)	C-9, C-11, C-13, C-14, C-15	H-15, H-16, H-17
13	66.7, C	-		
14	197.0, C	-		
15	26.0, CH	2.41 (1H, sep, 6.9)	C-12, C-13, C-14, C-16, C-17	H-12
16	16.5, CH_3	0.89 (3H, d, 6.9)	C-13, C-15, C-17	H-12
17	18.2, CH_3	0.98 (3H, d, 6.9)	C-13, C-15, C-16	H-12
18	173.2 C	-		
19	70.1, CH_2	4.70 (2H, m)	C-3, C-4, C-18	H-5, H-6α, H-6β
20	14.0, CH_3	1.08 (3H, s)	C-1, C-5, C-9, C-10	H-1β, H-2β, H-6β

[a] ^{1}H NMR (599.58) and ^{13}C NMR (150.76 MHz) data obtained with samples in $CDCl_3$. [b] Assignments based on HSQC and HMBC experiments.
[c] Multiplicities reported as apparent splittings: s = singlet, d = doublet, t = triplet, sep = septet, m = multiplet (incl. overlapping resonances), br = broad. α denotes hydrogen pointing into the plane and β denotes hydrogen pointing out of the plane.

Ref: Fang et al 2022: Fang, et al., *Journal of the American Chemical Society* **2022**, *144*, 2292-2300.
