# Supplementary material for: Author Correction: Tripterygium wilfordii cytochrome P450s catalyze the methyl shift and epoxidations in the biosynthesis of triptonide
Source: Nat Commun. 2025 Jul 24;16:6811. doi: 10.1038/s41467-025-62209-8 (PMC12290090; doi:10.1038/s41467-025-62209-8)
Supplement: Supplementary file 2 — Original, uncorrected Figs. 1, 2, 4 [file 41467_2025_62209_MOESM2_ESM.pdf]

Supplementary Information to Author Correction: *Tripterygium wilfordii* cytochrome P450s catalyze the methyl shift and epoxidations in the biosynthesis of triptonide

Kenneth T. Kongstad, Nikolaj Lervad Hansen, Louise Kjaerulff, Quinn Kalby Heck, Victor Forman, Dan Staerk, Birger Lindberg Møller & Johan Andersen-Ranberg

Author Correction to: *Nature Communications* https://doi.org/10.1038/s41467-022-32667-5, published online 25 August 2022.

Original, uncorrected Figs. 1, 2, 4

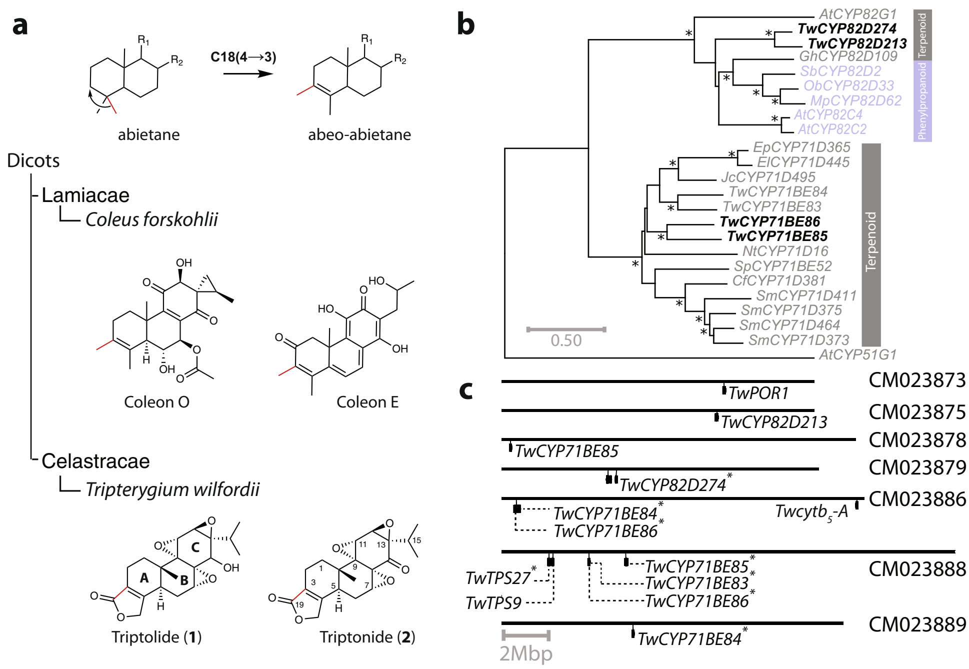

Original Fig. 1

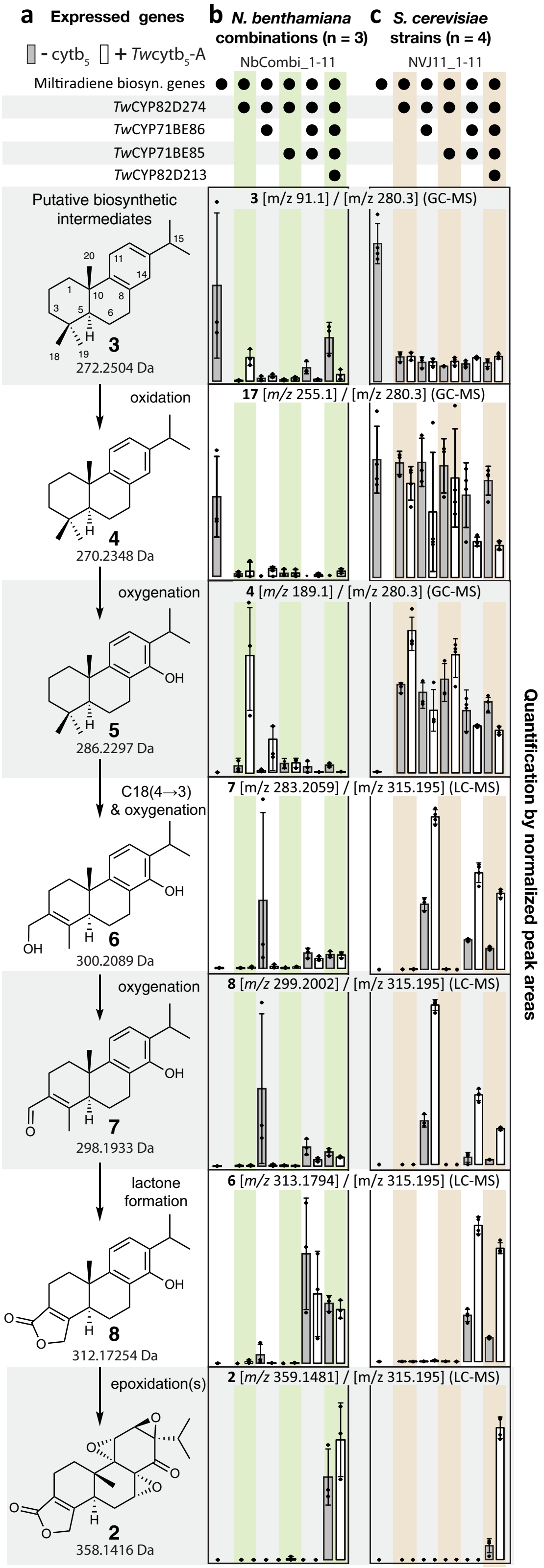

Original Fig. 2

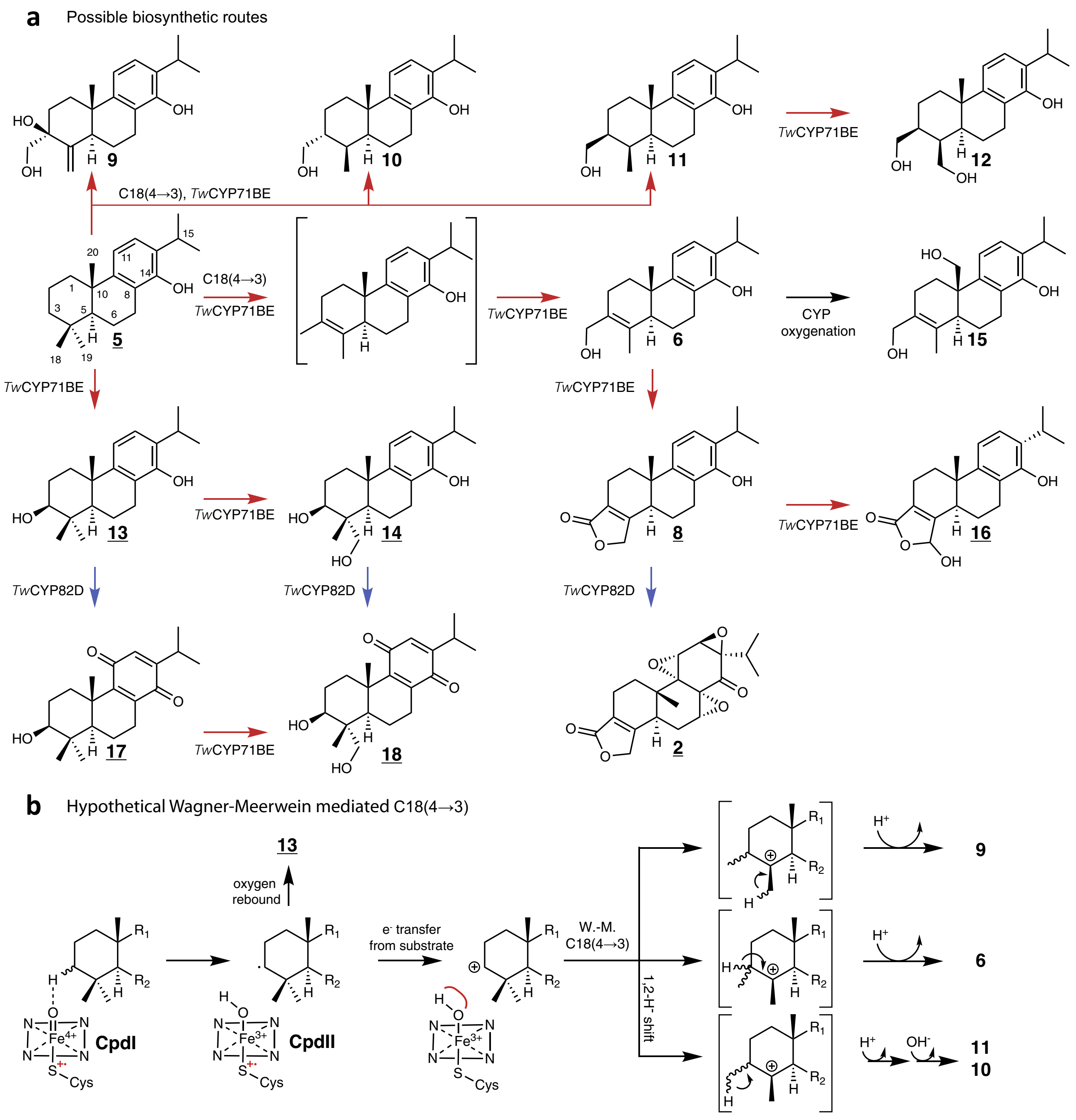

Original Fig. 4
